# Supplementary material for: Hemiphosphoindigos as a platform for chiroptical or water soluble photoswitching
Source: Nat Commun. 2025 Feb 19;16:1760. doi: 10.1038/s41467-025-56942-3 (PMC11840110; doi:10.1038/s41467-025-56942-3)
Supplement: Supplementary file 1 — Supplementary Information [file 41467_2025_56942_MOESM1_ESM.pdf]

Supplementary Information

Hemiphosphoindigos as a Platform for Chiroptical or Water Soluble Photoswitching

*Fabien Kohl, Theresa Vogl, Frank Hampel, Henry Dube**

Friedrich-Alexander Universität Erlangen-Nürnberg, Department of Chemistry and Pharmacy, Nikolaus-Fiebiger-Str. 10, 91058 Erlangen, Germany

* E-mail: henry.dube@fau.de

Table of Contents

Supplementary Note 1: Materials and Methods 4

Supplementary Note 2: HPI synthesis 6

Supplementary Note 3: Water solubility 40

Supplementary Note 4: Thermal isomerization of HPIs 40

1.1.1 Thermal isomerization of HPI 1 43

1.1.2 Thermal isomerization of HPI 1-OH 44

1.1.3 Thermal isomerization of HPI 2 45

1.1.4 Thermal isomerization of HPI 2-OH 46

1.1.5 Thermal isomerization of HPI 3 47

1.1.6 Thermal isomerization of HPI 3-OH 48

1.1.7 Thermal isomerization of HPI 4 49

1.1.8 Thermal isomerization of HPI 5 50

1.1.9 Thermal isomerization of HPI 6 51

1.1.10 Thermal isomerization of HPI 7 52

1.1.11 Thermal isomerization of HPI 8 53

1.1.12 Thermal isomerization of HPI 9 54

1.1.13 Thermal isomerization of HPI 10 55

1.1.14 Thermal isomerization of HPI 11 56

1.1.15 Thermal isomerization of HPI 11-OH 57

1.1.16 Thermal isomerization of HPI 12 58

1.1.17 Thermal isomerization of HPI 13 59

1.1.18 Thermal isomerization of HPI 14 60

1.1.19 Thermal isomerization of HPI 15 61

1.1.20 Thermal isomerization of HPI 16 62

1.1.21 Thermal isomerization of HPI 17 63

1.1.22 Thermal isomerization of HPI 17-OH 64

1.1.23 Thermal isomerization of HPI 18 65

1.1.24 Summary of all thermal isomerization reactions 66

Supplementary Note 5: Photochromism of HPIs 68

1.1.25 Determination of molar absorption coefficients of twisted and heterocyclic HPIs 68

1.1.26 Determination of molar absorption coefficients of planar HPIs 70

1.1.27 Comparison of HPI, HI and HTI 80

Supplementary Note 6: ECD spectra of the pure *Z* isomers and after light irradiation 82

Supplementary Note 7: Theoretical studies of HPI 3, 6 and 10 85
1.1.28 Ground state energies 85
1.1.29 UV and ECD spectra calculations 87
1.1.30 TDDFT Functional Screening 87
1.1.31 Optimized UV and ECD spectra calculations 97
Supplementary Note 8: Photoisomerization reactions in toluene 101
1.1.32 Photoisomerization of HPI 1 105
1.1.33 Photoisomerization of HPI 2 106
1.1.34 Photoisomerization of HPI 3 107
1.1.35 Photoisomerization of HPI 4 108
1.1.36 Photoisomerization of HPI 5 109
1.1.37 Photoisomerization of HPI 6 110
1.1.38 Photoisomerization of HPI 7 111
1.1.39 Photoisomerization of HPI 8 112
1.1.40 Photoisomerization of HPI 9 113
1.1.41 Photoisomerization of HPI 10 114
1.1.42 Photoisomerization of HPI 11 115
1.1.43 Photoisomerization of HPI 12 116
1.1.44 Photoisomerization of HPI 13 117
1.1.45 Photoisomerization of HPI 14 118
1.1.46 Photoisomerization of HPI 15 119
1.1.47 Photoisomerization of HPI 16 120
1.1.48 Photoisomerization of HPI 17 121
1.1.49 Photoisomerization of HPI 18 122
Supplementary Note 9: Photoisomerization reactions in water 123
Supplementary Note 10: Photoisomerization reactions of hydrolyzed derivatives in water 140
Supplementary Note 11: Summary of all photoisomerization experiments 148
Supplementary Note 12: Photofatigue experiments 150
Supplementary Note 13: Quantum yield determination 154
Supplementary Note 14: Error determination 162
Supplementary Note 15: NMR-spectra 165
Supplementary Note 16: Crystal structure analysis 217
Supplementary Note 17: Calculated structures for heterocyclic derivatives 225
Supplementary References 226

Supplementary Note 1: Materials and Methods

Reagents and solvents were obtained from *abcr*, *Acros Organics*, *Fluka*, *Merck* or *Tokyo Chemical Industry* in the qualities *puriss*., *p.a.* or *purum* and used as received. Solvents of technical purity were further purified by distillation with a rotary evaporator (*Heidolph Hei-VAP A*) before use for chromatography or extraction. Anhydrous solvents purchased from *Merck* and were used without further purification. Reaction progress monitoring of all reactions was performed by thin-layer chromatography (TLC) using aluminum plates coated with SiO_2 (*Merck 60, F-254*). Detection was done by irradiation with UV light (254 nm or 366 nm) in order to determine the retardation factors (R*f*).

Medium Pressure Liquid Chromatography (MPLC) was performed on a *Biotage Selekt* with prepacked *Chromabond Flash* SiO_2 (particle size 40-63 µm) columns from *Macherey-Nagel*.

^{1}H NMR and **^{13}C NMR** spectra were recorded on a *Bruker Avance NEO HD* 400 MHz, *Bruker Avance Neo HDX* 500 MHz, *Bruker Avance Neo HDX* 600 MHz with cryo probe DCH-Z^{13}C/^{1}H spectrometer. Deuterated solvents were obtained from *Deutero GmbH* and *Eurisotop* and used without further purification. Chemical shifts (δ) are denoted in parts per million (ppm) relative to tetramethylsilane as external standard. Residual solvent signals in the ^{1}H and ^{13}C NMR spectra were used as internal reference. $CDCl_3$: δ_H = 7.26 ppm, δ_C= 77.16 ppm; toluene-d_8: δ_H= 2.08 ppm, δ_C= 20.43 ppm; DMSO-d_6: δ_H= 2.50 ppm, δ_C= 39.5 ppm, *p*-xylene-d_{10}: δ_H =2.108 ppm. The resonance multiplicity is marked as s (singlet), d (doublet), t (triplet), q (quartet) and m (multiplet). The chemical shifts are given in parts per million (ppm) on the delta scale (δ) and the coupling constant values (J) are given in Hertz (Hz).

High Performance Liquid Chromatography (HPLC) was performed on a Shimadzu HPLC system consisting of a LC-20AP solvent delivery module, a CTO-20A column oven, a SPD-M20A photodiode array UV/vis detector and a CBM-20A system controller using a preparative *Chiralpak* ID column (particle size 5 µm) from *Daicel* and HPLC grade solvents (*n*-hexane, isopropanol, dichloromethane, methanol and ethyl acetate) from *Sigma-Aldrich*, *VWR* and *ROTH*.

Supercritical Fluid Chromatography (SFC) was performed on a *Waters- Prep 15* equipped with a *Waters 2998* photodiode array detector, using MeOH as eluent and *Chiralpak IC* (*Daicel Corporation*, particle size 5 µm, 10 mm x 250 mm) and *Viridis Silica OBD* (*Daicel Corporation*, particle size 5 µm, 10 mm x 250 mm) columns.

Mass spectrometry: Atmospheric pressure photoionization (APPI) and High Resolution Electrospray Ionization mass spectra were recorded on a MicrOTOF II spectrometer. The molecular ion mass is described as *M*.

Infrared spectra were recorded on a *Thermo Fisher Nicolet i5 FT-IR* spectrometer equipped with a *Thermo Fisher Nicolet iD7 ATR* device. Transmittance values are qualitatively described

by wavenumber (cm^{-1}) as very strong (vs), strong (s), medium (m), weak (w) and very weak (vw).

UV/Vis spectra were measured on an *Agilent Technologies Cary 60* spectrophotometer. The spectra were recorded in a quartz cuvette (pathlength 1 cm). Spectral grade solvents were obtained from *VWR* and *Merck*. Absorption wavelengths (λ) are reported in nm and the molar absorption coefficients (ε) in $L \cdot mol^{-1} \cdot cm^{-1}$.

Electronic circular dichroisom (ECD) spectra were measured on a *Chirascan V100* spectrometer from *Applied Photophysics*.

Melting points (M.p.) were measured on a *Büchi M-560 melting point* apparatus in open capillaries.

Photoisomerization experiments: Irradiations of solutions were conducted either in NMR tubes in different deuterated solvents (toluene-d_8) or in UV/vis cuvettes. Photoproducts were then either detected by ^1H NMR or absorption spectroscopy. Irradiations experiments were conducted using LEDs from Thorlabs, Inc (340 nm/ 69.2 mW, 365 nm/ 1400 mW, 395 nm/ 2050 mW, 405 nm/ 1700 mW, 430 nm/ 757.6 mW, 450 nm / 3041.5 mW, 470 nm/ 1161.7 mW, 505 nm/ 520 mW, 530 nm/ 480 mW, 565 nm/ 979 mW) and Roithner Lasertechnik GmbH (420 nm with an output power of 130 mW). For irradiation in NMR tubes the light sources were pointed directly at the NMR tube while maintaining a constant temperature, if needed using a transparent cooling bath.

X-ray diffraction of single crystals was performed on a *SuperNova Atlas* diffractometer using Cu-Kα-radiation.

Supplementary Note 2: HPI synthesis

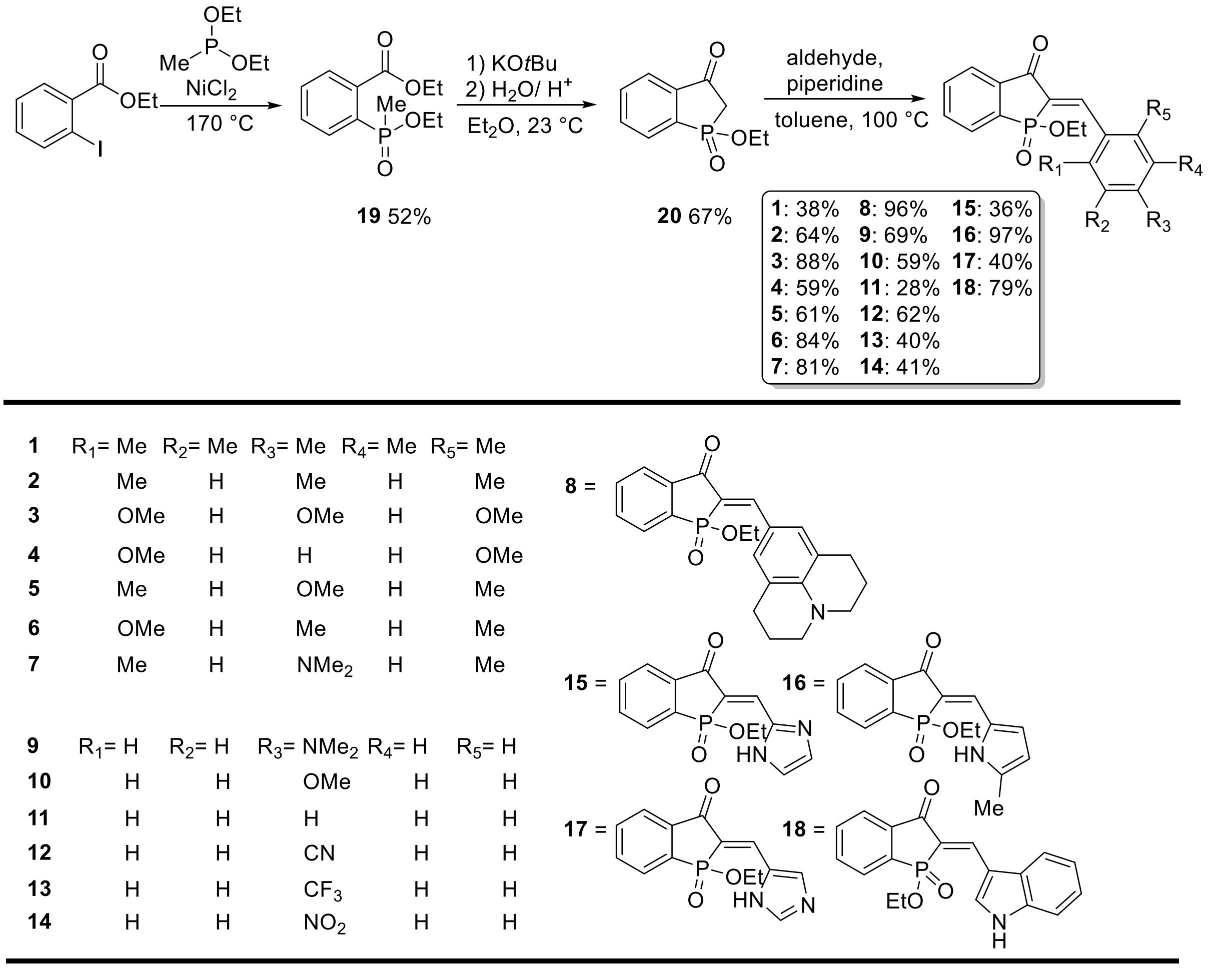

Supplementary Figure 1. General scheme for the synthesis all HPIs. Synthesis of twisted HPIs (**1** to **7**), planar *para*-substituted HPIs (**8** to **14**) and heteroaromatic HPIs (**15** to **18**).

Precursor synthesis

Precursors ethyl 2-(ethoxy(methyl)phosphoryl)benzoate **19**,[1] and 1-ethoxy-2-hydrophosphindol-3-one-1oxide **20**[2] were prepared according to adapted literature known procedures. For the sake of completeness, the small scale procedures are included below.

Ethyl 2-(ethoxy(methyl)phosphoryl)benzoate (19)

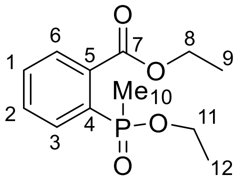

Ethyl 2-iodobenzoate (1.97 mL, 12.1 mmol, 1.00 equiv.) and $NiCl_2$ (314 mg, 2.42 mmol, 0.200 equiv.) were set under argon atmosphere in an oven-dried two-neck Schlenk flask. The mixture was heated to 165-170 °C. After reaching the desired temperature, the reaction flask was connected to a dry ice cooled receiver flask using a glass tube. Then, diethyl methyl phosphonite (2.00 mL, 13.3 mmol, 1.10 equiv.) was added dropwise over the course of 20 min under vigorous stirring, while generated and evaporating ethyl iodide was collected in the receiver flask. The mixture was further stirred at 170 °C for 30 min, after which no more evaporation could be observed. The reaction mixture was cooled to 23 °C and the sticky, yellow crude product was diluted with water (15 mL) and an aqueous HCl solution (2 M, 100 mL) was added. The aqueous phase was extracted with CH_2Cl_2 (3×150 mL) and the combined organic phases were dried over Na_2SO_4 and concentrated. FCC (SiO_2, CH_2Cl_2/MeOH = 99/1 → 98/2 → 96/4) afforded phosphoryl benzoate **19** (1.55 g, 6.05 mmol, 52%) as colorless oil.

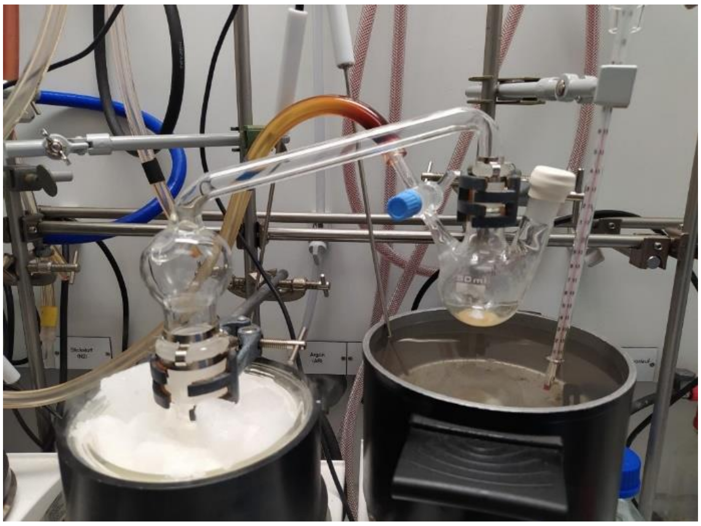

Supplementary Figure 2. Small scale distillation setup for the nickel-catalyzed Arbuzov reaction. It is also possible to use a cannula instead of the glass tube for connecting the two flasks.

^{1}H NMR (400 MHz, $CDCl_3$): *δ* (ppm) = 8.10 – 8.03 (m, 1H, H-C(3)), 7.78 – 7.73 (m, 1H, H-C(6)), 7.62 – 7.56 (m, 2H, H-C(1,2)), 4.40 (q, *J* = 7.1 Hz, 2H, H-C(8)), 4.14 – 3.78 (m, 2H, H-C(11)), 1.88 (d, *J* = 15.5 Hz, 3H, H-C(10)), 1.41 (t, *J* = 7.1 Hz, 3H, H-C(9)), 1.29 (t, *J* = 7.1 Hz, 3H, H-C(12)).

^{13}C NMR (101 MHz, $CDCl_3$): *δ* (ppm) = 168.2 (d, *J* = 3.4 Hz, C(7)), 135.9 (d, *J* = 7.9 Hz, C(5)), 134.1 (d, *J* = 8.2 Hz, C(3)), 132.0 (d, *J* = 2.2 Hz, C(1)), 131.6 (d, *J* = 120.5 Hz, C(4)), 131.1 (d, *J* = 11.3 Hz, C(2)), 129.7 (d, *J* = 9.7 Hz, C(6)), 62.0 (C(8)), 60.9 (d, *J* = 6.2 Hz, C(11)), 17.4 (d, *J* = 104.3 Hz, C(10)), 16.5 (d, *J* = 6.5 Hz, C(12)), 14.3 (C(9)).

^{31}P NMR (162 MHz, $CDCl_3$): *δ* (ppm) = 42.10, 30.49 (impurity).

Note: The product contains a small amount of an inseparable, unknown, phosphorus containing impurity.

IR: $\tilde{\nu}$/cm^{-1} = 3454 (w), 3433 (w), 3404 (w), 2983 (w), 2937 (w), 2904 (w), 1720 (s), 1685 (w), 1676 (w), 1670 (w), 1655 (w), 1647 (w), 1637 (w), 1589 (w), 1570 (w), 1473 (w), 1458 (w), 1439 (w), 1419 (w), 1390 (w), 1367 (m), 1281 (s), 1255 (s), 1201 (s), 1122 (s), 1097 (m), 1068 (s), 1028 (vs), 957 (s), 887 (s), 854 (m), 791 (s), 744 (s), 702 (m), 650 (m), 633 (m), 607 (m), 594 (m), 575 (m), 553 (m), 513 (s), 496 (s), 457 (s), 444 (s), 418 (s).

HR-MS (APPI) for $[C_{12}H_{18}O_4P]^+$, $[M+H]^+$, calcd. 257.0937, found 257.0943.

1-ethoxy-2-hydrophosphindol-3-one-1oxide (20)

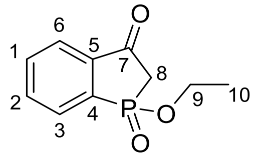

Phosphoryl benzoate **19** (1.46 g, 5.70 mmol, 1.00 equiv.) was dissolved in diethyl ether (67 mL) and KO*t*Bu (1.92 g, 17.1 mmol, 3.00 equiv.) was added at 23 °C under vigorous stirring. Upon base addition, the mixture turned yellow and precipitation was observed. After stirring the mixture at 23 °C for 45 min, an aqueous solution of HCl (2 M, 70 mL) was added. The aqueous phase was extracted with CH_2Cl_2 (3×150 mL) and the combined organic phases were dried over Na_2SO_4 and concentrated. FCC (SiO_2, CH_2Cl_2/MeOH = 99/1 → 98/2) afforded hydrophosphindol **20** (0.800 g, 3.79 mmol, 67%) as white solid.

^{1}H NMR (500 MHz, $CDCl_3$): δ (ppm) = 8.01 (ddt, J = 7.8, 3.2, 1.0 Hz, 1H, H-C(6)), 7.94 (ddt, J = 11.3, 7.5, 1.0 Hz, 1H, H-C(3)), 7.82 (tdd, J = 7.4, 3.5, 1.2 Hz, 1H, H-C(2)), 7.75 (tt, J = 7.5, 1.4 Hz, 1H, H-C(1)), 4.36 – 4.18 (m, 2H, H-C(9)), 3.03 – 2.88 (m, 2H, H-C(8)), 1.38 (t, J = 7.1 Hz, 3H, H-C(10)).

^{13}C NMR (126 MHz, $CDCl_3$): δ (ppm) = 192.9 (d, J = 19.3 Hz, C(7)), 141.4 (d, J = 15.7 Hz, C(5)), 139.5 (d, J = 119.5 Hz, C(4)), 135.6 (d, J = 11.9 Hz, C(2)), 133.7 (d, J = 2.5 Hz, C(1)), 127.9 (d, J = 4.8 Hz, C(3)), 124.8 (d, J = 13.8 Hz, C(6)), 62.6 (d, J = 6.4 Hz, C(9)), 36.0 (d, J = 101.1 Hz, C(8)), 16.7 (d, J = 6.4 Hz, C(10)).

^{31}P NMR (203 MHz, $CDCl_3$): δ (ppm) = 42.37.

IR: $\tilde{\nu}$/cm^{-1} = 2989 (w), 2954 (w), 2910 (w), 1707 (s), 1670 (w), 1587 (m), 1448 (w), 1396 (w), 1371 (m), 1319 (w), 1273 (m), 1227 (vs), 1176 (m), 1149 (w), 1130 (s), 1105 (w), 1070 (w), 1018 (s), 1011 (s), 958 (vs), 845 (m), 804 (m), 773 (s), 766 (s), 754 (s), 725 (m), 692 (m), 584 (w), 571 (m), 542 (s), 488 (vs), 432 (m), 426 (m), 418 (m).

M.p.: 81 °C.

HR-MS (APPI) for $[C_{10}H_{12}O_3P]^+$, $[M+H]^+$, calcd. 211.0519, found 211.0519.

R_f (SiO_2, CH_2Cl_2/MeOH = 98/2) = 0.38.

Aldehyde synthesis

Most of the aldehydes employed in this work were commercially available and aldehydes **21-22**[3], **23**[4] and **24**[5-6] were synthesized following adapted literature known procedures (Supplementary Figure 3), which are included below for the sake of completeness.

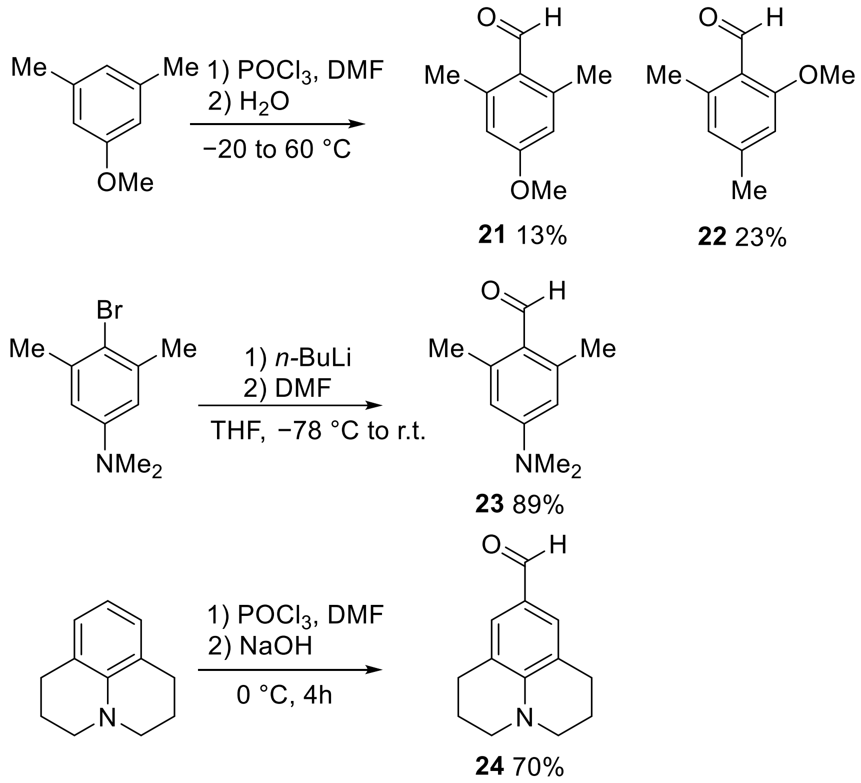

Supplementary Figure 3. Synthesis of aldehydes **21-24** following literature known procedures.

4-methoxy-2,6-dimethylbenzaldehyde (21) and 2-methoxy-4.6-dimethylbenzaldehyde (22)

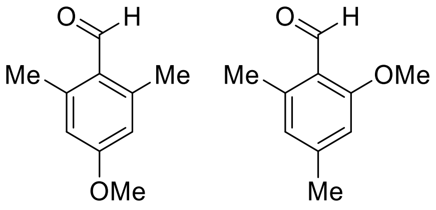

21 **22**

$POCl_3$ (3.78 mL, 40.4 mmol, 1.10 equiv.) was dissolved in DMF (9.95 mL, 128 mmol, 3.50 equiv.) and cooled to –20 °C. 3,5-dimethylanisole (5.19 mL, 36.7 mmol, 1.00 equiv.) was added dropwise at that temperature. After addition, the mixture was stirred at 20 °C for 1 h and at 60 °C for 40 h. The reaction mixture was poured on ice and NaOH (1 M) was added until pH=6 was reached. The aqueous phase was extracted with ethyl acetate (3×150 mL) and the combined organic phases were dried over Na_2SO_4, filtered and concentrated. FCC (SiO_2, *i*-hexane/EtOAc = 99/1 → 98/2) afforded one fraction of aldehyde **21** (0.803 g, 4.89 mmol, 13%) and one fraction of aldehyde **22** (1.40 g, 8.53 mmol, 23%), both as white solids.

21

^{1}H NMR (400 MHz, $CDCl_3$): δ (ppm) = 10.48 (s, 1H), 6.59 (s, 2H), 3.84 (s, 3H), 2.61 (s, 6H). These values agree with those reported in the literature.[3]

R_f (SiO_2, *i*-hexane/EtOAc = 98/2) = 0.26.

22

^{1}H NMR (400 MHz, $CDCl_3$): δ (ppm) = 10.58 (s, 1H), 6.63 (s, 1H), 6.62 (s, 1H), 3.88 (s, 3H), 2.54 (s, 3H), 2.35 (s, 3H). These values agree with those reported in the literature.[3]

R_f (SiO_2, *i*-hexane/EtOAc = 98/2) = 0.34.

4-(dimethylamino)-2,6-dimethylbenzaldehyde (23)

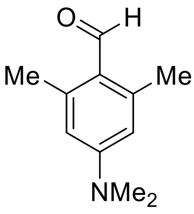

23

Under argon atmosphere, 4-bromo-*N*, *N*-3,5-tetramethylanilin (0.500 g, 2.19 mmol, 1.00 equiv.) was dissolved in anhydrous THF (10.9 mL) and the solution was cooled to –78 °C. *n*-BuLi (2.36 M, 1.45 mL, 3.43 mmol, 1.60 equiv.) was added dropwise at that temperature and the mixture was stirred at –78 °C for 1 h. Anhydrous DMF (0.266 mL, 3.43 mmol, 1.60 equiv.) was added dropwise and the mixture was further stirred at –78 °C for 15 min, allowed to warm up to 23 °C and stirred for 30 min. A saturated aqueous solution of ammonium chloride (11.0 mL) was added and the aqueous phase was extracted with CH_2Cl_2 (3×100 mL). The combined organic phases were dried over Na_2SO_4, filtered, and concentrated. FCC (SiO_2, *i*-hexane/EtOAc = 98/2 → 9/1 → 17/3) afforded aldehyde **23** (0.345 g, 1.95 mmol, 89%) as white solid.

^{1}H NMR (400 MHz, $CDCl_3$): δ (ppm) = 10.35 (s, 1H), 6.31 (s, 2H), 3.04 (s, 6H), 2.59 (s, 6H). These values agree with those reported in the literature.[4]

IR: $\tilde{\nu}$/cm^{-1} = 2974 (m), 2906 (m), 2883 (m), 2868 (m), 2817 (m), 2777 (m), 1664 (m), 1649 (s), 1587 (s), 1560 (s), 1541 (s), 1535 (s), 1522 (s), 1479 (m), 1454 (m), 1439 (m), 1421 (s), 1383 (m), 1352 (s), 1323 (s), 1267 (s), 1242 (s), 1221 (s), 1146 (s), 1122 (s), 1070 (s), 1047 (s), 1034 (s), 987 (m), 976 (m), 960 (m), 877 (m), 822 (vs), 781 (s), 712 (s), 625 (s), 611 (m), 577 (m), 553 (s), 532 (m), 501 (s), 453 (m), 442 (m), 432 (m), 418 (m), 409 (m).

M.p.: 85 °C.

R*f* (SiO_2, *i*-hexane/EtOAc = 95/5) = 0.16.

9-formyljulolidine (24)

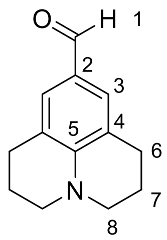

24

Julolidine (1.50 g, 8.66 mmol, 1.00 equiv.) was dissolved in DMF (3.80 mL, 48.9 mmol, 5.65 equiv.) and cooled to 0 °C. $POCl_3$ (0.840 mL, 9.00 mmol, 1.04 equiv.) was then added dropwise and the resulting solution was further stirred at 0 °C for 4 h. The reaction mixture was then poured on ice/water (150 mL) and pH=10 was adjusted using an aqueous solution of NaOH (2 M). The aqueous phase was extracted with EtOAc (3×150 mL), the combined organic phases were dried over Na_2SO_4, and concentrated. FCC (SiO_2, CH_2Cl_2/MeOH = 98/2 → 9/1) afforded aldehyde **24** (1.22 g, 6.06 mmol, 70%) as yellow solid.

^{1}H NMR (600 MHz, CD_2Cl_2): δ (ppm) = 9.55 (s, 1H, H-C(1)), 7.24 (s, 2H, H-C(3)), 3.29 – 3.26 (m, 4H, H-C(8)), 2.75 (t, J = 6.3 Hz, 4H, H-C(6)), 1.97 – 1.92 (m, 4H, H-C(7)).

^{13}C NMR (151 MHz, CD_2Cl_2): δ (ppm) = 190.0 (C(1)), 148.3 (C(5)), 129.5 (C(3)), 124.4 (C(2)), 120.8 (C(4)), 50.4 (C(8)), 28.1 (C(6)), 21.7 (C(7)).

These values agree with those reported in the literature.[5-6]

HR-MS (APPI) for $[C_{13}H_{16}NO]^+$, $[M+H]^+$, calcd. 202.1226, found 202.1226.

IR: $\tilde{\nu}$/cm^{-1} = 2945 (m), 2937 (m), 2925 (m), 2887 (m), 2854 (m), 2823 (m), 1651 (s), 1591 (vs), 1564 (s), 1523 (s), 1454 (s), 1433 (s), 1417 (s), 1398 (m), 1313 (vs), 1277 (s), 1265 (s), 1238 (s), 1203 (s), 1190 (s), 1180 (s), 1142 (vs), 1074 (s), 1049 (s), 1011 (s), 982 (s), 914 (s), 897 (vs), 879 (s), 864 (s), 818 (m), 806 (s), 750 (s), 733 (vs), 656 (m), 642 (s), 613 (s), 590 (s), 559 (s), 523 (s), 515 (s), 499 (s), 478 (s), 457 (s), 438 (s), 428 (s).

M.p.: 85 °C.

R_f (SiO_2, CH_2Cl_2/MeOH = 98/2) = 0.65.

Typical procedure for HPI photoswitch synthesis (TP1)

HPI synthesis proceeds analogously to previously reported Hemithioindigo (HTI) synthesis.[7]

Hydrophosphindol **20** (1.00 equiv.) and the corresponding aldehyde (1.00 equiv.) were dissolved in anhydrous toluene to obtain a 0.2 M solution. After addition of piperidine (2 drops), the mixture was stirred at 100 °C for 1-23 h, while monitoring the reaction progress by thin layer chromatography. The mixture was cooled to 23 °C and a saturated aqueous ammonium chloride solution (30 mL) was added. The aqueous phase was extracted with CH_2Cl_2 (3×80 mL) and the combined organic phases were dried over Na_2SO_4 and concentrated. The product was purified by column chromatography or recrystallization and if necessary further purified by HPLC or supercritical fluid chromatography (SFC). The purification conditions are specified below for each derivative.

Similar to the HTI photoswitches, the synthesis for the HPIs yield primarily the more stable *Z* isomers, in most cases pure *Z* isomers could be isolated. To determine the configuration of the central double bond, NOE experiments were performed for each derivative.

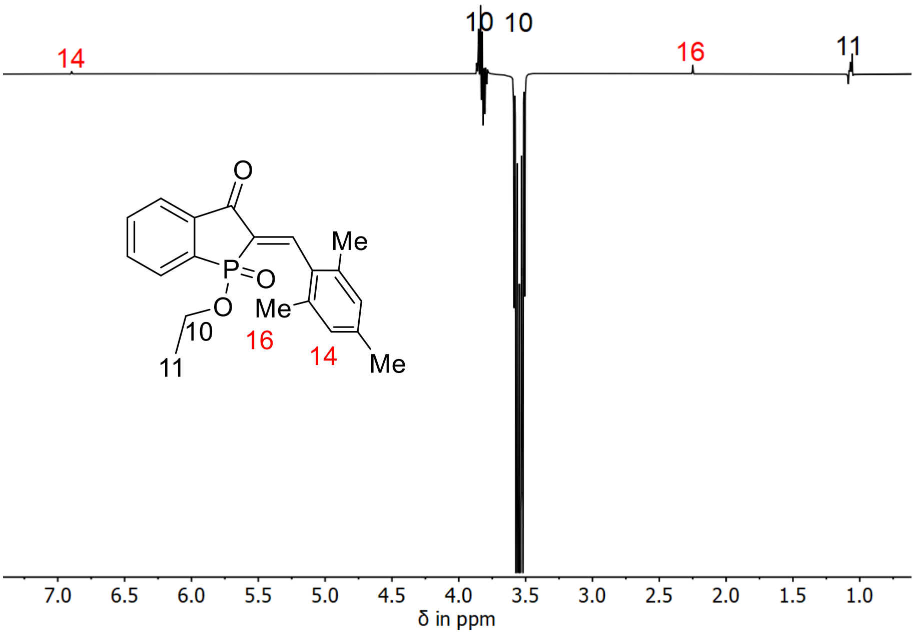

Supplementary Figure 4. Example for the determination of the configuration of the central double bond of HPI **2** with a 1D NOE (500 MHz, toluene-d_8, 25 °C) experiment. Selective irradiation of one proton of the CH_2 group 10 reveals through space coupling to 16 and 14, evidencing *Z* configuration.

(Z)-1-ethoxy-2-(2,3,4,5,6-pentamethylbenzylidene)-2-hydrophosphindol-3-one 1-oxide (1)

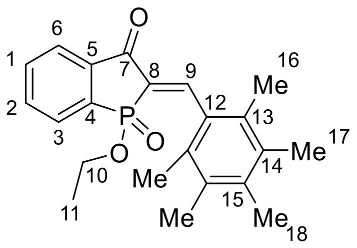

(*Z*)-1-ethoxy-2-(2,3,4,5,6-pentamethylbenzylidene)-2-hydrophosphindol-3-one 1-oxide **1** was synthesized according to **TP1** from hydrophosphindol **20** (0.750 mmol) and pentamethylbenzaldehyde (0.750 mmol). FCC (SiO_2, CH_2Cl_2/MeOH = 99/1 → 98/2) and subsequent recrystallization from CH_2Cl_2 and *n*-heptane afforded **1** as yellow solid (105 mg, 0.285 mmol, 38%).

^{1}H NMR (400 MHz, $CDCl_3$): δ (ppm) = 8.55 (d, *J* = 36.0 Hz, 1H, H-C(9)), 8.18 – 8.10 (m, 1H, H-C(6)), 7.90 – 7.80 (m, 1H, H-C(3)), 7.83 – 7.70 (m, 2H, H-C(1,2)), 3.80 (dp, *J* = 9.9, 7.1 Hz, 1H, H-C(10)), 3.50 (ddq, *J* = 10.0, 8.9, 7.1 Hz, 1H, H-C(10)), 2.26 (s, 3H, H-C(18)), 2.23 (s, 6H, H-C(17)), 2.20 (s, 6H, H-C(16)), 1.05 (t, *J* = 7.1 Hz, 3H, H-C(11)).

^{13}C NMR (101 MHz, $CDCl_3$): δ (ppm) = 185.0 (d, *J* = 43.6 Hz, C(7)), 155.5 (d, *J* = 5.0 Hz, C(9)), 140.5 (d, *J* = 12.0 Hz, C(5)), 138.1 (d, *J* = 126.3 Hz, C(4)), 135.7 (C(15)), 135.4 (d, *J* = 12.0 Hz, C(2)), 133.6 (d, *J* = 2.3 Hz, C(1)), 132.7 (C(14)), 131.6 (d, *J* = 5.5 Hz, C(12)), 131.3 (d, *J* = 138.4 Hz, C(8)), 130.6 (C(13)), 127.7 (d, *J* = 5.1 Hz, C(3)), 125.2 (d, *J* = 11.4 Hz, C(6)), 62.2 (d, *J* = 6.3 Hz, C(10)), 18.3 (C(16)), 17.0 (C(18)), 16.5 (d, *J* = 6.6 Hz, C(11)), 16.3 (C(17)).

^{31}P NMR (162 MHz, $CDCl_3$): δ (ppm) = 28.00.

IR: $\tilde{\nu}$/cm^{-1} = 3059 (w), 2995 (w), 2927 (w), 2902 (w), 1691 (s), 1604 (s), 1585 (m), 1570 (w), 1473 (w), 1458 (m), 1439 (m), 1385 (w), 1325 (w), 1313 (m), 1296 (w), 1273 (m), 1250 (w), 1230 (vs), 1209 (s), 1173 (w), 1155 (w), 1134 (s), 1101 (w), 1057 (m), 1034 (vs), 970 (s), 939 (m), 910 (m), 814 (w), 804 (m), 787 (s), 752 (s), 741 (s), 731 (s), 696 (s), 667 (m), 586 (w), 573 (m), 555 (s), 536 (m), 517 (s), 498 (w), 486 (m), 469 (vs), 422 (w), 409 (w).

M.p.: 197 °C.

HR-MS (APPI) for $[C_{22}H_{26}O_3P]^+$, $[M+H]^+$, calcd. 369.1614, found 369.1627.

R*f* (SiO_2, CH_2Cl_2/MeOH = 98/2) = 0.26.

(*E*)-1-ethoxy-2-(2,3,4,5,6-pentamethylbenzylidene)-2-hydrophosphindol-3-one 1-oxide (*E*-1)

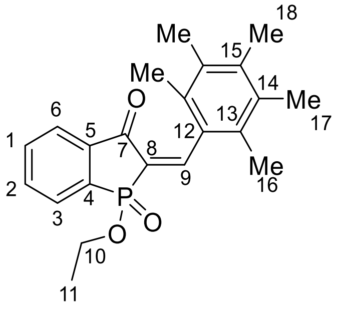

(*E*)-**1** was prepared by irradiation of a toluene solution of (*Z*)-**1** with 395 nm light. The (*E*)-enriched solution was purified by HPLC (*n*-hexane/*i*-PrOH=85/15).

^{1}H NMR (600 MHz, Tol-*d*$_8$): *δ* (ppm) = 7.98 (d, *J* = 18.3 Hz, 1H, H-C(9)), 7.71 – 7.67 (m, 1H, H-C(6)), 7.56 (ddt, *J* = 11.4, 7.4, 1.0 Hz, 1H, H-C(3)), 7.00 – 6.97 (m, 1H, H-C(2)), 6.92 (tt, *J* = 7.5, 1.2 Hz, 1H, H-C(1)), 4.07 – 3.99 (m, 2H, H-C(10)), 2.17 (s, 3H, H-C(16)), 2.05 (s, 3H, H-C(17)), 2.01 (s, 3H, H-C(18)), 1.96 (s, 3H, H-C(17)), 1.10 (t, *J* = 7.0 Hz, 3H, H-C(11)).

^{13}C NMR (151 MHz, Tol-*d*$_8$): *δ* (ppm) = 183.8 (d, *J* = 41.7 Hz, C(7)), 153.5 (d, *J* = 6.9 Hz, C(9)), 142.1 (d, *J* = 12.4 Hz, C(5)), 136.9 (d, *J* = 122.9 Hz, C(4)), 135.6 (C(15)), 134.3 (d, *J* = 11.8 Hz, C(2)), 133.0 (d, *J* = 2.3 Hz, C(1)), 132.4 (C(14)), 132.1 (C(14)), 132.0 (d, *J* = 14.7 Hz, C(12)), 131.1 (C(13)), 130.8 (C(13)), 130.5 (d, *J* = 132.2 Hz, C(8)), 127.7 (d, *J* = 5.0 Hz, C(3)), 124.6 (d, *J* = 12.0 Hz, C(6)), 61.9 (d, *J* = 6.2 Hz, C(10)), 17.9 (C(16)), 17.7 (C(16)), 16.8 (C(18)), 16.7 (d, *J* = 6.2 Hz, C(11)), 16.3 (C(17)), 16.1 (C(17)).

^{31}P NMR (162 MHz, Tol-*d*$_8$): *δ* (ppm) = 27.65.

Notes: Slow exchange →Two signals for H-C(16) and H-C(17) and C(13), C(14), C(16), C(17). The second signal for the H-C(16) can't be seen due to solvent overlap at 2.08 ppm.

IR: $\tilde{\nu}$/cm^{-1} = 3059 (w), 2981 (w), 2916 (w), 2871 (w), 1695 (s), 1604 (s), 1591 (m), 1566 (w), 1450 (m), 1383 (m), 1331 (m), 1232 (s), 1215 (s), 1163 (m), 1130 (s), 1072 (m), 1065 (m), 1012 (vs), 949 (vs), 777 (s), 744 (vs), 727 (s), 704 (s), 683 (m), 650 (m), 575 (s), 552 (s), 534 (s), 511 (s), 496 (s), 467 (s), 442 (m), 418 (m).

M.p.: 181 °C.

HR-MS (APPI) for $[C_{22}H_{26}O_3P]^+$, $[M+H]^+$, calcd. 369.1614, found 369.1620.

(*Z*)-1-hydroxy-2-(2,3,4,5,6-pentamethylbenzylidene)-2-hydrophosphindol-3-one 1-oxide (1-OH)

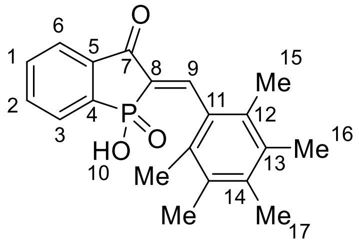

Under argon atmosphere, HPI **1** (40.0 mg, 0.109 mmol, 1.00 equiv.) was dissolved in anhydrous CH_2Cl_2 (1 mL) to obtain a 0.1 M solution. Then trimethylbromosilane (0.029 mL, 0.217 mmol, 2.00 equiv.) was added and the solution was stirred at 23 °C for 5 h. The solvent was removed *in vacuo* and the solid was taken up in methanol (10 mL) and water (3 mL) was added. Parts of the solvent were removed under reduced pressure until a solid precipitated. The precipitate was filtered off and washed with methanol. Drying under high vacuum afforded **1**-OH as yellow solid (25.2 mg, 0.109 mmol, 68%).

^{1}H NMR (600 MHz, DMSO-d_6): δ (ppm) = 8.28 (d, *J*= 34.0 Hz, 1H, H-C(9)), 8.07 – 8.03 (m, 1H, H-C(6)), 7.90 (tdd, *J* = 7.5, 3.2, 1.2 Hz, 1H, H-C(2)), 7.87 – 7.82 (m, 2H, H-C(3, 1)), 2.21 (s, 3H, H-C(17)), 2.16 (s, 6H, H-C(16)), 2.09 (s, 6H, H-C(15)).

^{13}C NMR (151 MHz, DMSO-d_6): δ (ppm) = 185.2 (d, *J* = 41.3 Hz, C(7)), 152.3 (d, *J* = 4.8 Hz, C(9)), 140.7 (d, *J* = 122.4 Hz, C(4)), 138.7 (d, *J* = 11.6 Hz, C(5)), 135.8 (d, *J* = 11.5 Hz, C(2)), 134.4 (C(14)), 133.2 (d, *J* = 2.2 Hz, C(1)), 132.7 (d, *J* = 136.2 Hz, C(8)), 131.6 (C(12/13)), 131.6 (C(12/13)), 129.8 (d, *J* = 0.8 Hz, C(11)), 127.1 (d, *J* = 5.4 Hz, C(3)), 124.4 (d, *J* = 10.7 Hz, C(6)), 17.7 (C(15)), 16.6 (C(17)), 16.0 (C(16)).

^{31}P NMR (162 MHz, DMSO-d_6): δ (ppm) = 20.47.

Notes: The signal for the H-O(10) could not be detected.

IR: $\tilde{\nu}$/cm^{-1} = 2920 (w), 2854 (w), 1701 (s), 1618 (s), 1589 (m), 1570 (m), 1560 (m), 1458 (m), 1450 (m), 1315 (m), 1277 (m), 1211 (s), 1176 (s), 1136 (s), 1063 (s), 1026 (m), 1020 (m), 962 (s), 933 (m), 904 (s), 889 (m), 802 (m), 746 (vs), 735 (s), 690 (vs), 671 (m), 573 (m), 557 (m), 536 (s), 482 (s), 469 (vs), 417 (m).

M.p.: 323 °C.

HR-MS (APPI) for $[C_{20}H_{22}O_3P]^+$, $[M+H]^+$, calcd. 341.1301, found 341.1308.

(*Z*)-1-ethoxy-2-(2,4,6-trimethylbenzylidene)-2-hydrophosphindol-3-one 1-oxide (2)

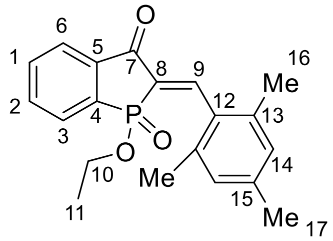

(*Z*)-1-ethoxy-2-(2,4,6-trimethylbenzylidene)-2-hydrophosphindol-3-one 1-oxide **2** was synthesized according to **TP1** from hydrophosphindol **20** (0.813 mmol) and 2,4,6-trimethylbenzaldehyde (0.813 mmol). FCC (SiO_2, CH_2Cl_2/MeOH = 99/1 → 98/2 → 97/3) afforded **2** as yellow solid (176 mg, 0.517 mmol, 64%).

^{1}H NMR (500 MHz, $CDCl_3$): δ (ppm) = 8.43 (d, J = 35.5 Hz, 1H, H-C(9)), 8.15 – 8.12 (m, 1H, H-C(6)), 7.89 – 7.84 (m, 1H, H-C(3)), 7.82 – 7.72 (m, 2H, H-C(1,2)), 6.92 (s, 2H, H-C(14)), 3.85 (dp, J = 10.0, 7.1 Hz, 1H, H-C(10)), 3.57 (ddq, J = 10.1, 9.1, 7.0 Hz, 1H, H-C(10)), 2.30 (s, 3H, H-C(17)), 2.27 (s, 6H, H-C(16)), 1.09 (t, J = 7.0 Hz, 3H, H-C(11)).

^{13}C NMR (126 MHz, $CDCl_3$): δ (ppm) = 184.8 (d, J = 43.2 Hz, C(7)), 153.1 (d, J = 5.1 Hz, C(9)), 140.4 (d, J = 12.4 Hz, C(5)), 138.6 (C(15)), 138.0 (d, J = 127.3 Hz, C(4)), 135.7 (d, J = 1.5 Hz, C(13)), 135.4 (d, J = 11.9 Hz, C(2)), 133.7 (d, J = 2.3 Hz, C(1)), 132.0 (d, J = 137.4 Hz, C(8)), 131.2 (d, J = 5.6 Hz, C(12)), 128.3 (C(14)), 127.7 (d, J = 5.1 Hz, C(3)), 125.2 (d, J = 11.0 Hz, C(6)), 62.4 (d, J = 6.0 Hz, C(10)), 21.3 (C(17)), 20.4 (C(16)), 16.6 (d, J = 6.4 Hz, C(11)).

^{31}P NMR (203 MHz, $CDCl_3$): δ (ppm) = 27.91.

IR: $\tilde{\nu}$/cm^{-1} = 3062 (vw), 2976 (w), 2918 (w), 2856 (vw), 1695 (m), 1618 (s), 1591 (m), 1566 (w), 1481 (w), 1450 (w), 1394 (w), 1379 (w), 1319 (w), 1300 (m), 1277 (w), 1228 (s), 1217 (m), 1207 (m), 1161 (w), 1130 (m), 1103 (w), 1063 (m), 1028 (s), 966 (m), 920 (m), 885 (w), 864 (m), 814 (w), 791 (w), 781 (m), 768 (m), 742 (s), 715 (m), 688 (s), 669 (m), 640 (w), 561 (s), 542 (m), 521 (m), 511 (m), 498 (m), 486 (m), 471 (vs), 444 (w), 418 (m), 413 (m).

M.p.: 170 °C.

HR-MS (APPI) for $[C_{20}H_{22}O_3P]^+$, $[M+H]^+$, calcd. 341.1301, found 341.1312.

R*f* (SiO_2, CH_2Cl_2/MeOH = 98/2) = 0.37.

(*Z*)-1-hydroxy-2-(2,4,6-trimethylbenzylidene)-2-hydrophosphindol-3-one 1-oxide (2-OH)

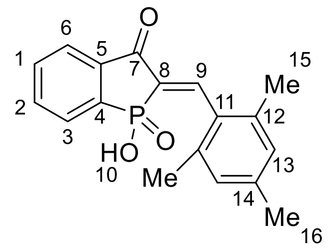

Under argon atmosphere, HPI **2** (22.0 mg, 0.065 mmol, 1.00 equiv.) was dissolved in anhydrous CH_2Cl_2 (0.5 mL) to obtain a 0.1 M solution. Then trimethylbromosilane (0.017 mL, 0.129 mmol, 2.00 equiv.) was added and the solution was stirred at 23 °C for 5 h. The solvent was removed *in vacuo* and the solid was taken up in methanol (5 mL) and water (3 mL) was added. Parts of the solvent were removed under reduced pressure until a solid precipitated. The precipitate was filtered off and washed with methanol. Drying under high vacuum afforded **2**-OH as off-white solid (9.9 mg, 0.065 mmol, 49%).

^{1}H NMR (600 MHz, DMSO-d_6): δ (ppm) = 8.21 (d, J = 33.6 Hz, 1H, H-C(9)), 8.06 – 8.02 (m, 1H, H-C(6)), 7.90 (tdd, J = 7.4, 3.3, 1.1 Hz, 1H, H-C(2)), 7.88 – 7.81 (m, 2H, H-C(3,1)), 6.90 (s, 2H, H-C(13)), 2.26 (s, 3H, H-C(16)), 2.17 (s, 6H, H-C(15)).

^{13}C NMR (151 MHz, DMSO-d_6): δ (ppm) = 185.1 (d, J = 41.0 Hz, C(7)), 150.0 (C(9)), 140.9 (d, J = 120.4 Hz, C(4)), 138.6 (d, J = 11.5 Hz, C(5)), 137.1 (C(14)), 135.9 (d, J = 11.4 Hz, C(2)), 134.9 (C(12)), 133.5 (d, J = 136.0 Hz, C(8)), 133.2 (C(1)), 131.4 (d, J = 5.6 Hz, C(11)), 127.7 (C(13)), 127.2 (d, J = 5.5 Hz, C(13)), 124.4 (d, J = 10.7 Hz, C(6)), 20.7 (C(16)), 19.9 (C(15)).

^{31}P NMR (162 MHz, DMSO-d_6): δ (ppm) = 19.98.

Notes: The signal for the H-O(10) could not be detected.

IR: $\tilde{\nu}$/cm^{-1} = 2941 (w), 2914 (w), 2563 (w), 2256 (w), 1695 (s), 1626 (s), 1591 (s), 1483 (m), 1454 (m), 1327 (m), 1308 (s), 1279 (s), 1221 (s), 1209 (s), 1165 (s), 1132 (s), 1074 (s), 1059 (s), 1032 (s), 964 (s), 949 (vs), 912 (s), 860 (s), 796 (s), 787 (s), 773 (m), 741 (vs), 715 (s), 688 (vs), 669 (s), 642 (m), 555 (s), 536 (s), 519 (s), 496 (s), 486 (s), 472 (vs), 447 (s).

M.p.: 307 °C.

HR-MS (APPI) for $[C_{18}H_{18}O_3P]^+$, $[M+H]^+$, calcd. 313.0988, found 313.0987.

(*Z*)-1-ethoxy-2-(2,4,6-trimethoxybenzylidene)-2-hydrophosphindol-3-one 1-oxide (3)

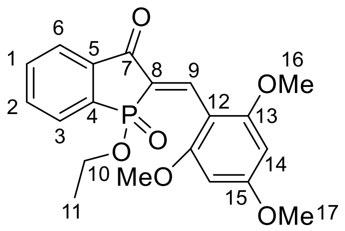

(*Z*)-1-ethoxy-2-(2,4,6-trimethoxybenzylidene)-2-hydrophosphindol-3-one 1-oxide **3** was synthesized according to **TP1** from hydrophosphindol **20** (0.750 mmol) and 2,4,6-trimethoxybenzaldehyde (0.750 mmol). FCC (SiO_2, CH_2Cl_2/MeOH = 99/1 → 98/2) afforded **3** as yellow solid (257 mg, 0.662 mmol, 88%).

^{1}H NMR (400 MHz, $CDCl_3$): *δ* (ppm) = 8.80 (d, *J* = 36.9 Hz, 1H, H-C(9)), 8.05 (dd, *J* = 7.5, 3.3 Hz, 1H, H-C(6)), 7.90 (dd, *J* = 10.4, 7.3 Hz, 1H, H-C(3)), 7.73 (tdd, *J* = 7.3, 3.2, 1.1 Hz, 1H, H-C(2)), 7.67 (tt, *J* = 7.4, 1.2 Hz, 1H, H-C(1)), 6.11 (s, 2H, H-C(14)), 3.98 (s, 6H, H-C(16)), 3.89 (s, 3H, H-C(17)), 3.77 (ddt, *J* = 15.0, 9.9, 7.1 Hz, 1H, H-C(10)), 3.67 – 3.56 (m, 1H, H-C(10)), 1.14 (t, *J* = 7.1 Hz, 3H, H-C(11)).

^{13}C NMR (101 MHz, $CDCl_3$): *δ* (ppm) = 188.1 (d, *J* = 45.2 Hz, C(7)), 166.7 (C(15)), 162.9 (C(13)), 143.6 (d, *J* = 3.4 Hz, C(9)), 140.0 (d, *J* = 11.3 Hz, C(5)), 137.9 (d, *J* = 124.4 Hz, C(4)), 134.7 (d, *J* = 11.6 Hz, C(2)), 132.8 (d, *J* = 2.2 Hz, C(1)), 127.5 (d, *J* = 4.9 Hz, C(3)), 124.3 (d, *J* = 10.8 Hz, C(6)), 117.9 (d, *J* = 146.0 Hz, C(8)), 106.3 (d, *J* = 2.9 Hz, C(12)), 90.4 (C(14)), 62.4 (d, *J* = 6.2 Hz, C(10)), 55.9 (C(16)), 55.8 (C(17)), 16.2 (d, *J* = 8.1 Hz, C(11)).

^{31}P NMR (162 MHz, $CDCl_3$): *δ* (ppm) = 29.15.

IR: $\tilde{\nu}$/cm^{-1} = 3082 (w), 3022 (w), 2985 (w), 2972 (w), 2939 (w), 2895 (w), 2843 (w), 1674 (s), 1606 (s), 1589 (m), 1574 (s), 1539 (vs), 1471 (s), 1450 (s), 1431 (m), 1415 (m), 1381 (s), 1340 (s), 1306 (s), 1281 (s), 1254 (w), 1234 (s), 1225 (s), 1211 (s), 1201 (vs), 1161 (s), 1130 (vs), 1095 (m), 1074 (w), 1057 (m), 1038 (vs), 1026 (vs), 1012 (s), 984 (s), 957 (s), 933 (s), 914 (s), 829 (m), 804 (m), 785 (s), 752 (s), 733 (s), 708 (m), 694 (s), 663 (w), 650 (m), 623 (s), 590 (m), 548 (m), 521 (m), 503 (s), 482 (m), 471 (m), 451 (m), 426 (w).

M.p.: 180 °C.

HR-MS (APPI) for $[C_{20}H_{22}O_6P]^+$, $[M+H]^+$, calcd. 389.1149, found 389.1158.

R*f* (SiO_2, CH_2Cl_2/MeOH = 98/2) = 0.37.

(*Z*)-1-hydroxy-2-(2,4,6-trimethoxybenzylidene)-2-hydrophosphindol-3-one 1-oxide (3-OH)

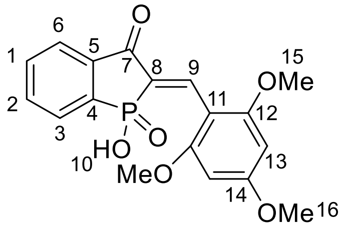

Under argon atmosphere, HPI **3** (40.0 mg, 0.103 mmol, 1.00 equiv.) was dissolved in anhydrous CH_2Cl_2 (1 mL) to obtain a 0.1 M solution. Then trimethylbromosilane (0.027 mL, 0.206 mmol, 2.00 equiv.) was added and the solution was stirred at 23 °C for 5 h. The solvent was removed *in vacuo* and the solid was redissolved in methanol (2 mL) and water (5 drops) was added. The precipitate was filtered off and washed with cold methanol. Drying under high vacuum afforded **3**-OH as yellow solid (35.8 mg, 0.0994 mmol, 96%).

^{1}H NMR (600 MHz, DMSO-*d*$_6$): δ (ppm) = 8.34 (d, *J* = 35.3 Hz, 1H, H-C(9)), 7.91 (dd, *J* = 7.7, 3.2 Hz, 1H, H-C(6)), 7.85 – 7.79 (m, 2H, H-C(2, 3)), 7.75 – 7.71 (m, 1H, H-C(1)), 6.32 (s, 2H, H-C(13)), 3.92 (s, 6H, H-C(15)), 3.91 (s, 3H, H-C(16)).

^{13}C NMR (151 MHz, DMSO-*d*$_6$): δ (ppm) = 187.9 (d, *J* = 43.3 Hz, C(7)), 165.8 (C(14)), 161.8 (C(12)), 141.7 (d, *J* = 124.4 Hz, C(4)), 139.2 (d, *J* = 3.5 Hz, C(9)), 137.9 (d, *J* = 11.3 Hz, C(5)), 134.9 (d, *J* = 11.3 Hz, C(2)), 132.2 (d, *J* = 1.9 Hz, C(1)), 126.7 (d, *J* = 5.2 Hz, C(3)), 123.4 (d, *J* = 10.4 Hz, C(6)), 123.2 (d, *J* = 145.3 Hz, C(8)), 105.1 (d, *J* = 3.0 Hz, C(11)), 90.7 (C(13)), 55.9 (C(15)), 55.9 (C(16)).

^{31}P NMR (162 MHz, DMSO-*d*$_6$): δ (ppm) = 19.65.

Notes: The signal for the H-O(10) could not be detected.

IR: $\tilde{\nu}$/cm^{-1} = 3074 (vw), 2995 (w), 2947 (w), 2839 (vw), 1670 (m), 1606 (m), 1576 (s), 1541 (s), 1491 (w), 1471 (m), 1448 (s), 1427 (m), 1417 (m), 1373 (s), 1336 (m), 1308 (m), 1279 (m), 1242 (m), 1213 (s), 1205 (s), 1192 (s), 1159 (s), 1134 (vs), 1090 (m), 1059 (s), 1034 (s), 1012 (s), 968 (s), 930 (s), 916 (s), 885 (s), 818 (s), 808 (s), 752 (s), 735 (s), 710 (m), 688 (s), 665 (s), 648 (m), 625 (s), 592 (s), 557 (m), 546 (m), 509 (vs), 488 (vs), 480 (vs), 467 (vs), 449 (vs), 424 (vs), 409 (vs).

M.p.: 235 °C (decomposition).

HR-MS (APPI) for $[C_{18}H_{18}O_6P]^+$, $[M+H]^+$, calcd. 361.0836, found 361.0843.

(*Z*)-1-ethoxy-2-(2,6-dimethoxybenzylidene)-2-hydrophosphindol-3-one 1-oxide (4)

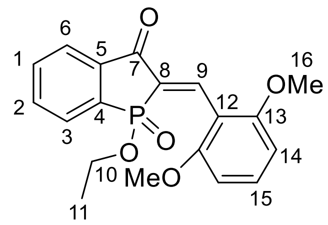

(*Z*)-1-ethoxy-2-(2,6-dimethoxybenzylidene)-2-hydrophosphindol-3-one 1-oxide **4** was synthesized according to **TP1** from hydrophosphindol **20** (0.750 mmol) and 2,6-trimethoxybenzaldehyde (0.750 mmol). FCC (SiO_2, CH_2Cl_2/MeOH = 99/1 → 98/2 → 97/3) afforded **4** as yellow solid (158 mg, 0.441 mmol, 59%).

^{1}H NMR (400 MHz, $CDCl_3$): δ (ppm) = 8.77 (d, *J* = 37.0 Hz, 1H, H-C(9)), 8.08 (dd, *J* = 7.5, 3.3 Hz, 1H, H-C(6)), 7.91 (ddt, *J* = 10.6, 7.4, 1.0 Hz, 1H, H-C(3)), 7.76 (tdd, *J* = 7.4, 3.3, 1.3 Hz, 1H, H-C(2)), 7.70 (tt, *J* = 7.4, 1.2 Hz, 1H, H-C(1)), 7.42 (t, *J* = 8.4 Hz, 1H, H-C(15)), 6.58 (d, *J* = 8.4 Hz, 2H, H-C(14)), 3.97 (s, 6H, H-C(16)), 3.88 – 3.76 (m, 1H, H-C(10)), 3.71 – 3.60 (m, 1H, H-C(10)), 1.15 (t, *J* = 7.0 Hz, 3H, H-C(11)).

^{13}C NMR (101 MHz, $CDCl_3$): δ (ppm) = 187.7 (d, *J* = 44.6 Hz, C(7)), 160.9 (C(13)), 143.7 (d, *J* = 2.9 Hz, C(9)), 139.9 (d, *J* = 11.1 Hz, C(5)), 138.1 (d, *J* = 124.4 Hz, C(4)), 135.1 (C(15)), 135.0 (d, *J* = 11.7 Hz, C(2)), 133.0 (d, *J* = 2.2 Hz, C(1)), 127.6 (d, *J* = 5.0 Hz, C(3)), 124.5 (d, *J* = 10.7 Hz, C(6)), 122.9 (d, *J* = 143.9 Hz, C(8)), 112.0 (d, *J* = 3.5 Hz, C(12)), 103.5 (C(14)), 62.5 (d, *J* = 6.3 Hz, C(10))), 55.9 (C(16)), 16.2 (d, *J* = 8.1 Hz, C(11)).

^{31}P NMR (162 MHz, $CDCl_3$): δ (ppm) = 31.03 (*E* isomer), 27.38.

Note: The sample contains 3% of the *E* isomer.

IR: $\tilde{\nu}$/cm^{-1} = 3059 (w), 3012 (w), 2997 (w), 2964 (w), 2931 (w), 2839 (w), 1685 (m), 1597 (s), 1581 (s), 1566 (m), 1560 (m), 1479 (m), 1469 (m), 1460 (m), 1448 (m), 1437 (m), 1425 (m), 1346 (m), 1321 (m), 1306 (w), 1255 (s), 1238 (s), 1230 (vs), 1201 (s), 1130 (m), 1111 (vs), 1074 (m), 1049 (s), 1030 (s), 1011 (s), 958 (s), 945 (s), 930 (s), 810 (m), 783 (s), 775 (s), 739 (vs), 719 (vs), 694 (s), 679 (m), 656 (m), 613 (m), 592 (s), 550 (vs), 530 (m), 511 (vs), 486 (s), 471 (s), 444 (m), 424 (m), 405 (m).

M.p.: 143 °C.

HR-MS (APPI) for $[C_{19}H_{19}O_5P]^+$, $[M+H]^+$, calcd. 358.0965, found 358.0961.

R*f* (SiO_2, CH_2Cl_2/MeOH = 98/2) = 0.24.

(*Z*)-1-ethoxy-2-(4-methoxy-2,6-dimethylbenzylidene)-2-hydrophosphindol-3-one 1-oxide (5)

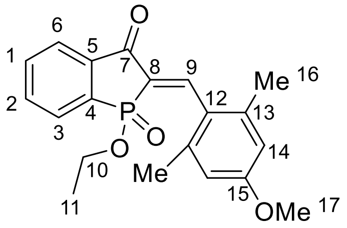

(*Z*)-1-ethoxy-2-(4-methoxy-2,6-dimethylbenzylidene)-2-hyd rophosphindol-3-one 1-oxide **5** was synthesized according to **TP1** from hydrophosphindol **20** (0.750 mmol) and 2-methoxy-4-6-dimethylbenzaldehyde **21** (0.500 mmol). FCC (SiO_2, CH_2Cl_2/MeOH = 99/1 → 98/2) and subsequent recrystallization from CH_2Cl_2 and *n*-heptane afforded **5** as yellow solid (109 mg, 0.306 mmol, 61%).

^{1}H NMR (500 MHz, $CDCl_3$): δ (ppm) = 8.44 – 8.35 (m, 1H, H-C(9)), 8.15 – 8.11 (m, 1H, H-C(6)), 7.89 – 7.83 (m, 1H, H-C(3)), 7.81 – 7.77 (m, 1H, H-C(2)), 7.77 – 7.73 (m, 1H, H-C(1)), 6.65 (s, 2H, H-C(14)), 3.92 – 3.81 (m, 1H, H-C(10)), 3.81 (s, 3H, H-C(17)), 3.59 (ddq, *J* = 10.0, 8.9, 7.1 Hz, 1H, H-C(10)), 2.30 (s, 6H, H-C(16)), 1.10 (t, *J* = 7.0 Hz, 3H, H-C(11)).

^{13}C NMR (126 MHz, $CDCl_3$): δ (ppm) = 184.9 (d, *J* = 43.3 Hz, C(7)), 159.9 (C(15)), 152.8 (d, *J* = 4.8 Hz, C(9)), 140.4 (d, *J* = 11.9 Hz, C(5)), 138.0 (d, *J* = 127.3 Hz, C(4)), 137.7 (d, *J* = 1.1 Hz, C(13)), 135.4 (d, *J* = 12.2 Hz, C(2)), 133.7 (d, *J* = 2.3 Hz, C(1)), 132.0 (d, *J* = 137.4 Hz, C(8)), 127.7 (d, *J* = 5.1 Hz, C(3)), 126.6 (d, *J* = 5.6 Hz, C(12)), 125.2 (d, *J* = 11.5 Hz, C(6)), 113.1 (C(14)), 62.4 (d, *J* = 6.4 Hz, C(10)), 55.3 (C(17)), 20.8 (C(16)), 16.6 (d, *J* = 6.3 Hz, C(11)).

^{31}P NMR (203 MHz, $CDCl_3$): δ (ppm) = 27.99.

IR: $\tilde{\nu}$/cm^{-1} = 3053 (w), 2970 (w), 2912 (w), 2837 (w), 1739 (w), 1695 (m), 1620 (s), 1591 (s), 1479 (m), 1460 (m), 1369 (w), 1308 (s), 1279 (m), 1219 (vs), 1186 (m), 1144 (s), 1128 (s), 1061 (s), 1020 (s), 995 (s), 962 (s), 941 (s), 920 (s), 906 (s), 899 (s), 874 (s), 841 (s), 796 (m), 775 (m), 742 (vs), 714 (m), 688 (vs), 667 (m), 642 (m), 592 (m), 553 (s), 532 (m), 523 (m), 515 (s), 490 (s), 471 (s), 449 (s), 428 (s), 418 (s), 409 (s).

M.p.: 133 °C.

HR-MS (APPI) for $[C_{20}H_{22}O_4P]^+$, $[M+H]^+$, calcd. 357.1250, found 357.1253

R*f* (SiO_2, CH_2Cl_2/MeOH = 98/2) = 0.21.

(*Z*)-1-ethoxy-2-(2-methoxy-4,6-dimethylbenzylidene)-2-hydrophosphindol-3-one 1-oxide (6)

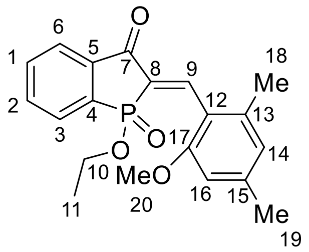

(*Z*)-1-ethoxy-2-(2-methoxy-4,6-dimethylbenzylidene)-2-hydrophosphindol-3-one 1-oxide **6** was synthesized according to **TP1** from hydrophosphindol **20** (0.750 mmol) and 2-methoxy-4-6-dimethylbenzaldehyde **22** (0.750 mmol). FCC (SiO_2, CH_2Cl_2/MeOH = 99/1 → 98/2) and subsequent recrystallization from CH_2Cl_2 and *n*-heptane afforded **6** as yellow solid (224 mg, 0.629 mmol, 84%).

^{1}H NMR (500 MHz, toluene-d_8): δ (ppm) = 8.64 – 8.55 (m, 1H, H-C(9)), 7.91 – 7.88 (m, 1H, H-C(6)), 7.59 – 7.54 (m, 1H, H-C(3)), 7.04 (tdd, *J* = 7.4, 3.2, 1.3 Hz, 1H, H-C(2)), 7.02 – 6.98 (m, 1H, H-C(1)), 6.40 (s, 1H, H-C(14)), 6.33 (s, 1H, H-C(16)), 3.68 – 3.61 (m, 1H, H-C(10)), 3.60 (s, 3H, H-C(20)), 3.48 – 3.38 (m, 1H, H-C(10)), 2.25 (s, 3H, H-C(18)), 2.07 (s, 3H, H-C(19)), 0.83 (t, *J* = 7.0 Hz, 3H, H-C(11)).

^{13}C NMR (126 MHz, toluene-d_8): δ (ppm) = 185.9 (d, *J* = 42.7 Hz, C(7)), 159.0 (C(17)), 147.1 (d, *J* = 4.0 Hz, C(9)), 142.2 (C(18)), 140.8 (C(13)), 140.5 (d, *J* = 11.1 Hz, C(5)), 139.2 (d, *J* = 124.9 Hz, C(4)), 134.5 (d, *J* = 11.7 Hz, C(2)), 132.6 (d, *J* = 2.3 Hz, C(1)), 128.3 (d, *J* = 137.9 Hz, C(8)), 127.6 (d, *J* = 5.1 Hz, C(3)), 124.6 (d, *J* = 11.0 Hz, C(6)), 124.0 (C(14)), 120.5 (d, *J* = 4.3 Hz, C(12)), 109.2 (C(16)), 61.7 (d, *J* = 6.3 Hz, C(10)), 55.1 (C(20)), 21.7 (C(19)), 20.2 (C(18)), 16.2 (d, *J* = 7.4 Hz, C(12)).

^{31}P NMR (203 MHz, toluene-d_8): δ (ppm) = 28.12 (*E* isomer), 25.06.

Notes: The sample contains 5% of the *E* isomer and the signal for H-C(1) is overlapping with the residual toluene signal, resulting in a larger integral.

IR: $\tilde{\nu}$/cm^{-1} = 3006 (w), 2983 (w), 2968 (w), 2933 (w), 2904 (w), 2837 (w), 1695 (m), 1662 (w), 1616 (s), 1591 (m), 1570 (m), 1508 (w), 1489 (w), 1454 (m), 1410 (w), 1392 (m), 1367 (w), 1321 (s), 1311 (m), 1281 (m), 1232 (s), 1221 (s), 1203 (s), 1153 (m), 1138 (s), 1093 (s), 1051 (s), 1014 (vs), 957 (vs), 924 (s), 895 (m), 831 (s), 796 (m), 773 (s), 750 (s), 741 (s), 725 (s), 687 (s), 669 (m), 646 (m), 629 (w), 607 (m), 586 (m), 559 (s), 548 (s), 534 (s), 517 (s), 498 (s), 478 (vs), 451 (m), 426 (w), 409 (m).

M.p.: 152 °C.

HR-MS (APPI) for $[C_{20}H_{22}O_4P]^+$, $[M+H]^+$, calcd. 357.1250, found 357.1255

R*f* (SiO_2, CH_2Cl_2/MeOH = 98/2) = 0.30.

(*Z*)-2-(4-(dimethylamino)-2,6-dimethylbenzylidene)-1-ethoxy-2-hydrophosphindol-3-one 1-oxide (7)

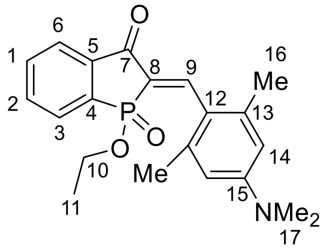

(*Z*)-2-(4-(dimethylamino)-2,6-dimethylbenzylidene)-1-ethoxy-2-hydrophosphindol-3-one 1-oxide **7** was synthesized according to **TP1** from hydrophosphindol **20** (0.750 mmol) and 2-methoxy-4-6-dimethylbenzaldehyde **23** (0.750 mmol). FCC (SiO_2, CH_2Cl_2/MeOH = 99/1 → 88/12) and subsequent purification by SFC (SiO_2, CO_2/MeOH= 93/7) afforded **7** (224 mg, 0.605 mmol, 81%) as a yellow solid and a mixture of *Z* and *E* isomers (91/9).

^{1}H NMR (400 MHz, toluene-d_8): δ (ppm) = 8.53 (d, *J* = 35.0 Hz, 1H, H-C(9)), 7.92 – 7.86 (m, 1H, H-C(6)), 7.52 – 7.45 (m, 1H, H-C(3)), 7.03 – 6.98 (m, 2H, H-C(1,2)), 6.31 (s, 2H, H-C(14)), 3.74 (ddq, *J* = 10.0, 7.9, 7.0 Hz, 1H, H-C(10)), 3.50 (ddq, *J* = 10.1, 8.9, 7.1 Hz, 1H, H-C(10)), 2.52 (s, 6H, H-C(17)), 2.36 (s, 6H, H-C(16)), 0.84 (t, *J* = 7.1 Hz, 3H, H-C(11)).

^{13}C NMR (101 MHz, toluene-d_8): δ (ppm) = 184.5 (d, *J* = 42.5 Hz, C(7)), 152.8 (d, *J* = 5.1 Hz, C(9)), 151.1 (C(15)), 140.9 (d, *J* = 11.9 Hz, C(5)), 139.1 (d, *J* = 126.8 Hz, C(4)), 137.9 (C(13)), 134.4 (d, *J* = 11.8 Hz, C(2)), 132.9 (d, *J* = 2.4 Hz, C(1)), 131.5 (d, *J* = 135.9 Hz, C(8)), 127.5 (d, *J* = 5.2 Hz, C(3)), 124.7 (C(6)), 123.4 (d, *J* = 5.3 Hz, C(12)), 111.7 (C(14)), 61.8 (d, *J* = 6.0 Hz, C(10)), 39.8 (C(17)), 21.3 (C(16)), 16.6 (d, *J* = 6.4 Hz, C(11)).

^{31}P NMR (126 MHz, toluene-d_8): δ (ppm) = 29.75 (*E* isomer), 26.79 (*Z* isomer).

Notes: The sample contains 10% of the *E* isomer and the signals for H-C(1,2) are overlapping with the residual toluene signal, resulting in a larger integral.

IR: $\tilde{\nu}$/cm^{-1} = 2981 (w), 2920 (w), 2854 (w), 2821 (w), 1739 (w), 1693 (m), 1591 (s), 1577 (m), 1560 (m), 1500 (m), 1448 (m), 1361 (s), 1309 (m), 1279 (m), 1227 (s), 1217 (vs), 1165 (w), 1132 (s), 1122 (m), 1063 (m), 1030 (s), 1014 (s), 960 (s), 930 (s), 910 (m), 881 (m), 858 (m), 841 (w), 823 (s), 796 (m), 777 (m), 742 (vs), 708 (m), 688 (s), 669 (m), 650 (w), 602 (m), 577 (m), 567 (m), 552 (s), 517 (m), 509 (m), 480 (vs), 457 (m), 444 (s), 430 (m), 418 (s), 407 (s).

M.p.: 150 °C.

HR-MS (APPI) for $[C_{21}H_{25}NO_3P]^+$, $[M+H]^+$, calcd. 370.1567, found 370.1574.

R*f* (SiO_2, CH_2Cl_2/MeOH = 98/2) = 0.26.

(*Z*)-1-ethoxy-2-((2,3,6,7-tetrahydro-1*H*,5*H*-pyrido[3,2,1-*ij*]quinolin-9-yl)methylene)-2-hydrophosphindol-3-one 1-oxide (8)

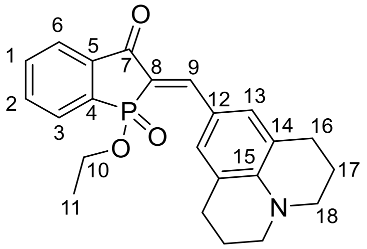

(*Z*)-1-ethoxy-2-((2,3,6,7-tetrahydro-1*H*,5*H*-pyrido[3,2,1-*ij*]quinolin-9-yl)methylene)-2-hydrophosphindol-3-one 1-oxide **8** was synthesized according to **TP1** from hydrophosphindol **20** (0.500 mmol) and 9-formyljulolidine **24** (0.500 mmol). Crystallization from CH_2Cl_2 and *n*-heptane afforded **8** (188 mg, 0.478 mmol, 96%) as red crystals.

^{1}H NMR (500 MHz, $CDCl_3$): δ (ppm) = 8.16 – 8.07 (m, 1H, H-C(9)), 8.07 – 8.04 (m, 1H, H-C(6)), 7.93 – 7.87 (m, 1H, H-C(3)), 7.74 – 7.67 (m, 2H, H-C(1,2)), 7.64 (s, 2H, H-C(13)), 3.90 (dqd, *J* = 8.7, 7.0, 5.3 Hz, 2H, H-C(10)), 3.36 – 3.32 (m, 4H, H-C(18)), 2.86 – 2.74 (m, 4H, H-C(16)), 2.01 – 1.94 (m, 4H, H-C(17)), 1.20 (t, *J* = 7.0 Hz, 3H, H-C(11)).

^{13}C NMR (126 MHz, $CDCl_3$): δ (ppm) = 185.8 (d, *J* = 42.3 Hz, C(7)), 153.6 (d, *J* = 5.5 Hz, C(9)), 148.0 (C(15)), 141.6 (d, *J* = 13.3 Hz, C(5)), 135.8 (d, *J* = 124.2 Hz, C(4)), 134.1 (d, *J* = 11.9 Hz, C(2)), 133.8 (C(13)), 133.2 (d, *J* = 2.4 Hz, C(1)), 127.3 (d, *J* = 5.5 Hz, C(3)), 124.3 (d, *J* = 11.8 Hz,C(6)), 121.2 (C(14)), 120.4 (d, *J* = 4.2 Hz, C(12)), 112.2 (d, *J* = 142.7 Hz, C(8)), 62.5 (d, *J* = 6.3 Hz, C(10)), 50.4 (C(18)), 27.7 (C(16)), 21.3 (C(17)), 16.5 (d, *J* = 6.6 Hz, C(11)).

^{31}P NMR (203 MHz, $CDCl_3$): δ (ppm) = 36.08.

IR: $\tilde{\nu}$/cm^{-1} = 2972 (w), 2933 (m), 2843 (w), 1655 (m), 1612 (m), 1587 (m), 1576 (m), 1547 (m), 1508 (s), 1502 (s), 1458 (m), 1450 (m), 1435 (s), 1415 (s), 1365 (m), 1354 (m), 1336 (m), 1302 (s), 1257 (s), 1215 (s), 1203 (s), 1169 (s), 1161 (s), 1134 (s), 1084 (m), 1072 (m), 1059 (s), 1028 (vs), 993 (s), 955 (vs), 928 (m), 901 (m), 881 (m), 868 (m), 769 (m), 737 (s), 712 (m), 688 (s), 625 (m), 590 (w), 548 (m), 517 (m), 507 (s), 494 (m), 440 (m).

M.p.: 192 °C.

HR-MS (APPI) for $[C_{23}H_{25}NO_3P]^+$, $[M+H]^+$, calcd. 394.1567, found 394.1576.

(*Z*) -2-(4-(dimethylamino)benzylidene)-1-ethoxy-2-hydrophosphindol-3-one 1-oxide (9)

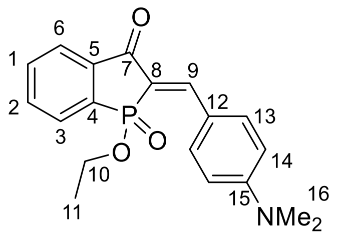

(*Z*)-2-(4-(dimethylamino)benzylidene)-1-ethoxy-2-hydrophosphindol-3-one 1-oxide **9** was synthesized according to **TP1** from hydrophosphindol **20** (0.500 mmol) and 4-dimethylaminobenzaldehyde (0.500 mmol). FCC (SiO_2, CH_2Cl_2/MeOH = 99/1 → 98/2→ 97/3) afforded **9** (117 mg, 0.343 mmol, 69%) as a red solid.

^{1}H NMR (500 MHz, $CDCl_3$): δ (ppm) = 8.24 (d, J = 34.7 Hz, 1H, H-C(9)), 8.11 – 8.07 (m, 3H, H-C(6, 13)), 7.98 – 7.93 (m, 1H, H-C(3)), 7.80 – 7.73 (m, 1H, H-C(2)), 7.76 – 7.69 (m, 1H, H-C(1)), 6.83 – 6.79 (m, 2H, H-C(14)), 3.90 (dqd, J = 8.6, 7.0, 5.4 Hz, 2H, H-C(10)), 3.13 (s, 6H, H-C(16)), 1.19 (t, J = 7.1 Hz, 3H, H-C(11)).

^{13}C NMR (126 MHz, $CDCl_3$): δ (ppm) = 185.8 (d, J = 42.1 Hz, C(7)), 153.5 (d, J = 5.1 Hz, C(9)), 153.4 (C(15)), 141.4 (d, J = 12.9 Hz, C(13)), 135.8 (d, J = 124.6 Hz, C(14)), 134.5 (d, J = 12.1 Hz, C(2)), 133.4 (d, J = 2.4 Hz, C(1)), 127.5 (d, J = 5.5 Hz, C(3)), 124.6 (d, J = 11.7 Hz, C(6)), 121.8 (C(12)), 114.9 (d, J = 140.2 Hz, C(8)), 112.3 (C(14)), 62.7 (d, J = 6.2 Hz, C(10)), 40.5 (C(16)), 16.4 (d, J = 6.9 Hz, C(11)).

^{31}P NMR (203 MHz, $CDCl_3$): δ (ppm) = 34.80.

IR: $\tilde{\nu}$/cm^{-1} = 2983 (m), 2897 (m), 1664 (s), 1606 (m), 1547 (s), 1508 (s), 1491 (s), 1466 (m), 1458 (m), 1448 (m), 1437 (s), 1379 (m), 1352 (m), 1319 (m), 1215 (s), 1190 (s), 1171 (s), 1130 (m), 1059 (m), 1016 (vs), 974 (m), 947 (s), 928 (m), 825 (s), 769 (m), 735 (s), 710 (m), 688 (s), 557 (s), 536 (s).

M.p.: 182 °C.

HR-MS (APPI) for $[C_{19}H_{21}NO_3P]^+$, $[M+H]^+$, calcd. 342.1254, found 342.1257.

R*f* (SiO_2, CH_2Cl_2/MeOH = 98/2) = 0.22.

(*Z*)-1-ethoxy-2-(4-methoxybenzylidene)-2-hydrophosphindol-3-one 1-oxide (10)

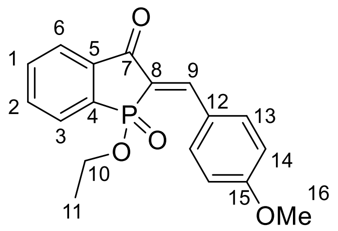

(*Z*)-1-ethoxy-2-(4-methoxybenzylidene)-2-hydrophosphindol-3-one 1-oxide **10** was synthesized according to **TP1** from hydrophosphindol **20** (0.309 mmol) and 4-methoxybenzaldehyde (0.309 mmol). FCC (SiO_2, CH_2Cl_2/MeOH = 99/1 → 98/2→ 97/3) and subsequent crystallization from CH_2Cl_2/*n*-heptane afforded **10** (60 mg, 0.182 mmol, 59%) as a yellow solid.

^{1}H NMR (500 MHz, $CDCl_3$): δ (ppm) = 8.28 (d, J = 34.3 Hz, 1H, H-C(9)), 8.18 – 8.14 (m, 2H, H-C(13)), 8.13 – 8.10 (m, 1H, H-C(6)), 7.98 (ddt, J = 11.2, 7.5, 1.0 Hz, 1H, H-C(3)), 7.80 (tdd, J = 7.3, 3.5, 1.4 Hz, 1H, H-C(2)), 7.76 (tt, J = 7.3, 1.3 Hz, 1H, H-C(1)), 7.06 – 7.01 (m, 2H, H-C(14)), 3.99 – 3.91 (m, 2H, H-C(10)), 3.90 (s, 3H, H-C(16)), 1.19 (t, J = 7.1 Hz, 3H, H-C(11)).

^{13}C NMR (126 MHz, $CDCl_3$): δ (ppm) = 185.6 (d, J = 41.8 Hz, C(7)), 163.8 (C(15)), 153.0 (d, J = 4.6 Hz, C(9)), 141.0 (d, J = 12.4 Hz, C(5)), 136.1 (d, J = 125.9 Hz, C(4)), 135.3 (d, J = 0.7 Hz, C(13)), 135.1 (d, J = 11.9 Hz, C(2)), 133.7 (d, J = 2.3 Hz, C(1)), 127.7 (d, J = 5.5 Hz, C(3)), 126.3 (d, J = 5.1 Hz, C(12)), 125.0 (d, J = 11.5 Hz, C(6)), 119.9 (d, J = 139.3 Hz, C(8)), 114.9 (C(14)), 63.0 (d, J = 6.4 Hz, C(10)), 55.7 (C(16)), 16.4 (d, J = 6.9 Hz, C(11)).

^{31}P NMR (203 MHz, $CDCl_3$): δ (ppm) = 32.59.

IR: $\tilde{\nu}$/cm^{-1} = 2989 (w), 2931 (w), 2897 (w), 2843 (w), 1680 (s), 1577 (s), 1545 (s), 1508 (s), 1477 (m), 1466 (m), 1458 (m), 1450 (m), 1429 (s), 1392 (m), 1363 (m), 1311 (m), 1269 (s), 1236 (s), 1215 (vs), 1203 (s), 1180 (vs), 1132 (s), 1074 (s), 1053 (s), 1018 (vs), 957 (vs), 930 (s), 881 (s), 835 (vs), 783 (s), 760 (s), 739 (vs), 725 (vs), 687 (vs), 660 (s), 621 (s), 550 (vs), 538 (vs), 499 (vs), 488 (vs), 445 (s), 432 (s), 424 (s), 417 (m), 409 (m).

M.p.: 134 °C.

HR-MS (APPI) for $[C_{18}H_{18}O_4P]^+$, $[M+H]^+$, calcd. 329.0937, found 329.0937.

R*f* (SiO_2, CH_2Cl_2/MeOH = 98/2) = 0.28.

(*Z*)-2-benzylidene-1-ethoxy-2-hydrophosphindol-3-one 1-oxide (11)

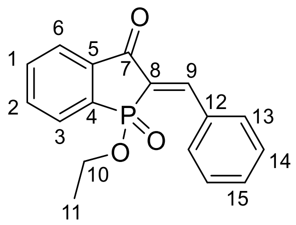

(*Z*)-2-benzylidene-1-ethoxy-2-hydrophosphindol-3-one 1-oxide **11** was synthesized according to **TP1** from hydrophosphindol **20** (0.357 mmol) and benzaldehyde (0.357 mmol). FCC (SiO_2, CH_2Cl_2/MeOH = 99/1 → 98/2→ 95/5) and subsequent purification *via* SFC (SiO_2, CO_2/MeOH =85/15, 120 bar) afforded **11** (30 mg, 0.101 mmol, 28%) as a colorless solid.

^{1}H NMR (400 MHz, $CDCl_3$): δ (ppm) = 8.34 (d, *J* = 34.9 Hz, 1H, H-C(9)), 8.20 – 8.16 (m, 2H, H-C(13)), 8.14 (ddt, *J* = 7.4, 3.6, 0.9 Hz, 1H, H-C(6)), 7.99 (ddt, *J* = 11.2, 7.5, 1.2 Hz, 1H, H-C(3)), 7.83 (tdd, *J* = 7.2, 3.4, 1.3 Hz, 1H, H-C(2)), 7.78 (tt, *J* = 7.4, 1.1 Hz, 1H, H-C(1)), 7.56 – 7.52 (m, 3H, H-C(14, 15)), 4.07 – 3.92 (m, 2H, H-C(10)), 1.18 (t, *J* = 7.0 Hz, 3H, H-C(11)).

^{13}C NMR (101 MHz, $CDCl_3$): δ (ppm) = 185.5 (d, *J* = 41.5 Hz, C(7)), 153.2 (d, *J* = 4.1 Hz, C(9)), 140.7 (d, *J* = 12.0 Hz, C(5)), 136.5 (d, *J* = 127.0 Hz, C(4)), 135.4 (d, *J* = 12.2 Hz, C(2)), 133.9 (d, *J* = 2.3 Hz, C(1)), 133.5 (d, *J* = 5.1 Hz, C(12)), 132.9 (C(15)), 132.6 (d, *J* = 0.8 Hz, C(13)), 129.4 (C(14)), 127.8 (d, *J* = 5.5 Hz, C(3)), 125.2 (d, *J* = 11.3 Hz, C(6)), 123.6 (d, *J* = 137.4 Hz, C(8)), 63.1 (d, *J* = 6.5 Hz, C(10)), 16.4 (d, *J* = 6.6 Hz, C(11)).

^{31}P NMR (162 MHz, $CDCl_3$): δ (ppm) = 31.19.

IR: $\tilde{\nu}$/cm^{-1} = 3062 (w), 2983 (w), 2922 (w), 2852 (w), 1691 (s), 1587 (vs), 1574 (s), 1564 (s), 1496 (w), 1450 (m), 1388 (w), 1361 (m), 1319 (m), 1277 (m), 1221 (s), 1203 (s), 1194 (s), 1169 (m), 1134 (s), 1105 (w), 1074 (m), 1047 (s), 1009 (vs), 960 (vs), 928 (s), 881 (m), 796 (m), 781 (m), 762 (vs), 735 (vs), 694 (m), 681 (vs), 661 (s), 615 (m), 565 (m), 553 (s), 534 (s), 503 (vs), 490 (vs), 449 (m), 424 (w).

M.p.: 119 °C.

HR-MS (APPI) for $[C_{17}H_{16}O_3P]^+$, $[M+H]^+$, calcd. 299.0832, found 299.0839.

R*f* (SiO_2, CH_2Cl_2/MeOH = 98/2) = 0.23.

(*Z*)-2-benzylidene-1-hydroxy-2-hydrophosphindol-3-one 1-oxide (11-OH)

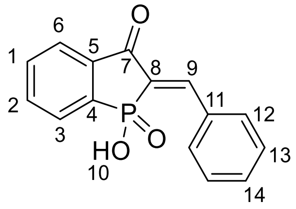

Under argon atmosphere, HPI **11** (22.0 mg, 0.074 mmol, 1.00 equiv.) was dissolved in anhydrous CH_2Cl_2 (0.5 mL). Then trimethylbromosilane (0.0179mL, 0.147 mmol, 2.00 equiv.) was added and the solution was stirred at 23 °C for 5 h. The solvent was removed *in vacuo* and the solid was taken up in methanol (5 mL) and water (3 mL) was added. Parts of the solvent were removed under reduced pressure until a solid precipitated. The precipitate was filtered off and washed with water. Drying under high vacuum afforded **11**-OH as off-white solid (7.0 mg, 0.026 mmol, 35%).

^{1}H NMR (500 MHz, DMSO-d_6): δ (ppm) = 8.28 – 8.22 (m, 2H, H-C(12)), 8.13 (d, J = 33.0 Hz, 1H, H-C(9)), 8.04 (dd, J = 7.7, 3.3 Hz, 1H, H-C(6)), 7.99 – 7.94 (m, 1H, H-C(3)), 7.94 – 7.91 (m, 1H, H-C(2)), 7.87 – 7.83 (m, 1H, H-C(1)), 7.62 – 7.54 (m, 2H, H-C(14, 13)).

^{13}C NMR (126 MHz, DMSO-d_6): δ (ppm) = 185.7 (d, J = 39.7 Hz, C(7)), 149.1 (d, J = 3.5 Hz, C(9)), 139.9 (d, J = 124.1 Hz, C(4)), 138.8 (d, J = 11.9 Hz, C(5)), 135.7 (d, J = 11.7 Hz, C(2)), 133.4 (d, J = 4.9 Hz, C(11)), 133.2 (d, J = 1.8 Hz, C(1)), 132.2 (C(14)), 132.2 (C(12)), 129.0 (C(13)), 127.5 (d, J = 133.7 Hz, C(8)), 127.2 (d, J = 5.9 Hz, C(3)), 124.3 (d, J = 10.9 Hz, C(6)).

^{31}P NMR (162 MHz, DMSO-d_6): δ (ppm) = 22.97.

Notes: The signal for the H-O(10) could not be detected.

IR: $\tilde{\nu}$/cm^{-1} = 2920 (w), 2850 (w), 1691 (m), 1587 (s), 1566 (m), 1491 (m), 1448 (m), 1323 (m), 1277 (m), 1227 (m), 1203 (m), 1163 (m), 1140 (s), 1105 (m), 1086 (m), 1055 (s), 1032 (s), 1014 (s), 989 (s), 953 (s), 922 (s), 897 (s), 796 (m), 756 (s), 750 (s), 737 (s), 677 (vs), 623 (m), 615 (s), 565 (m), 550 (s), 521 (vs), 503 (vs), 482 (vs), 444 (s), 426 (s), 420 (s), 413 (s), 405 (s).

M.p.: 254 °C (decomposition).

HR-MS (APPI) for $[C_{15}H_{12}O_3P]^+$, $[M+H]^+$, calcd. 271.0519, found 271.0520.

(*Z*)-4-((1-ethoxy-1-oxido-3-oxo-3-hydrophosphindol-2-ylidene)methyl)benzonitrile (12)

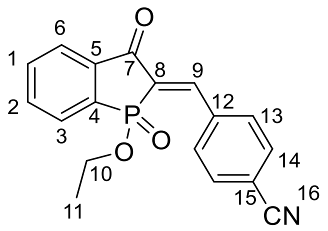

(*Z*)-4-((1-ethoxy-1-oxido-3-oxo-3-hydrophosphindol-2-ylidene)methyl)benzonitrile **12** was synthesized according to **TP1** from hydrophosphindol **20** (0.500 mmol) and 4-formylbenzonitrile (0.500 mmol). FCC (SiO_2, CH_2Cl_2/MeOH = 99/1 → 98/2→ 97/3) afforded **12** (100 mg, 0.309 mmol, 62%) as a colorless solid.

^{1}H NMR (400 MHz, $CDCl_3$): δ (ppm) = 8.30 – 8.26 (m, 2H, H-C(13)), 8.26 (d, J = 34.4 Hz, 1H, H-C(9)), 8.18 – 8.14 (m, 1H, H-C(6)), 8.03 – 7.97 (m, 1H, H-C(3)), 7.87 (tdd, J = 7.4, 3.6, 1.4 Hz, 1H, H-C(2)), 7.84 – 7.79 (m, 1H, H-C(1)), 7.83 – 7.79 (m, 2H, H-C(14)), 4.02 (dqd, J = 8.5, 7.0, 2.3 Hz, 2H, H-C(10)), 1.20 (t, J = 7.1 Hz, 3H, H-C(11)).

^{13}C NMR (101 MHz, $CDCl_3$): δ (ppm) = 184.8 (d, J = 40.6 Hz, C(7)), 149.7 (d, J = 3.3 Hz, C(9)), 140.6 (d, J = 11.6 Hz, C(5)), 137.6 (d, J = 5.4 Hz, C(12)), 136.3 (d, J = 127.9 Hz, C(4)), 135.9 (d, J = 12.3 Hz, C(2)), 134.2 (d, J = 2.5 Hz, C(1)), 132.9 (C(14)), 132.4 (d, J = 1.1 Hz, C(13)), 128.0 (d, J = 5.7 Hz, C(3)), 127.7 (d, J = 135.5 Hz, C(8)), 125.5 (d, J = 11.3 Hz, C(6)), 118.2 (C(16)), 115.4 (C(15)), 63.4 (d, J = 6.6 Hz, C(10)), 16.4 (d, J = 6.6 Hz, C(11)).

^{31}P NMR (162 MHz, $CDCl_3$): δ (ppm) = 29.79.

IR: $\tilde{\nu}$/cm^{-1} = 3062 (w), 3043 (w), 2985 (w), 2898 (w), 2229 (m), 1691 (s), 1601 (s), 1589 (s), 1554 (m), 1533 (w), 1506 (m), 1452 (m), 1415 (m), 1352 (m), 1321 (m), 1279 (m), 1223 (vs), 1205 (s), 1171 (m), 1130 (s), 1101 (m), 1074 (w), 1047 (s), 1020 (vs), 966 (vs), 949 (s), 928 (m), 839 (s), 822 (m), 789 (s), 742 (s), 725 (m), 708 (m), 688 (vs), 652 (w), 629 (m), 557 (s), 517 (w), 505 (s), 480 (s), 447 (s).

M.p.: 188 °C.

HR-MS (APPI) for $[C_{18}H_{15}NO_3P]^+$, $[M+H]^+$, calcd. 324.0784, found 324.0793.

R*f* (SiO_2, CH_2Cl_2/MeOH = 98/2) = 0.32.

(*Z*) -1-ethoxy-2-(4-(trifluoromethyl)benzylidene)-2-hydrophosphindol-3-one 1-oxide (13)

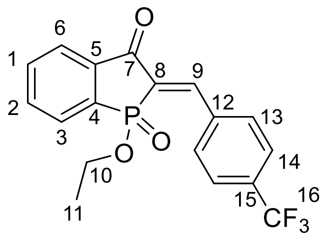

(*Z*)-1-ethoxy-2-(4-(trifluoromethyl)benzylidene)-2-hydrophosphindol-3-one 1-oxide **13** was synthesized according to **TP1** from hydrophosphindol **20** (0.500 mmol) and 4-trifluoromethylbenzaldehyde (0.500 mmol). FCC (SiO_2, CH_2Cl_2/MeOH = 100/0 → 99/1→ 98/2) afforded **13** (15 mg, 0.041 mmol, 40%) as a colorless solid.

^{1}H NMR (400 MHz, $CDCl_3$): *δ* (ppm) = 8.31 (d, *J* = 34.6 Hz, 1H, H-C(9)), 8.29 (d, *J* = 8.2 Hz, 2H, H-C(13)), 8.19 – 8.14 (m, 1H, H-C(6)), 8.04 – 7.97 (m, 1H, H-C(3)), 7.86 (tdd, *J* = 7.6, 3.6, 1.5 Hz, 1H, H-C(2)), 7.83 – 7.80 (m, 1H, H-C(1)), 7.78 (d, *J* = 8.1 Hz, 2H, H-C(14)), 4.03 (dqd, *J* = 8.7, 7.0, 4.3 Hz, 2H, H-C(10)), 1.20 (t, *J* = 7.0 Hz, 3H, H-C(11)).

^{13}C NMR (101 MHz, $CDCl_3$): *δ* (ppm) = 185.0 (d, *J* = 40.8 Hz, C(7)), 150.6 (d, *J* = 3.6 Hz, C(9)), 140.6 (d, *J* = 11.8 Hz, C(5)), 136.8 – 136.7 (m, C(12)), 136.4 (d, *J* = 128.8 Hz, C(4)), 135.7 (d, *J* =12.4 Hz, C(2)), 134.1 (d, *J* = 2.5 Hz, C(1)), 133.6 (q, *J* = 32.7 Hz, C(15)), 132.4 (q, *J* = 0.9 Hz, C(13)), 128.0 (d, *J* = 5.6 Hz, C(3)), 126.7 (d, *J* = 136.6 Hz, C(8)), 126.2 (q, *J* = 3.7 Hz, C(14)), 125.4 (d, *J* = 11.3 Hz, C(6)), 123.8 (q, *J* = 272.5 Hz, C(16)), 63.3 (d, *J* = 6.5 Hz, C(10)), 16.4 (d, *J* = 6.5 Hz, C(11)).

^{31}P NMR (162 MHz, $CDCl_3$): *δ* (ppm) = 30.09.

^{19}F NMR (377 MHz, $CDCl_3$): *δ* (ppm) = –63.12.

IR: $\tilde{\nu}$/cm^{-1} = 2991 (w), 2979 (w), 2854 (w), 1699 (s), 1604 (m), 1591 (m), 1568 (w), 1450 (w), 1414 (w), 1315 (s), 1277 (m), 1230 (m), 1219 (s), 1205 (m), 1194 (m), 1171 (s), 1161 (s), 1128 (vs), 1065 (s), 1041 (s), 1009 (vs), 958 (s), 945 (s), 926 (m), 885 (w), 868 (w), 831 (s), 816 (m), 793 (m), 771 (s), 744 (s), 731 (s), 704 (m), 688 (s), 627 (m), 607 (w), 592 (s), 553 (s), 530 (s), 507 (s), 492 (s), 463 (m), 444 (m), 424 (m), 407 (m).

M.p.: 117 °C.

HR-MS (APPI) for $[C_{18}H_{15}F_3O_3P]^+$, $[M+H]^+$, calcd. 367.0705, found 367.0712.

R*f* (SiO_2, CH_2Cl_2/MeOH = 98/2) = 0.28.

(*Z*)-1-ethoxy-2-(4-nitrobenzylidene)-2-hydrophosphindol-3-one 1-oxide (14)

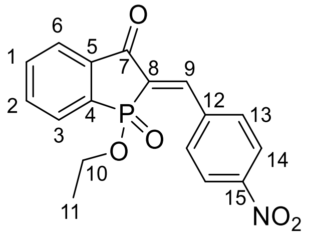

(*Z*)-1-ethoxy-2-(4-nitrobenzylidene)-2-hydrophosphindol-3-one 1-oxide **14** was synthesized according to **TP1** from hydrophosphindol **20** (0.500 mmol) and 4-nitrobenzaldehyde (0.500 mmol). FCC (SiO_2, CH_2Cl_2/MeOH = 99/1→ 98/2 → 97/3) and subsequent recrystallization from CH_2Cl_2/*n*-heptane afforded **14** (70 mg, 0.204 mmol, 41%) as a colorless solid.

^{1}H NMR (400 MHz, $CDCl_3$): *δ* (ppm) = 8.40 – 8.32 (m, 4H, H-C(13, 14)), 8.31 (d, *J* = 34.1 Hz, 1H, H-C(9)), 8.17 (ddt, *J* = 7.4, 3.6, 0.9 Hz, 1H, H-C(6)), 8.02 (ddt, *J* = 11.5, 7.5, 1.2 Hz, 1H, H-C(3)), 7.88 (tdd, *J* = 7.4, 3.6, 1.4 Hz, 1H, H-C(2)), 7.83 (tt, *J* = 7.4, 1.4 Hz, 1H, H-C(1)), 4.05 (dqd, *J* = 8.6, 7.0, 2.8 Hz, 2H, H-C(10)), 1.22 (t, *J* = 7.0 Hz, 3H, H-C(11)).

^{13}C NMR (101 MHz, $CDCl_3$): *δ* (ppm) = 184.7 (d, *J* = 40.7 Hz, C(7)), 149.5 (C(15)), 149.0 (d, *J* = 3.3 Hz, C(9)), 140.5 (d, *J* = 11.7 Hz, C(5)), 139.4 (d, *J* = 5.3 Hz, C(12)), 136.4 (d, *J* = 127.9 Hz, C(4)), 135.9 (d, *J* = 12.3 Hz, C(2)), 134.3 (d, *J* = 2.5 Hz, C(1)), 132.9 (d, *J* = 1.1 Hz, C(13)), 128.4 (d, *J* = 135.5 Hz, C(8)), 128.0 (d, *J* = 5.6 Hz, C(3)), 125.6 (d, *J* = 11.4 Hz, C(6)), 124.3 (C(14)), 63.4 (d, *J* = 6.7 Hz, C(10)), 16.5 (d, *J* = 6.4 Hz, C(11)).

^{31}P NMR (162 MHz, $CDCl_3$): *δ* (ppm) = 29.60.

IR: $\tilde{\nu}$/cm^{-1} = 3116 (w), 3055 (w), 2989 (w), 2931 (w), 2895 (w), 1691 (s), 1603 (m), 1587 (s), 1568 (m), 1510 (s), 1450 (w), 1414 (w), 1338 (s), 1323 (s), 1300 (m), 1281 (s), 1217 (vs), 1203 (s), 1157 (w), 1136 (s), 1109 (m), 1043 (s), 1022 (vs), 960 (s), 949 (s), 928 (s), 893 (w), 852 (s), 795 (m), 777 (w), 752 (s), 735 (vs), 679 (s), 619 (m), 553 (m), 528 (s), 490 (s), 455 (m), 409 (m).

M.p.: 198 °C.

HR-MS (APPI) for $[C_{17}H_{15}NO_5P]^+$, $[M+H]^+$, calcd. 344.0682, found 344.0691.

R*f* (SiO_2, CH_2Cl_2/MeOH = 98/2) = 0.28.

2-((1*H*-imidazol-2-yl)methylene)-1-ethoxy-2-hydrophosphindol-3-one 1-oxide (15)

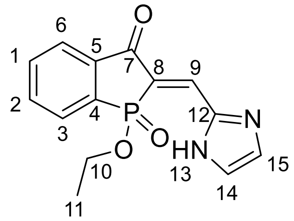

2-((1*H*-imidazol-2-yl)methylene)-1-ethoxy-2-hydrophosphindol-3-one 1-oxide **15** was synthesized according to **TP1** from hydrophosphindol **20** (0.750 mmol) and 1*H*-imidazole-2-carbaldehyde (0.750 mmol). FCC (SiO_2, CH_2Cl_2/MeOH = 99/1 → 98/2 → 97/3) and subsequent purification by HPLC (SiO_2, *n*-hexane/EA/*i*PrOH= 60/27/13) afforded **15** (78 mg, 0.271 mmol, 36%) as a yellow solid and a mixture of *Z* and *E* isomers.

^{1}H NMR (600 MHz, toluene-d_8): δ (ppm) = 13.64 (s, 1H, H-N(13)$_E$), 13.04 (s, 1H, H-N(13)$_Z$), 8.43 (d, *J* = 34.0 Hz, 1H, H-C(9)$_Z$), 7.92 (d, *J* = 18.5 Hz, 1H, H-C(9)$_E$), 7.81 – 7.80 (m, 1H, H-C(6)$_E$), 7.80 – 7.78 (m, 1H, H-C(13)$_Z$), 7.49 – 7.45 (m, 1H, H-C(3)$_E$), 7.44 – 7.40 (m, 1H, H-C(3)$_Z$), 7.27 (dd, *J* = 1.6, 0.8 Hz, 1H, H-C(14 or 15)$_E$), 7.23 (t, *J* = 1.1 Hz, 1H, H-C(14 or 15)$_Z$), 7.00 – 6.99 (m, 2H, H-C(1, 2)$_E$), 6.96 – 6.93 (m, 2H, H-C(1, 2)$_Z$), 6.45 – 6.43 (m, 1H, H-C(14 or 15)$_Z$), 6.36 – 6.34 (m, 1H, H-C(14 or 15)$_E$), 3.90 (ddq, *J* = 10.0, 8.2, 7.1 Hz, 1H, H-C(10)$_E$), 3.78 (ddq, *J* = 10.0, 8.2, 7.0 Hz, 1H, H-C(10)$_E$), 3.50 (ddq, *J* = 9.9, 8.0, 7.0 Hz, 1H, H-C(10)$_Z$), 3.31 (ddq, *J* = 9.9, 8.1, 7.0 Hz, 1H, H-C(10)$_Z$), 0.96 (t, *J* = 7.0 Hz, 3H, H-C(11)$_E$), 0.69 (t, *J* = 7.0 Hz, 3H, H-C(11)$_Z$).

^{13}C NMR (151 MHz, toluene-d_8): δ (ppm) = 187.5 (d, *J* = 40.1 Hz, C(7)$_E$), 183.6 (d, *J* = 38.8 Hz, C(7)$_Z$), 145.2 (d, *J* = 19.1 Hz, C(12)$_E$), 144.1 (d, *J* = 6.8 Hz, C(12)$_Z$), 142.1 (d, *J* = 12.8 Hz, C(5)$_Z$), 141.0 (d, *J* = 13.8 Hz, C(5)$_E$), 138.8 (d, *J* = 4.3 Hz, C(9)$_Z$), 138.2 (d, *J* = 9.0 Hz, C(9)$_E$), 135.5 (C(14 or 15)$_E$), 135.0 (d, *J* = 11.7 Hz, C(2)$_E$), 134.6 (C(14 or 15)$_Z$), 134.5 (d, *J* = 131.0 Hz, C(4)$_Z$), 134.4 (d, *J* = 12.8 Hz, C(2)$_Z$), 133.7 (d, *J* = 2.4 Hz, C(1)$_Z$), 133.0 (d, *J* = 2.4 Hz, C(1)$_E$), 127.4 (d, *J* = 5.7 Hz, C(3)$_Z$), 124.9 (C(6)$_Z$), 121.9 (d, *J* = 137.8 Hz, C(8)$_E$), 121.7 (C(14 or 15)$_Z$), 121.1 (d, *J* = 131.9 Hz, C(8)$_Z$), 120.8 (C(14 or 15)$_E$), 63.0 (d, *J* = 6.5 Hz, C(10)$_Z$), 62.0 (d, *J* = 6.2 Hz, C(10)$_E$), 16.6 (d, *J* = 6.4 Hz, C(11)$_E$), 16.0 (d, *J* = 6.9 Hz, C(11)$_Z$).

^{31}P NMR (162 MHz, toluene-d_8): δ (ppm) = 34.14 (*Z* isomer), 29.81 (*E* isomer).

Notes: The sample contains 15% of the *E* isomer. Due to tautomerization of the imidazole, signals for 14 and 15 can't be unambiguously assigned. Signals for C(3)$_E$, C(4)$_E$ and C(6)$_E$ could not be found due to low signal intensity and residual solvent overlap. For the *E* and *Z* conformation, the NOE experiments do not allow unambiguous assignment. Since the *Z* isomer proved to be the most stable isomer for all other HPI derivatives, it can be assumed that this is also the case for HPI **15**.

IR: $\tilde{\nu}$/cm^{-1} = 3197 (w), 3168 (w), 3122 (m), 3099 (m), 3049 (w), 3033 (w), 2979 (w), 2970 (w), 2918 (w), 1736 (w), 1684 (s), 1601 (s), 1587 (s), 1541 (m), 1435 (s), 1417 (m), 1388 (w), 1319 (m), 1304 (m), 1277 (m), 1246 (m), 1217 (s), 1207 (vs), 1169 (s), 1130 (s), 1095 (s), 1074 (m), 1059 (s), 1011 (vs), 945 (s), 926 (m), 906 (s), 795 (m), 779 (m), 768 (m), 756 (s), 746 (s), 723 (s), 714 (s), 688 (s), 636 (m), 552 (s), 525 (m), 509 (s), 484 (m), 418 (w).

M.p.: 182 °C.

HR-MS (APPI) for $[C_{14}H_{13}N_2O_3P]^+$, $[M+H]^+$, calcd. 288.0658, 288.0656.

R*f* (SiO_2, CH_2Cl_2/MeOH = 98/2) = 0.14.

1-ethoxy-2-((5-methyl-1*H*-pyrrol-2-yl)methylene)-2-hydrophosphindol-3-one 1-oxide (16)

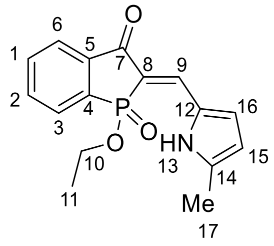

1-ethoxy-2-((5-methyl-1*H*-pyrrol-2-yl)methylene)-2-hydrophosphindol-3-one 1-oxide **16** was synthesized according to **TP1** from hydrophosphindol **20** (0.750 mmol) and 5-methyl-1*H*-pyrrole-2-carbaldehyde (0.750 mmol). FCC (SiO_2, CH_2Cl_2/MeOH = 99/1 → 98/2) and subsequent purification by HPLC (SiO_2, CH_2Cl_2/MeOH= 98.5/1.5) afforded **16** (220 mg, 0.730 mmol, 97%) as a red oil and a mixture of *Z* and *E* isomers.

^{1}H NMR (600 MHz, toluene-d_8): δ (ppm) = 13.42 (s, 1H, H-N(13)$_E$), 12.13 (s, 1H, H-N(13)$_{Zz}$), 8.19 – 8.03 (d, J = 32.9 Hz, 1H, H-C(9)$_Z$), 7.96 – 7.93 (m, 2H, H-C(6)$_{E\text{ and }Z}$), 7.60 – 7.56 (m, 1H, H-C(3)$_E$), 7.55 – 7.50 (m, 1H, H-C(3)$_Z$), 7.39 (d, J = 17.3 Hz, 1H, H-C(9)$_E$), 7.09 – 7.01 (m, 4H, H-C(1 and 2)$_{E\text{ and }Z}$), 6.49 (s, 1H, H-C(16)$_Z$), 6.46 (dd, J = 3.9, 2.1 Hz, 1H, H-C(16)$_E$), 5.85 – 5.83 (m, 1H, H-C(15)$_E$), 5.81 (t, J = 3.0 Hz, 1H, H-C(15)$_Z$), 4.01 – 3.90 (m, 2H, H-C(10)$_E$), 3.65 – 3.57 (m, 1H, H-C(10)$_Z$), 3.53 – 3.44 (m, 1H, H-C(10)$_Z$), 1.90 (s, 3H, H-C(17)$_Z$), 1.83 (s, 3H, H-C(17)$_E$), 1.06 (t, J = 7.0 Hz, 3H, H-C(11)$_E$), 0.78 (t, J = 7.0 Hz, 3H, H-C(11)$_Z$).

^{13}C NMR (151 MHz, toluene-d_8): δ (ppm) = 186.5 (d, J = 41.0 Hz, C(7)$_E$), 184.0 (d, J = 39.8 Hz, C(7)$_Z$), 142.9 (d, J = 13.6 Hz, C(5)$_Z$), 141.8 (d, J = 14.9 Hz, C(5)$_E$), 141.4 (C(14)$_Z$), 140.8 (C(14)$_E$), 138.3 (d, J = 6.3 Hz, C(9)$_Z$), 137.5 (C(9)$_E$), 136.7 (d, J = 121.6 Hz, C(4)$_Z$), 134.5 (d, J = 128.3 Hz, C(4)$_E$), 133.7 (d, J = 11.7 Hz, C(2)$_E$), 133.6 (d, J = 12.3 Hz, C(2)$_Z$), 133.2 (d, J = 2.4 Hz, C(1)$_Z$), 132.7 (d, J = 2.5 Hz, C(1)$_E$), 132.0 (d, J = 17.0 Hz, C(12)$_E$), 129.5 (d, J = 6.2 Hz, C(12)$_Z$), 127.2 (d, J = 4.9 Hz, C(3)$_E$), 127.1 (d, J = 5.5 Hz, C(3)$_Z$), 126.9 (C(16)$_E$), 126.7 (C(16)$_Z$), 124.4 (d, J = 12.3 Hz, C(6)$_Z$), 124.3 (d, J = 12.8 Hz, C(6)$_E$), 113.9 (C(15)$_E$), 112.6 (C(15)$_Z$), 111.3 (d, J = 145.5 Hz, C(8)$_E$), 111.2 (d, J = 138.7 Hz, C(8)$_Z$), 62.4 (d, J = 6.4 Hz, C(10)$_Z$), 61.7 (d, J = 6.0 Hz, C(10)$_E$), 16.6 (d, J = 6.5 Hz, C(11)$_E$), 16.1 (d, J = 6.9 Hz, C(11)$_E$), 13.4 (C(17)$_E$), 13.4 (C(17)$_Z$).

^{31}P NMR (202 MHz, toluene-d_8): δ (ppm) = 38.03 (*Z* isomer), 34.53 (*E* isomer).

Notes: The sample contains 33% of the *E* isomer. C(9)$_E$ overlaps with the toluene residual signal.

IR: $\tilde{\nu}$/cm^{-1} = 3446 (w), 3176 (w), 3060 (w), 2978 (w), 2898 (w), 1670 (m), 1651 (m), 1579 (s), 1554 (s), 1527 (vs), 1450 (m), 1404 (m), 1377 (w), 1340 (m), 1323 (m), 1292 (vs), 1275 (s), 1200 (s), 1184 (vs), 1161 (s), 1128 (s), 1082 (m), 1026 (s), 1005 (s), 982 (s), 945 (s), 910 (m), 889 (w), 783 (s), 746 (s), 729 (s), 717 (s), 704 (m), 692 (m), 679 (m), 656 (m), 642 (w), 609 (m), 553 (m), 505 (s), 469 (w).

HR-MS (APPI) for $[C_{16}H_{17}NO_3P]^+$, $[M+H]^+$, calcd. 302.0941, found 302.0946.

R*f* (SiO_2, CH_2Cl_2/MeOH = 98/2) = 0.36 (*Z* isomer), 0.26 (*E* isomer).

2-((1*H*-imidazol-4-yl)methylene)-1-ethoxy-2-hydrophosphindol-3-one 1-oxide (17)

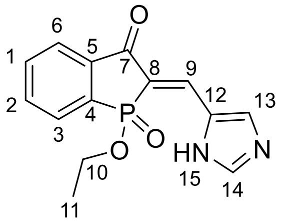

2-((1*H*-imidazol-4-yl)methylene)-1-ethoxy-2-hydrophosphindol-3-one 1-oxide **17** was synthesized according to **TP1** from hydrophosphindol **20** (0.750 mmol) and 1*H*-imidazole-4-carbaldehyde (0.750 mmol). Recrystallization from MeOH afforded **17** (87 mg, 0.302 mmol, 40%) as a yellow solid and a mixture of *Z* and *E* isomers.

^{1}H NMR (600 MHz, $CDCl_3$): δ (ppm) = 8.17 (d, J = 33.1 Hz, 1H, H-C(9)$_Z$), 8.13 – 8.11 (m, 1H, H-C(6)$_Z$), 8.11 – 8.09 (m, 1H, H-C(6)$_E$), 8.01 (s, 1H, H-C(14)$_E$), 8.00 – 7.96 (m, 1H, H-C(3)$_Z$), 7.94 (s, 1H, H-C(14)$_Z$), 7.93 – 7.91 (m, 1H, H-C(3)$_E$), 7.85 – 7.84 (m, 1H, H-C(2)$_E$), 7.83 (s, 1H, H-C(13)$_E$), 7.83 (s, 1H, H-C(13)$_Z$), 7.82 – 7.76 (m, 3H, H-C(1)$_{E \text{ and } Z}$, H-C(2)$_E$), 7.60 (s, J = 18.2 Hz, 1H, H-C(9)$_E$), 4.22 – 4.16 (m, 2H, H-C(10)$_E$), 4.02 (ddq, J = 9.9, 8.0, 7.0 Hz, 1H, H-C(10)$_Z$), 3.90 (ddq, J = 9.9, 8.2, 7.0 Hz, 1H, H-C(10)$_Z$), 1.35 (t, J = 7.1 Hz, 3H, H-C(11)$_E$), 1.20 (t, J = 7.1 Hz, 3H, H-C(11)$_E$).

^{13}C NMR (151 MHz, $CDCl_3$): δ (ppm) = 187.3 (d, J = 41.5 Hz, C(7)$_E$), 184.6 (d, J = 40.1 Hz, C(7)$_Z$), 142.5 (C(13)$_E$), 141.5 (d, J = 12.9 Hz, C(5)$_Z$), 140.8 (d, J = 13.2 Hz, C(5)$_E$), 140.8 (C(14)$_Z$), 140.2 (C(14)$_E$), 138.3 (C(13)$_Z$), 137.5 (C(9)$_Z$), 135.9 (d, J = 9.0 Hz, C(9)$_E$), 135.6 (d, J = 124.9 Hz, C(4)$_E$), 135.5 (d, J = 12.1 Hz, C(2)$_E$), 135.3 (d, J = 12.5 Hz, C(2)$_Z$), 134.4 (d, J = 129.6 Hz, C(4)$_E$), 134.3 (d, J = 2.4 Hz, C(1)$_Z$), 134.0 (d, J = 2.5 Hz, C(1)$_E$), 129.7 (C(12)$_Z$), 129.6 (d, J = 17.4 Hz, C(12)$_E$), 127.8 (d, J = 5.6 Hz, C(3)$_Z$), 127.7 (d, J = 4.9 Hz, C(3)$_E$), 125.3 (d, J = 11.9 Hz, C(6)$_E$), 125.2 (d, J = 12.0 Hz, C(6)$_Z$), 118.2 (d, J = 136.8 Hz, C(8)$_Z$), 117.9 (d, J = 141.1 Hz, C(8)$_E$), 63.6 (d, J = 6.5 Hz, C(10)$_Z$), 62.9 (d, J = 6.3 Hz, C(10)$_E$), 16.7 (d, J = 6.6 Hz, C(11)$_E$), 16.4 (d, J = 6.9 Hz, C(11)$_Z$).

^{31}P NMR (202 MHz, $CDCl_3$): δ (ppm) = 34.90 (*Z* isomer), 32.83 (*E* isomer).

Notes: The sample contains 22% of the *E* isomer.

IR: $\tilde{\nu}$/cm^{-1} = 3130 (w), 3109 (w), 3062 (w), 2983 (m), 2897 (m), 2827 (m), 2773 (w), 2744 (w), 2655 (w), 2569 (w), 1674 (s), 1581 (vs), 1568 (s), 1520 (s), 1431 (m), 1394 (w), 1344 (m), 1315 (m), 1273 (m), 1230 (m), 1217 (s), 1194 (vs), 1165 (m), 1140 (m), 1128 (s), 1101 (w), 1076 (m), 1063 (s), 1049 (s), 1028 (s), 982 (s), 962 (m), 949 (s), 926 (s), 912 (m), 843 (s), 827 (m), 798 (w), 773 (m), 741 (s), 721 (s), 704 (m), 690 (s), 679 (m), 621 (s), 546 (s), 530 (s), 505 (s), 488 (s), 471 (m).

M.p.: 222 °C.

HR-MS (APPI) for $[C_{14}H_{14}N_2O_3P]^+$, $[M+H]^+$, calcd. 289.0737, found 289.0743.

(*Z*)-1-hydroxy-2-(2,4,6-trimethylbenzylidene)-2-hydrophosphindol-3-one 1-oxide (17-OH)

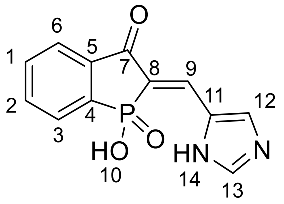

Under argon atmosphere, HPI **17** (22.0 mg, 0.065 mmol, 1.00 equiv.) was dissolved in anhydrous CH_2Cl_2 (1.0 mL) to obtain a 0.1 M solution. Then trimethylbromosilane (0.028 mL, 0.215 mmol, 2.00 equiv.) was added and the solution was stirred at 23 °C for 3 h. The solvent was removed *in vacuo* and the solid was taken up in methanol (5 mL) and water (3 mL) was added. Parts of the solvent were removed under reduced pressure until a solid precipitated. The precipitate was filtered off and washed with water. Drying under high vacuum afforded **17**-OH as off-white solid (15 mg, 0.058 mmol, 54%).

^{1}H NMR (600 MHz, DMSO-*d*$_6$): δ (ppm) = 8.51 (s, 1H, H-C(13)), 8.22 (s, 1H, H-C(12)), 7.94 (ddt, *J* = 7.7, 3.1, 1.0 Hz, 1H, H-C(6)), 7.88 – 7.84 (m, 2H, H-C(3, 2)), 7.81 (d, *J* = 30.1 Hz, 1H, H-C(9)), 7.76 – 7.72 (m, 1H, H-C(1)).

^{13}C NMR (151 MHz, DMSO-*d*$_6$): δ (ppm) = 186.6 (d, *J* = 36.2 Hz, C(7)), 142.1 (d, *J* = 124.5 Hz, C(4)), 138.9 (d, *J* = 11.8 Hz, C(5)), 137.6 (C(13)), 135.2 (d, *J* = 11.4 Hz, C(2)), 133.0 (C(9), 132.4 (C(11)), 132.1 (d, *J* = 1.9 Hz, C(1)), 128.7 (d, *J* = 132.2 Hz, C(8)), 126.7 (d, *J* = 5.7 Hz, C(3)), 126.5 (C(12)), 123.9 (d, *J* = 10.7 Hz, C(6)).

^{31}P NMR (162 MHz, DMSO-*d*$_6$): δ (ppm) = 20.01

Notes: The signal for the H-O(10) and H-N(14) could not be detected.

IR: $\tilde{\nu}$/cm^{-1} = 3222 (m), 3132 (m), 3124 (m), 3024 (m), 2563 (m), 1684 (s), 1616 (s), 1589 (m), 1525 (w), 1448 (m), 1361 (m), 1321 (m), 1279 (m), 1217 (s), 1149 (s), 1140 (s), 1084 (s), 1061 (s), 1030 (s), 893 (m), 864 (m), 793 (m), 748 (s), 710 (s), 696 (s), 673 (s), 656 (s), 619 (s), 546 (s), 534 (s), 513 (vs), 498 (s), 471 (s), 445 (s), 424 (m), 413 (s).

M.p.: 317 °C.

HR-MS (APPI) for $[C_{12}H_{10}N_2O_3P]^+$, $[M+H]^+$, calcd. 261.0424, found 261.0421.

Z-2-((1H-indol-3-yl)methylene)-1-ethoxy-2-hydrophosphindol-3-one 1-oxideoxide (18)

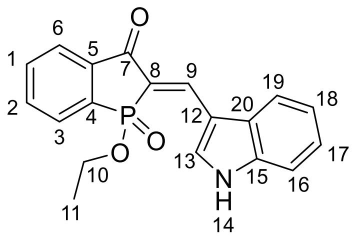

(2-((1H-indol-3-yl)methylene)-1-ethoxy-2-hydrophosphindol-3-one 1-oxide **18** was synthesized according to **TP1** from hydrophosphindol **20** (0.500 mmol) and 1*H*-indole-3-carbaldehyde (0.500 mmol). FCC (SiO_2, CH_2Cl_2/MeOH = 99/1 → 98/2) and subsequent crystallization from CH_2Cl_2/ *n*-heptane afforded **18** (133 mg, 0.394 mmol, 79%) as a yellow solid.

^{1}H NMR (500 MHz, $CDCl_3$): δ (ppm) = 11.73 (s, 1H, H-N(14)), 8.92 (d, J = 2.9 Hz, 1H, H-C(13)), 8.81 (d, J = 34.5 Hz, 1H, H-C(9)), 8.22 – 8.17 (m, 1H, H-C(6)), 8.06 – 8.01 (m, 1H, H-C(3)), 7.93 – 7.88 (m, 1H, H-C(19)), 7.87 – 7.79 (m, 2H, H-C(1, 2)), 7.52 – 7.47 (m, 1H, H-C(16)), 7.32 – 7.27 (m, 2H, H-C(18)), 3.87 – 3.70 (m, 2H, H-C(10)), 1.12 (t, J = 7.0 Hz, 3H, H-C(11)).

^{13}C NMR (126 MHz, $CDCl_3$): δ (ppm) = 185.1 (d, J = 42.3 Hz, C(7)), 144.3 (d, J = 6.0 Hz, C(9)), 142.1 (d, J = 13.5 Hz, C(5)), 137.0 (C(15)), 134.6 (d, J = 12.0 Hz, C(2)), 134.4 (d, J = 124.6 Hz, C(4)), 134.2 (C(13)), 134.0 (d, J = 2.8 Hz, C(1)), 128.3 (C(20)), 127.5 (d, J = 5.5 Hz, C(3)), 124.8 (d, J = 12.0 Hz, C(6)), 123.9 (C(17)), 122.8 (C(18)), 118.6 (C(19)), 113.0 (C(16)), 112.8 (d, J = 5.4 Hz, C(12)), 112.1 (d, J = 140.2 Hz, C(8)), 63.3 (d, J = 6.1 Hz, C(10)), 16.3 (d, J = 7.2 Hz, C(11)).

^{31}P NMR (203 MHz, $CDCl_3$): δ (ppm) = 37.17.

IR: $\tilde{\nu}$/cm^{-1} = 3273 (w), 3261 (w), 3120 (w), 3051 (w), 2981 (w), 1738 (w), 1668 (s), 1587 (m), 1576 (m), 1556 (vs), 1506 (s), 1491 (s), 1462 (m), 1435 (m), 1360 (s), 1336 (s), 1321 (s), 1300 (m), 1277 (m), 1246 (w), 1221 (s), 1207 (vs), 1167 (m), 1128 (s), 1119 (s), 1090 (s), 1070 (m), 1032 (s), 953 (s), 926 (s), 887 (w), 879 (w), 868 (w), 787 (m), 775 (m), 762 (w), 741 (s), 714 (s), 688 (vs), 671 (s), 609 (s), 584 (m), 553 (s), 540 (s), 519 (s), 501 (s), 482 (m), 467 (m), 426 (s).

M.p.: 238 °C.

HR-MS (APPI) for $[C_{19}H_{17}NO_3P]^+$, $[M+H]^+$, calcd. 338.0941, found 338.0950.

R*f* (SiO_2, CH_2Cl_2/MeOH = 98/2) = 0.22.

Supplementary Note 3: Water solubility

Water solubility was quantified for HPIs **3**, **11**, **15** and **17** and hydrolyzed derivatives **1**-OH, **2**-OH, **3**-OH, **11**-OH and **17**-OH **15** and **17**. Small solid samples were taken and water (purified with a Q-POD device from *Merck Millipore*) was added with a microliter pipette at 22 °C. After each addition step, the sample was sonicated for 2 min. If solids were still present, the previous steps were repeated until full dissolution was observed. For each derivative, this process was performed two times and the averaged water solubilities are given below:

Supplementary Table 1. Water solubility of different HPIs and hydrolyzed HPIs.

HPI	Water solubility [g/L]	Saturation concentration [mM]
**1**-OH	0.02	0.05
**2**-OH	0.02	0.06
**3**	0.02	0.06
**3**-OH	0.11	0.30
**11**	0.09	0.31
**11**-OH	0.16	0.58
**15**	0.25	0.85
**17**	0.15	0.54
**17**-OH	0.16	0.65

Supplementary Note 4: Thermal isomerization of HPIs

The thermal isomerizations of the metastable *E* isomers of the HPIs were investigated by charging NMR tubes with the respective *Z* isomers (3.0 mg to 7.0 mg), as obtained from the synthesis, and adding toluene-d_8 or *p*-xylene-d_{10} (0.7 mL, previously filtered over basic Al_2O_3 and anhydrous Na_2SO_4). An *E* isomer enriched solution was obtained by irradiation with light of different wavelengths (see Supplementary Note 8). The NMR tubes were then heated to the corresponding temperatures (65 °C to 130 °C) for isomerization reactions to occur and the kinetics were followed by ^{1}H NMR measurements in defined time intervals. The conversion and isomer concentration for each interval was determined by integration of well separated indicative proton signals.

The thermal isomerizations of the metastable *E* isomers for the hydrolyzed HPI derivatives HPI **1-OH**, **2-OH, 3-OH, 11-OH** and **17-OH** were investigated by preparing saturated solutions in D_2O which were filtered before measurement using a syringe filter. The samples were then measured as described above.

For all derivatives analyzed in this work, the thermal double bond isomerization between a metastable *E* and a stable *Z* isomer proceed towards a thermal equilibrium with a stable isomer composition:

$$\mathrm{E} \underset{k_Z}{\overset{k_E}{\rightleftharpoons}} \mathrm{Z} \tag{1}$$

with k_E and k_Z being the rate constants of the *E* to *Z* and *Z* to *E* isomerization reactions, respectively.

The overall rate constant *k* is given by:

$$k = k_E + k_Z \tag{2}$$

The thermal double bond isomerization, a unimolecular first order reaction proceeding towards an equilibrium, can be described by (3):

$$\ln\left(\frac{x_{\mathrm{eq}}}{x_{\mathrm{eq}} - x}\right) = \ln\left(\frac{[\mathrm{E_0}] - [\mathrm{E_{eq}}]}{[\mathrm{E_t}] - [\mathrm{E_{eq}}]}\right) = (k_E + k_Z)t = kt \tag{3}$$

with $x_{\mathrm{eq}} = [\mathrm{E_0}] - [\mathrm{E_{eq}}]$ and $x = [\mathrm{E_0}] - [\mathrm{E_t}]$

with $[E_0]$ being the initial concentration of *E* isomer at the time *t* =0, $[E_{eq}]$ being the concentration of *E* isomer at equilibrium, $[E_t]$ being the concentration of *E* isomer at specific time *t*, and *k* representing the overall rate constant.

The overall rate constant *k* can be directly obtained by plotting ln(x_{eq}/x_{eq}-x) versus time *t*. The slope of the linear fit is equivalent to the overall rate constant *k*.

$$K = \frac{k_E}{k_Z} = \frac{[\mathrm{Z_{eq}}]}{[\mathrm{E_{eq}}]} \quad \rightarrow \quad k_Z = k_E \frac{[\mathrm{E_{eq}}]}{[\mathrm{Z_{eq}}]} \tag{4}$$

Using the law of mass action (4) and (2), the rate constant k_E can than be calculated from the overall rate constant *k*:

$$k_E = \frac{k}{1 + \frac{[\mathrm{E_{eq}}]}{[\mathrm{Z_{eq}}]}} \tag{5}$$

The *Eyring* equation describes the relation between the rate constant k_E and the corresponding *Gibbs* energy of activation $\Delta G^{\ddagger}$:

$$k_E = \frac{k_B T}{h} e^{-\frac{\Delta G^{\ddagger}}{RT}} \quad (6)$$

with

k_B = Boltzmann constant ($1.381 \cdot 10^{-23}$ JK^{-1})
T = temperature in K
h = Planck constant ($6.626 \cdot 10^{-34}$ Js)
R = Gas constant (8.314 JK^{-1}mol^{-1})

The *Gibbs* energy of activation $\Delta G^{\ddagger}$ for the thermal *E* to *Z* isomerizations can then be calculated using the rate constants k_E and rearranged (6):

$$\Delta G_E \rightarrow Z^{\ddagger} = -RT \ln\left(\frac{k_E h}{k_B T}\right) \quad (7)$$

The half-life $\tau_{1/2}$ of a first order reaction is given by:

$$\tau_{1/2} = \frac{\ln 2}{k_E} \quad (8)$$

For a thermal isomerization reaching equilibrium, it is important to note, that k is the overall rate constant, which takes back isomerization into account and not the specific rate constant k_E, which was used to calculate $\Delta G^{\ddagger}$ and half life values.

The overall rate constant was linearly extrapolated to 25 °C using (9) and (7) under the assumption that the *Gibbs* energy of activation $\Delta G^{\ddagger}$ is not temperature dependent.

$$k_{25\,°C} = \frac{k_B T_{25\,°C}}{h} e^{-\frac{\Delta G^{\ddagger}}{RT_{25\,°C}}} \quad (9)$$

The determined rate constants k, the calculated *Gibbs* energies of activation $\Delta G^{\ddagger}$ and half-lives $\tau_{1/2}$ for all measured thermal isomerization reactions are summarized in Supplementary Table 2.

1.1.1 Thermal isomerization of HPI 1

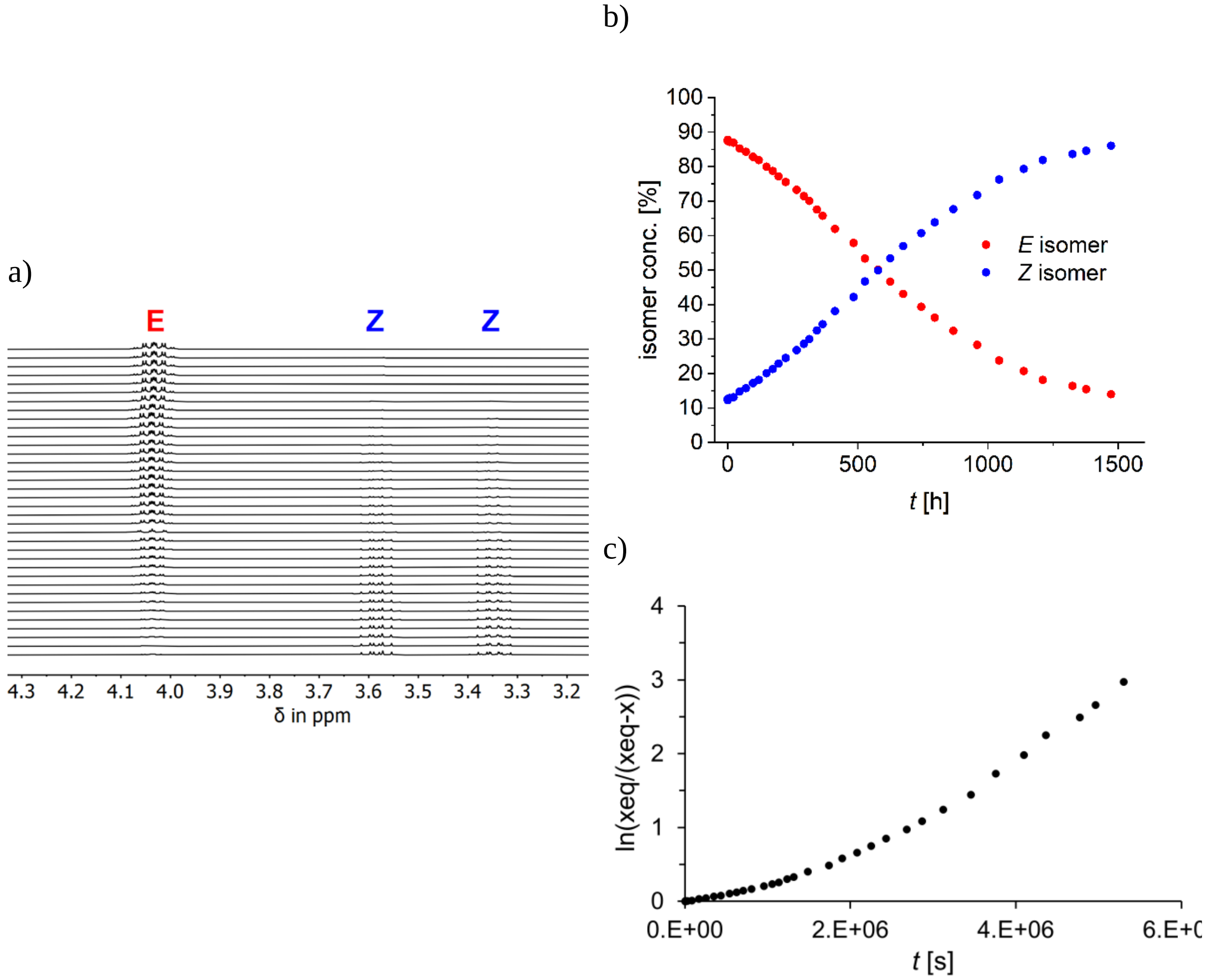

Supplementary Figure 5. Thermal *E* to *Z* isomerization of HPI **1** in *p*-xylene-d_{10} in the dark starting from *E* enriched solution, which was obtained by first irradiating the solution with 395 nm light. a) Thermal *E* to *Z* isomerization of HPI **1** in *p*-xylene-d_{10} at 130 °C in the dark followed by ^{1}H NMR spectroscopy (400 MHz, 23 °C) in regular time intervals. b) Isomer conversion over time. c) First order kinetic analysis of the thermal isomerization of *E* to *Z* proceeding towards a thermal equilibrium. The sigmoidal shape of the curve indicates a more complex order for the thermal isomerization and is most probably due to aggregation. For this reason, no rate constant and corresponding *Gibbs* energy of activation $\Delta G^{\ddagger}$ could be determined. The half-life at 130 °C was determined by fitting a polynomial function to the data in b) and the value is given in Supplementary Table 2.

Note: For a rough estimation of the *Gibbs* energy of activation $\Delta G^{\ddagger}$, it was calculated for three data points (548 h, 714 h and 1110 h) and in all cases exceeded 35.5 kcal/mol.

1.1.2 Thermal isomerization of HPI 1-OH

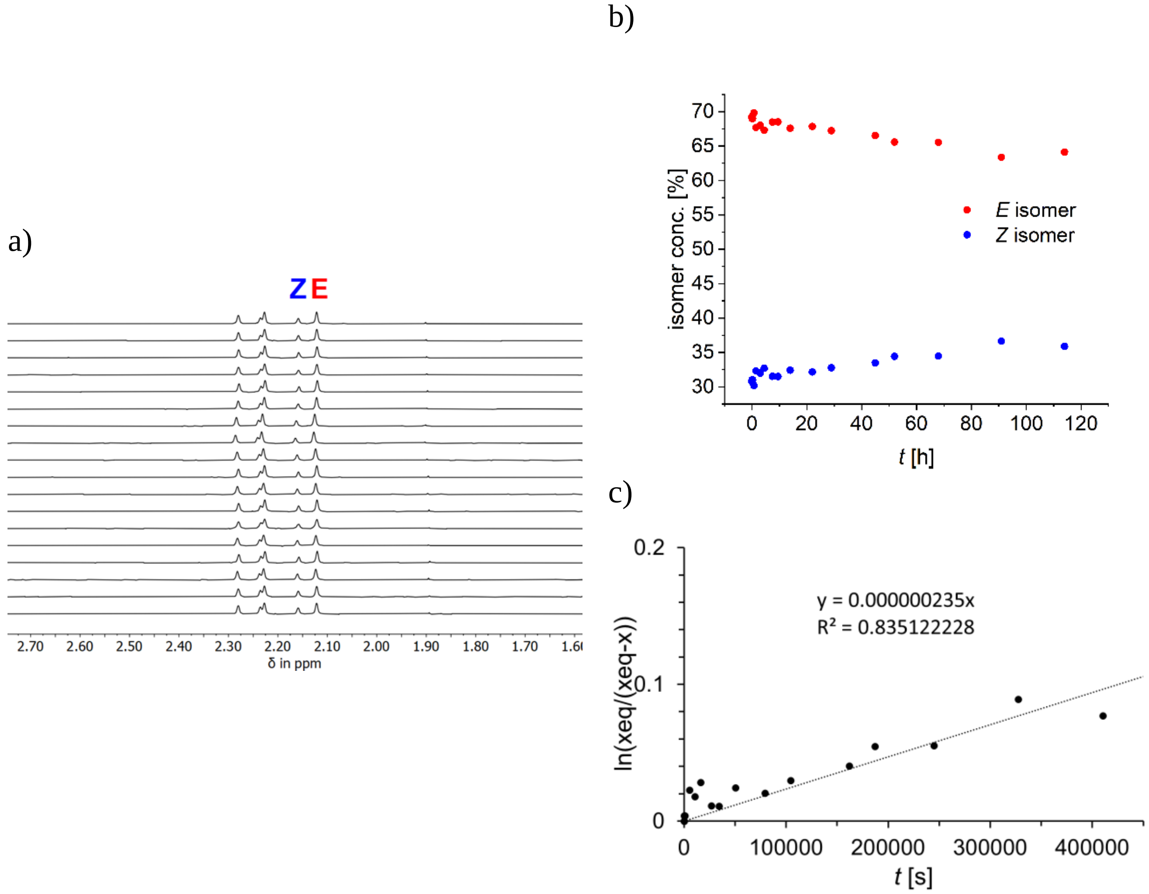

Supplementary Figure 6. Thermal *E* to *Z* isomerization of HPI **1**-OH in D_2O in the dark starting from *E* enriched solution, which was obtained by first irradiating the solution with 365 nm light. a) Thermal *E* to *Z* isomerization of HPI **1**-OH in D_2O at 90 °C was followed by ^{1}H NMR spectroscopy in the dark (400 MHz, 23 °C) in regular time intervals. b) Isomer conversion over time. c) First order kinetic analysis of the thermal isomerization of *E* to *Z* proceeding towards a thermal equilibrium. By plotting according to equation (3), the slope *m* can be translated into the rate constant k_E using equation (5). The corresponding *Gibbs* energy of activation $\Delta G_{E\rightarrow Z}^{\ddagger}$ for the thermal *E* to *Z* isomerization is given in Supplementary Table 2.

Note: Since the thermal *E* to *Z* isomerization is very slow, even at 90 °C, the thermal equilibrium could not be reached in reasonable time. For the first order kinetic analysis in c) an isomer composition at thermal equilibrium of *Z*/*E* =100/0 was assumed.

1.1.3 Thermal isomerization of HPI 2

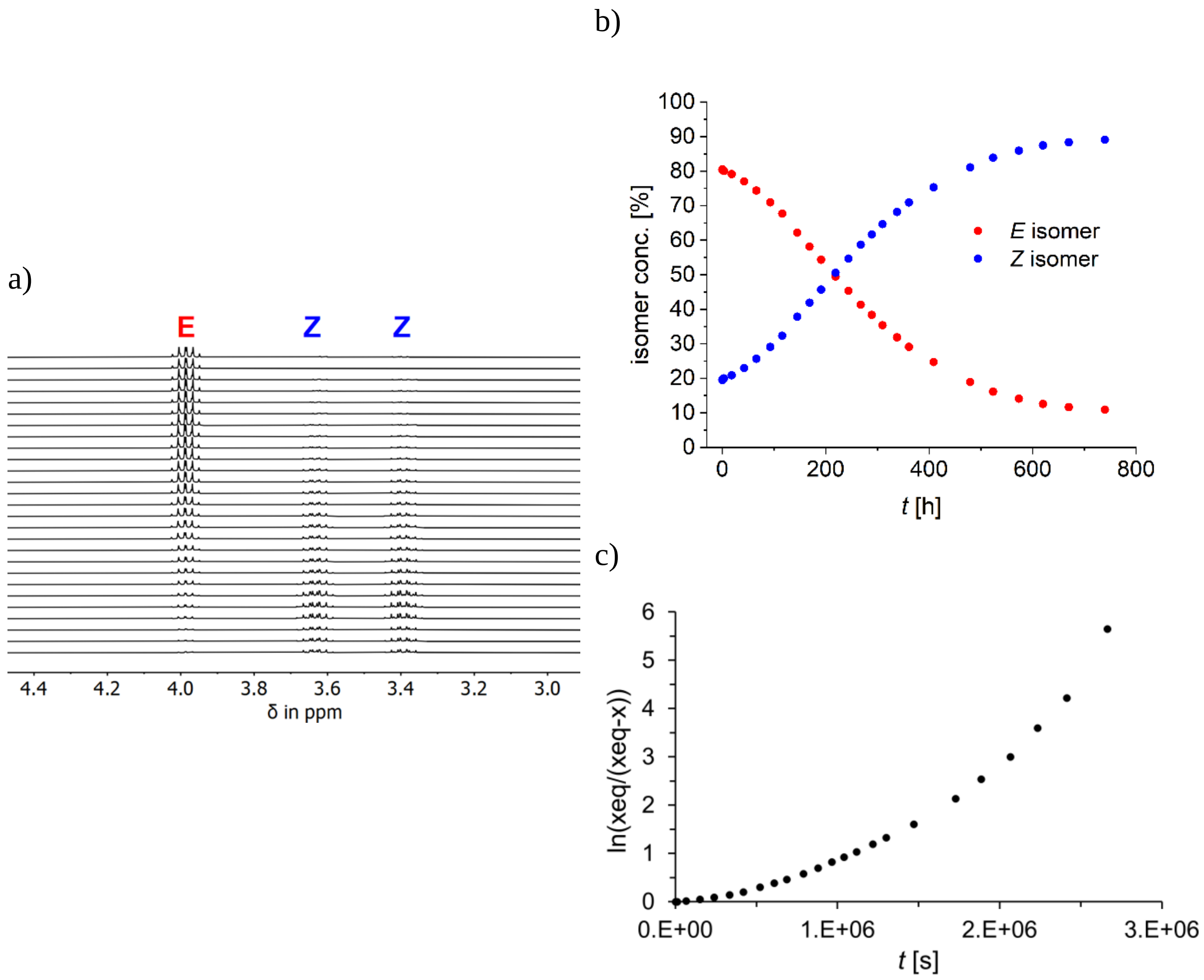

Supplementary Figure 7. Thermal *E* to *Z* isomerization of HPI **2** in *p*-xylene-d_{10} in the dark starting from *E* enriched solution, which was obtained by first irradiating the solution with 300 nm light. a) Thermal *E* to *Z* isomerization of HPI **2** in *p*-xylene-d_{10} at 130 °C in the dark was followed by ^{1}H NMR spectroscopy (400 MHz, 23 °C) in regular time intervals. b) Isomer conversion over time. c) First order kinetic analysis of the thermal isomerization of *E* to *Z* proceeding towards a thermal equilibrium. The sigmoidal shape of the curve indicates a more complex order for the thermal isomerization and is most probably due to aggregation. For this reason, no rate constant and corresponding *Gibbs* energy of activation $\Delta G^{\ddagger}$ could be determined. The half-life at 130 °C was determined by fitting a polynomial function to the data in b) and the value is given in Supplementary Table 2.

Note: For a rough estimation of the *Gibbs* energy of activation $\Delta G^{\ddagger}$, it was calculated for three data points (116 h, 219 h and 574 h) and in all cases exceeded 35.0 kcal/mol.

1.1.4 Thermal isomerization of HPI 2-OH

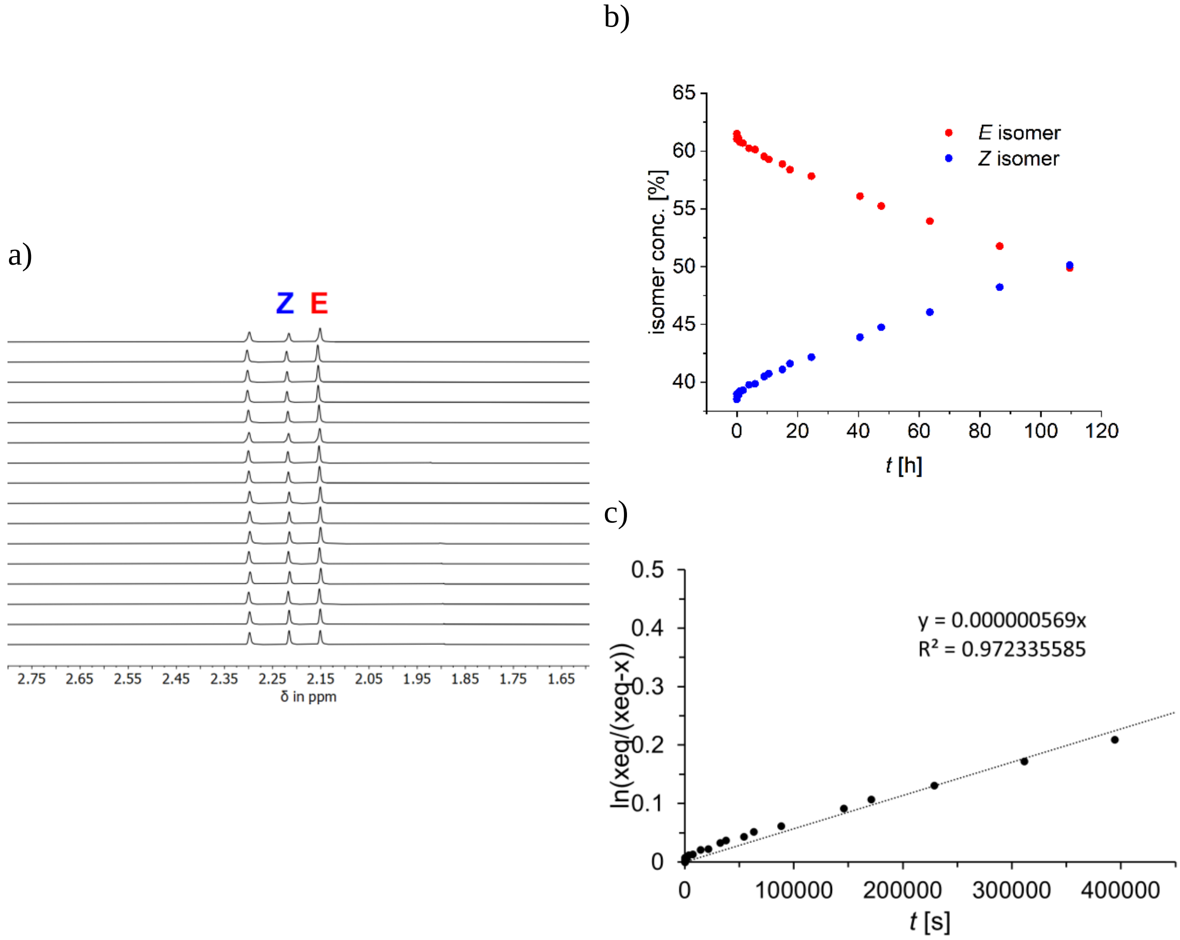

Supplementary Figure 8. Thermal *E* to *Z* isomerization of HPI **2**-OH in D_2O in the dark starting from *E* enriched solution, which was obtained by first irradiating the solution with 365 nm light. a) Thermal *E* to *Z* isomerization of HPI **2**-OH in D_2O at 90 °C was followed by ^{1}H NMR spectroscopy in the dark (400 MHz, 23 °C) in regular time intervals. b) Isomer conversion over time. c) First order kinetic analysis of the thermal isomerization of *E* to *Z* proceeding towards a thermal equilibrium. By plotting according to equation (3), the slope *m* can be translated into the rate constant k_E using equation (5). The corresponding *Gibbs* energy of activation $\Delta G_{E\rightarrow Z}^{\ddagger}$ for the thermal *E* to *Z* isomerization is given in Supplementary Table 2.

Note: Since the thermal *E* to *Z* isomerization is very slow, even at 90 °C, the thermal equilibrium could not be reached in reasonable time. For the first order kinetic analysis in c) an isomer composition at thermal equilibrium of *Z*/*E* =100/0 was assumed.

1.1.5 Thermal isomerization of HPI 3

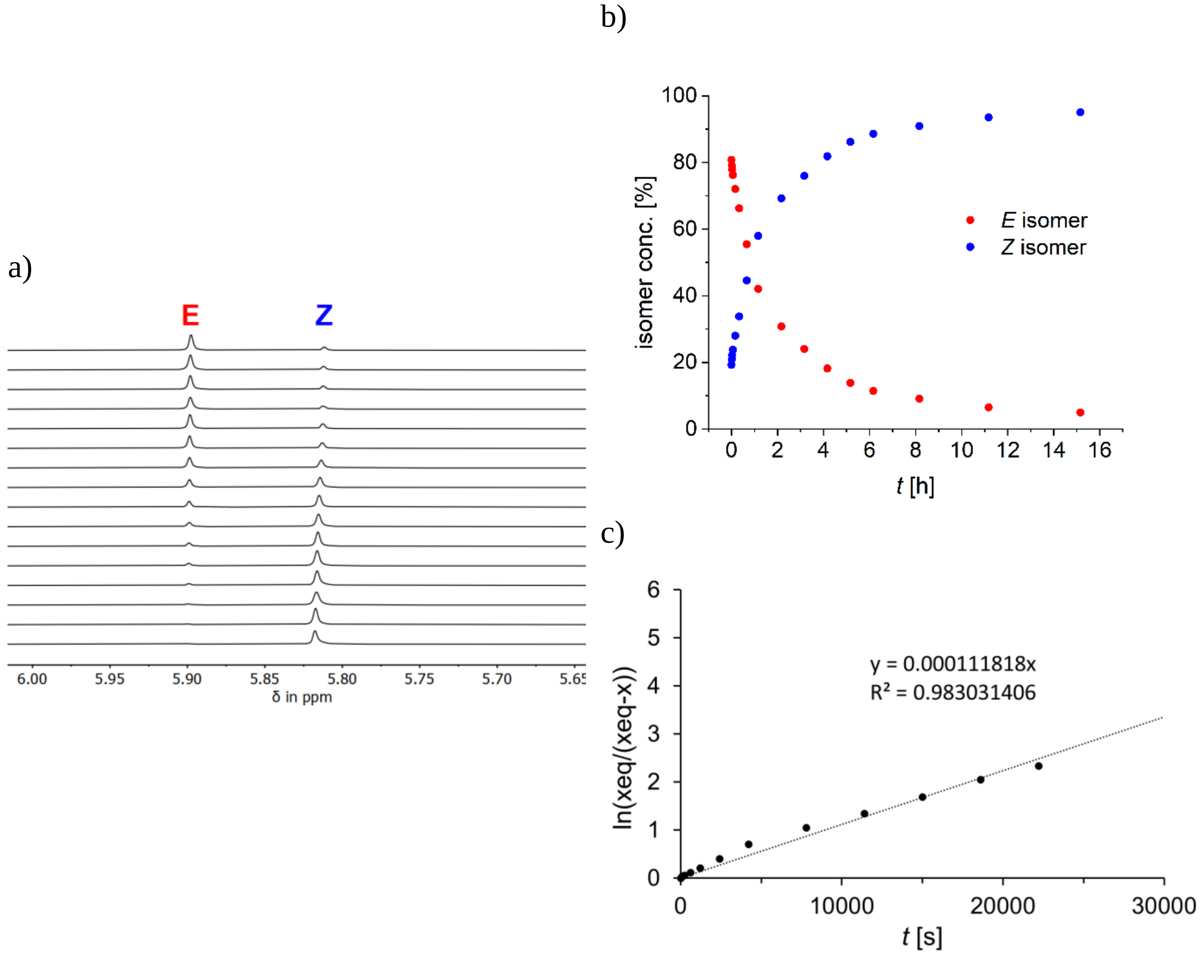

Supplementary Figure 9. Thermal *E* to *Z* isomerization of HPI **3** in toluene-d_8 in the dark starting from *E* enriched solution, which was obtained by first irradiating the solution with 395 nm light. a) Thermal *E* to *Z* isomerization of HPI **3** in toluene-d_8 at 100 °C in the dark was followed by ^{1}H NMR spectroscopy (400 MHz, 23 °C) in regular time intervals. b) Isomer conversion over time. c) First order kinetic analysis of the thermal isomerization of *E* to *Z* proceeding towards a thermal equilibrium. By plotting according to equation (3), the slope *m* can be translated into the rate constant k_E using equation (5). The corresponding *Gibbs* energy of activation $\Delta G_{E \rightarrow Z}^{\ddagger}$ for the thermal *E* to *Z* isomerization is given in Supplementary Table 2.

1.1.6 Thermal isomerization of HPI 3-OH

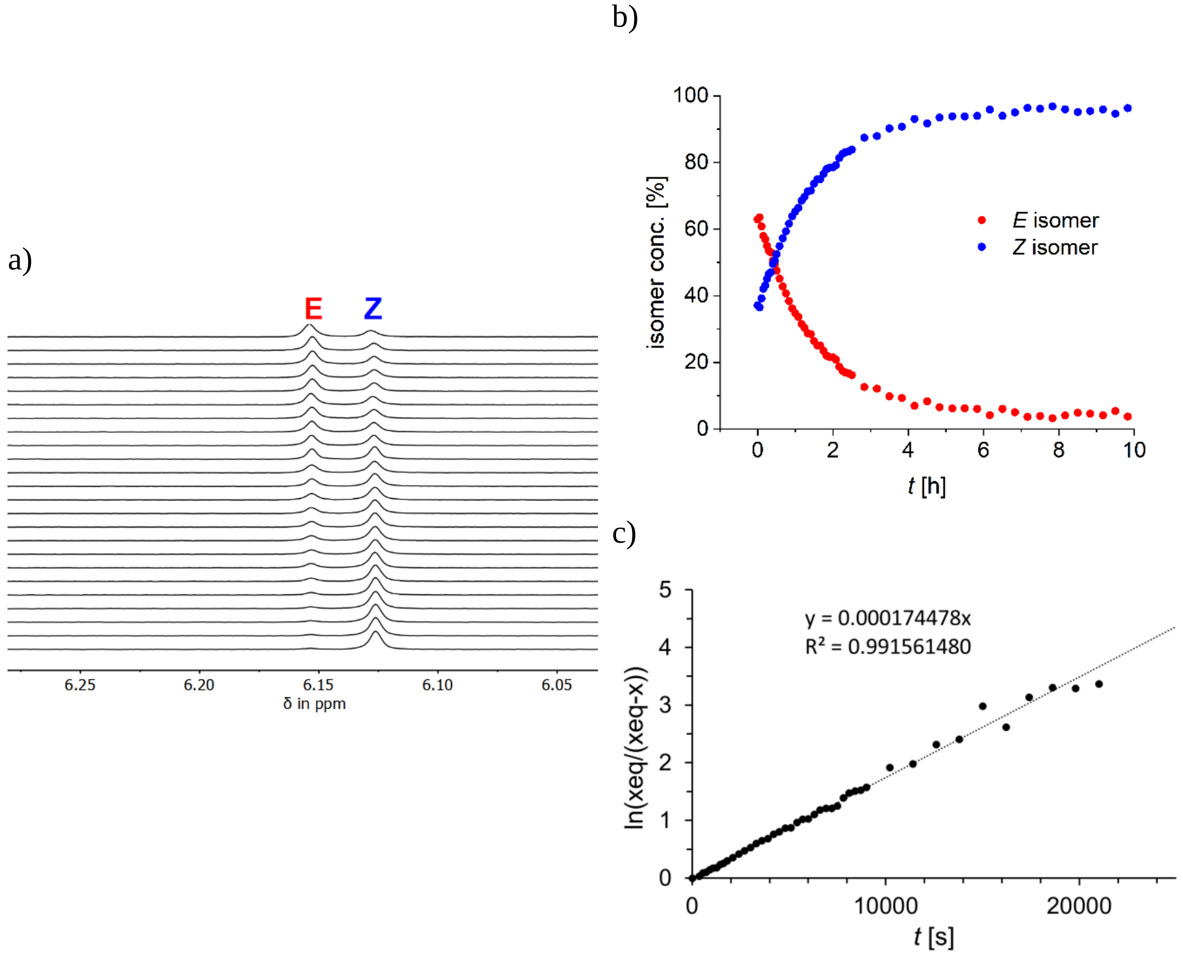

Supplementary Figure 10. Thermal *E* to *Z* isomerization of HPI **3**-OH in D_2O in the dark starting from *E* enriched solution, which was obtained by first irradiating the solution with 405 nm light. a) Thermal *E* to *Z* isomerization of HPI **3**-OH in D_2O at 6 °C was followed by ^1H NMR spectroscopy in the dark (400 MHz, 6 °C) in regular time intervals. b) Isomer conversion over time. c) First order kinetic analysis of the thermal isomerization of *E* to *Z* proceeding towards a thermal equilibrium. By plotting according to equation (3), the slope *m* can be translated into the rate constant k_E using equation (5). The corresponding *Gibbs* energy of activation $\Delta G_{E\rightarrow Z}^{\ddagger}$ for the thermal *E* to *Z* isomerization is given in Supplementary Table 2.

1.1.7 Thermal isomerization of HPI 4

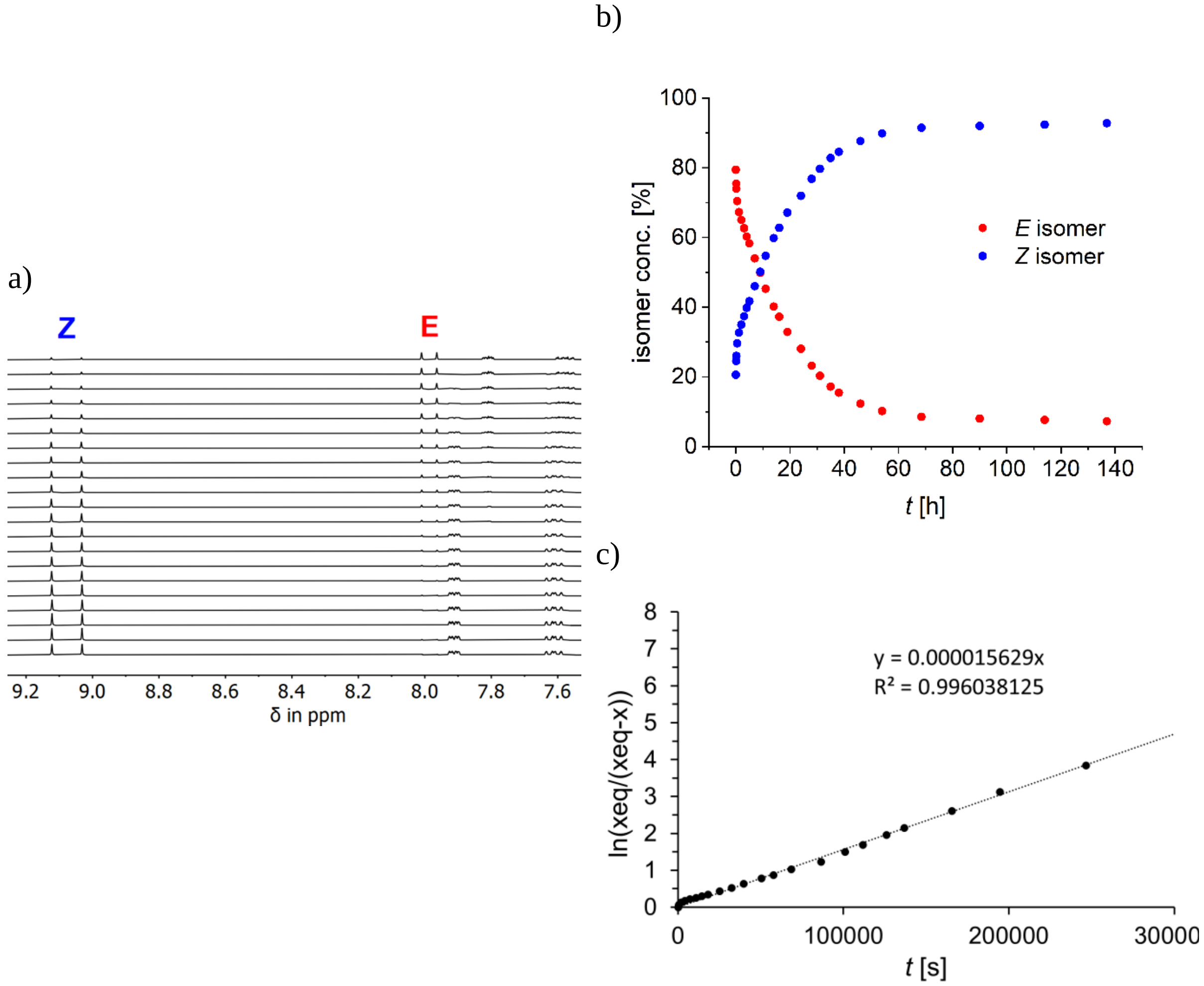

Supplementary Figure 11. Thermal *E* to *Z* isomerization of HPI **4** in toluene-d_8 in the dark starting from *E* enriched solution, which was obtained by first irradiating the solution with 395 nm light. a) Thermal *E* to *Z* isomerization of HPI **4** in toluene-d_8 at 100 °C was followed by ^{1}H NMR spectroscopy in the dark (400 MHz, 23 °C) in regular time intervals. b) Isomer conversion over time. c) First order kinetic analysis of the thermal isomerization of *E* to *Z* proceeding towards a thermal equilibrium. By plotting according to equation (3), the slope *m* can be translated into the rate constant k_E using equation (5). The corresponding *Gibbs* energy of activation $\Delta G_{E\rightarrow Z}^{\ddagger}$ for the thermal *E* to *Z* isomerization is given in Supplementary Table 2.

1.1.8 Thermal isomerization of HPI 5

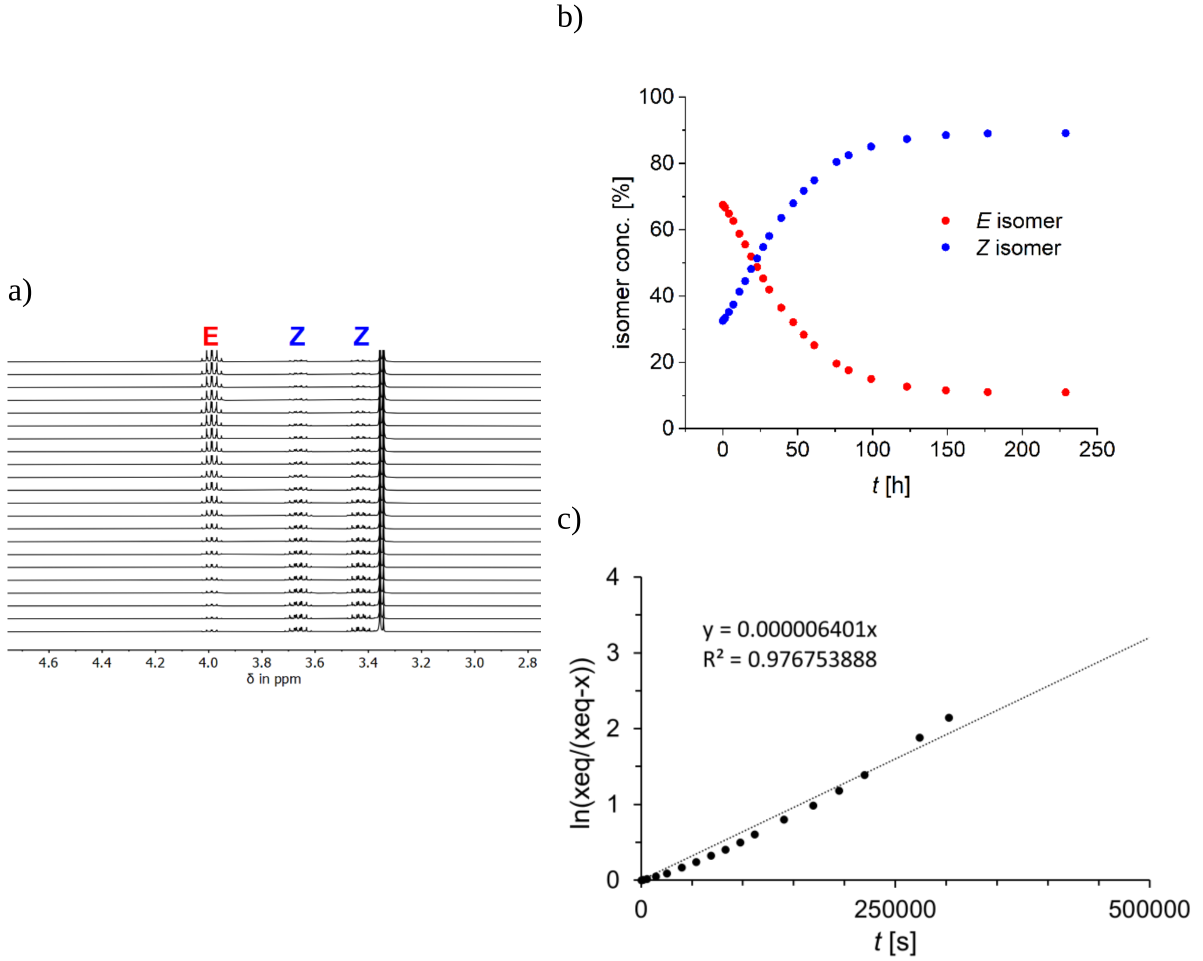

Supplementary Figure 12. Thermal *E* to *Z* isomerization of HPI **5** in *p*-xylene-d_{10} in the dark starting from *E* enriched solution, which was obtained by first irradiating the solution with 395 nm light. a) Thermal *E* to *Z* isomerization of HPI **5** in *p*-xylene-d_{10} at 130 °C in the dark was followed by ^{1}H NMR spectroscopy (400 MHz, 23 °C) in regular time intervals. b) Isomer conversion over time. c) First order kinetic analysis of the thermal isomerization of *E* to *Z* proceeding towards a thermal equilibrium. By plotting according to equation (3), the slope *m* can be translated into the rate constant k_E using equation (5). The corresponding *Gibbs* energy of activation $\Delta G_{E\to Z}^{\ddagger}$ for the thermal *E* to *Z* isomerization is given in Supplementary Table 2.

1.1.9 Thermal isomerization of HPI 6

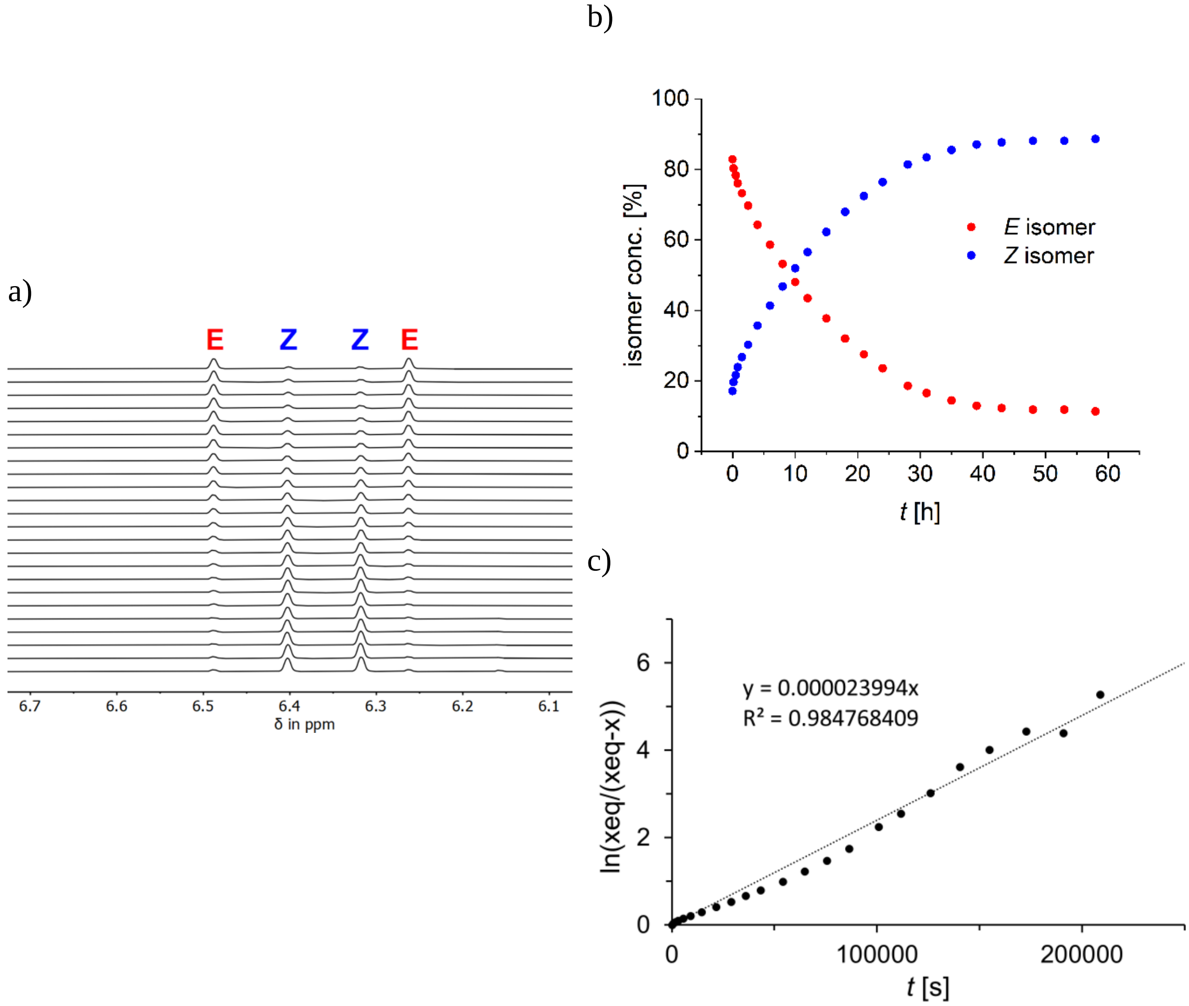

Supplementary Figure 13. Thermal *E* to *Z* isomerization of HPI **6** in toluene-d_8 in the dark starting from *E* enriched solution, which was obtained by first irradiating the solution with 395 nm light. a) Thermal *E* to *Z* isomerization of HPI **6** in toluene-d_8 at 100 °C in the dark was followed by ^{1}H NMR spectroscopy (400 MHz, 23 °C) in regular time intervals. b) Isomer conversion over time. c) First order kinetic analysis of the thermal isomerization of *E* to *Z* proceeding towards a thermal equilibrium. By plotting according to equation (3), the slope *m* can be translated into the rate constant k_E using equation (5). The corresponding *Gibbs* energy of activation $\Delta G_{E\rightarrow Z}^{\ddagger}$ for the thermal *E* to *Z* isomerization is given in Supplementary Table 2.

1.1.10 Thermal isomerization of HPI 7

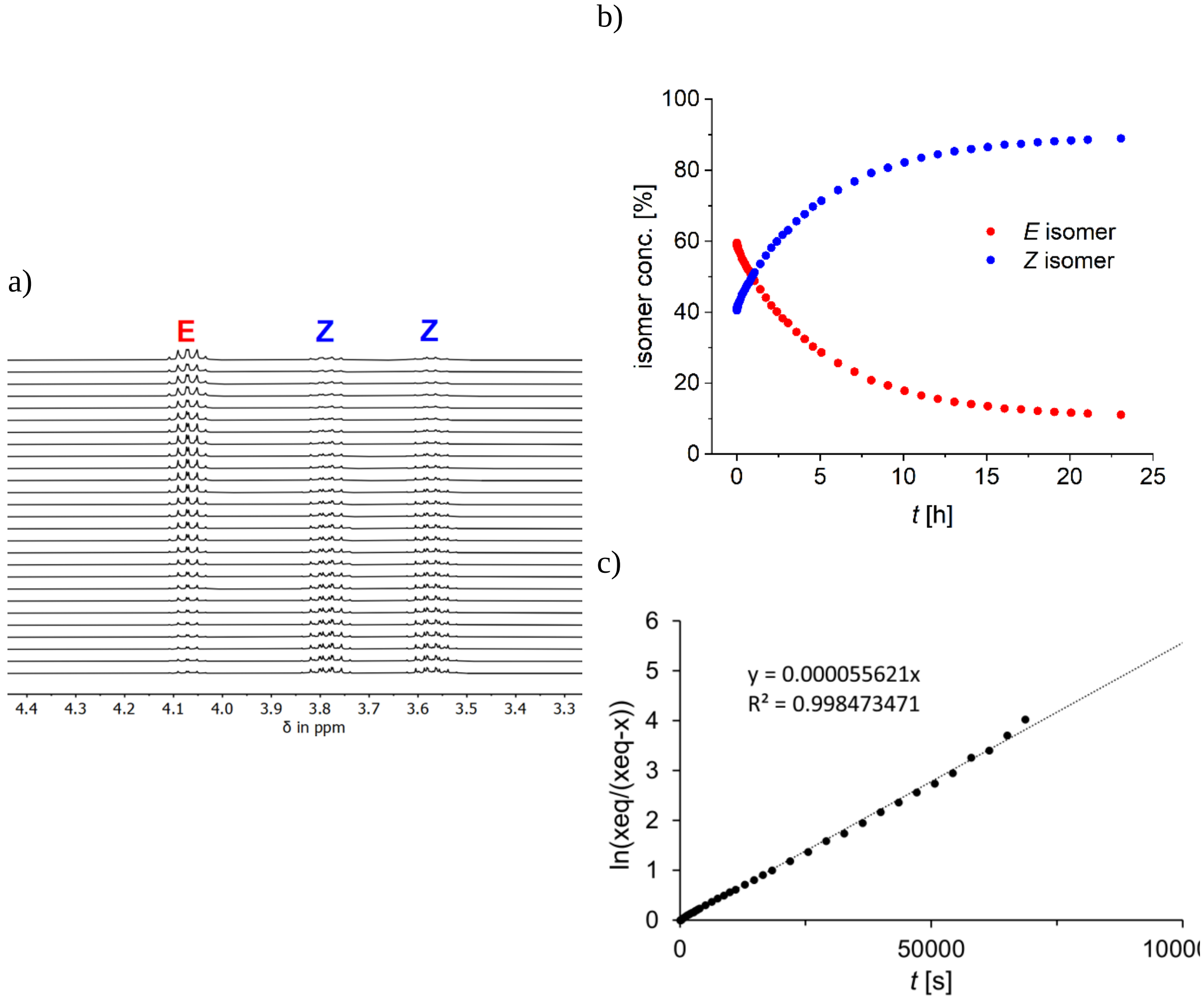

Supplementary Figure 14. Thermal *E* to *Z* isomerization of HPI **7** in toluene-d_8 in the dark starting from *E* enriched solution, which was obtained by first irradiating the solution with 430 nm light. a) Thermal *E* to *Z* isomerization of HPI **7** in toluene-d_8 at 65 °C in the dark was followed by ^{1}H NMR spectroscopy (400 MHz, 65 °C) in regular time intervals. b) Isomer conversion over time. c) First order kinetic analysis of the thermal isomerization of *E* to *Z* proceeding towards a thermal equilibrium. By plotting according to equation (3), the slope *m* can be translated into the rate constant k_E using equation (5). The corresponding *Gibbs* energy of activation $\Delta G_{E\rightarrow Z}^{\ddagger}$ for the thermal *E* to *Z* isomerization is given in Supplementary Table 2.

1.1.11 Thermal isomerization of HPI 8

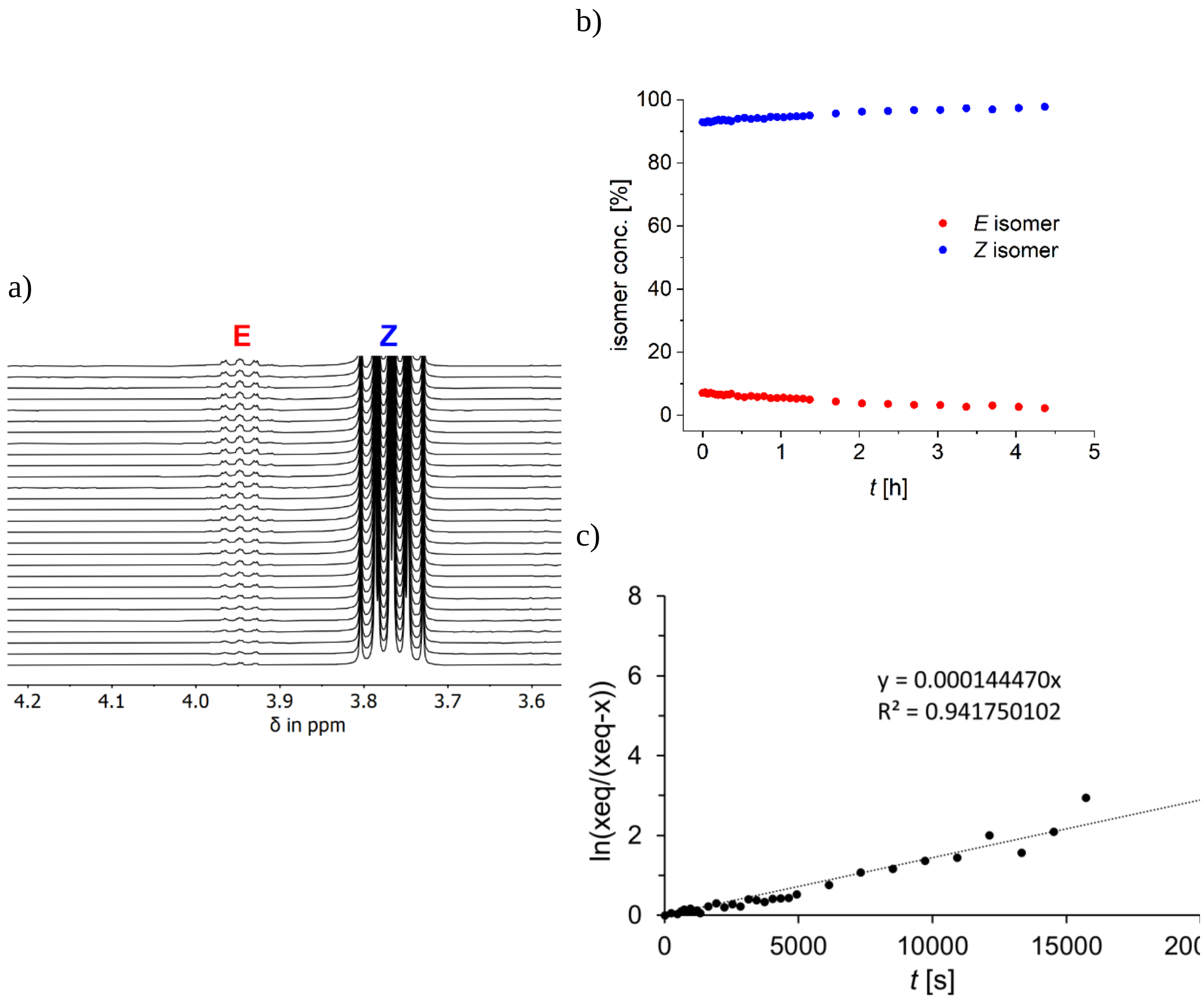

Supplementary Figure 15. Thermal *E* to *Z* isomerization of HPI **8** in toluene-d_8 in the dark starting from *E* enriched solution, which was obtained by first irradiating the solution with 470 nm light. a) Thermal *E* to *Z* isomerization of HPI **8** in toluene-d_8 at 25 °C in the dark was followed by ^{1}H NMR spectroscopy (400 MHz, 25 °C) in regular time intervals. b) Isomer conversion over time. c) First order kinetic analysis of the thermal isomerization of *E* to *Z* proceeding towards a thermal equilibrium. By plotting according to equation (3), the slope *m* can be translated into the rate constant k_E using equation (5). The corresponding *Gibbs* energy of activation $\Delta G_{E\rightarrow Z}^{\ddagger}$ for the thermal *E* to *Z* isomerization is given in Supplementary Table 2.

1.1.12 Thermal isomerization of HPI 9

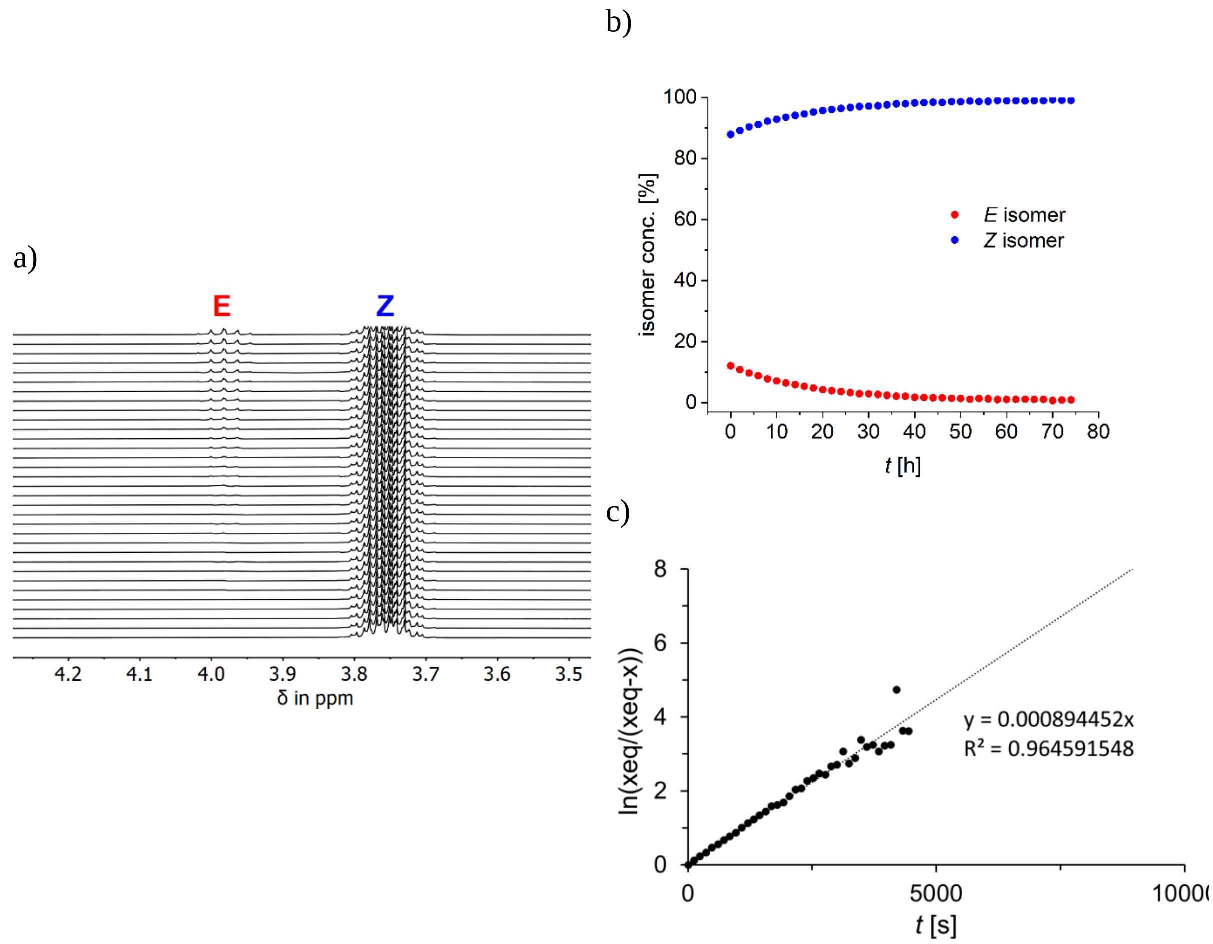

Supplementary Figure 16. Thermal *E* to *Z* isomerization of HPI **9** in toluene-d_8 in the dark starting from *E* enriched solution, which was obtained by first irradiating the solution with 450 nm light. a) Thermal *E* to *Z* isomerization of HPI **9** in toluene-d_8 at 30 °C in the dark was followed by ^{1}H NMR spectroscopy (400 MHz, 30 °C) in regular time intervals. b) Isomer conversion over time. c) First order kinetic analysis of the thermal isomerization of *E* to *Z* proceeding towards a thermal equilibrium. By plotting according to equation (3), the slope *m* can be translated into the rate constant k_E using equation (5). The corresponding *Gibbs* energy of activation $\Delta G_{E\rightarrow Z}^{\ddagger}$ for the thermal *E* to *Z* isomerization is given in Supplementary Table 2.

1.1.13 Thermal isomerization of HPI 10

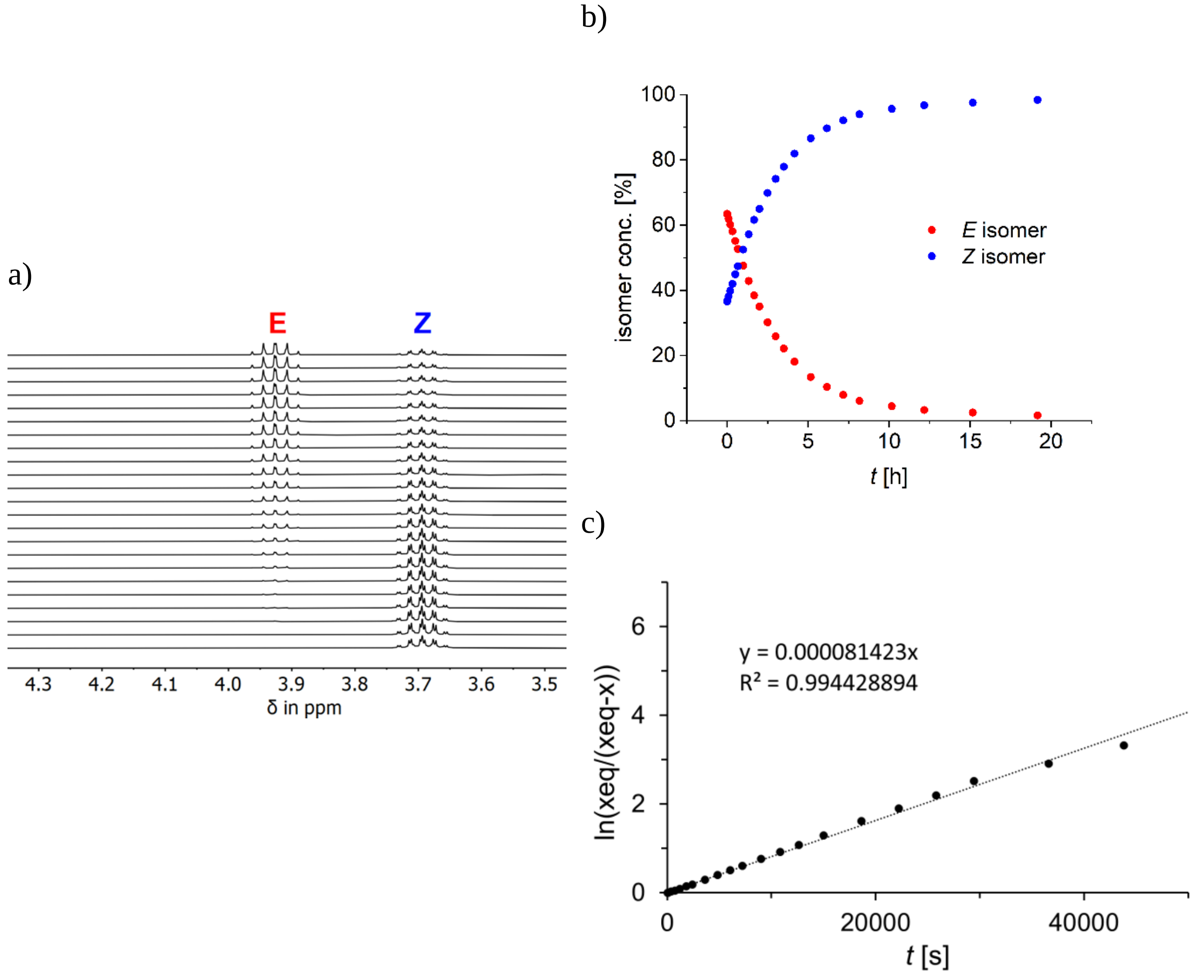

Supplementary Figure 17. Thermal *E* to *Z* isomerization of HPI **10** in toluene-d_8 in the dark starting from *E* enriched solution, which was obtained by first irradiating the solution with 365 nm light. a) Thermal *E* to *Z* isomerization of HPI **10** in toluene-d_8 at 100 °C in the dark was followed by ^{1}H NMR spectroscopy (400 MHz, 23 °C) in regular time intervals. b) Isomer conversion over time. c) First order kinetic analysis of the thermal isomerization of *E* to *Z* proceeding towards a thermal equilibrium. By plotting according to equation (3), the slope *m* can be translated into the rate constant k_E using equation (5). The corresponding *Gibbs* energy of activation $\Delta G_{E\rightarrow Z}^{\ddagger}$ for the thermal *E* to *Z* isomerization is given in Supplementary Table 2.

1.1.14 Thermal isomerization of HPI 11

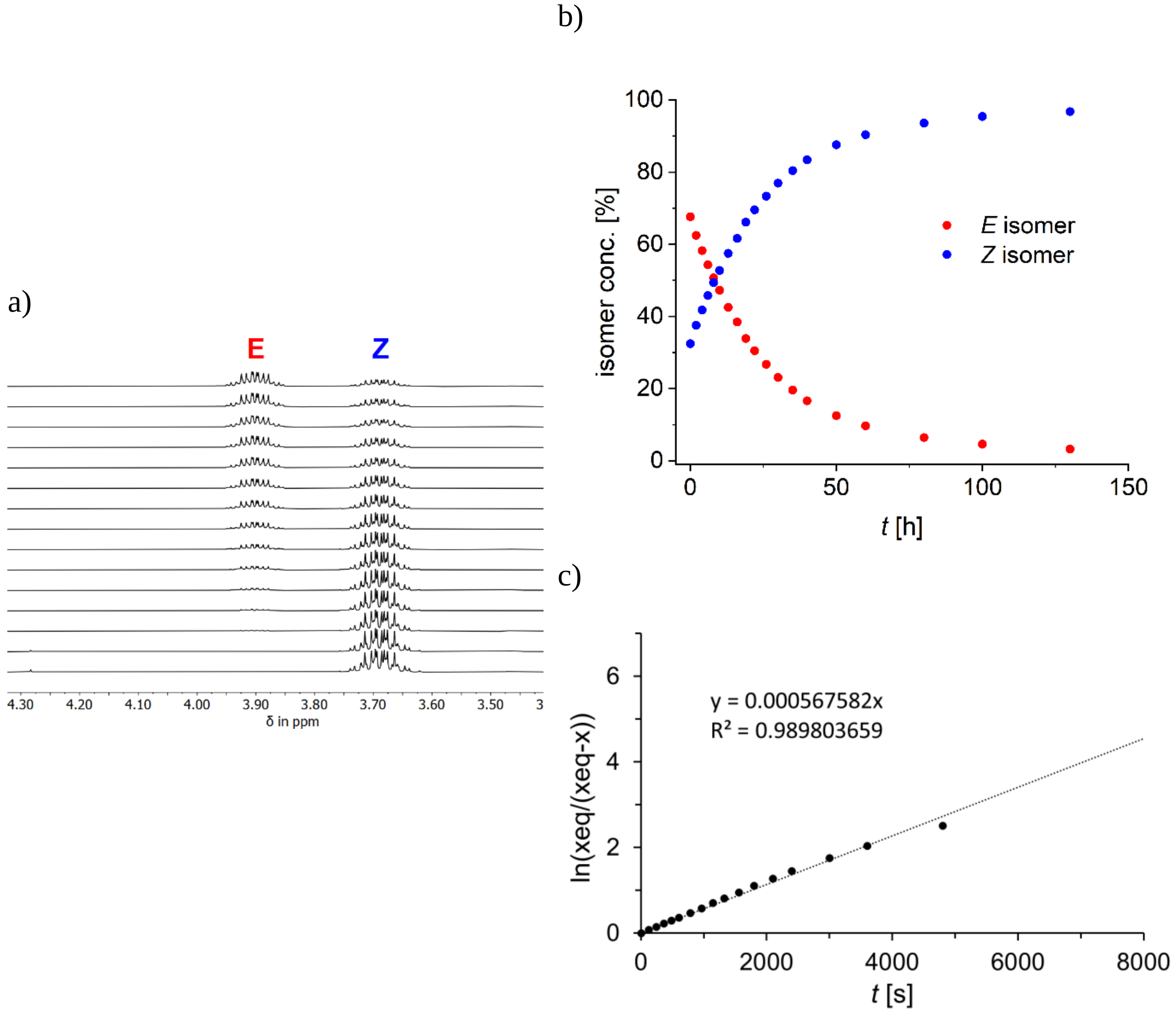

Supplementary Figure 18. Thermal *E* to *Z* isomerization of HPI **11** in toluene-d_8 in the dark starting from *E* enriched solution, which was obtained by first irradiating the solution with 340 nm light. a) Thermal *E* to *Z* isomerization of HPI **11** in toluene-d_8 at 100 °C in the dark was followed by ^{1}H NMR spectroscopy (400 MHz, 23 °C) in regular time intervals. b) Isomer conversion over time. c) First order kinetic analysis of the thermal isomerization of *E* to *Z* proceeding towards a thermal equilibrium. By plotting according to equation (3), the slope *m* can be translated into the rate constant k_E using equation (5). The corresponding *Gibbs* energy of activation $\Delta G_{E\rightarrow Z}^{\ddagger}$ for the thermal *E* to *Z* isomerization is given in Supplementary Table 2.

1.1.15 Thermal isomerization of HPI 11-OH

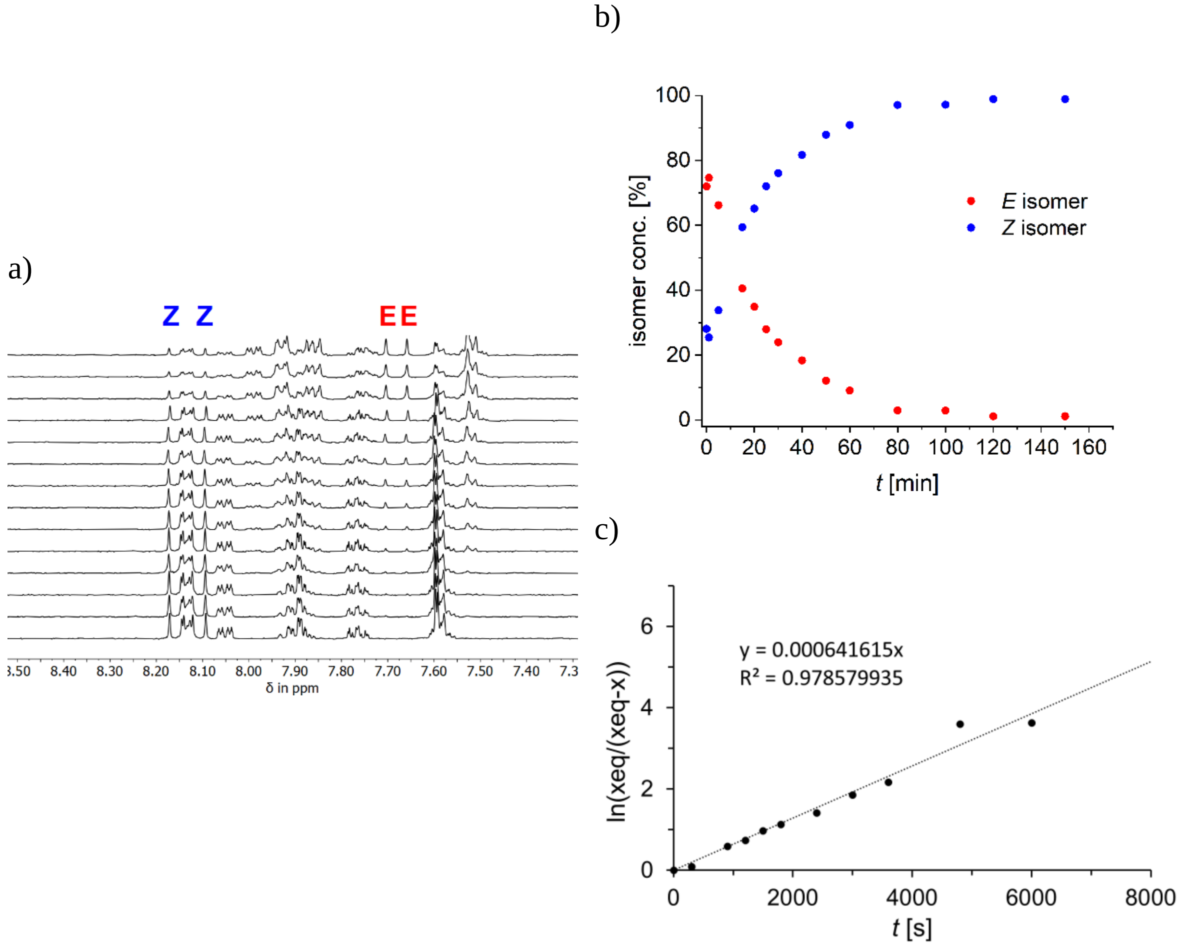

Supplementary Figure 19. Thermal *E* to *Z* isomerization of HPI **11**-OH in D_2O in the dark starting from *E* enriched solution, which was obtained by first irradiating the solution with 385 nm light. a) Thermal *E* to *Z* isomerization of HPI **11**-OH in D_2O at 90 °C was followed by ^{1}H NMR spectroscopy in the dark (400 MHz, 23 °C) in regular time intervals. b) Isomer conversion over time. c) First order kinetic analysis of the thermal isomerization of *E* to *Z* proceeding towards a thermal equilibrium. By plotting according to equation (3), the slope *m* can be translated into the rate constant k_E using equation (5). The corresponding *Gibbs* energy of activation $\Delta G_{E\rightarrow Z}^{\ddagger}$ for the thermal *E* to *Z* isomerization is given in Supplementary Table 2.

1.1.16 Thermal isomerization of HPI 12

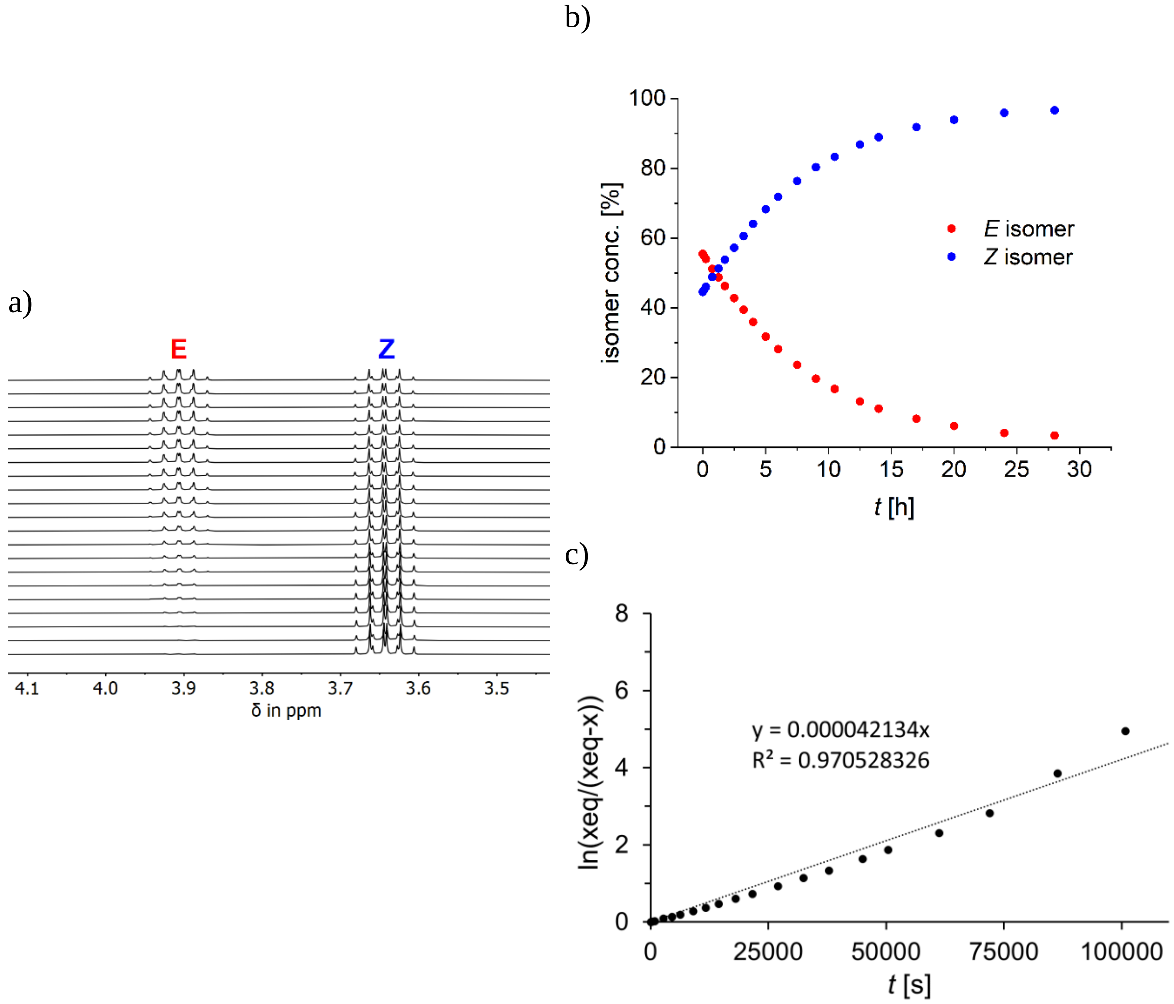

Supplementary Figure 20. Thermal *E* to *Z* isomerization of HPI **12** in toluene-d_8 in the dark starting from *E* enriched solution, which was obtained by first irradiating the solution with 450 nm light. a) Thermal *E* to *Z* isomerization of HPI **12** in toluene-d_8 at 100 °C in the dark was followed by ^{1}H NMR spectroscopy (400 MHz, 23 °C) in regular time intervals. b) Isomer conversion over time. c) First order kinetic analysis of the thermal isomerization of *E* to *Z* proceeding towards a thermal equilibrium. By plotting according to equation (3), the slope *m* can be translated into the rate constant k_E using equation (5). The corresponding *Gibbs* energy of activation $\Delta G_{E\rightarrow Z}^{\ddagger}$ for the thermal *E* to *Z* isomerization is given in Supplementary Table 2.

1.1.17 Thermal isomerization of HPI 13

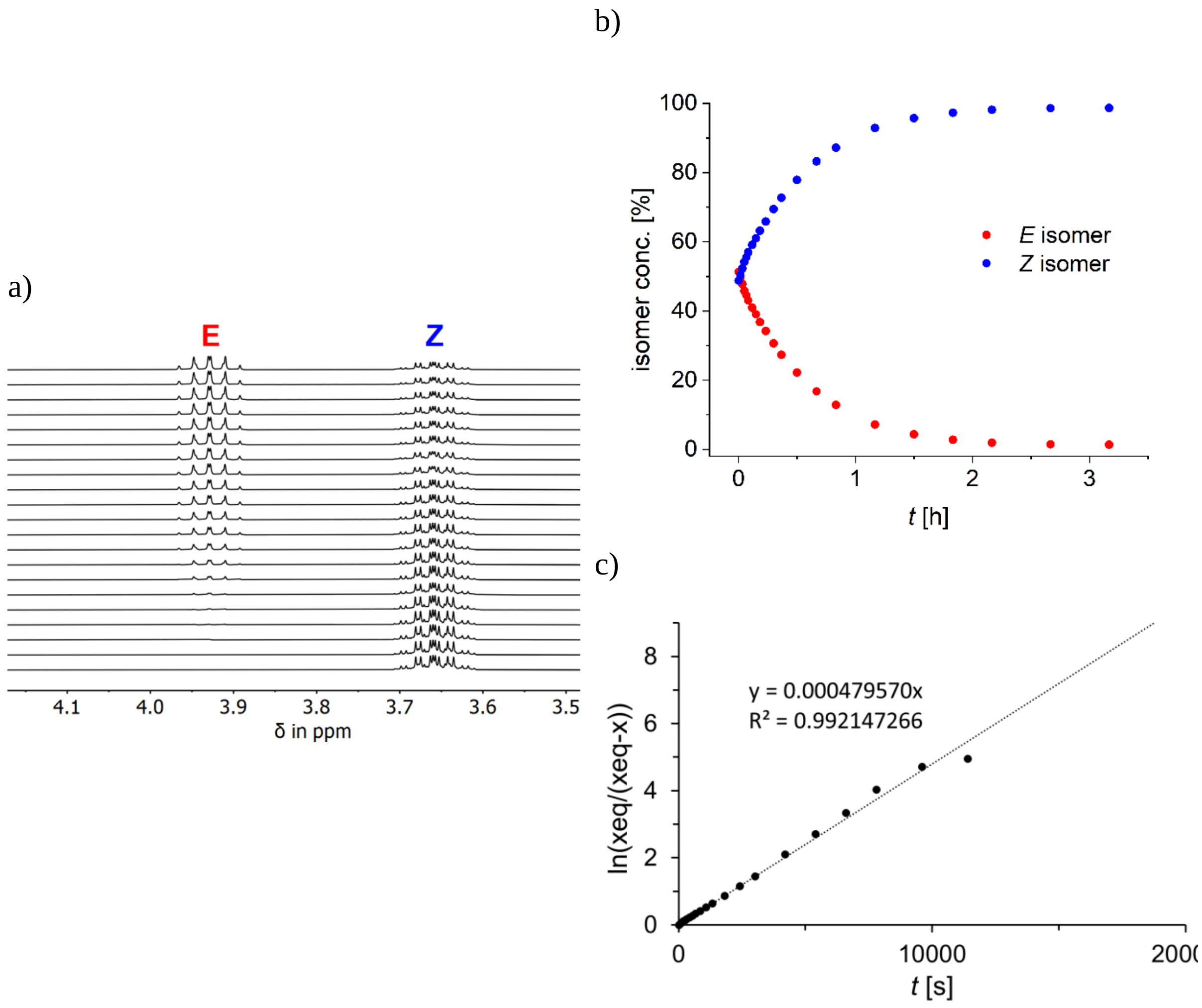

Supplementary Figure 21. Thermal *E* to *Z* isomerization of HPI **13** in toluene-d_8 in the dark starting from *E* enriched solution, which was obtained by first irradiating the solution with 340 nm light. a) Thermal *E* to *Z* isomerization of HPI **13** in toluene-d_8 at 100 °C in the dark was followed by ^{1}H NMR spectroscopy (400 MHz, 23 °C) in regular time intervals. b) Isomer conversion over time. c) First order kinetic analysis of the thermal isomerization of *E* to *Z* proceeding towards a thermal equilibrium. By plotting according to equation (3), the slope *m* can be translated into the rate constant k_E using equation (5). The corresponding *Gibbs* energy of activation $\Delta G_{E\rightarrow Z}^{\ddagger}$ for the thermal *E* to *Z* isomerization is given in Supplementary Table 2.

1.1.18 Thermal isomerization of HPI 14

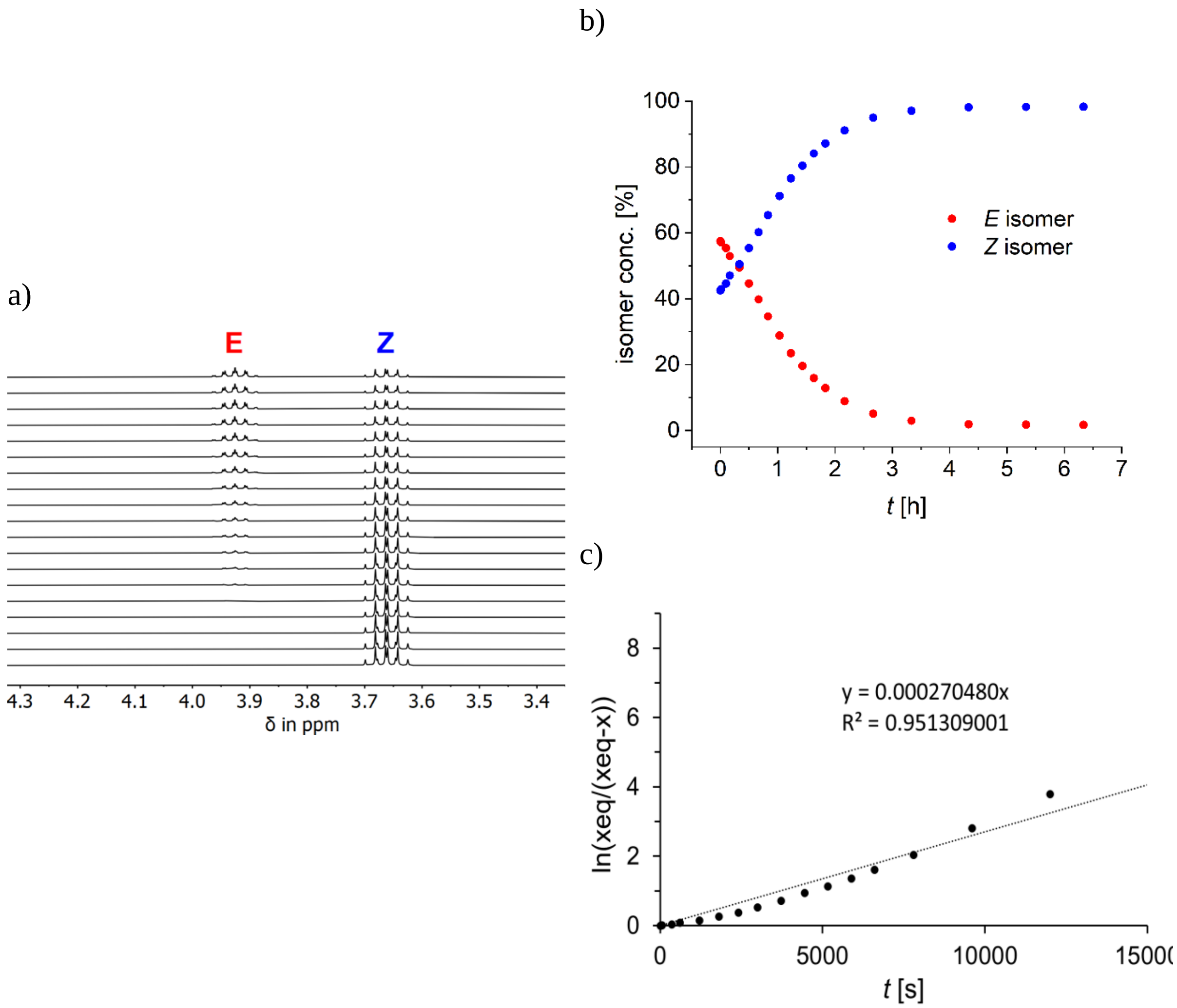

Supplementary Figure 22. Thermal *E* to *Z* isomerization of HPI **14** in toluene-d_8 in the dark starting from *E* enriched solution, which was obtained by first irradiating the solution with 365 nm light. a) Thermal *E* to *Z* isomerization of HPI **14** in toluene-d_8 at 100 °C in the dark was followed by ^{1}H NMR spectroscopy (400 MHz, 23 °C) in regular time intervals. b) Isomer conversion over time. c) First order kinetic analysis of the thermal isomerization of *E* to *Z* proceeding towards a thermal equilibrium. By plotting according to equation (3), the slope *m* can be translated into the rate constant k_E using equation (5). The corresponding *Gibbs* energy of activation $\Delta G_{E \rightarrow Z}^{\ddagger}$ for the thermal *E* to *Z* isomerization is given in Supplementary Table 2.

1.1.19 Thermal isomerization of HPI 15

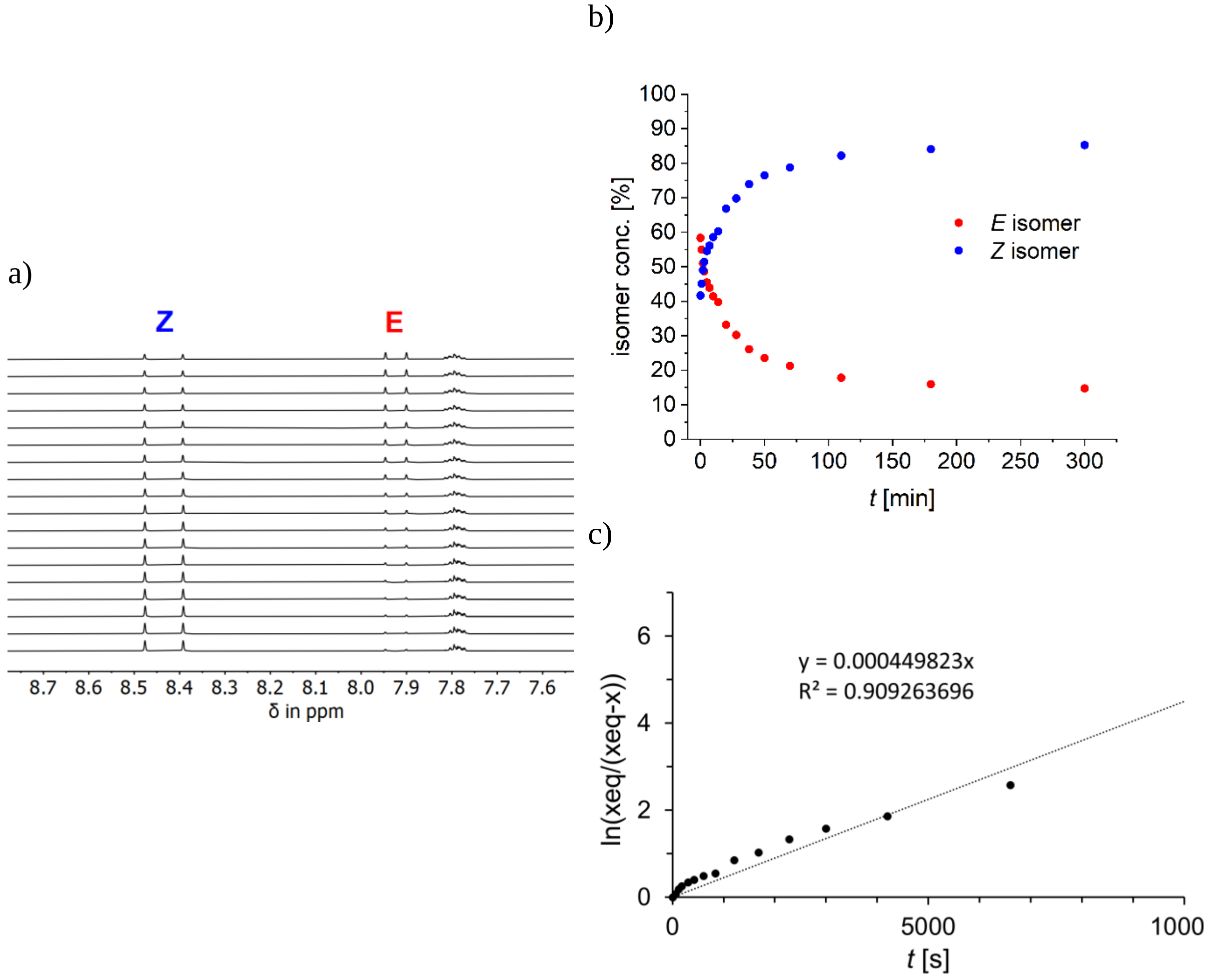

Supplementary Figure 23. Thermal *E* to *Z* isomerization of HPI **15** in toluene-d_8 in the dark starting from *E* enriched solution, which was obtained by first irradiating the solution with 365 nm light. a) Thermal *E* to *Z* isomerization of HPI **15** in toluene-d_8 at 50 °C in the dark was followed by ^{1}H NMR spectroscopy (400 MHz, 23 °C) in regular time intervals. b) Isomer conversion over time. c) First order kinetic analysis of the thermal isomerization of *E* to *Z* proceeding towards a thermal equilibrium. By plotting according to equation (3), the slope *m* can be translated into the rate constant k_E using equation (5). The corresponding *Gibbs* energy of activation $\Delta G_{E\rightarrow Z}^{\ddagger}$ for the thermal *E* to *Z* isomerization is given in Supplementary Table 2.

1.1.20 Thermal isomerization of HPI 16

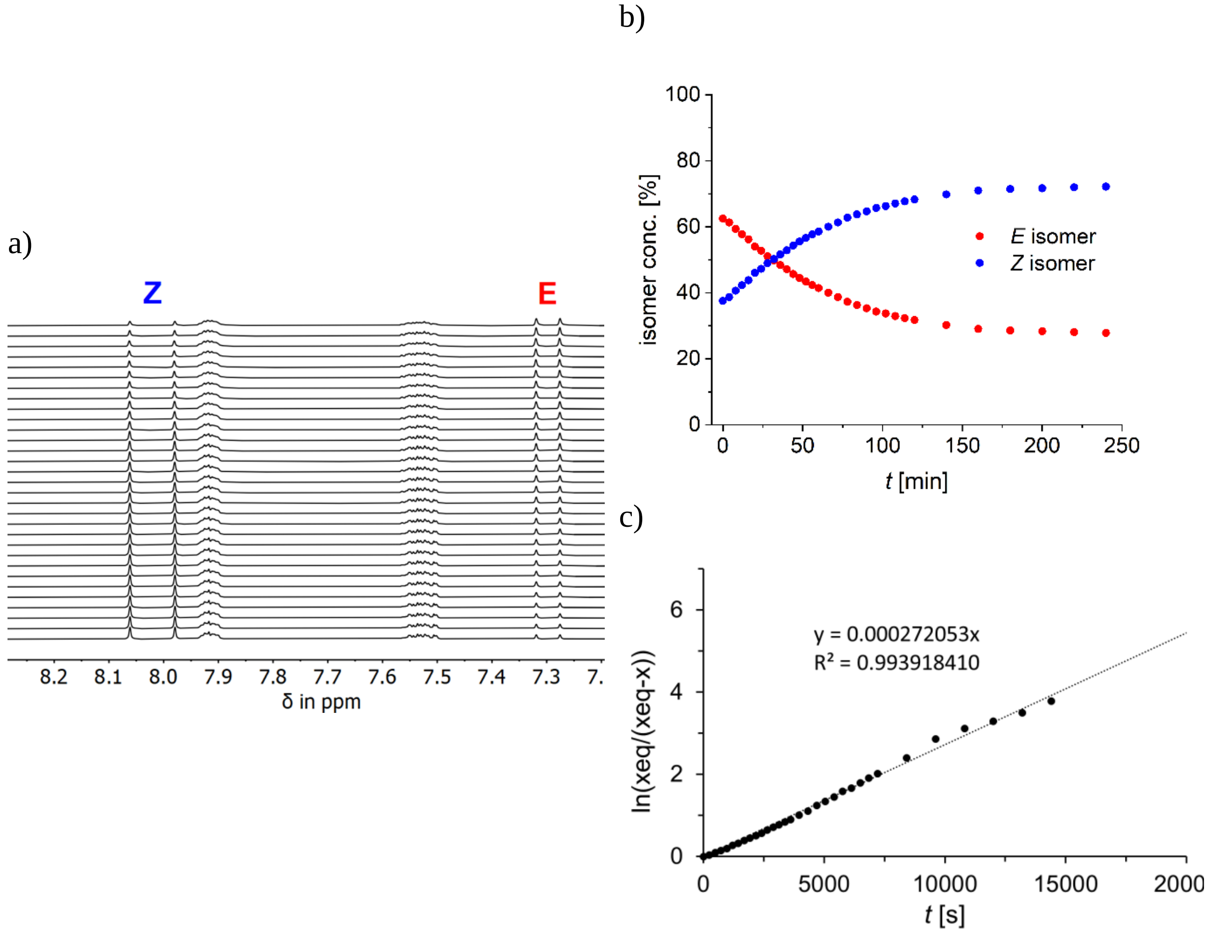

Supplementary Figure 24. Thermal *E* to *Z* isomerization of HPI **16** in toluene-d_8 in the dark starting from *E* enriched solution, which was obtained by first irradiating the solution with 340 nm light. a) Thermal *E* to *Z* isomerization of HPI **16** in toluene-d_8 at 75 °C in the dark was followed by ^{1}H NMR spectroscopy (400 MHz, 75 °C) in regular time intervals. b) Isomer conversion over time. c) First order kinetic analysis of the thermal isomerization of *E* to *Z* proceeding towards a thermal equilibrium. By plotting according to equation (3), the slope *m* can be translated into the rate constant k_E using equation (5). The corresponding *Gibbs* energy of activation $\Delta G_{E\rightarrow Z}^{\ddagger}$ for the thermal *E* to *Z* isomerization is given in Supplementary Table 2.

1.1.21 Thermal isomerization of HPI 17

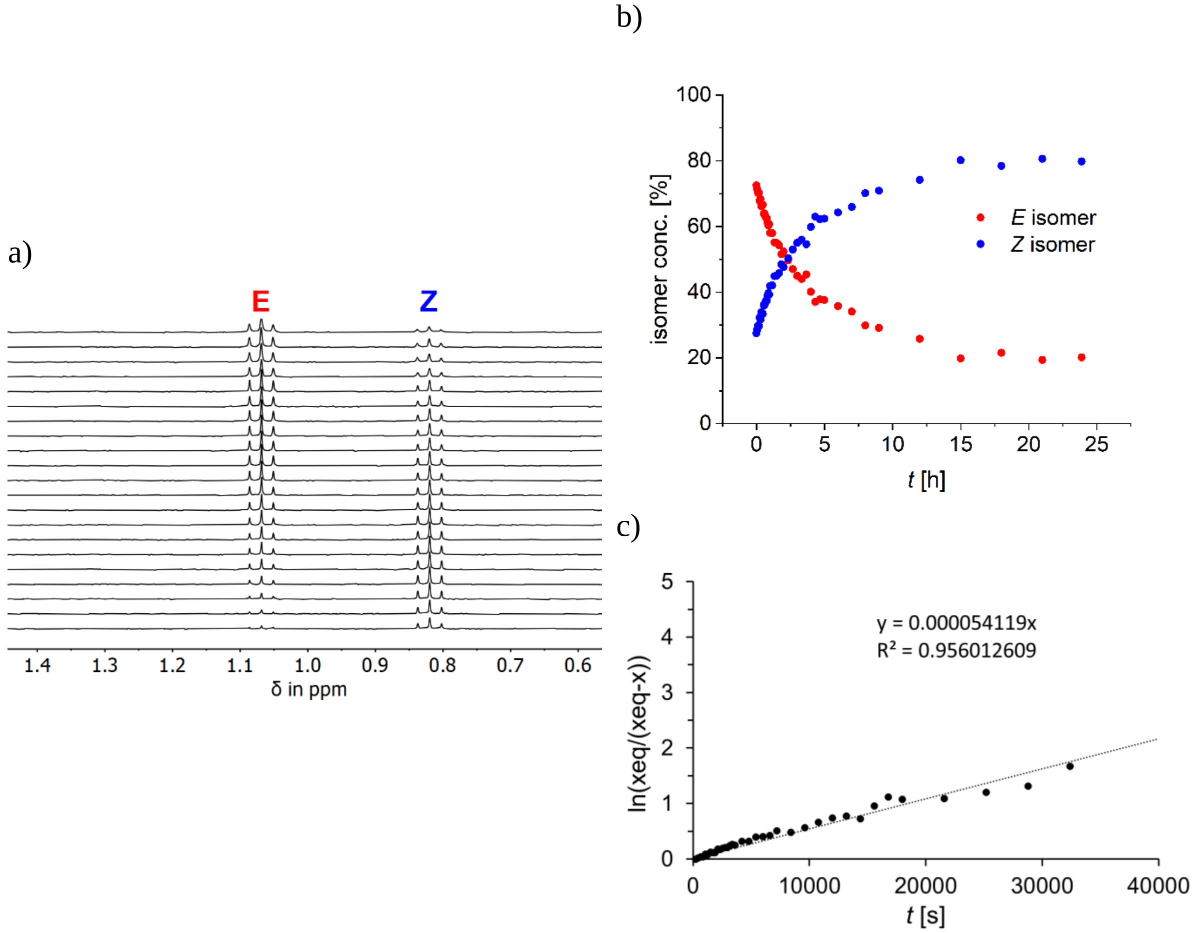

Supplementary Figure 25. Thermal *E* to *Z* isomerization of HPI **17** in toluene-d_8 in the dark starting from *E* enriched solution, which was obtained by first irradiating the solution with 340 nm light. a) Thermal *E* to *Z* isomerization of HPI **17** in toluene-d_8 at 100 °C in the dark was followed by ^{1}H NMR spectroscopy (400 MHz, 100 °C) in regular time intervals. b) Isomer conversion over time. c) First order kinetic analysis of the thermal isomerization of *E* to *Z* proceeding towards a thermal equilibrium. By plotting according to equation (3), the slope *m* can be translated into the rate constant k_E using equation (5). The corresponding *Gibbs* energy of activation $\Delta G_{E\rightarrow Z}^{\ddagger}$ for the thermal *E* to *Z* isomerization is given in Supplementary Table 2.

1.1.22 Thermal isomerization of HPI 17-OH

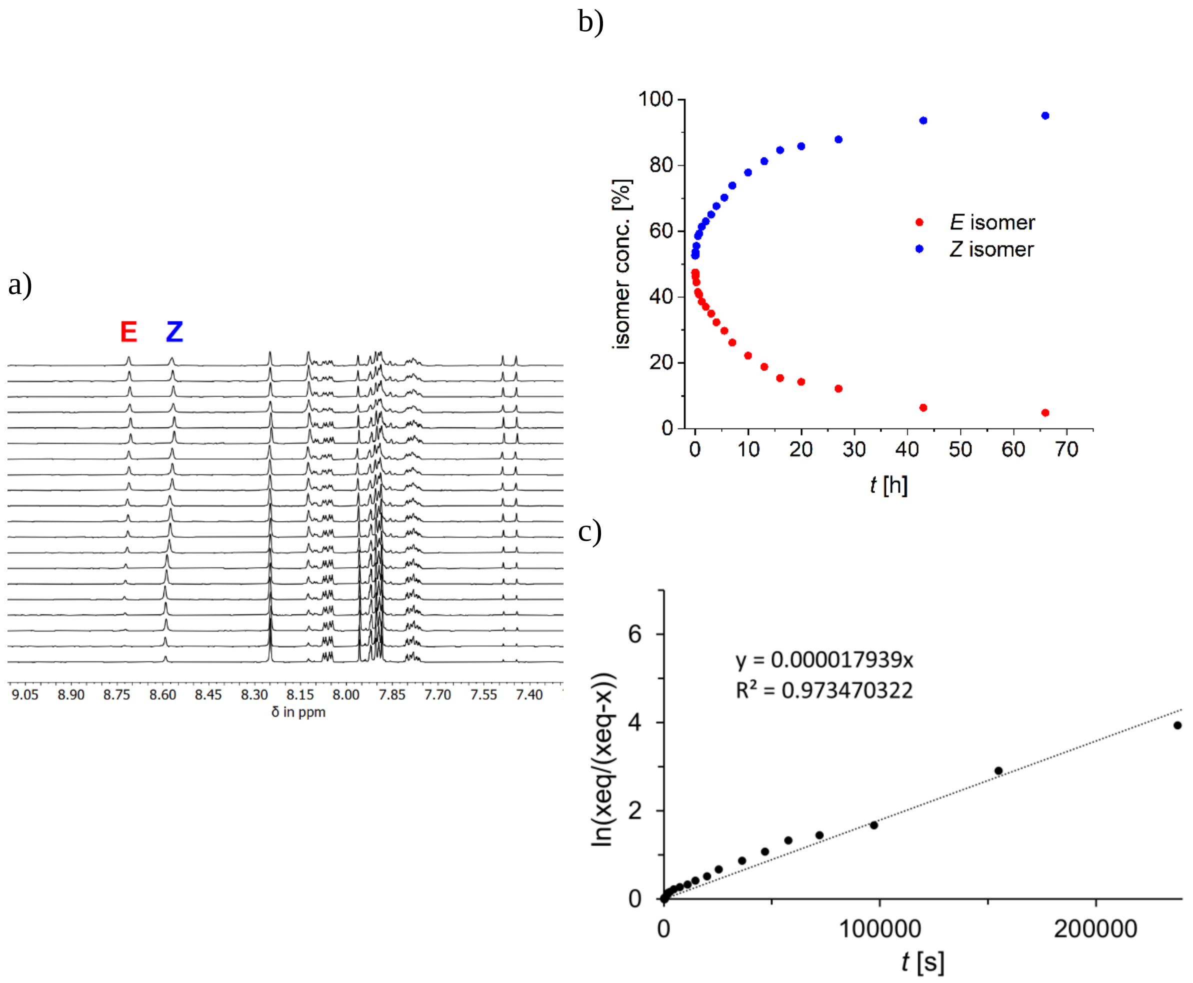

Supplementary Figure 26. Thermal *E* to *Z* isomerization of HPI **17**-OH in D_2O in the dark starting from *E* enriched solution, which was obtained by first irradiating the solution with 365 nm light. a) Thermal *E* to *Z* isomerization of HPI **17**-OH in D_2O at 50 °C was followed by ^{1}H NMR spectroscopy in the dark (400 MHz, 23 °C) in regular time intervals. b) Isomer conversion over time. c) First order kinetic analysis of the thermal isomerization of *E* to *Z* proceeding towards a thermal equilibrium. By plotting according to equation (3), the slope *m* can be translated into the rate constant k_E using equation (5). The corresponding *Gibbs* energy of activation $\Delta G_{E\rightarrow Z}^{\ddagger}$ for the thermal *E* to *Z* isomerization is given in Supplementary Table 2.

1.1.23 Thermal isomerization of HPI 18

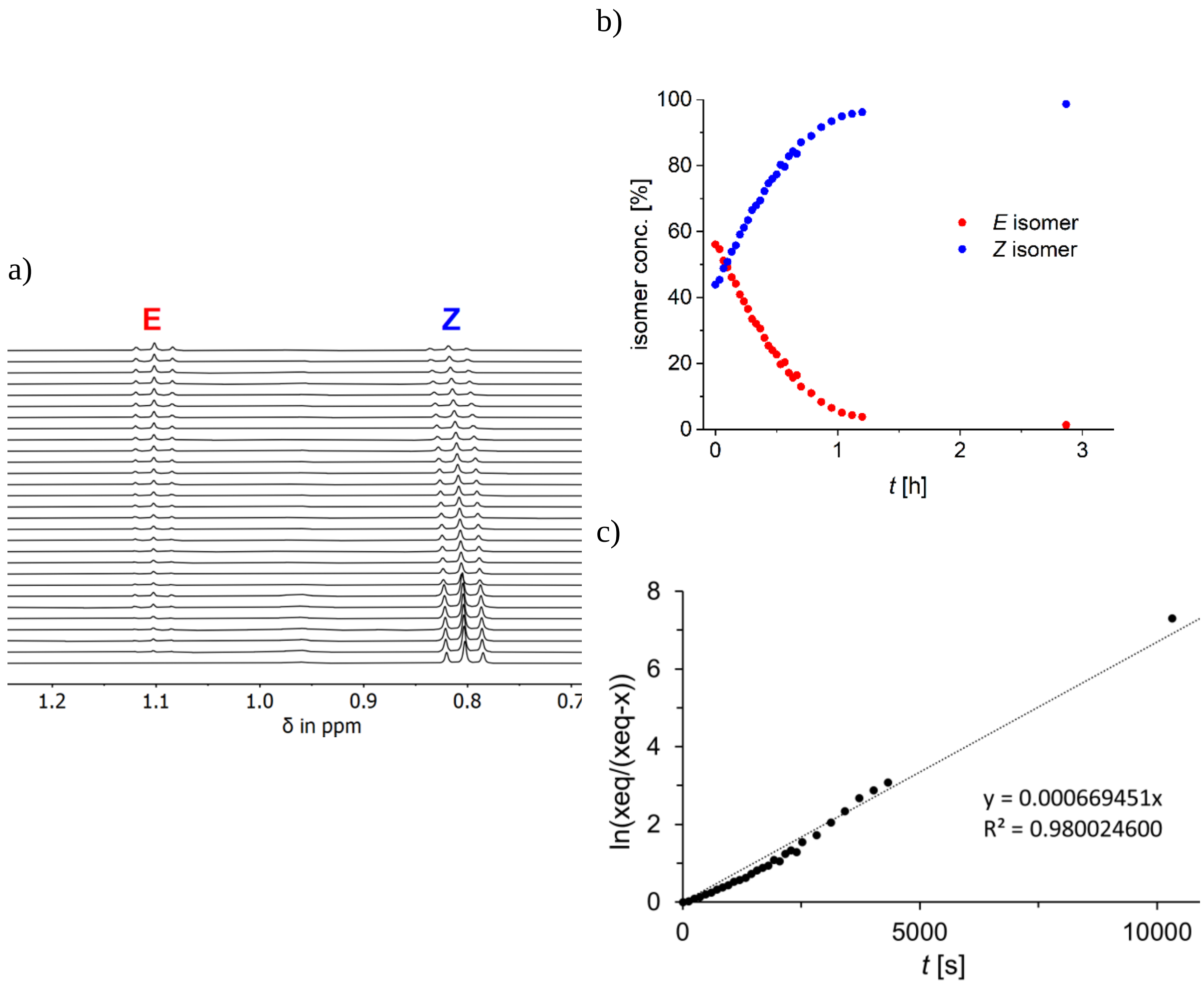

Supplementary Figure 27. Thermal *E* to *Z* isomerization of HPI **18** in toluene-d_8 in the dark starting from *E* enriched solution, which was obtained by first irradiating the solution with 430 nm light. a) Thermal *E* to *Z* isomerization of HPI **18** in toluene-d_8 at 30 °C in the dark was followed by ^{1}H NMR spectroscopy (400 MHz, 30 °C) in regular time intervals. b) Isomer conversion over time. c) First order kinetic analysis of the thermal isomerization of *E* to *Z* proceeding towards a thermal equilibrium. By plotting according to equation (3), the slope *m* can be translated into the rate constant k_E using equation (5). The corresponding *Gibbs* energy of activation $\Delta G_{E\rightarrow Z}^{\ddagger}$ for the thermal *E* to *Z* isomerization is given in Supplementary Table 2.

1.1.24 Summary of all thermal isomerization reactions

Supplementary Table 2. Thermal isomerization behavior of the *E* isomers of different HPI photoswitches in toluene-d_8,*p*-xylene-d_{10} and D_2O at different temperatures. The values for the *Gibbs* energies of activation for the *E* to *Z* isomerization $\Delta G_{E\rightarrow Z}^{\ddagger}$ were obtained by plotting experimental kinetic data according to equation (3) and subsequently using equation (5) and equation (7). Thermal half-lives at 298 K are calculated using the *Eyring* equation (9) and the approximation that the energy barrier for the isomerization $\Delta G^{\ddagger}$ is temperature independent.

**HPI**	**Temperature *T* [°C]**	**Solvent**	***Z* isomer at thermal equilibrium [%]**	**Half-life of *E* isomer at indicated *T***	**k_E [s^{-1}] at indicated *T***	**$\Delta G^{\ddagger}_{E\rightarrow Z}$ [kcal/mol] at indicated *T***	**Half-life of *E* isomer at 298 K**
**1**	130 °C	*p*-xylene-d_{10}	> 86	691 h	-	> 35.5	> 380000 a
**1-OH**	90 °C	D_2O	unknown[a)]	819 h [a)]	2.35×10^{-7} [a)]	32.4 [a)]	2100 a [a)]
**2**	130 °C	*p*-xylene-d_{10}	> 90	273 h	-	> 35.0	> 165000 a
**2-OH**	90 °C	D_2O	unknown[a)]	338 h [a)]	5.69×10^{-7} [a)]	31.8 [a)]	714 a [a)]
**3**	100 °C	toluene-d_8	96	1.7 h	1.07×10^{-4}	28.8	4 a
**3-OH**	6 °C	D_2O	96	1.1 h	1.67×10^{-4}	21.1	6 min
**4**	100 °C	toluene-d_8	93	12.3 h	1.45×10^{-5}	30.3	50 a
**5**	130 °C	*p*-xylene-d_{10}	89	30.1 h	5.70×10^{-6}	33.5	11500 a
**6**	100 °C	toluene-d_8	89	8.0 h	2.14×10^{-5}	30.0	30 a
**7**	65 °C	toluene-d_8	89	3.5 h	4.95×10^{-5}	26.5	33 d
**8**	25 °C	toluene-d_8	98	1.4 h	1.42×10^{-4}	22.7	1.4 h
**9**	30 °C	toluene-d_8	99	13 min	8.89×10^{-4}	22.0	25 min
**10**	100 °C	toluene-d_8	99	2.4 h	8.06×10^{-5}	29.0	7 a

**11**	100 °C	toluene-d_8	99	21 min	$5.62x10^{-4}$	27.6	210 d
**11-OH**	90 °C	D_2O	99	18 min	$6.35x10^{-4}$	26.7	50 d
**12**	100 °C	toluene-d_8	97	4.7 h	$4.09x10^{-5}$	29.5	15 a
**13**	100 °C	toluene-d_8	99	24 min	$4.75x10^{-4}$	27.7	260 d
**14**	100 °C	toluene-d_8	98	43 min	$2.66x10^{-4}$	28.1	1.5 a
**15**	50 °C	toluene-d_8	86	26 min	$3.85x10^{-5}$	24.0	3 d
**16**	75 °C	toluene-d_8	73	42 min	$1.99x10^{-4}$	26.4	5 d
**17**	100 °C	toluene-d_8	80	4.5 h	$4.33x10^{-5}$	29.5	14 a
**17-OH**	50 °C	D_2O	96	11.2 h	$1.72x10^{-5}$	26.0	15 d
**18**	30 °C	toluene-d_8	99	17 min	$6.61x10^{-4}$	22.2	34 min

a) Very slow thermal isomerization. For the kinetic analysis, an isomer composition at thermal equilibrium of *Z*/*E* =100/0 was assumed.

Supplementary Note 5: Photochromism of HPIs

1.1.25 Determination of molar absorption coefficients of twisted and heterocyclic HPIs

In case of the the twisted and heterocyclic HPI derivatives, the synthesis did not yield the pure *Z* isomers. For this reason, the molar absorption coefficients of pure *E* and *Z* isomers were obtained from solutions containing a mixture of both isomers. This is possible if the following prerequisites are met:

1) The total concentration of *E* and *Z* isomers has to be known and is constant over the course of the whole measurement → no degradation.
2) Isomers are thermally stable to avoid change of isomer composition during ^{1}H NMR measurement and UV/Vis sample preparation.
3) Only two species in solution are present, i.e. the *E* and *Z* isomer of the respective photoswitch.

For each HPI derivative, samples of *E* and *Z* isomer mixtures with known total concentration (1.8×10^{-3} to 3.8×10^{-3} mol L^{-1}) were prepared in toluene-d_8 solution. Since the *Z* isomer of each HPI is energetically more stable than the *E* isomer, the prepared solutions were enriched with *Z* isomer (Z^{+}). The *Z* to *E* ratio was determined by integration of well-separated signals in the ^{1}H NMR spectra. Defined volumes of the NMR sample solutions were diluted in 2.5 mL spectroscopic toluene to obtain final concentrations between 1.2×10^{-5} and 6.8×10^{-5} mol L^{-1}. UV/Vis spectra were then recorded. Subsequently, the NMR sample solution was irradiated with LEDs of corresponding wavelengths to obtain a *E* isomer enriched solution (E^{+}). The same UV/Vis measurement procedure was perfomed as described above. To minimize dilution errors, several samples were prepared for the UV/Vis measurements or the spectra were scaled to the position of the isosbestic points, which was determined after irradation with light of a suitable wavelength.

In order to calculate the absorption spectrum for the pure *E* and *Z* isomers, a system of linear equation has to be solved ((10) and (11))

$$\mathrm{Abs}(E^{+}) = \mathrm{Abs}(E)e_1 + \mathrm{Abs}(Z)z_1 \tag{10}$$

With:
$\mathrm{Abs}(E^{+})$ = absorption of the recorded *E* isomer enriched solution
$\mathrm{Abs}(E)$ = absorption of pure *E* isomer
$\mathrm{Abs}(Z)$ = absorption of pure *Z* isomer
e_1= percentage of *E* isomer in the *E* enriched solution
z_1= percentage of *Z* isomer in the *E* enriched solution

$$\mathrm{Abs}(Z^+) = \mathrm{Abs}(E)e_2 + \mathrm{Abs}(Z)z_2 \tag{11}$$

With:

$\mathrm{Abs}(Z^+)$ = absorption of the recorded *Z* isomer enriched solution

$\mathrm{Abs}(E)$ = absorption of pure *E* isomer

$\mathrm{Abs}(Z)$ = absorption of pure *Z* isomer

e_2= percentage of *E* isomer in the *E* enriched solution

z_2= percentage of *Z* isomer in the *E* enriched solution

$\mathrm{Abs}(E^+)$ and $\mathrm{Abs}(Z^+)$ can be directly obtained from the recorded UV/Vis spectra of the corresponding enriched isomer mixtures while the percentages e_1, z_1, e_2 and z_2 are calculated by integration of the signals in the ^{1}H NMR spectra. Hence, the system of linear equations can be solved and the results are shown in (12) and (13).

$$\mathrm{Abs}(E) = \frac{\mathrm{Abs}(E^+)z_2 - \mathrm{Abs}(Z^+)z_1}{e_1 z_2 - z_1 e_2} \tag{12}$$

$$\mathrm{Abs}(Z) = \frac{\mathrm{Abs}(Z^+)e_1 - \mathrm{Abs}(E^+)e_2}{e_1 z_2 - z_1 e_2} \tag{13}$$

The molar absorption coefficents can be calculated using *Lambert-Beer* law ((14) and (15)).

$$\mathrm{Abs}(E) = \varepsilon_E [E] d \tag{14}$$

with:

ε_E = molar absorption coefficient of *E* isomer in L mol^{-1} cm^{-1}

$[E]$= concentration of *E* isomer in mol L^{-1}

d = path length of the cuvette in cm

$$\mathrm{Abs}(Z) = \varepsilon_Z [Z] d \tag{15}$$

with:

ε_Z = molar absorption coefficient of *Z* isomer in L mol^{-1} cm^{-1}

$[Z]$= concentration of *Z* isomer in mol L^{-1}

d = path length of the cuvette in cm

At the isosbestic points, the absorption of both *E* and *Z* isomers is the same. Since the sample concentration was constant during UV/Vis measurements, the calculated absorption spectra of pure *E* and *Z* isomers can be scaled to the mixed spectrum with known concentration ($[E + Z]$). This is expressed by (16) and (17):

$$\varepsilon_E = \frac{\mathrm{Abs}(E)d}{[E + Z]} \tag{16}$$

$$\varepsilon_Z = \frac{\mathrm{Abs}(Z)d}{[E + Z]} \tag{17}$$

1.1.26 Determination of molar absorption coefficients of planar HPIs

For the planar HPIs derivatives **8-14**, the synthesis yielded exclusively the thermally more stable *Z* isomers. Hence, Abs(*Z*) can be directly measured and from an *E* enriched NMR sample with known isomer concentration and the corresponding UV/Vis spectrum, Abs(*E*) can be calculated using equation (10) and the molar absorption coefficients can then be calculated using *Lambert-Beer* law (equation (14) and (15)).

For validation of the method, the *E* isomer for HPI **1** was isolated (see section 1.2) and its molar absorption coefficients were determined directly (Supplementary Figure 28). The calculated molar absorption compare well to the directly determined ones, showing the same band shape and position of the isosbestic points. Differences in value can be attributed to differences and errors in sample preparation.

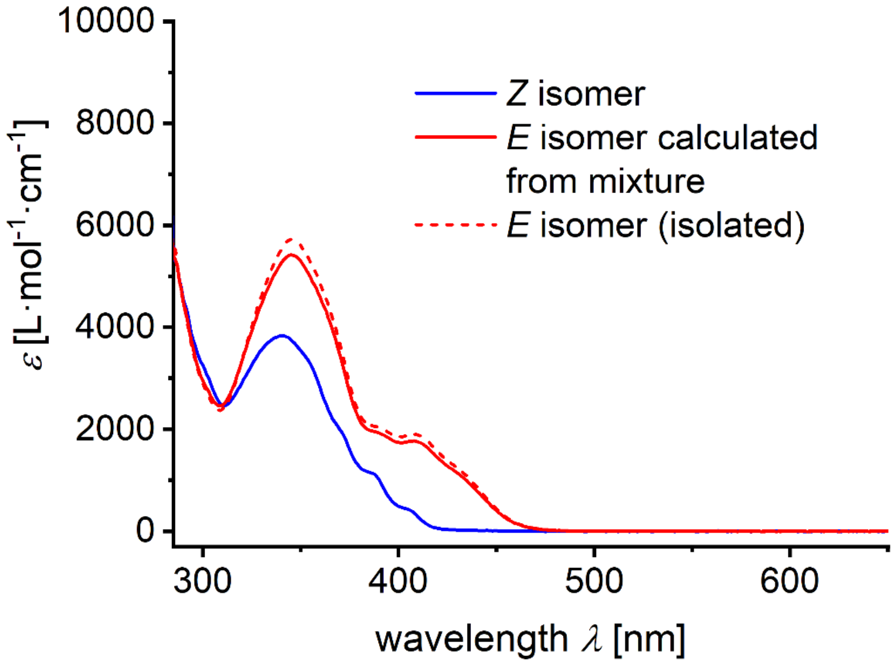

Supplementary Figure 28. Experimentally determined molar absorption coefficients for the pure *Z* (blue) and *E* (red) isomers of HPI **1** in toluene at 23 °C. For comparison, the directly molar absorption coefficients for the *E* isomer after isolation are shown (dotted red).

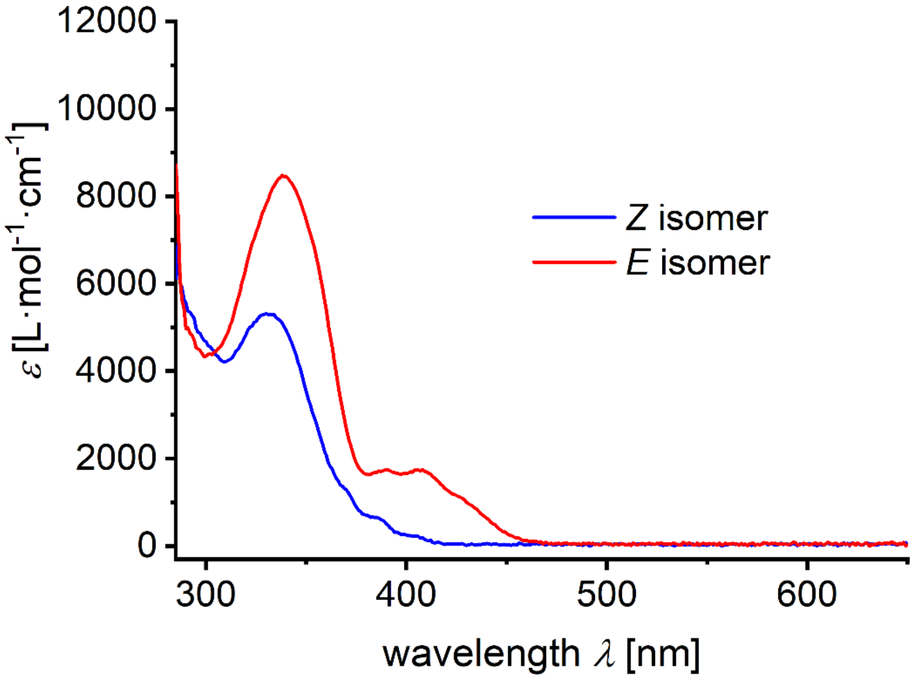

Supplementary Figure 29. Experimentally determined molar absorption coefficients for the pure *Z* (blue) and *E* (red) isomers of HPI **2** in toluene at 23 °C.

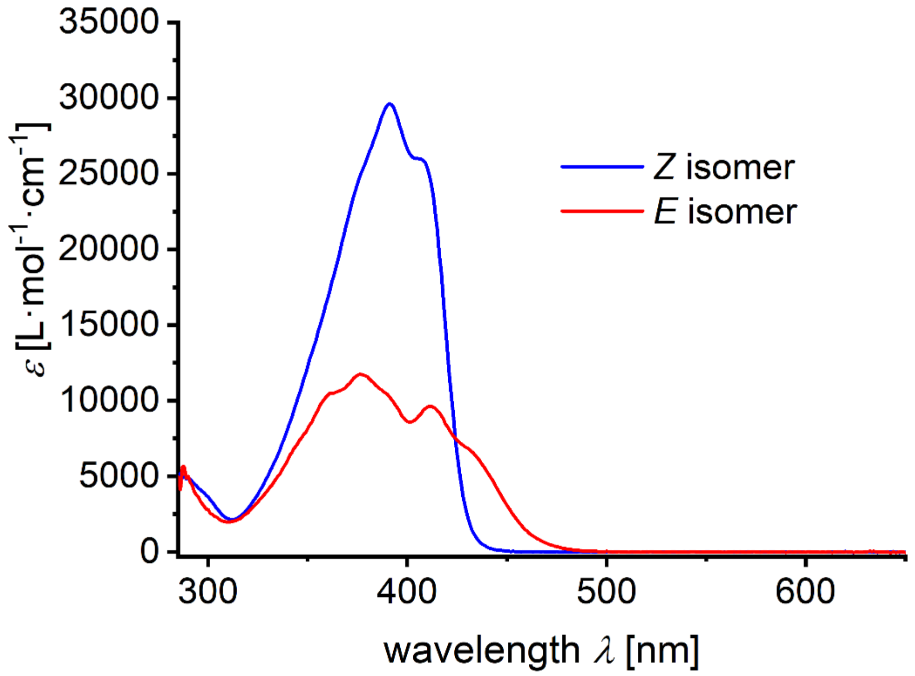

Supplementary Figure 30. Experimentally determined molar absorption coefficients for the pure *Z* (blue) and *E* (red) isomers of HPI **3** in toluene at 23 °C.

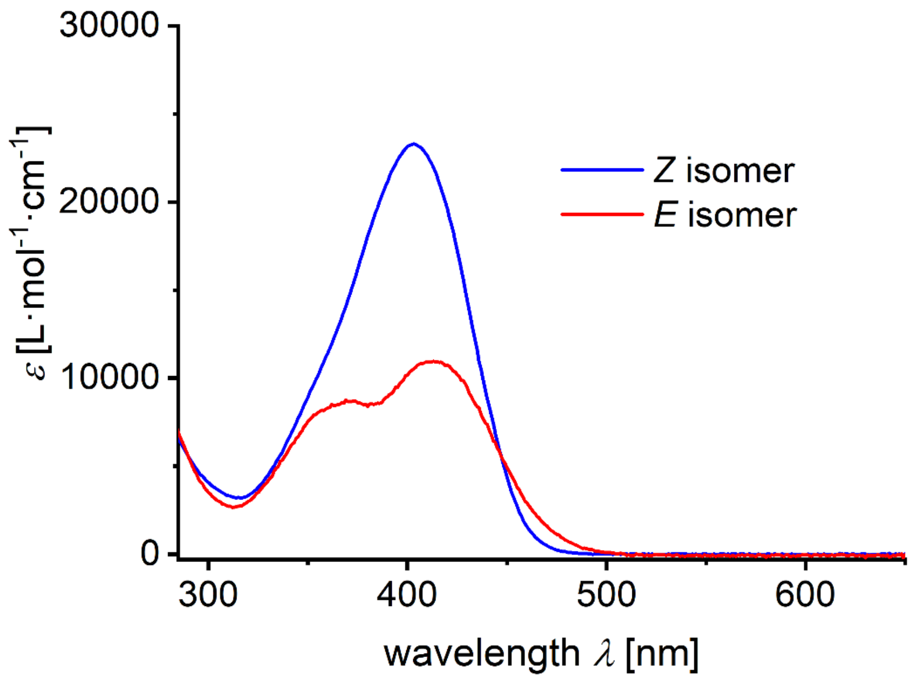

Supplementary Figure 31. Experimentally determined molar absorption coefficients for the pure *Z* (blue) and *E* (red) isomers of HPI **3-OH** in water at 23 °C.

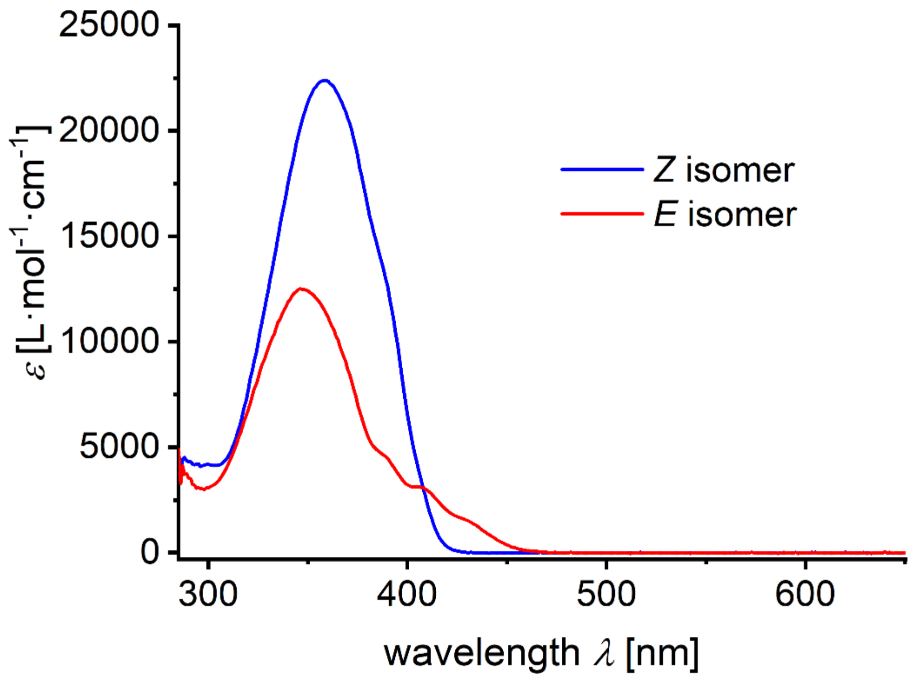

Supplementary Figure 32. Experimentally determined molar absorption coefficients for the pure *Z* (blue) and *E* (red) isomers of HPI **4** in toluene at 23 °C.

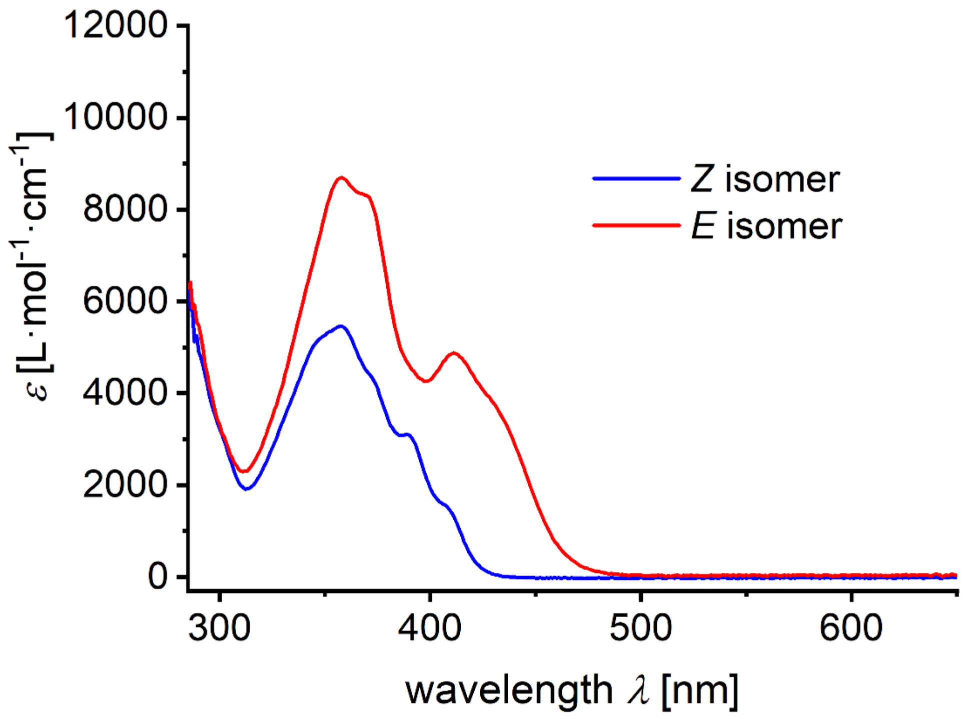

Supplementary Figure 33. Experimentally determined molar absorption coefficients for the pure *Z* (blue) and *E* (red) isomers of HPI **5** in toluene at 23 °C.

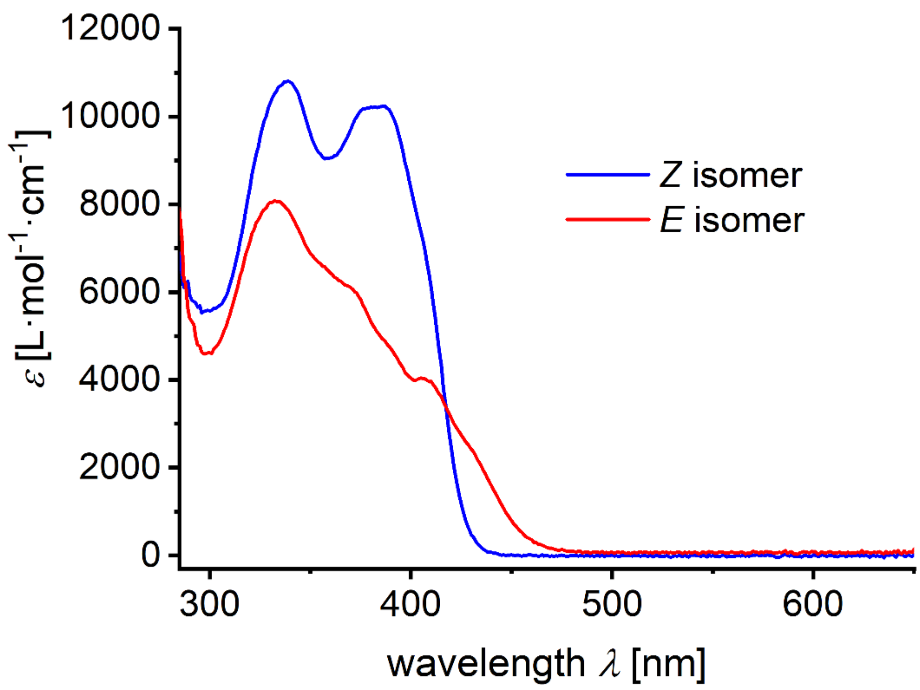

Supplementary Figure 34. Experimentally determined molar absorption coefficients for the pure *Z* (blue) and *E* (red) isomers of HPI **6** in toluene at 23 °C.

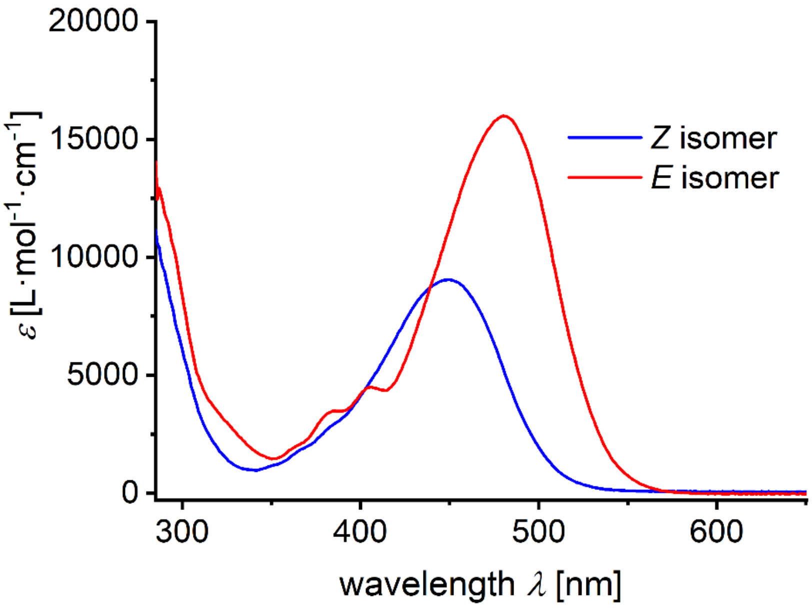

Supplementary Figure 35. Experimentally determined molar absorption coefficients for the pure *Z* (blue) and *E* (red) isomers of HPI **7** in toluene at 23 °C.

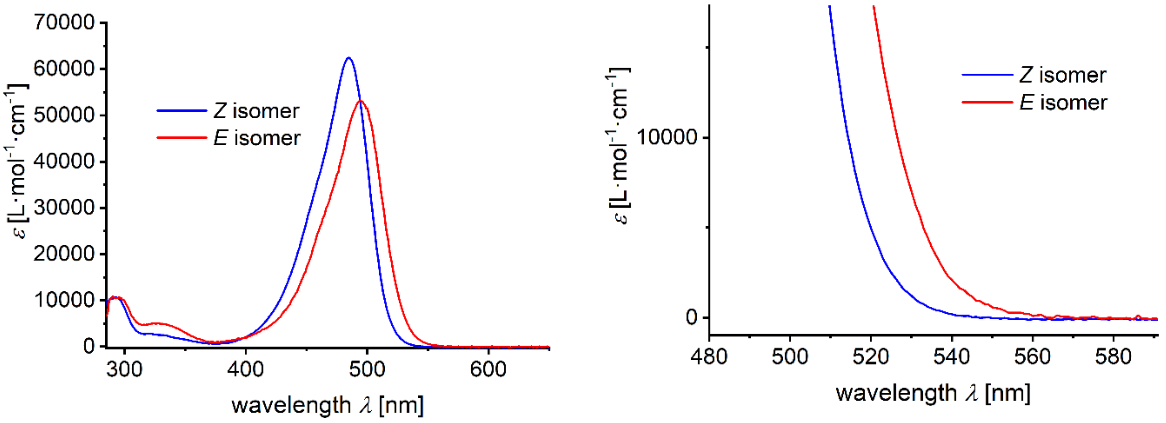

Supplementary Figure 36. (left) Experimentally determined molar absorption coefficients for the pure *Z* (blue) and *E* (red) isomers of HPI **8** in toluene at 23 °C. (right) Enlarged region of the same spectrum.

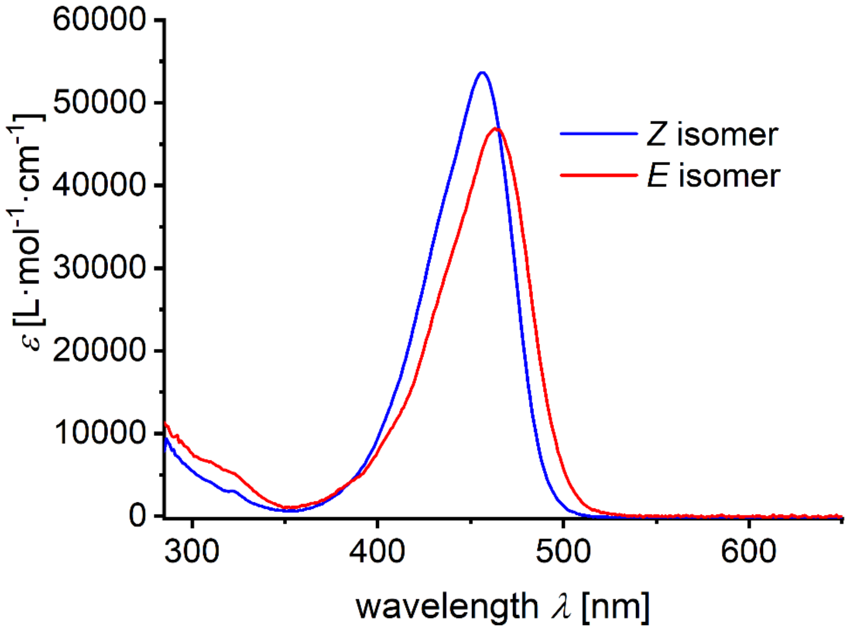

Supplementary Figure 37. Experimentally determined molar absorption coefficients for the pure *Z* (blue) and *E* (red) isomers of HPI **9** in toluene at 23 °C.

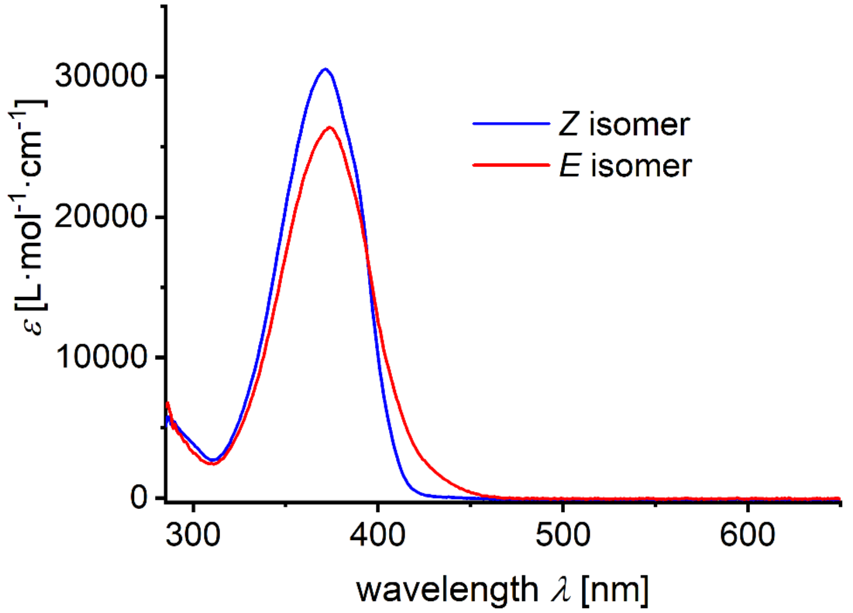

Supplementary Figure 38. Experimentally determined molar absorption coefficients for the pure *Z* (blue) and *E* (red) isomers of HPI **10** in toluene at 23 °C.

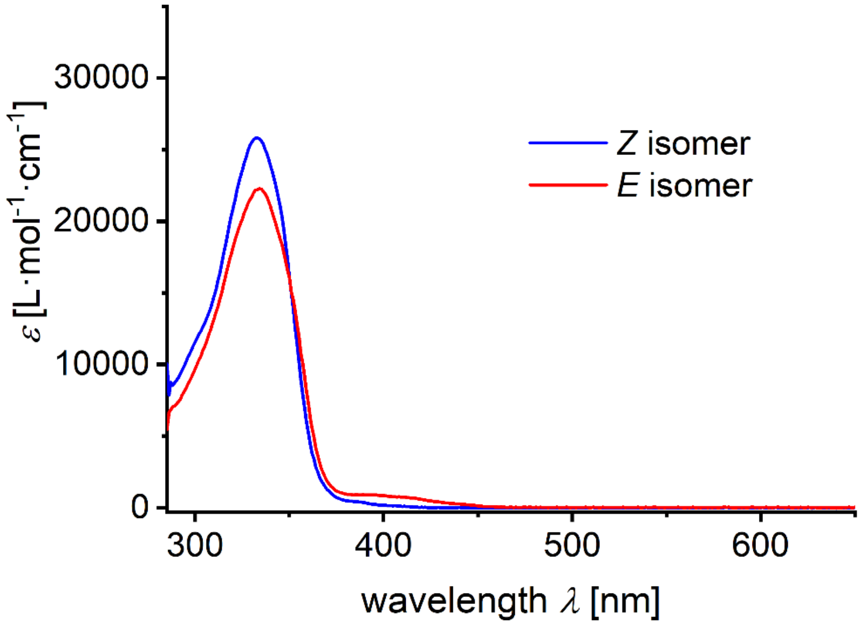

Supplementary Figure 39. Experimentally determined molar absorption coefficients for the pure *Z* (blue) and *E* (red) isomers of HPI **11** in toluene at 23 °C.

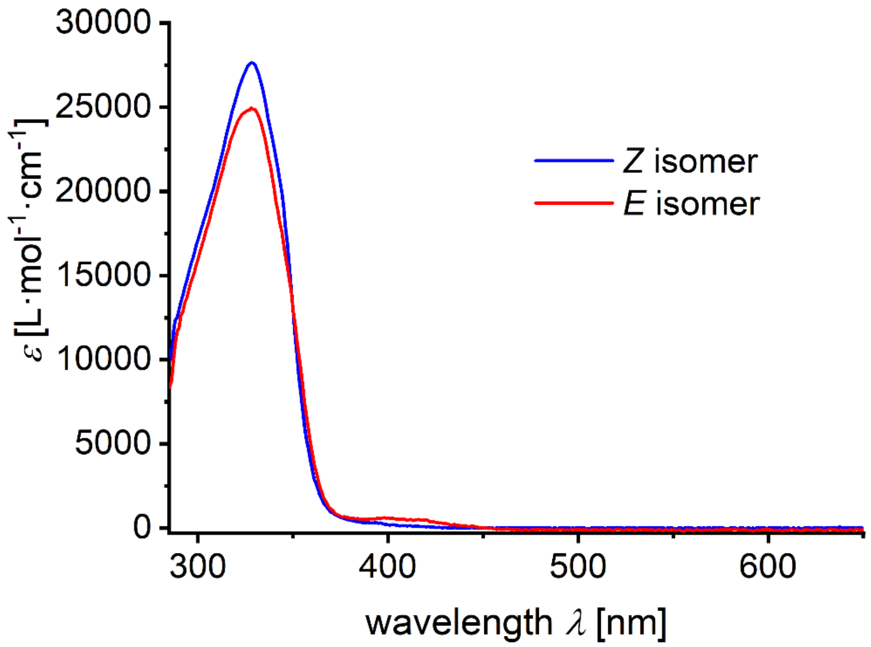

Supplementary Figure 40. Experimentally determined molar absorption coefficients for the pure *Z* (blue) and *E* (red) isomers of HPI **12** in toluene at 23 °C.

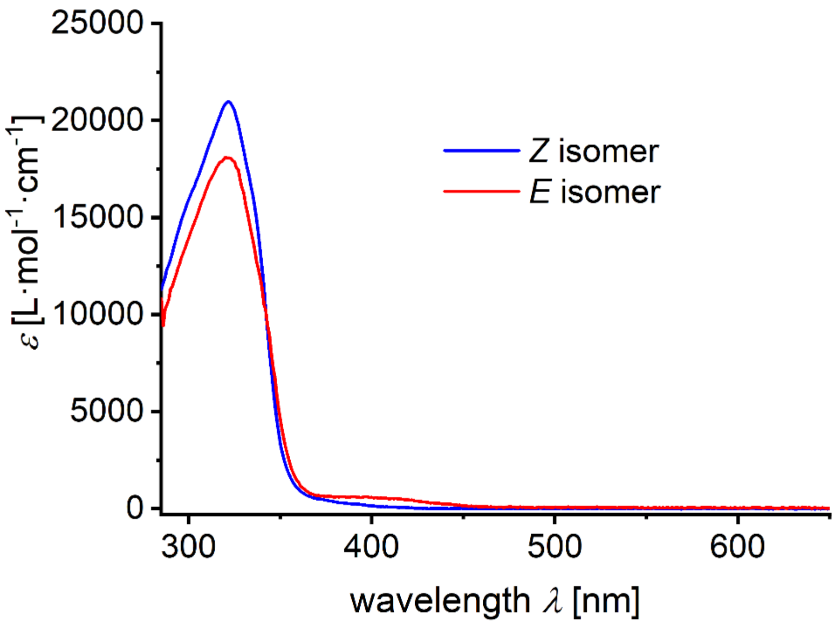

Supplementary Figure 41. Experimentally determined molar absorption coefficients for the pure *Z* (blue) and *E* (red) isomers of HPI **13** in toluene at 23 °C.

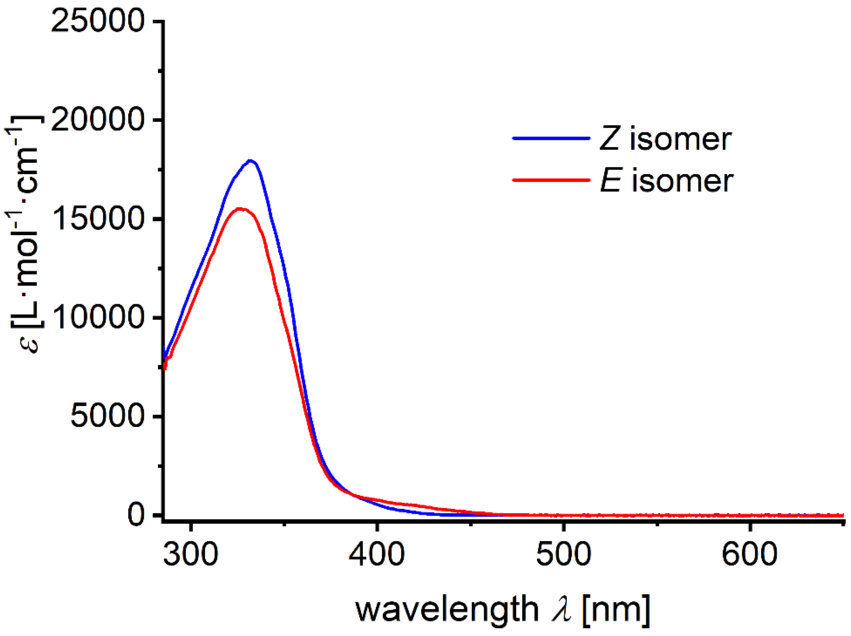

Supplementary Figure 42. Experimentally determined molar absorption coefficients for the pure *Z* (blue) and *E* (red) isomers of HPI **14** in toluene at 23 °C.

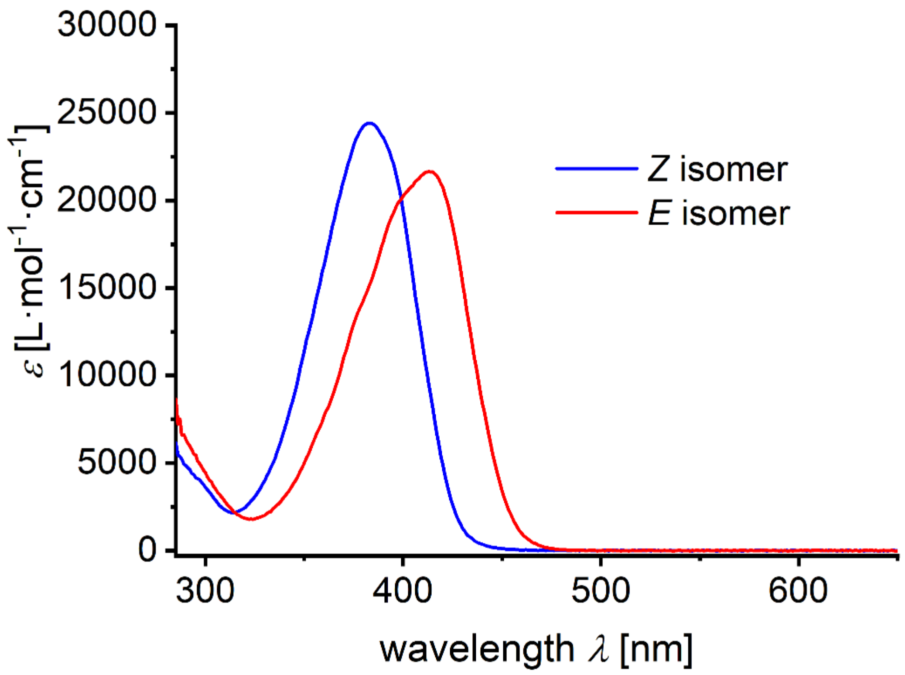

Supplementary Figure 43. Experimentally determined molar absorption coefficients for the pure *Z* (blue) and *E* (red) isomers of HPI **15** in toluene at 23 °C.

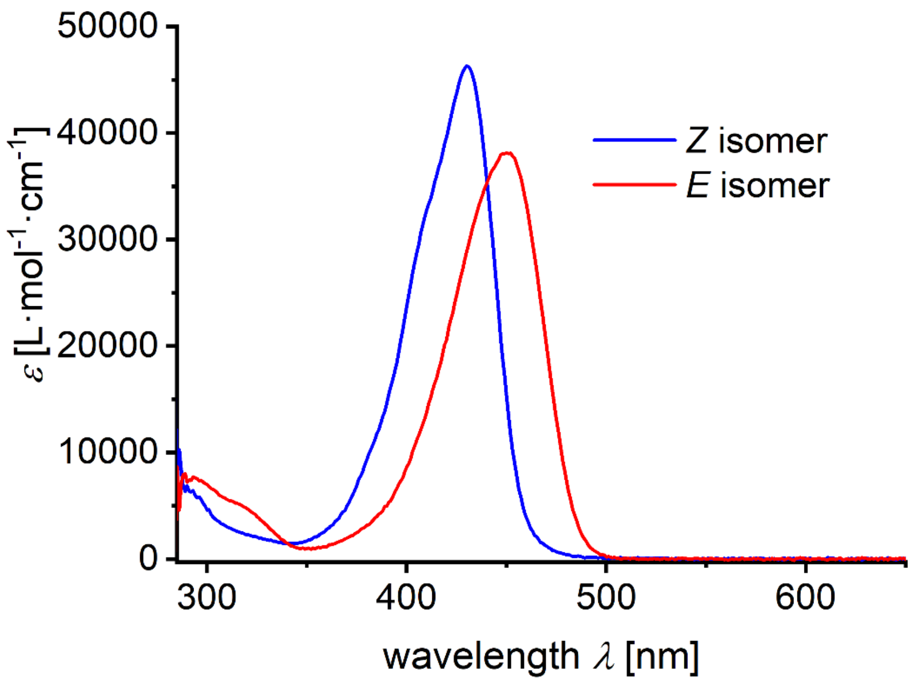

Supplementary Figure 44. Experimentally determined molar absorption coefficients for the pure *Z* (blue) and *E* (red) isomers of HPI **16** in toluene at 23 °C.

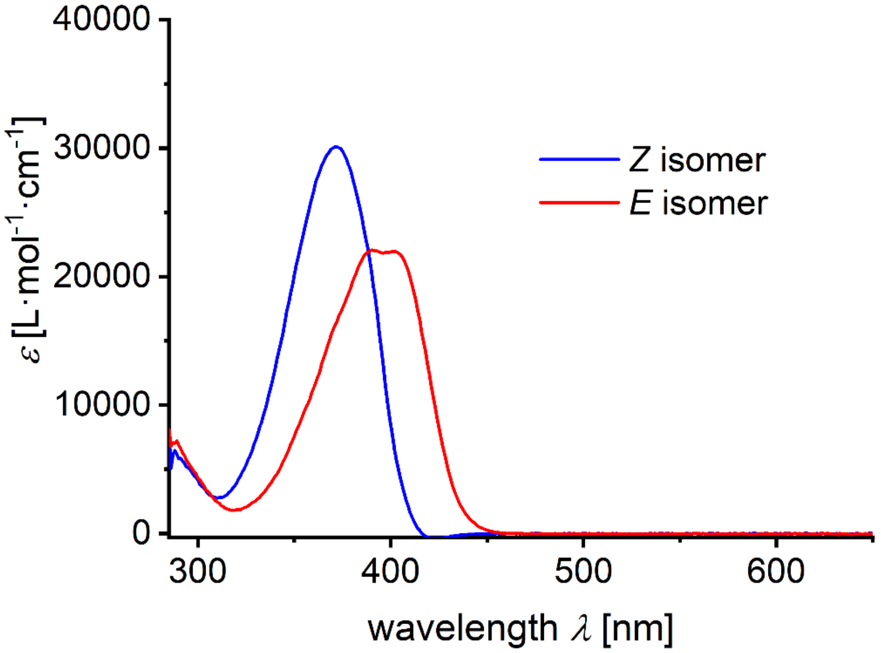

Supplementary Figure 45. Experimentally determined molar absorption coefficients for the pure *Z* (blue) and *E* (red) isomers of HPI **17** in toluene at 23 °C.

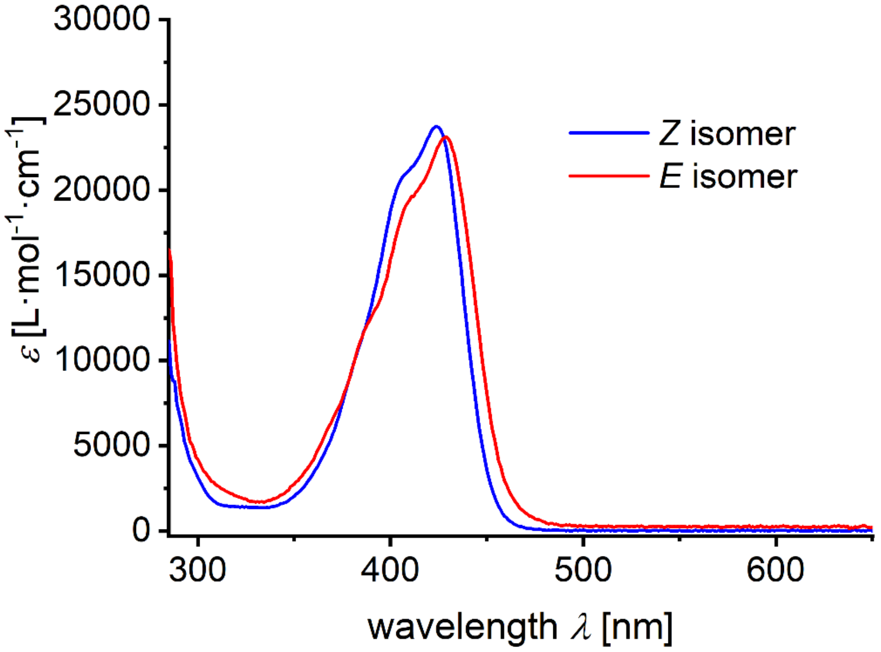

Supplementary Figure 46. Experimentally determined molar absorption coefficients for the pure *Z* (blue) and *E* (red) isomers of HPI **18** in toluene at 23 °C.

1.1.27 Comparison of HPI, HI and HTI

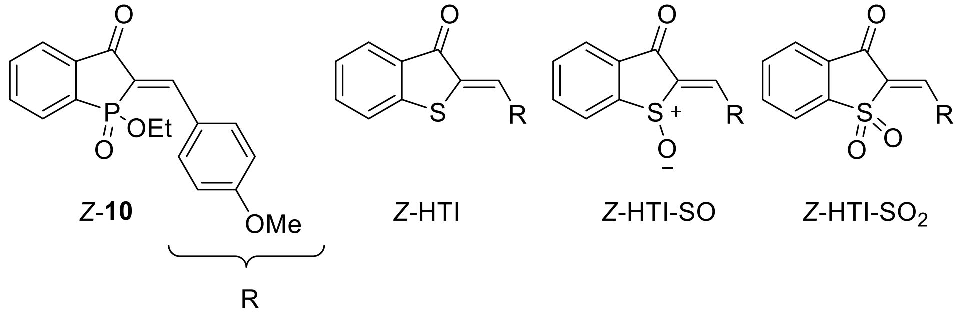

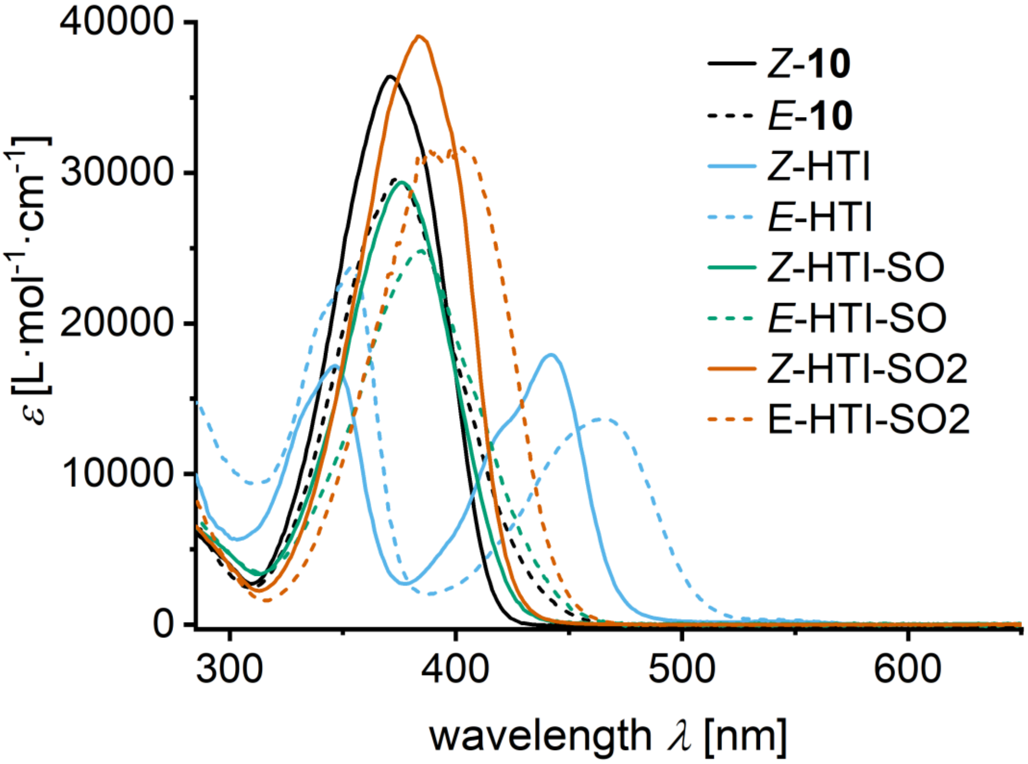

Supplementary Figure 47. Comparison of the molar absorption coefficients of different indigoid photoswitches in dichloromethane solution. (top) Molecular structures (bottom) Experimentally determined molar absorption coefficients in CH_2Cl_2 solution. Spectral data for the different indigoid photoswitches except HPI were published in: [8].

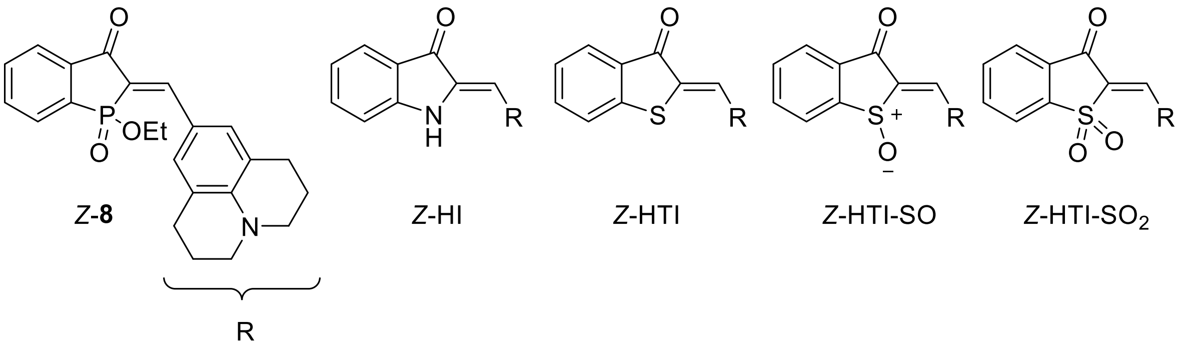

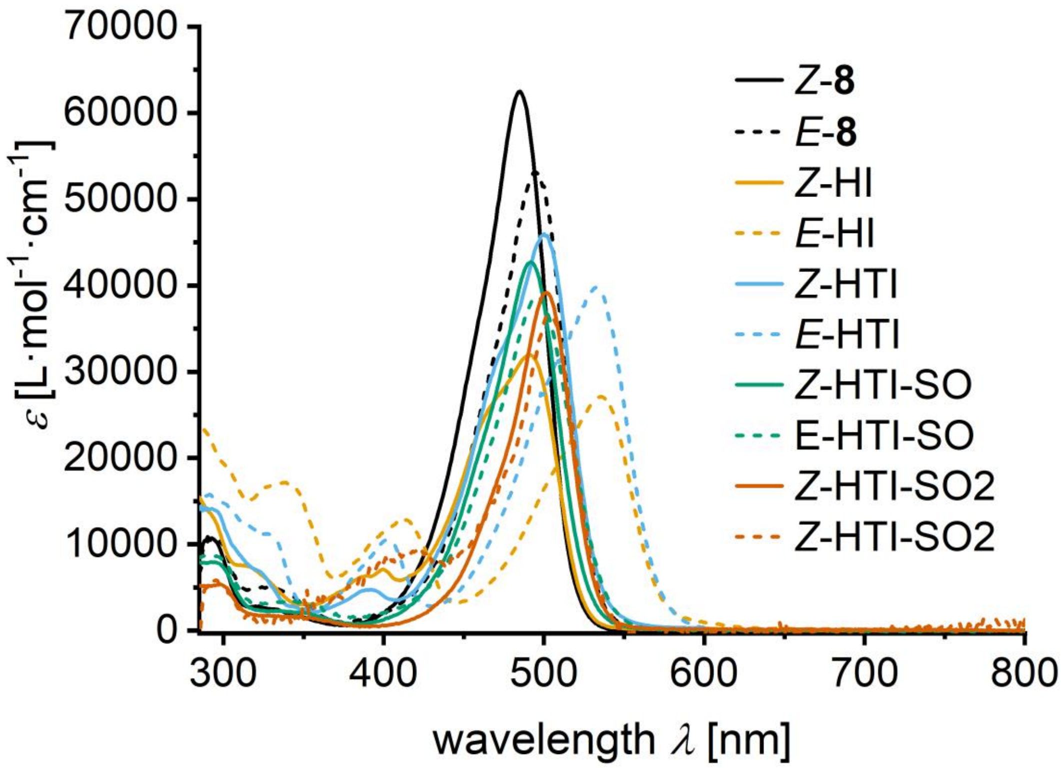

Supplementary Figure 48. Comparison of the molar absorption coefficients of different indigoid photoswitches in toluene solution. (top) Molecular structures (bottom) Experimentally determined molar absorption coefficients in toluene. Spectral data for the different indigoid photoswitches except HPI were published in: [8-9].

Supplementary Note 6: ECD spectra of the pure *Z* isomers and after light irradiation

The concentration of samples for ECD measurements was adjusted until the absorption of the highest absorption band was between 0.8 and 1.0 (absorbance for optimal signal to noise ratio: 0.89). For better comparability, the concentration independent g factors were calculated: g factor= θ/ Abs.

In order to convert the ellipticity in mdeg to Abs, the ellipiticy values were divided by 32980 (conversion factor).

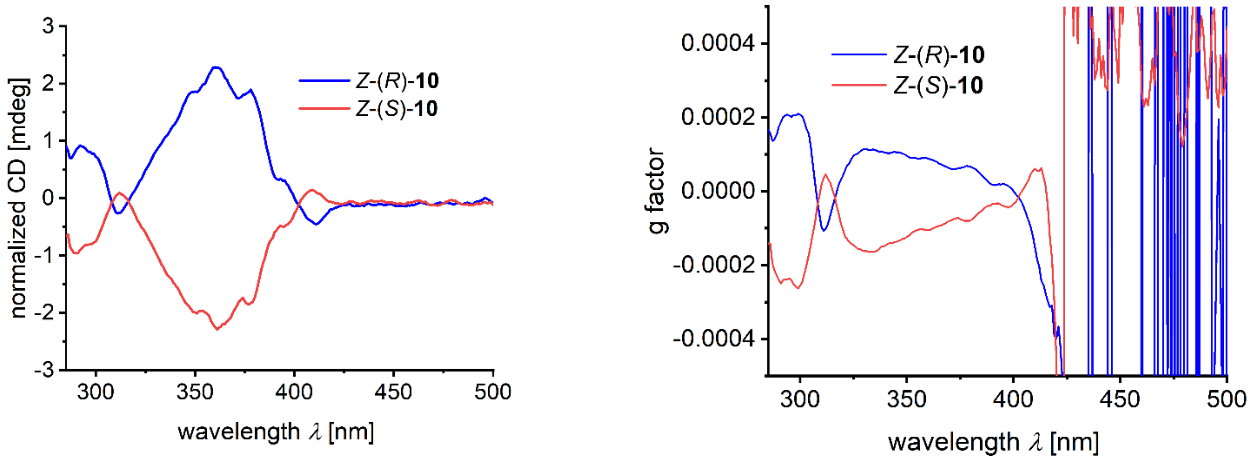

Supplementary Figure 49. Experimental ECD spectrum of planar HPI *Z*-**10** in toluene at 24 °C. (left) ECD spectrum of HPI *Z*-**10**. Enantiomer assignment was performed using the enantiomeric pure crystal structures of the corresponding HPLC fraction. (right) Calculated g factors.

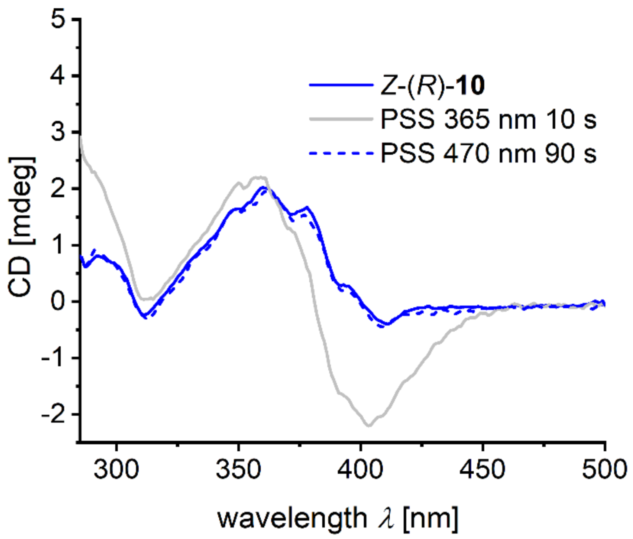

Supplementary Figure 50. Experimental ECD spectrum of planar HPI *Z*-(*R*)-**10** in toluene at 24 °C before and after irradiation with 365 nm (*Z* to *E*) and 470 nm (*E* to *Z*) light.

Note: For HPI **10**, enantiomeric pure crystals could be obtained. For the corresponding crystals structures see Supplementary Note 16 “Crystal structure analysis” below.

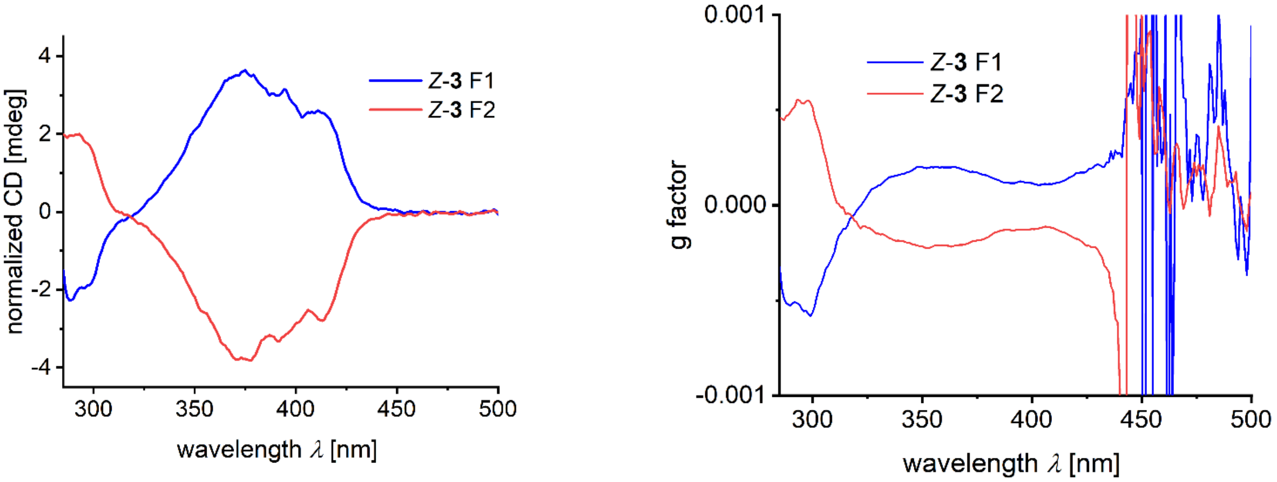

Supplementary Figure 51. Experimental ECD spectrum of twisted HPI *Z*-**3** in toluene at 24 °C. (left) ECD spectrum of HPI *Z*-**3**. Enantiomers are numbered according to the elution order during HPLC. (right) Calculated g factors.

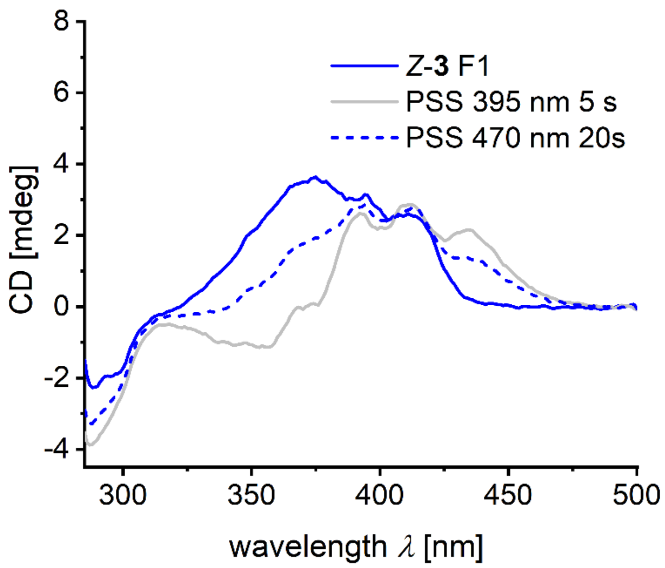

Supplementary Figure 52. Experimental ECD spectrum of twisted HPI *Z*-**3** F1 in toluene at 24 °C before and after irradiation with 395 nm (*Z* to *E*) and 470 nm (*E* to *Z*) light.

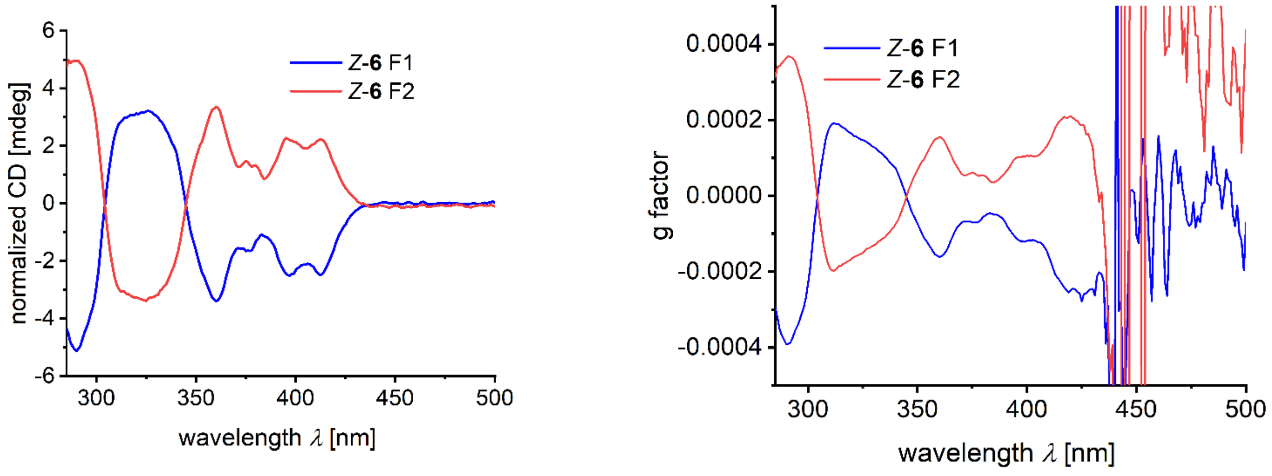

Supplementary Figure 53. Experimental ECD spectrum of twisted HPI *Z*-**6** in toluene at 24 °C. (left) ECD spectrum of HPI *Z*-**6**. Enantiomers are numbered according to the elution order during HPLC. (right) Calculated g factors.

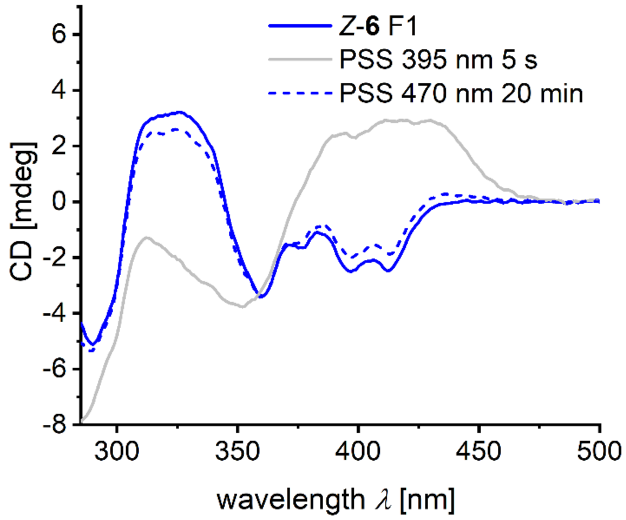

Supplementary Figure 54. Experimental ECD spectrum of twisted HPI *Z*-**6** F1 in toluene at 24 °C before and after irradiation with 395 nm (*Z* to *E*) and 470 nm (*E* to *Z*) light.

Supplementary Note 7: Theoretical studies of HPI 3, 6 and 10

1.1.28 Ground state energies

All calculations for HPIs **3**, **6** and **10** were carried out using the Gaussian16 Revision C.03 program package.[10] Conformational searches for all derivatives were performed using the MacroModel[11] software package from the Schroedinger suite.
A mixed torsional/low-mode sampling was carried out for both the *Z* and *E* isomers with a sampling threshold of 40 kJ/mol. A static field with a dielectric constant of ε = 2.4 (corresponding to the dielectric constant of toluene)[10] was chosen as continuum solvation. The obtained 10 structures for each isomer with the lowest energies were then further DFT-optimized at different levels of theory (specified below) and dispersion correction. Geometrically and energetically identic conformers were sorted out and a frequency analysis was performed to check if the found structures were minima (no imaginary frequencies present).

To determine the calculated relative ground state energies of the corresponding *Z* and *E* isomers, two methods were used: The first being simple subtraction of the lowest energy conformers for both *Z* and *E* isomers, while the second included Boltzmann weighting. Since the relative energies of the conformers were low (between 0 and 2 kcal/mol) the latter method allows for better comparability with the experiment. For this, a Boltzmann weighting factor was calculated for each conformer of one isomer at 100 °C (measurement temperature of all experiments with these derivatives) and the energies of each conformer were then multiplied with the corresponding factor and added up to the overall energy of the isomers' conformational space. Subsequent subtraction of the energies of both conformational spaces (*Z* and *E*) resulted in the relative ground state energies. All values are summarized in the table below.

Supplementary Table 3. Experimental and calculated Δ*G* values of the stable *Z* isomer and the metastable *E* isomer of twisted HPIs **3** and **6** and planar HPI **10**. All calculations were performed using a PCM solvation model with toluene as solvent. Boltzmann weighting was conducted with *T*=100 °C, since the experimental Δ*G* values were determined at that temperature.

HPI	Level of theory	Dispersion	ΔG_{exp} [kcal/mol	$\Delta G_{lowest\ conformers}$ [kcal/mol]	$\Delta G_{Boltzmann}$ [kcal/mol]
**3**	CAM-B3LYP/ 6-311+ G(d,p)	gd3bj	2.4	3.50	2.98
**3**	PBE0/ 6-311++ G(d,p)	gd3bj	2.4	3.58	3.38
**3**	M06/ 6-311++ G(d,p)	gd3	2.4	4.27	4.07
**3**	PW6B95D3/ 6-311++ G(d,p)	included in functional	2.4	3.03	2.81
**6**	CAM-B3LYP/ 6-311+ G(d,p)	gd3bj	1.6	2.88	2.88
**6**	PBE0/ 6-311++ G(d,p)	gd3bj	1.6	2.50	2.80
**6**	M06/ 6-311++ G(d,p)	gd3	1.6	4.09	4.11
**6**	PW6B95D3/ 6-311++ G(d,p)	included in functional	1.6	1.72	2.11
**10**	CAM-B3LYP/ 6-311+ G(d,p)	gd3bj	3.1	3.94	3.88
**10**	PBE0/ 6-311++ G(d,p)	gd3bj	3.1	3.58	3.99
**10**	M06/ 6-311++ G(d,p)	gd3	3.1	4.32	3.94
**10**	PW6B95D3/ 6-311++ G(d,p)	included in functional	3.1	4.22	3.93

1.1.29 UV and ECD spectra calculations

For the calculation of the UV and ECD spectra, a time-dependent density functional theory (TDDFT) based calculation was performed. For a first functional screening, the optimized geometries of the conformers originating from the CAM-B3LYP/6-311+G(d, p) calculations were used and the following functionals were applied for the TDDFT calculation (30 states): PBE0, M06; PW6B95D3, CAM-B3LYP, B3LYP and wB97XD. The screening was performed for HPI **3**, **6**, and **10**. Analysis of the UV/Vis absorption spectra and the ECD spectra was performed using the SpecDis Software Version 1.71[12] and the spectra of different conformers were summed up according to their corresponding Boltzmann weighting factor (at T= 298 K). The results for the screening for all three HPIs are plotted with an exponential half-width σ=0.29 eV and no UV shift correction and compared with the experimental data (see below). Since toluene is absorbing in the UV region, the spectra are only plotted for wavelengths >285 nm.

1.1.30 TDDFT Functional Screening

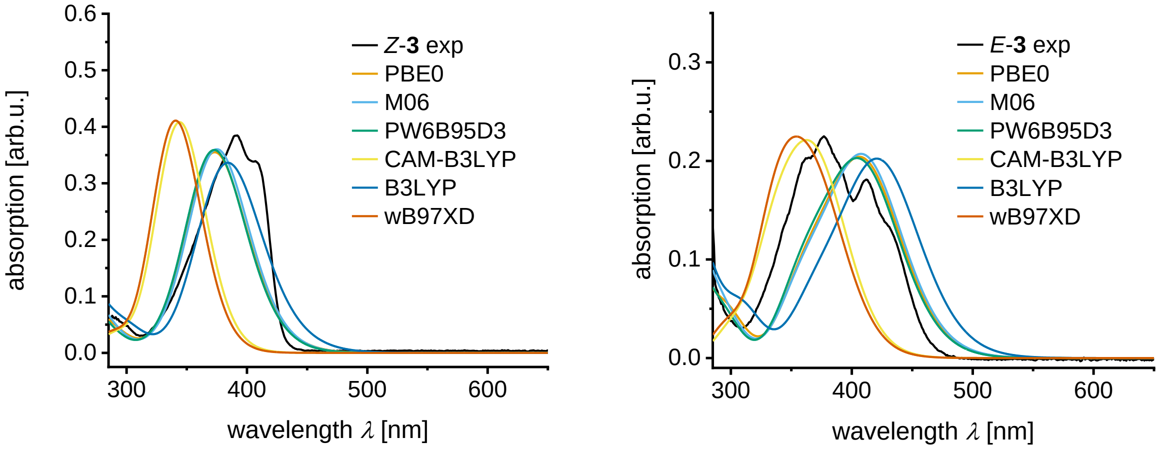

Supplementary Figure 55. Screening of UV spectra for *Z*-**3** (left) and *E*-**3** (right), calculated with the indicated functional and 6-311+G(d,p) as basis set, using a PCM solvent model with toluene as solvent. All TDDFT calculations are based on the optimized structures obtained from CAM-B3LYP/6-311+G(d,p) level of theory. The UV spectra are plotted with an exponential half-width of σ=0.29 eV and no UV shift correction.

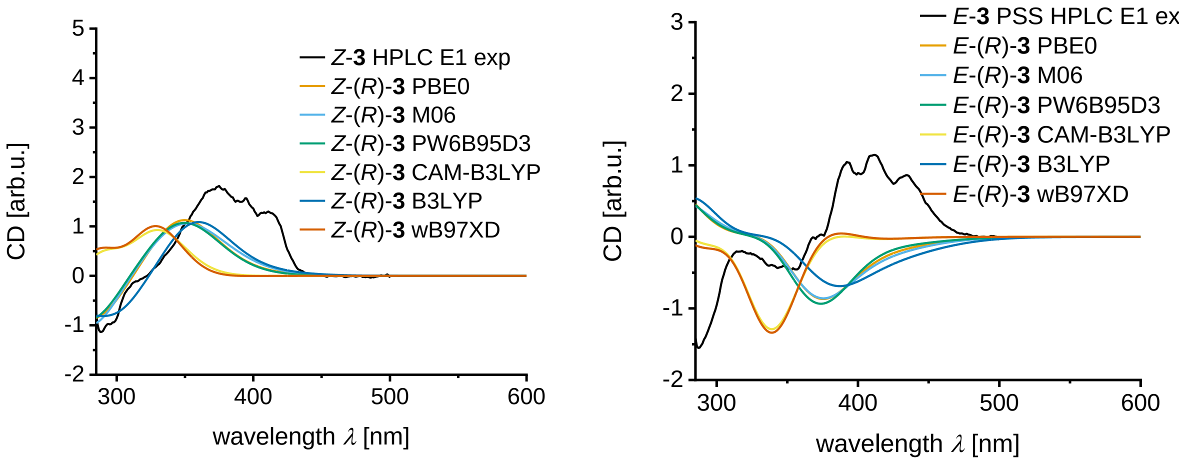

Supplementary Figure 56. Screening of ECD spectra for *Z*-**3** (left) and *E*-**3** (right), calculated with the indicated functional and 6-311+G(d,p) as basis set, using a PCM solvent model with toluene as solvent. All TDDFT calculations are based on the optimized structures obtained from CAM-B3LYP/6-311+G(d,p) level of theory. The ECD spectra are plotted with an exponential half-width of σ=0.29 eV and no ECD shift correction.

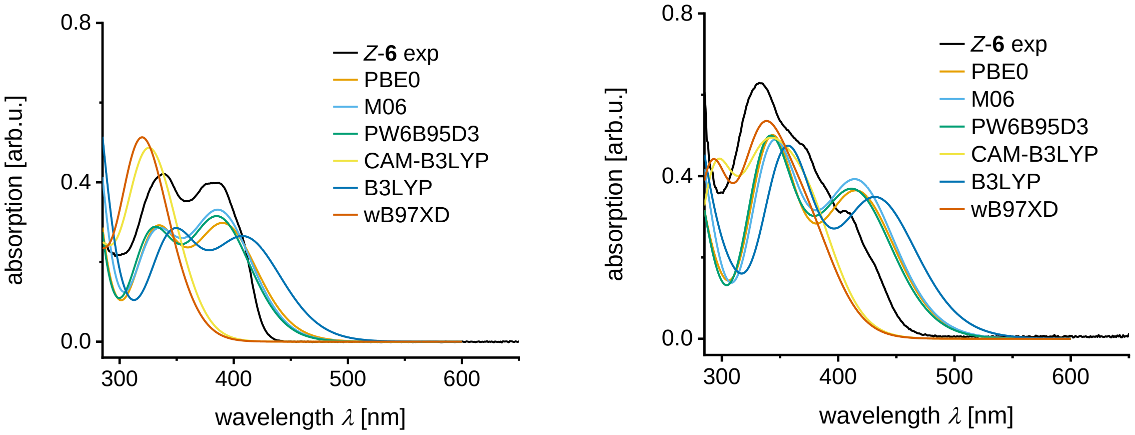

Supplementary Figure 57. Screening of UV spectra for *Z*-**6** (left) and *E*-**6** (right), calculated with the indicated functional and 6-311+G(d,p) as basis set, using a PCM solvent model with toluene as solvent. All TDDFT calculations are based on the optimized structures obtained from CAM-B3LYP/6-311+G(d,p) level of theory. The UV spectra are plotted with an exponential half-width of σ=0.29 eV and no UV shift correction.

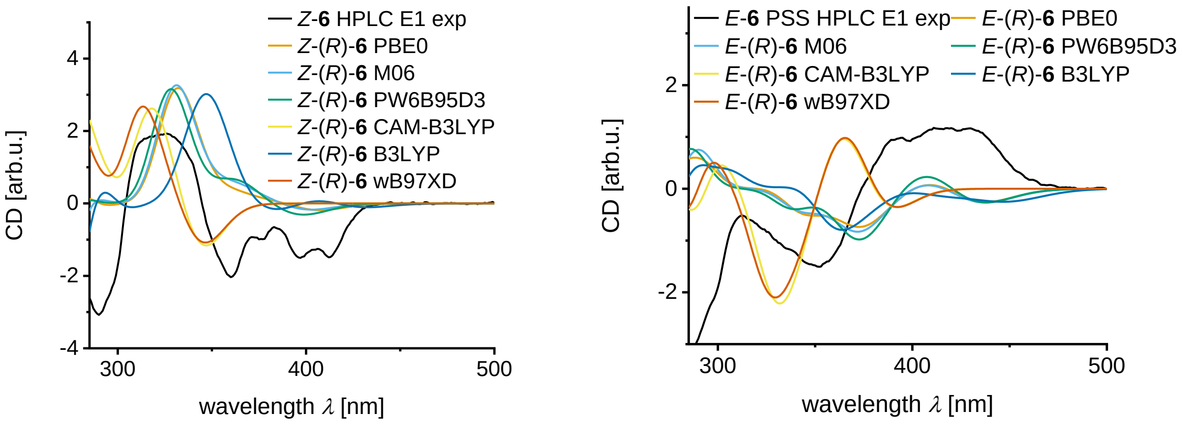

Supplementary Figure 58. Screening of ECD spectra for *Z*-**6** (left) and *E*-**6** (right), calculated with the indicated functional and 6-311+G(d,p) as basis set, using a PCM solvent model with toluene as solvent. All TDDFT calculations are based on the optimized structures obtained from CAM-B3LYP/6-311+G(d,p) level of theory. The ECD spectra are plotted with an exponential half-width of σ=0.29 eV and no ECD shift correction.

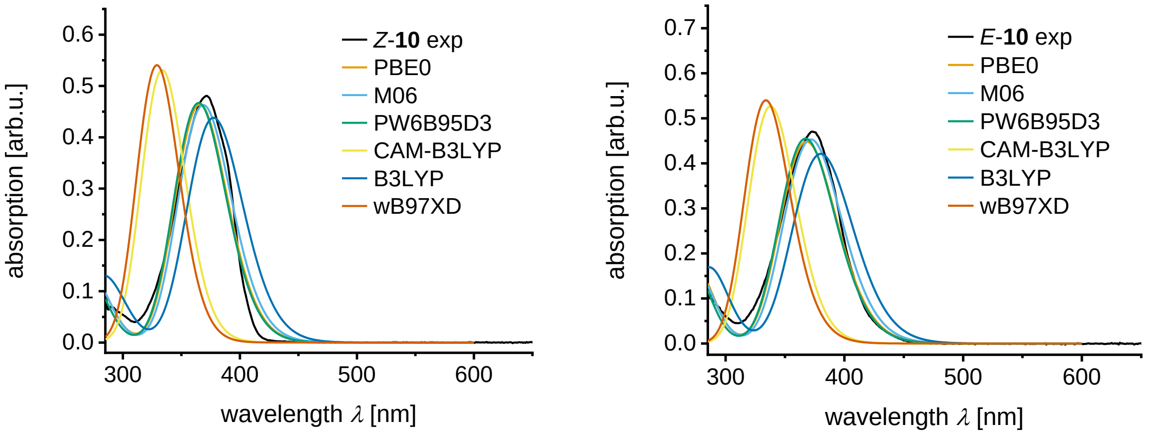

Supplementary Figure 59. Screening of UV spectra for *Z*-**10** (left) and *E*-**10** (right), calculated with the indicated functional and 6-311+G(d,p) as basis set, using a PCM solvent model with toluene as solvent. All TDDFT calculations are based on the optimized structures obtained from CAM-B3LYP/6-311+G(d,p) level of theory. The UV spectra are plotted with an exponential half-width of σ=0.29 eV and no UV shift correction.

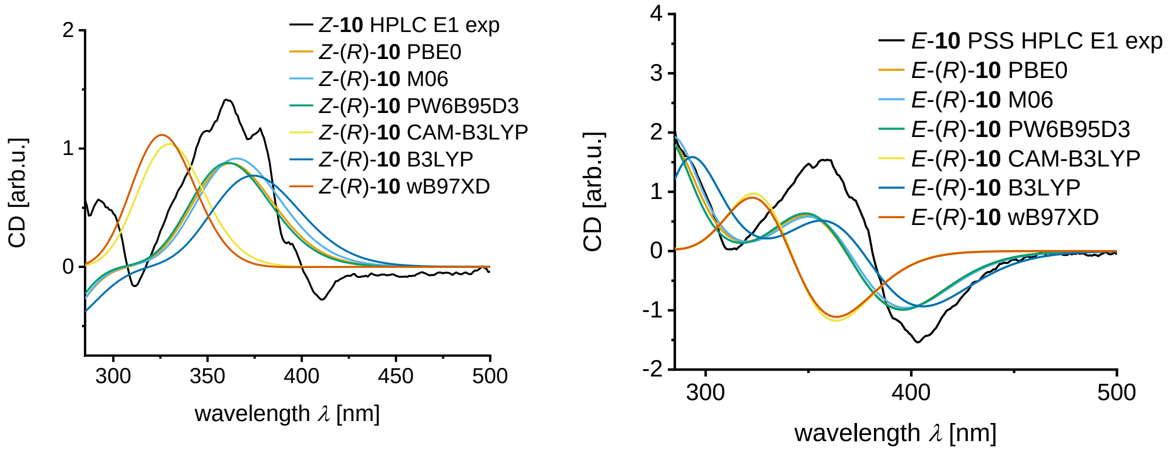

Supplementary Figure 60. Screening of ECD spectra for *Z*-**10** (left) and *E*-**10** (right), calculated with the indicated functional and 6-311+G(d,p) as basis set, using a PCM solvent model with toluene as solvent. All TDDFT calculations are based on the optimized structures obtained from CAM-B3LYP/6-311+G(d,p) level of theory. The ECD spectra are plotted with an exponential half-width of σ=0.29 eV and no ECD shift correction.

From the functional screening two conclusions can be drawn: First, CAM-B3LYP and wB97XD give similar strongly hypsochromically shifted UV and ECD spectra with differences of >40 nm for all three derivatives while the B3LYP functional overestimates the bathochromic shift with >20 nm compared to the corresponding experimental values. Spectra calculated with PBE0, M06 and PW6B95D3 give very similar results and are in best agreement (in shift and curve shape) with the experimental values for the three HPIs investigated. Second, for the twisted derivatives **3** and **6**, all functionals have more trouble reproducing the experimental data, compared to the planar HPI **10**, which is evident when looking at the corresponding calculated ECD spectra for the *E*-isomers. The reason for this is, that the orientation of the substituted stilbene part relative to the HPI core has a significant influence on the UV and ECD

spectra in shift of the spectrum and also the sign of the ECD spectra of the *E* isomers. Since the stilbene part can rotate freely around the single bond connected to the central double bond, a number of different conformers are contributing, which are very close in energy. This problem is further illustrated in Supplementary Table 4 and Supplementary Table 5 where optimization and TDDFT calculations were performed on a PBE0/6-311++G(d,p) level of theory for HPI **3**. The energy range for the conformers of the *Z* isomer is 0-2.53 kcal/mol while the energy range for the conformers of the *E*-isomer is even smaller with 0-1.51 kcal/mol. While the ring orientation of the *Z*-isomer conformers mainly affects shift and band broadening of the ECD spectra, the impact is much stronger with the conformers of the *E*-isomer. This becomes clear in the case of conformer 6 and 7 which possess the same energy but display complete sign inversion in the calculated ECD spectra due to a different ring orientation. Consequently, errors in energy of the conformers have a strong impact on the final Boltzmann weighted calculated spectra. Thus, obtaining calculated spectra which are in good agreement with the experimental values is difficult since the energies of the conformers lie in the typical error range for DFT calculations.

Supplementary Table 4. Conformational analysis of HPI Z-(*R*)-**3** with corresponding optimized molecular geometries and calculated ECD spectra on a PBE0/6-311++G(d,p) level of theory.

HPI *Z*-(*R*)-**3**	Relative energy [kcal/mol]	Optimized Geometry	Calculated ECD spectrum
Conf 1	1.81	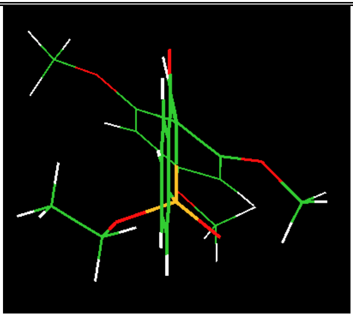	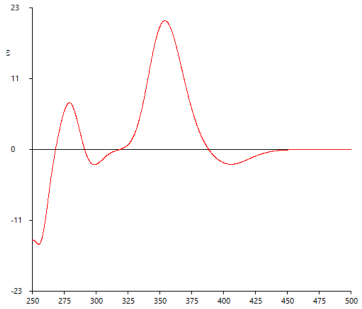
Conf 2	0.45	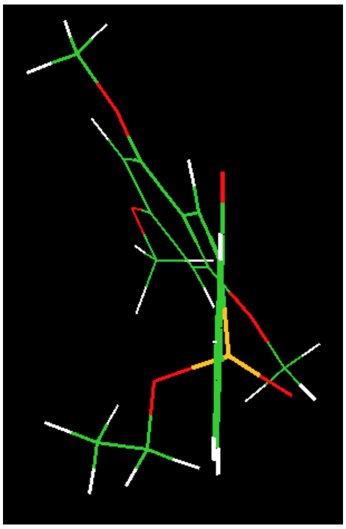	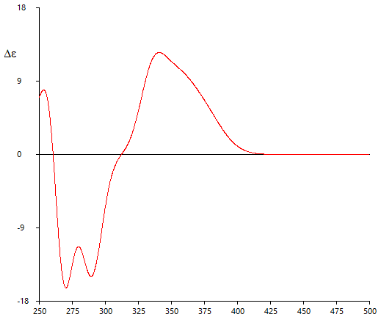

Conf 4 0.64

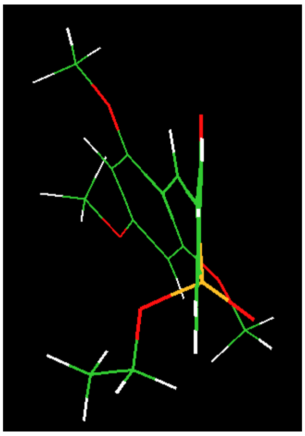

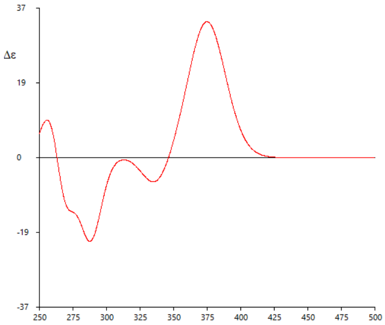

Conf 5 2.53

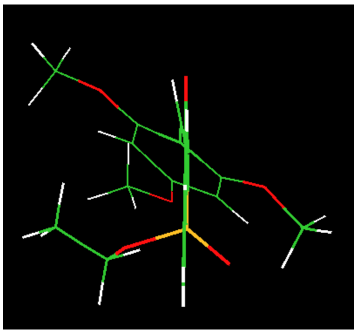

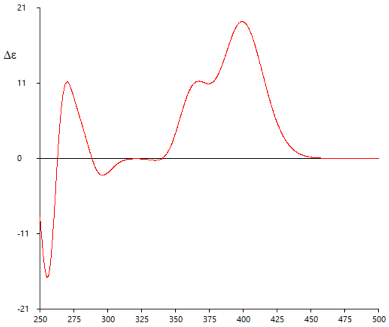

Conf 7 1.33

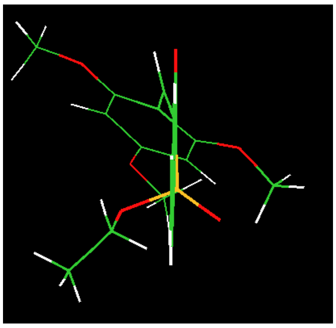

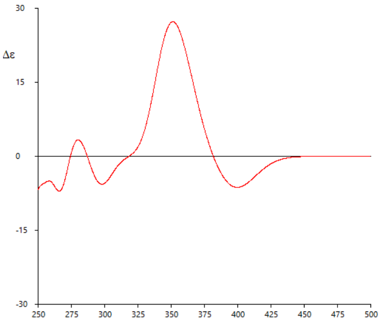

Conf 8 0.76

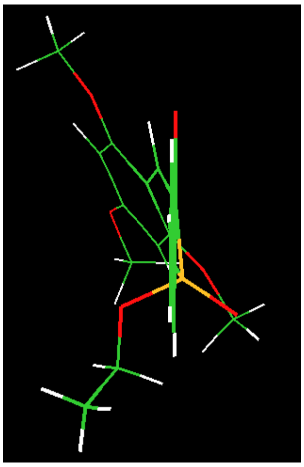

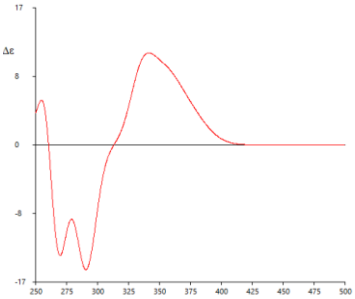

Conf 9	1.03	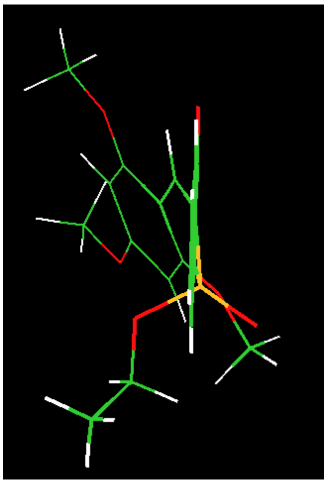	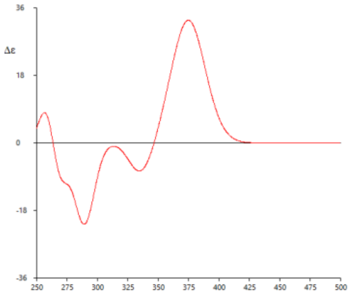
Conf 10	0.00	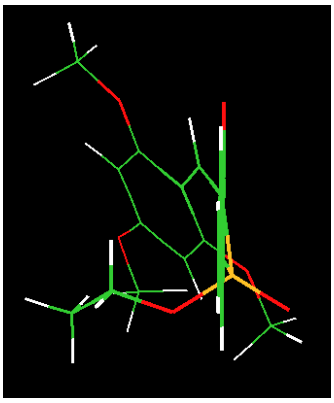	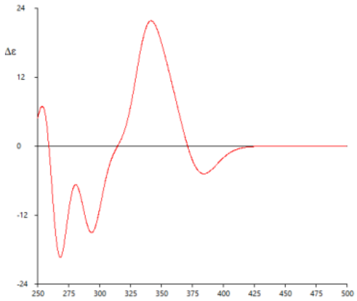

Supplementary Table 5. Conformational analysis of HPI E-(*R*)-**3** with corresponding optimized molecular geometries and calculated ECD spectra on a PBE0/6-311++G(d,p) level of theory.

HPI *E*-(*R*)-**3**	Relative energy [kcal/mol]	Optimized Geometry	Calculated ECD spectrum
Conf 1	0.47	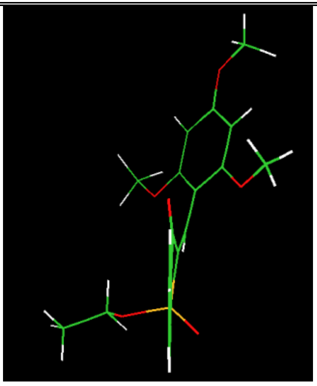	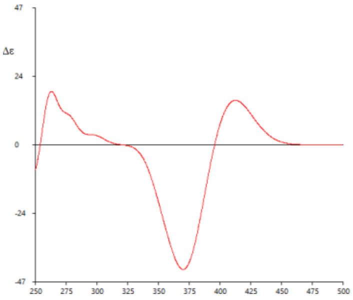
Conf 2	0.25	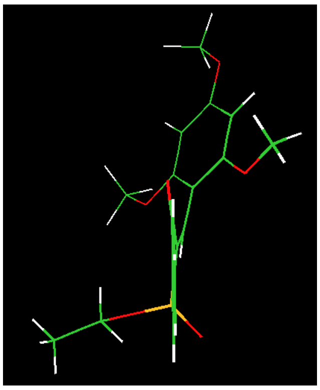	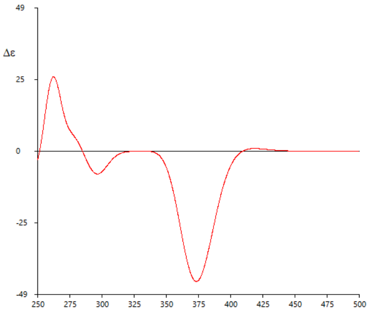
Conf 3	0.29	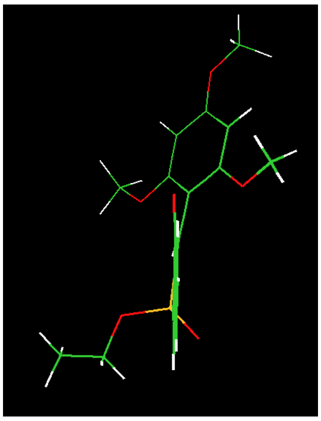	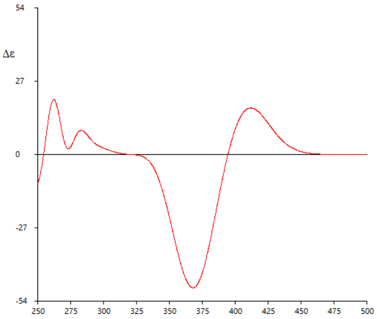
Conf 4	0.58	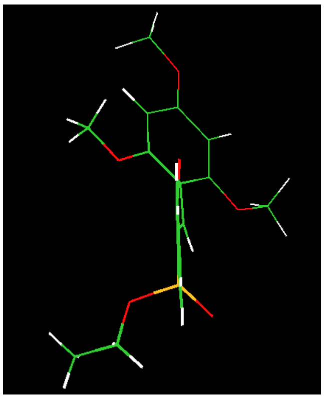	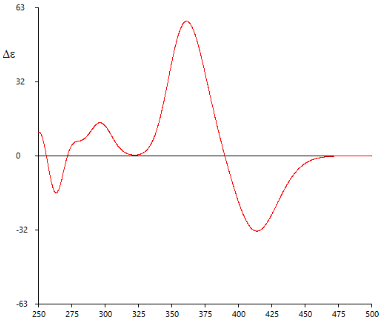

Conf 5 0.04

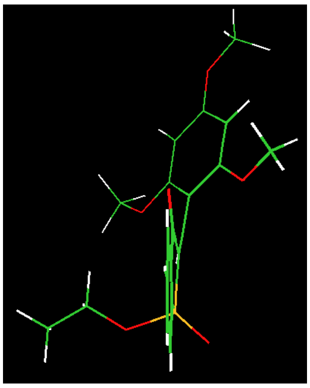

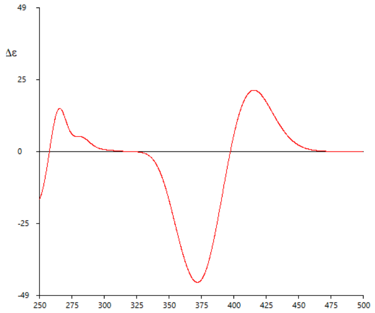

Conf 6 1.51

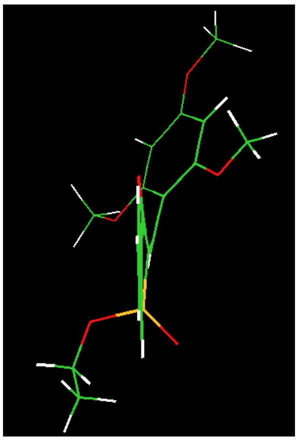

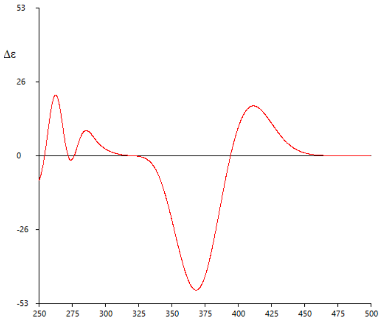

Conf 7 1.51

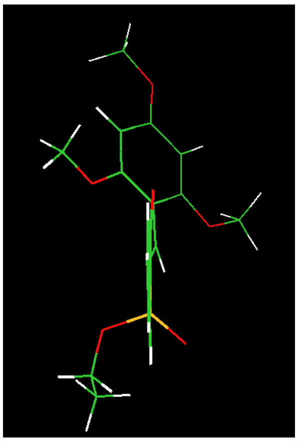

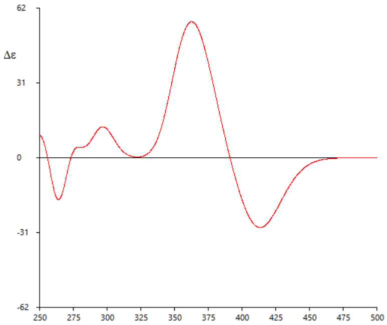

Conf 8 0.00

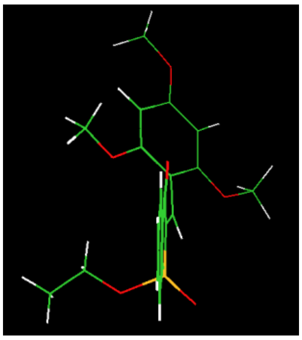

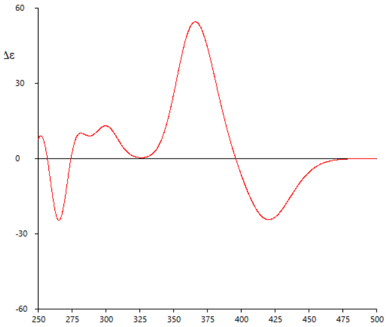

Conf 9 0.43

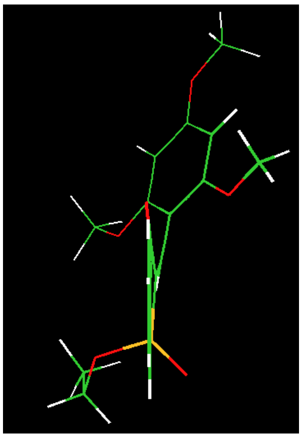

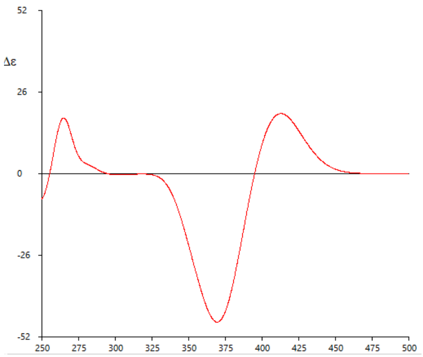

Conf 10 0.61

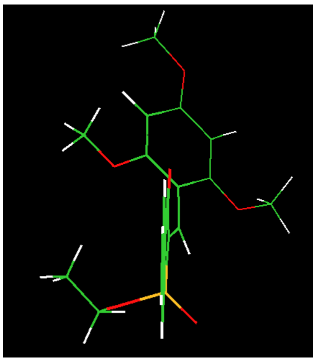

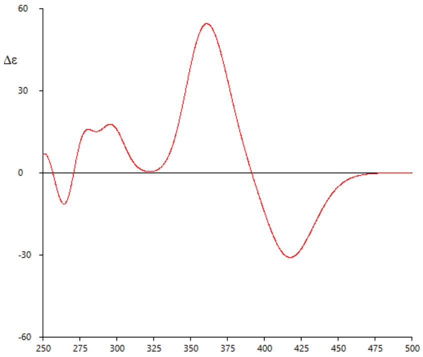

1.1.31 Optimized UV and ECD spectra calculations

In an attempt to obtain more precise energies and calculated spectra in better agreement with the experimental data, optimization and subsequent TDDFT calculations were performed with the best functionals from the screening, namely PBE0, M06 and PW6B95D3. Using the SpecDis program, for each HPI and functional, the calculated UV and ECD spectra were individually fitted to the experimental data employing the similarity factors function, which optimizes the exponential half-width σ and the UV shift correction. Both parameters were then averaged, and all spectra plotted with the averaged parameters. The fitted spectra are summarized in Supplementary Figures 61-66 and the parameters are denoted in Supplementary Table 6. The quality of the calculations can be seen for HPI **10**, where all three levels of theory provide UV and ECD spectra which are in good agreement with the experimental data and the crystal structure of enantiomerically pure *Z* isomer. For the *E* isomers of the twisted HPI structures **3** and **6**, the agreement is still poor (see chapter above), but since the different conformers of the *Z*-isomer only result in a different shift of the bands of the ECD spectrum (see Supplementary Table 4 and not in sign inversion the stereoisomer configuration can still be assigned by comparing the calculated ECD spectra with the experimental ones of the *Z* isomers. Hence, for all three HPIs **3**, **6**, and **10** the first HPLC fraction (HPLC E1) corresponds to the (*R*)-configured enantiomer.

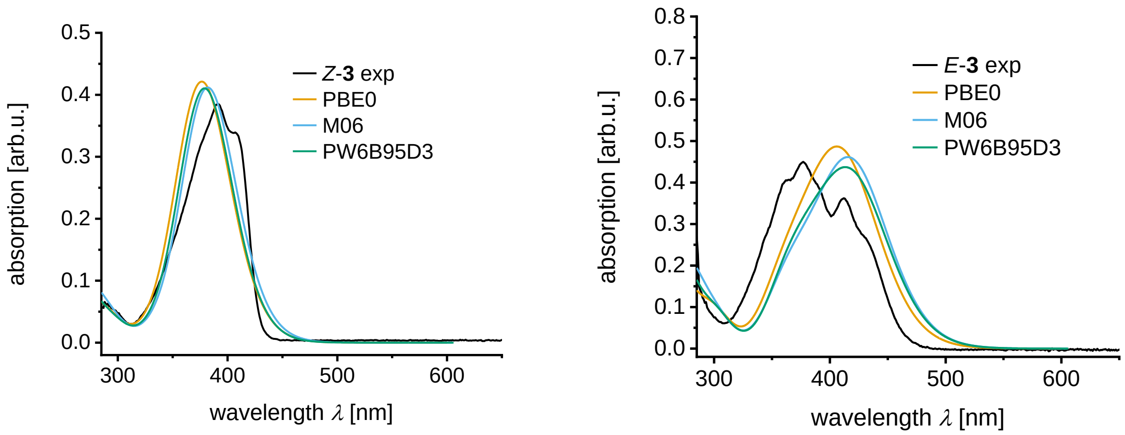

Supplementary Figure 61. Screening of UV spectra for *Z*-**3** (left) and *E*-**3** (right), calculated with the indicated functional and 6-311++G(d,p) as basis set, using a PCM solvent model with toluene as solvent. All TDDFT calculations are based on the structures obtained from the optimizations with the same corresponding level of theory. The fitting parameters are summarized in Supplementary Table 6.

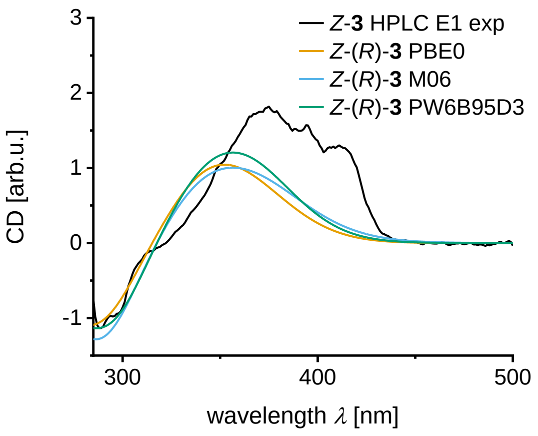

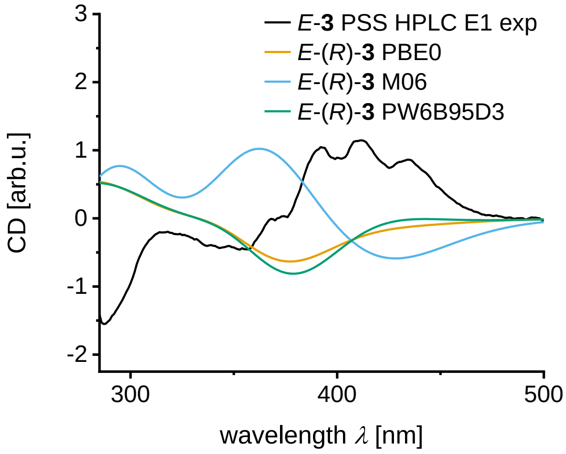

Supplementary Figure 62. Screening of ECD spectra for *Z*-**3** (left) and *E*-**3** (right), calculated with the indicated functional and 6-311++G(d,p) as basis set, using a PCM solvent model with toluene as solvent. All TDDFT calculations are based on the structures obtained from the optimizations with the same corresponding level of theory. The fitting parameters are summarized in Supplementary Table 6.

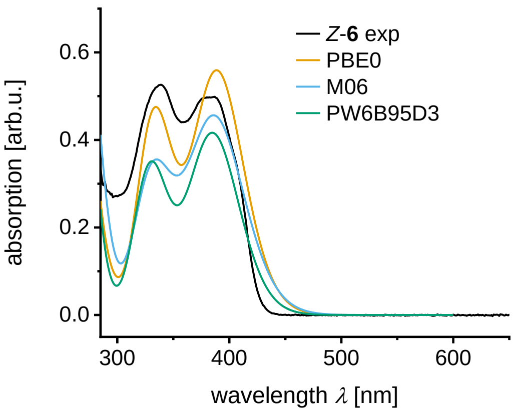

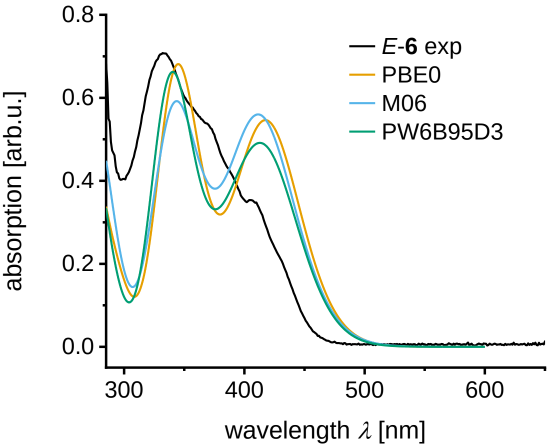

Supplementary Figure 63. Screening of UV spectra for *Z*-**6** (left) and *E*-**6** (right), calculated with the indicated functional and 6-311++G(d,p) as basis set, using a PCM solvent model with toluene as solvent. All TDDFT calculations are based on the structures obtained from the optimizations with the same corresponding level of theory. The fitting parameters are summarized in Supplementary Table 6.

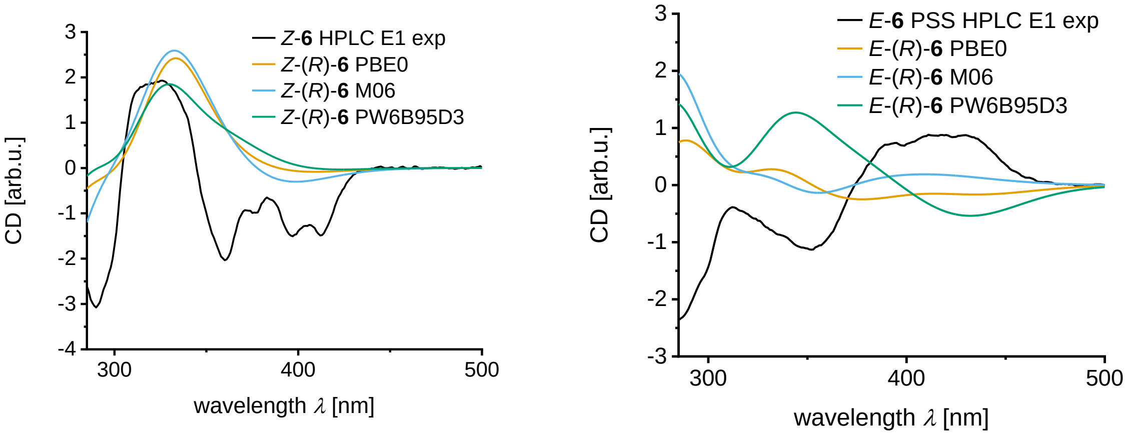

Supplementary Figure 64. Screening of ECD spectra for *Z*-**6** (left) and *E*-**6** (right), calculated with the indicated functional and 6-311++G(d,p) as basis set, using a PCM solvent model with toluene as solvent. All tddft calculations are based on the structures obtained from the optimizations with the same corresponding level of theory. The fitting parameters are summarized in Supplementary Table 6.

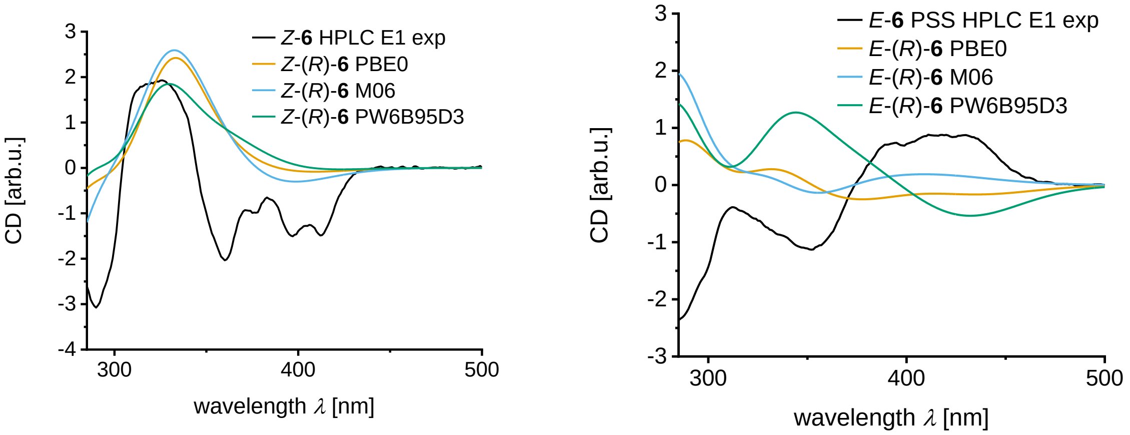

Supplementary Figure 65. Screening of UV spectra for *Z*-**10** (left) and *E*-**10** (right), calculated with the indicated functional and 6-311++G(d,p) as basis set, using a PCM solvent model with toluene as solvent. All TDDFT calculations are based on the structures obtained from the optimizations with the same corresponding level of theory. The fitting parameters are summarized in Supplementary Table 6.

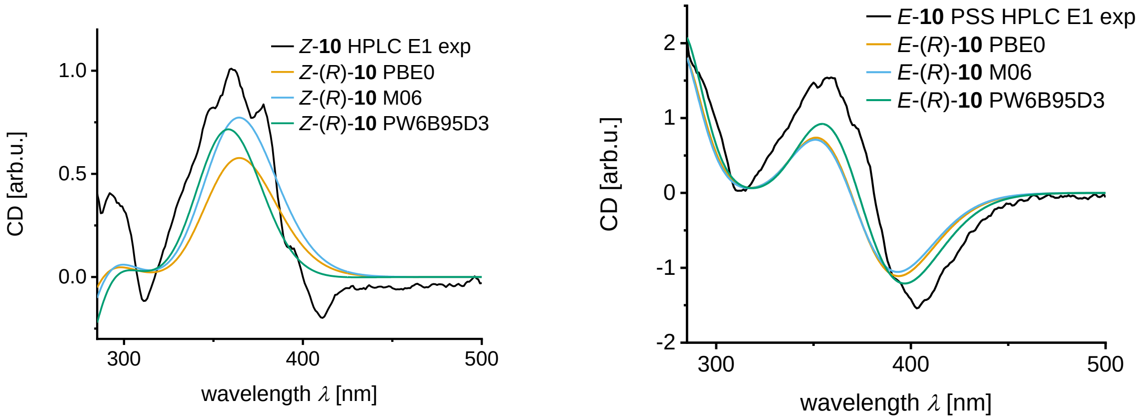

Supplementary Figure 66. Screening of ECD spectra for *Z*-**10** (left) and *E*-**10** (right), calculated with the indicated functional and 6-311++G(d,p) as basis set, using a PCM solvent model with toluene as solvent. All TDDFT calculations are based on the structures obtained from the optimizations with the same corresponding level of theory. The fitting parameters are summarized in Supplementary Table 6.

Supplementary Table 6. Summary of the fitting parameters for the calculated spectra shown in Supplementary Figure 61-66.

HPI	Level of theory	Average UV/ECD shift correction [nm]	Average σ [eV]
**3**	PBE0/ 6-311++ G(d,p)	0	0.29
**3**	M06/ 6-311++ G(d,p)	5	0.29
**3**	PW6B95D3/ 6-311++ G(d,p)	6	0.29
**6**	PBE0/ 6-311++ G(d,p)	-10	0.245
**6**	M06/ 6-311++ G(d,p)	-10	0.27
**6**	PW6B95D3/ 6-311++ G(d,p)	-10	0.24
**10**	PBE0/ 6-311++ G(d,p)	-3	0.255
**10**	M06/ 6-311++ G(d,p)	-4	0.255
**10**	PW6B95D3/ 6-311++ G(d,p)	1	0.255

Supplementary Note 8: Photoisomerization reactions in toluene

The isomer composition in the pss is generally a function of quantum yields for individual transformations of both isomers and their spectral overlaps at a given wavelength of irradiation. Since LEDs were used for irradiation their corresponding emission profiles have also to be taken into account instead of just their nominal maximum wavelengths, which can lead to deviations in the observed pss compositions from the expected ones at a single wavelength. Information about the used LEDs emissions can be found e.g. here Ref.[9,13]

Upon exposure to light of different wavelengths, all HPIs undergo *Z* to *E* isomerization resulting in a new similar signal set with different chemical shift, corresponding to the *E* isomer. To further evidence the *E*-isomer being the product of the photoisomerization reaction, NOE experiments were performed with both isomers of HPI **6** after isomer separation using HPLC. The experiments are shown in Supplementary Figure 67 and 68 and clearly evidencing the configuration of the central double bond.

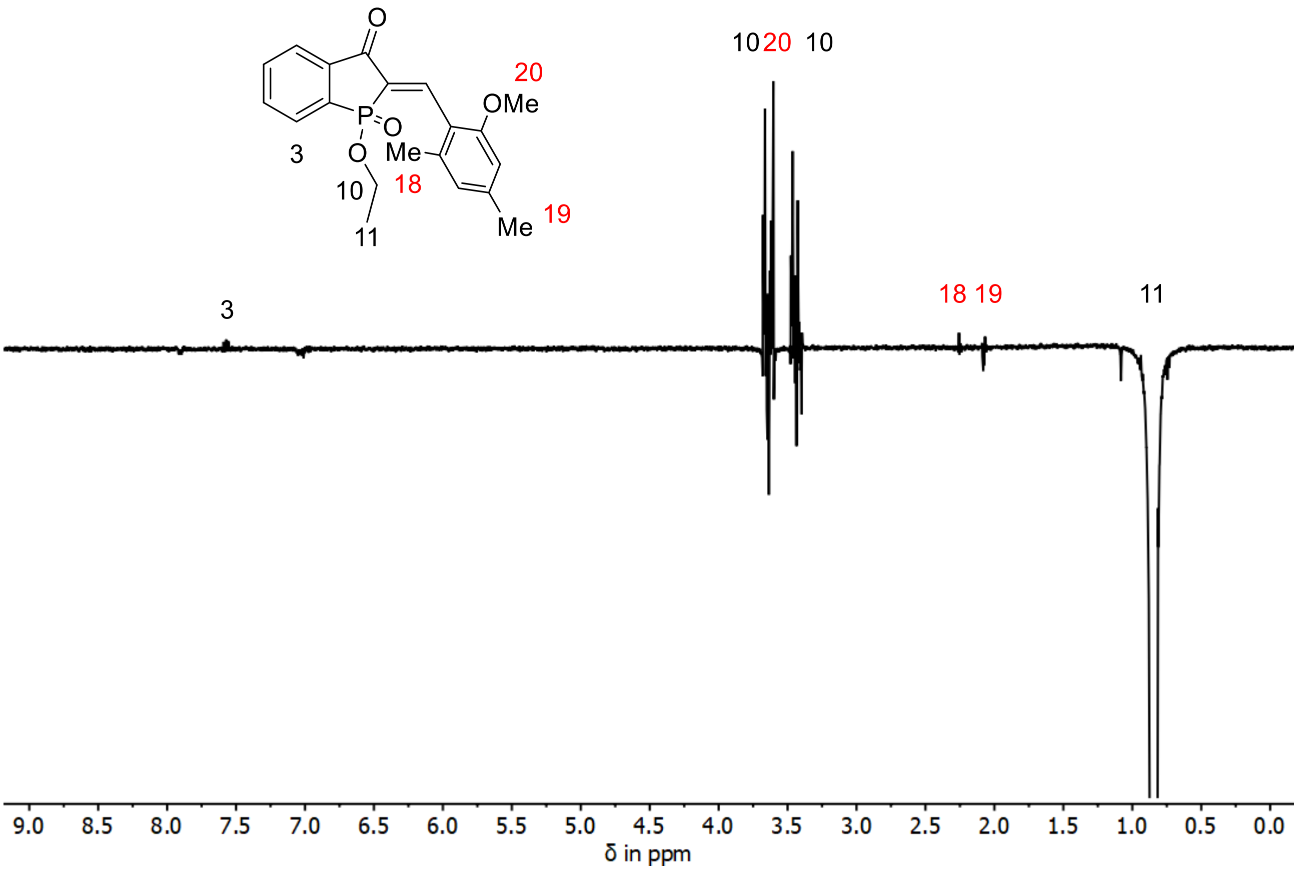

Supplementary Figure 67. Determination of the configuration of the central double bond of HPI *Z*-**6** with a 1D NOE (500 MHz, toluene-d_8, 25 °C) experiment. Selective irradiation of the CH_3 group 11 reveals through space coupling to 18, 19 and 20, evidencing *Z* configuration.

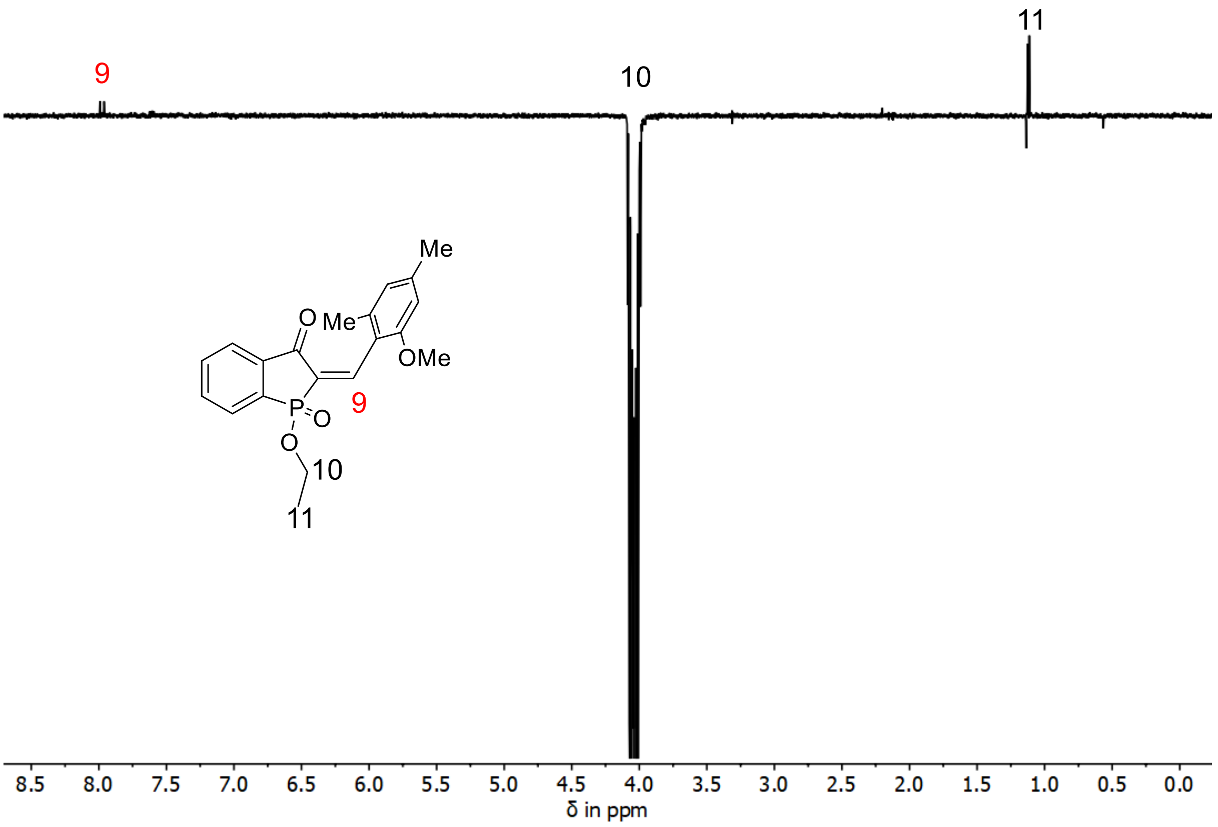

Supplementary Figure 68. Determination of the configuration of the central double bond of HPI *E*-**6** with a 1D NOE (600 MHz, toluene-d_8, 25 °C) experiment. Selective irradiation of the CH_2 group 10 reveals through space coupling to 9 evidencing *E* configuration.

Moreover, a $^3J_{H\text{-}P}$ vicinal coupling constant analysis was conducted for both isomers of each derivative which are summarized in Supplementary Table 7. All *Z* isomers show coupling constants of 32.7-36.4 Hz, while the corresponding *E* isomers display smaller coupling constants with values of 16.7-20.6 Hz. This behavior is in very good agreement with the *Karplus* relationship,[14-15] originally describing the dependence of vicinal H-H coupling constants of the dihedral angle. This behavior results from the degree of orbital overlap, where maximum overlap occurs at a dihedral angle of 0° and 180°, resulting in large H-H coupling constants and low overlap at 90°. In the case of vicinal *Z* or *E* protons in alkenes, maximum orbital is achieved in *E* conformation, since the C-H bonds in question are parallel, in contrary the C-H bonds in *Z* conformation are tilted in different directions, hence decreasing orbital overlap and thus lowering the coupling constant for *Z* conformation. This behavior can also be seen for H-P couplings. In the *Z* isomer of the HPIs, the phosphorous is *trans* to the proton it couples with and in consequence displaying larger coupling constants than in the case of the *E* isomer.

Supplementary Table 7. ${}^3J_{H-P}$ Vicinal coupling constant analysis of the proton located at the central double bond of both *Z* and *E* isomers of all HPI derivatives measured on a 400 MHz NMR spectrometer.

HPI	Solvent	${}^3J_{H-P}$ *Z*-isomer [Hz]	${}^3J_{H-P}$ *E*-isomer [Hz]
**1**	toluene-d_8	35.4	18.4
**2**	toluene-d_8	35.1	18.4
**3**	toluene-d_8	36.4	18.3
**3-OH**	D_2O	33.0	16.7
**4**	toluene-d_8	36.4	18.4
**5**	toluene-d_8	34.7	18.3
**6**	toluene-d_8	35.5	18.4
**7**	toluene-d_8	34.9	18.4
**8**	toluene-d_8	34.6	19.6
**9**	toluene-d_8	34.4	20.1
**10**	toluene-d_8	34.5	20.4
**11**	toluene-d_8	34.6	20.6
**12**	toluene-d_8	34.0	20.1
**13**	toluene-d_8	34.6	20.1
**14**	toluene-d_8	33.9	20.0
**15**	toluene-d_8	33.7	18.5
**16**	toluene-d_8	32.7	17.3
**17**	toluene-d_8	32.9	17.4
**18**	toluene-d_8	34.4	19.0

For more proof of the geometry of the different HPIs in solution, a shift analysis was conducted for the planar and twisted derivatives **1**-**14**. The chemical shifts of the ethyl group of the phosphinate in the ^{1}H NMR spectra were compared (Supplementary Table 8). In case of the twisted derivatives **1**-**7**, interaction between the ring current of the aromatic ring of the stilbene part is possible, which leads to an upfield shift for the signals of the CH_2 and CH_3 groups compared to those of the planar derivatives **8**-**14**. In case of the *E* isomers, no interaction is possible due to the large distance in this geometry, which leads to an apparent downfield shift. A similar analysis of aromatic ring-current induced signal shifting in HTIs to evidence twisting in solution was reported previously by our group.[16-17]

Supplementary Table 8. Chemical shift analysis of the ^{1}H NMR signal of the ethyl group of the phosphinate (400 MHz, 23 °C). For comparison, the chemical shift is given in ppm and the value is given for the center of the signal.

HPI	CH_2 signal(s) [ppm]	CH_3 signal [ppm]
***Z*-1**	3.80/3.50	1.05
***E*-1**	4.03	1.10
***Z*-2**	3.85/3.57	1.09
***Z*-3**	3.77/ 3.62	1.14
***Z*-4**	3.82/3.65	1.15
***Z*-5**	3.86/3.59	1.10
***Z*-6**	3.64/3.43	0.83
***Z*-7**	3.74/3.50	0.84
***Z*-8**	3.90	1.20
***Z*-9**	3.90	1.19
***Z*-10**	3.95	1.19
***Z*-11**	4.00	1.18
***Z*-12**	4.02	1.20
***Z*-13**	4.03	1.20
***Z*-14**	4.05	1.22

1.1.32 Photoisomerization of HPI 1

a)

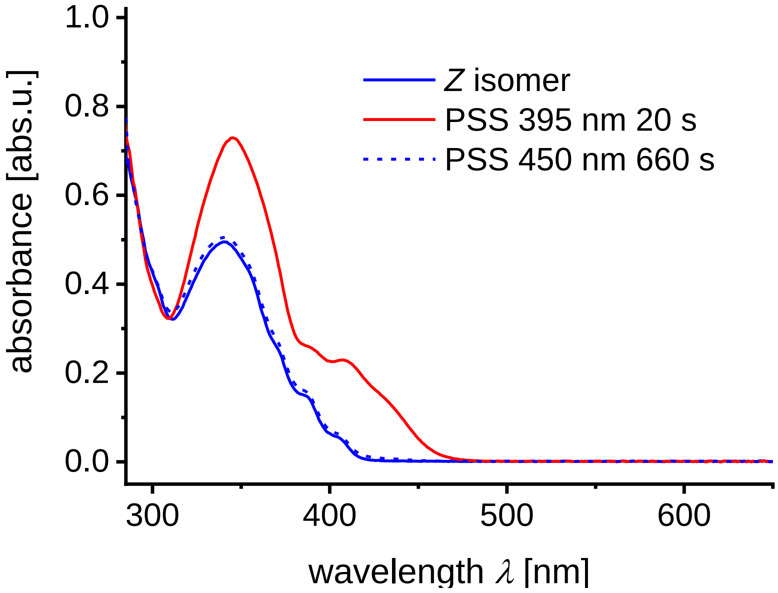

b)

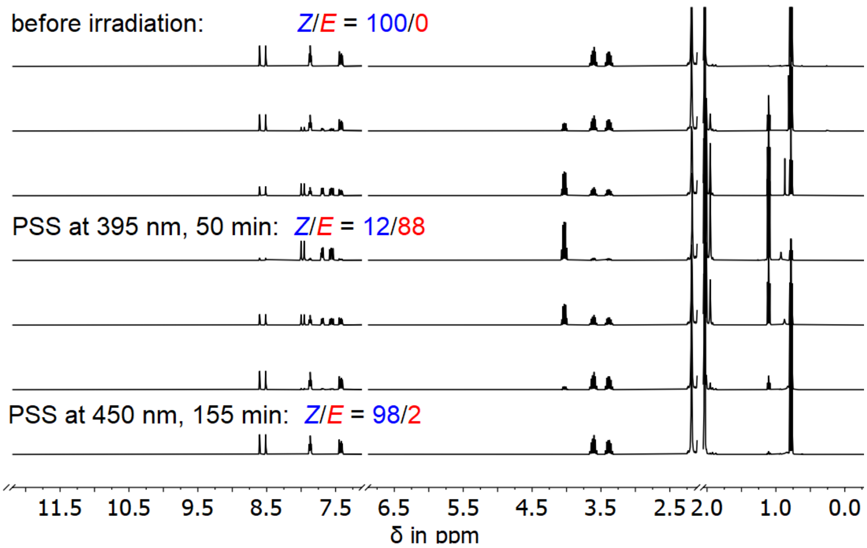

Supplementary Figure 69. Photoconversion of HPI **1** upon irradiation with 395 nm and 450 nm light. a) UV/Vis absorption spectra of HPI **1** in toluene at 23 °C recorded before (blue) and after irradiation with 395 nm (red) and 450 nm (blue dashed). The spectra after irradiation were recorded after reaching the PSS. b) ^{1}H NMR spectra (400 MHz, toluene-d_8, 23 °C) of a different sample of HPI **1** recorded before and after irradiation using 395 nm and 450 nm light. The NMR spectra are shown in the order of the experiments from top to bottom and isomeric composition of *Z* and *E* isomer at PSS are indicated. Unlabeled NMR spectra illustrate the isomer enrichment before reaching the PSS.

1.1.33 Photoisomerization of HPI 2

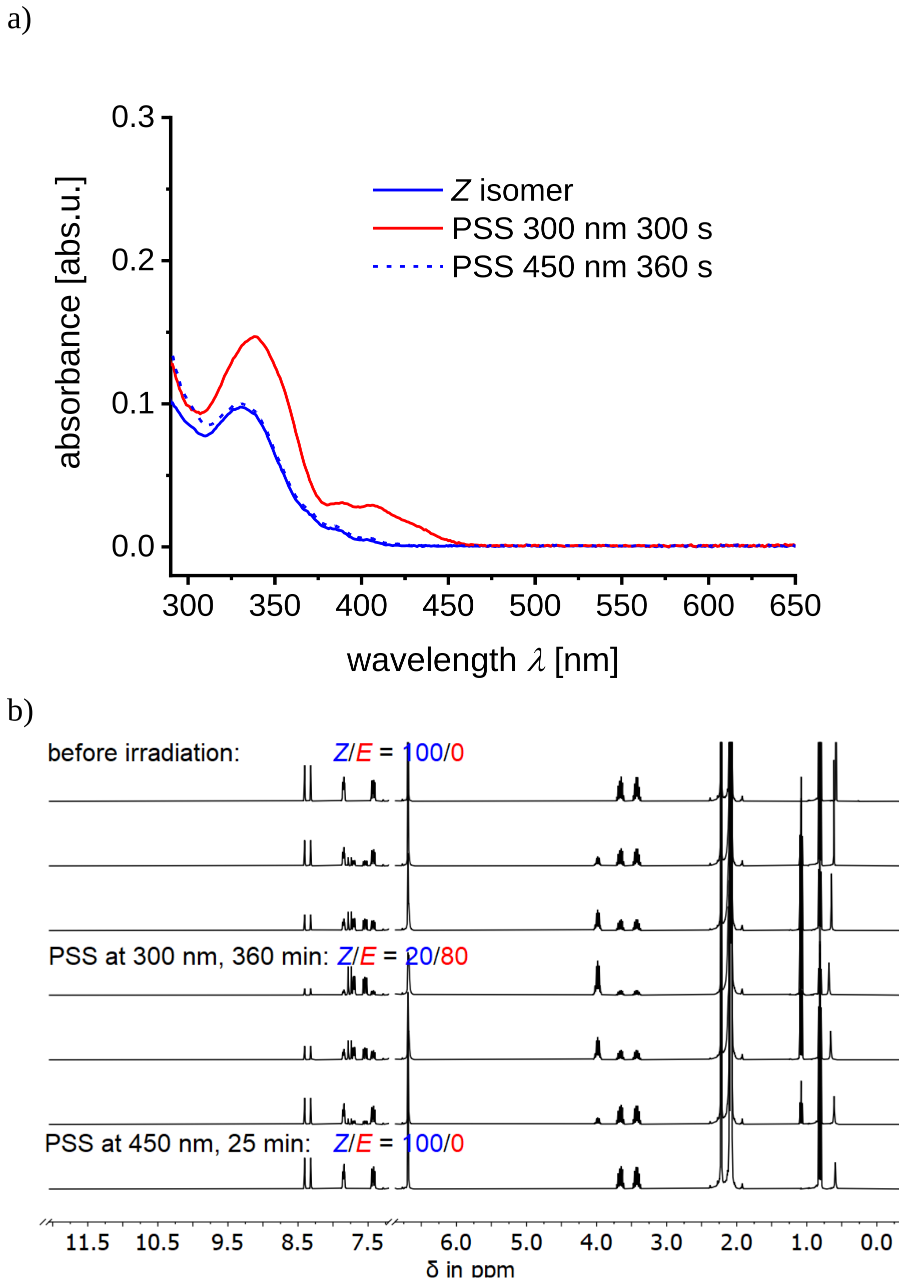

Supplementary Figure 70. Photoconversion of HPI **2** upon irradiation with 300 nm and 450 nm light. a) UV/Vis absorption spectra of HPI **2** in toluene at 23 °C recorded before (blue) and after irradiation with 300 nm (red) and 450 nm (blue dashed) light. The spectra after irradiation were recorded after reaching the PSS. b) ^{1}H NMR spectra (400 MHz, toluene-d_8, 23 °C) of a different sample of HPI **2** recorded before and after irradiation using 300 nm and 450 nm light. The NMR spectra are shown in the order of the experiments from top to bottom and isomeric composition of *Z* and *E* isomer at PSS are indicated. Unlabeled NMR spectra illustrate the isomer enrichment before reaching the PSS.

1.1.34 Photoisomerization of HPI 3

a)

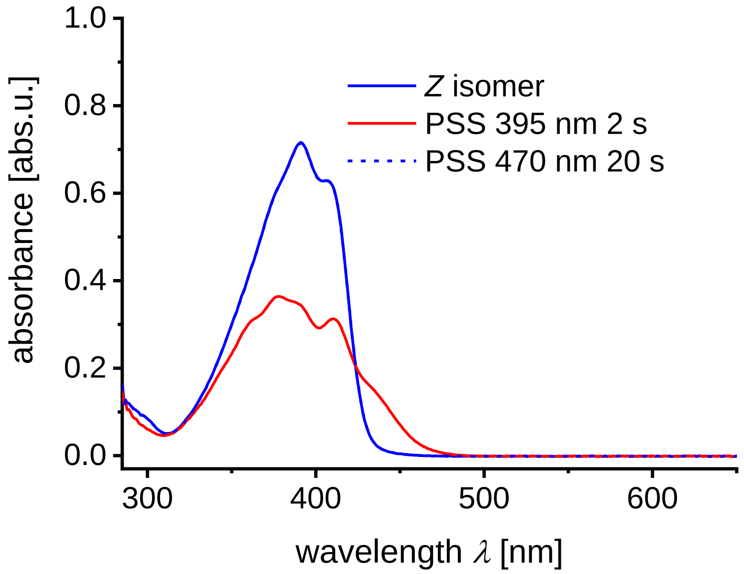

b)

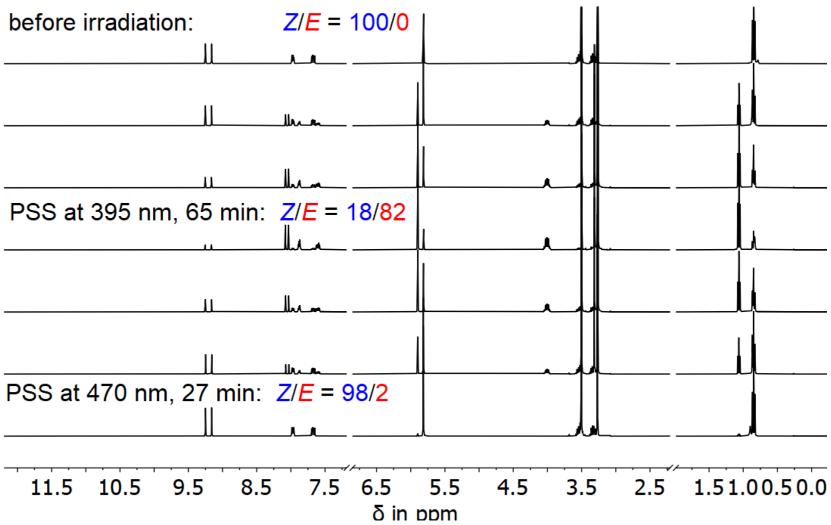

Supplementary Figure 71. Photoconversion of HPI **3** upon irradiation with 395 nm and 470 nm light. a) UV/Vis absorption spectra of HPI **3** in toluene at 23 °C recorded before (blue) and after irradiation with 395 nm (red) and 470 nm (blue dashed) light. The spectra after irradiation were recorded after reaching the PSS. b) ^{1}H NMR spectra (400 MHz, toluene-d_8, 23 °C) of a different sample of HPI **3** recorded before and after irradiation using 395 nm and 470 nm light. The NMR spectra are shown in the order of the experiments from top to bottom and isomeric composition of *Z* and *E* isomer at PSS are indicated. Unlabeled NMR spectra illustrate the isomer enrichment before reaching the PSS.

1.1.35 Photoisomerization of HPI 4

a)

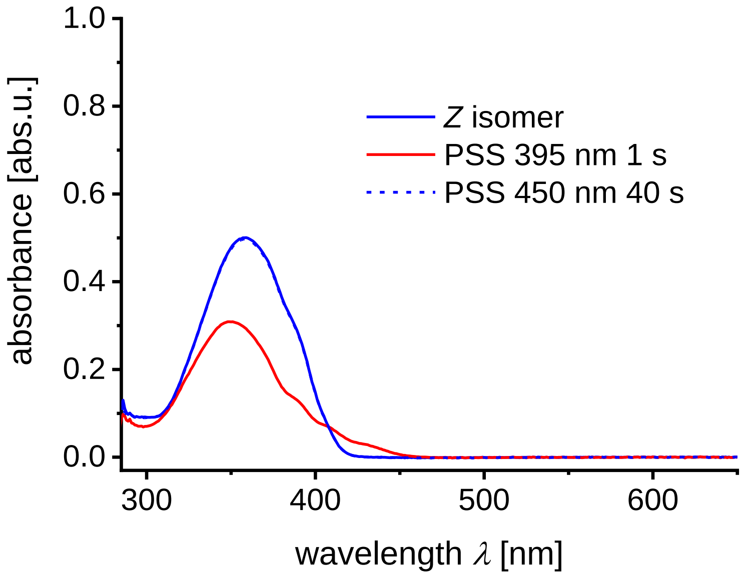

b)

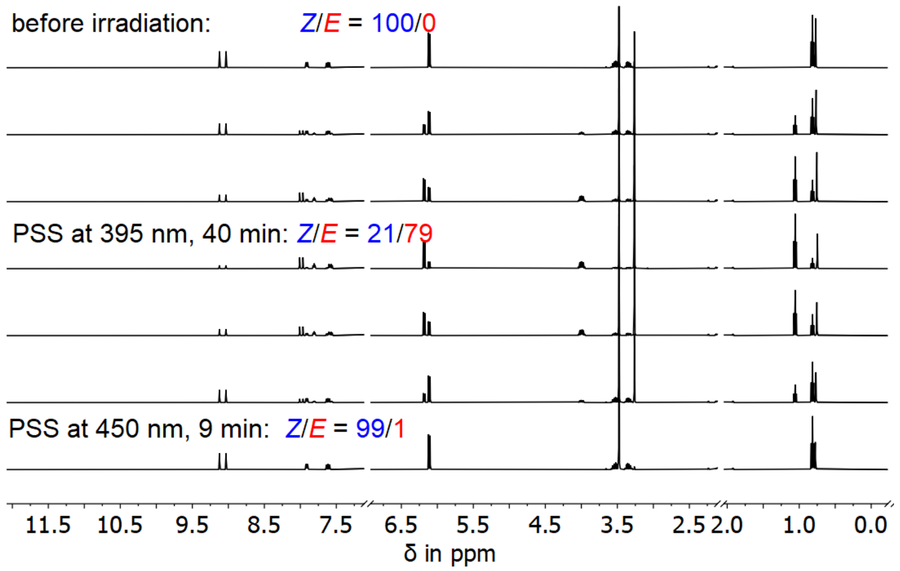

Supplementary Figure 72. Photoconversion of HPI **4** upon irradiation with 395 nm and 450 nm light. a) UV/Vis absorption spectra of HPI **4** in toluene at 23 °C recorded before (blue) and after irradiation with 395 nm (red) and 450 nm (blue dashed) light. The spectra after irradiation were recorded after reaching the PSS. b) ^{1}H NMR spectra (400 MHz, toluene-d_8, 23 °C) of a different sample of HPI **4** recorded before and after irradiation using 395 nm and 450 nm light. The NMR spectra are shown in the order of the experiments from top to bottom and isomeric composition of *Z* and *E* isomer at PSS are indicated. Unlabeled NMR spectra illustrate the isomer enrichment before reaching the PSS.

1.1.36 Photoisomerization of HPI 5

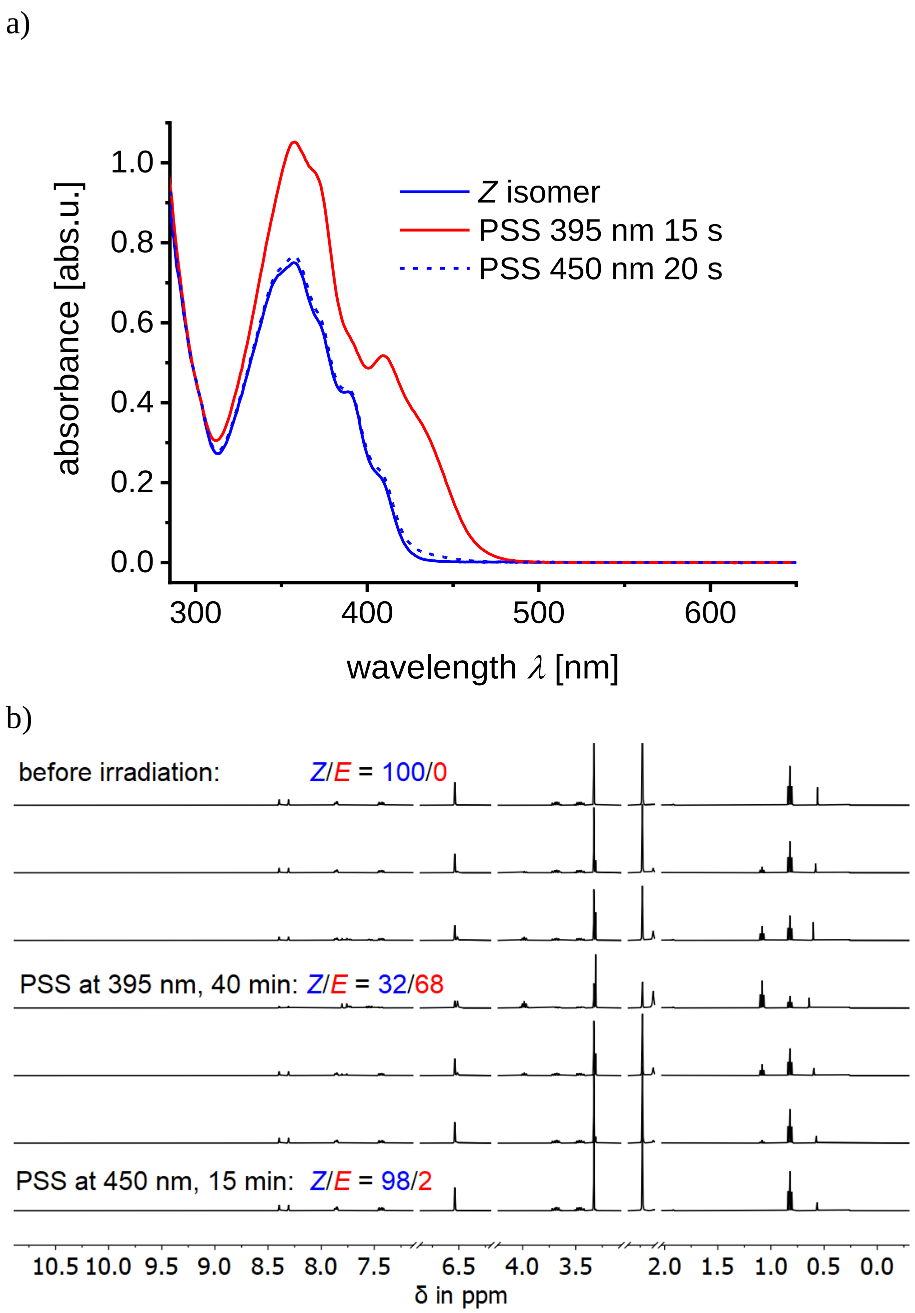

Supplementary Figure 73. Photoconversion of HPI **5** upon irradiation with 395 nm and 450 nm light. a) UV/Vis absorption spectra of HPI **5** in toluene at 23 °C recorded before (blue) and after irradiation with 395 nm (red) and 450 nm (blue dashed) light. The spectra after irradiation were recorded after reaching the PSS. b) ^{1}H NMR spectra (400 MHz, toluene-d_8, 23 °C) of a different sample of HPI **5** recorded before and after irradiation using 395 nm and 450 nm light. The NMR spectra are shown in the order of the experiments from top to bottom and isomeric composition of *Z* and *E* isomer at PSS are indicated. Unlabeled NMR spectra illustrate the isomer enrichment before reaching the PSS.

1.1.37 Photoisomerization of HPI 6

a)

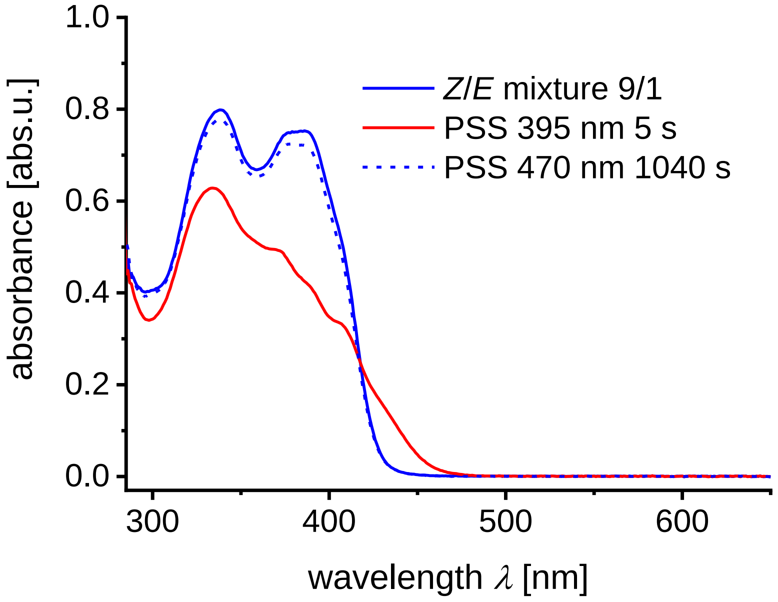

b)

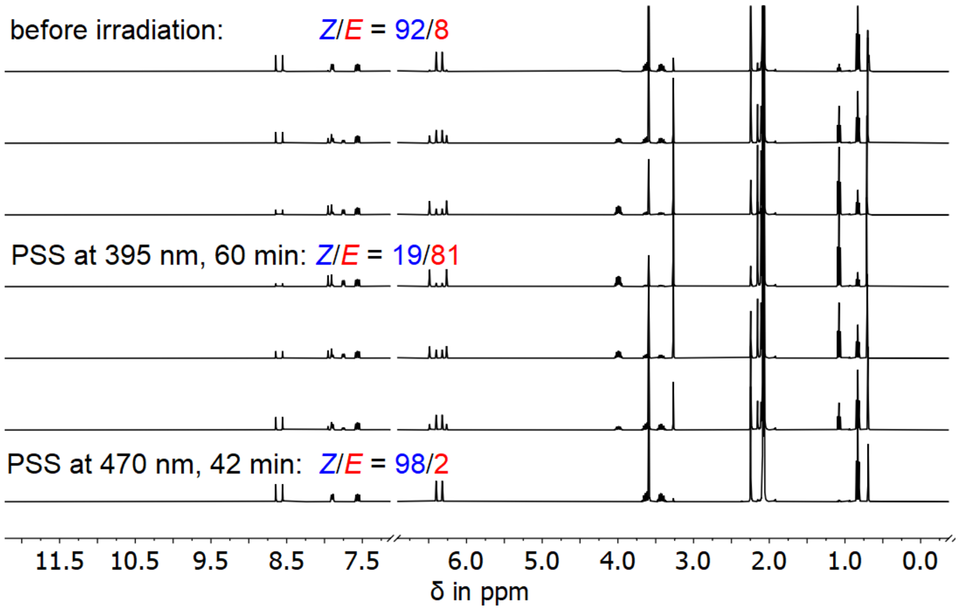

Supplementary Figure 74. Photoconversion of HPI **6** upon irradiation with 395 nm and 470 nm light. a) UV/Vis absorption spectra of HPI **6** in toluene at 23 °C recorded before (blue) and after irradiation with 395 nm (red) and 470 nm (blue dashed) light. The spectra after irradiation were recorded after reaching the PSS. b) ^{1}H NMR spectra (400 MHz, toluene-d_8, 23 °C) of a different sample of HPI **6** recorded before and after irradiation using 395 nm and 470 nm light. The NMR spectra are shown in the order of the experiments from top to bottom and isomeric composition of *Z* and *E* isomer at PSS are indicated. Unlabeled NMR spectra illustrate the isomer enrichment before reaching the PSS.

1.1.38 Photoisomerization of HPI 7

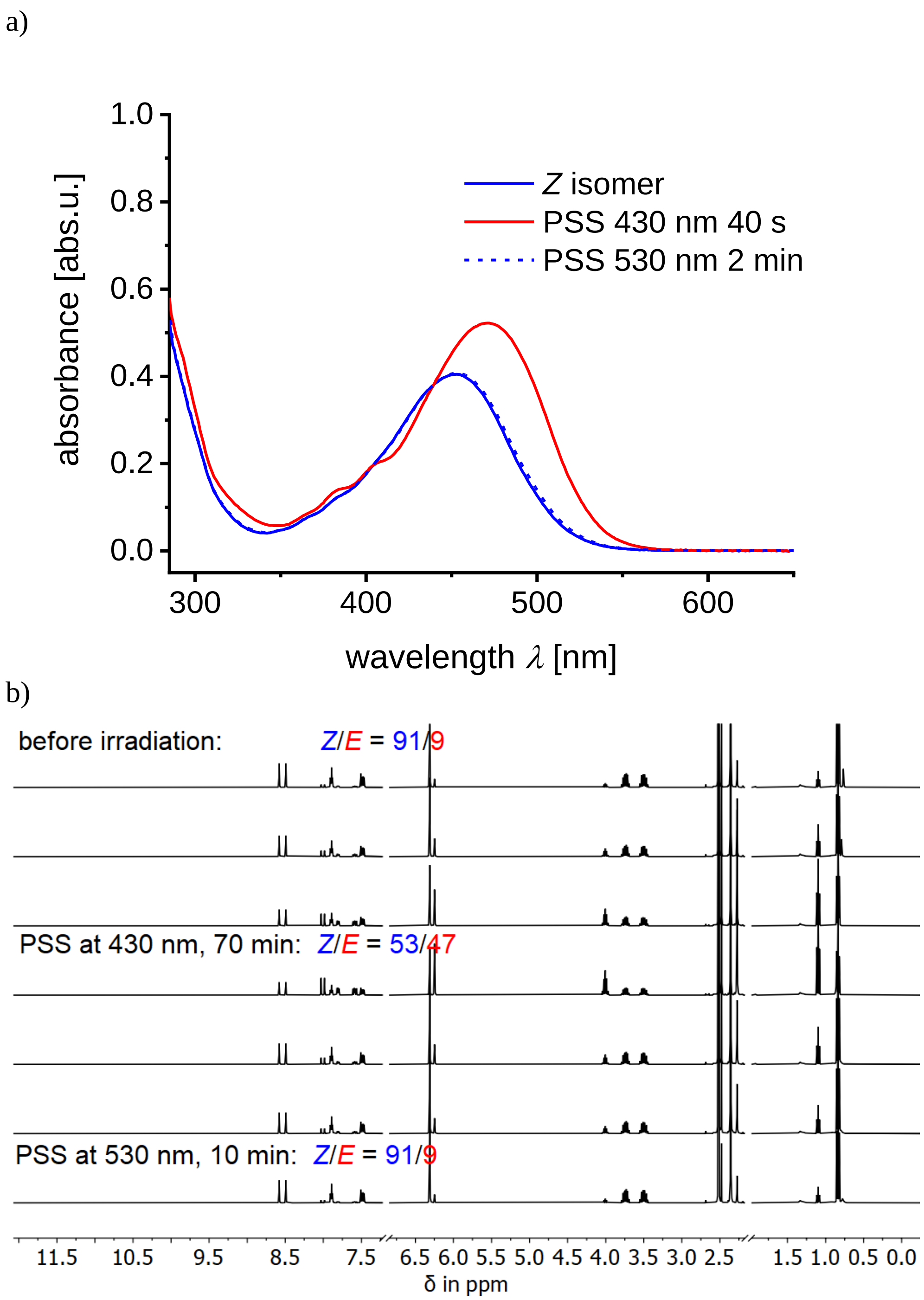

Supplementary Figure 75. Photoconversion of HPI **7** upon irradiation with 430 nm and 530 nm light. a) UV/Vis absorption spectra of HPI **7** in toluene at 23 °C recorded before (blue) and after irradiation with 430 nm (red) and 530 nm (blue dashed) light. The spectra after irradiation were recorded after reaching the PSS. b) ^{1}H NMR spectra (400 MHz, toluene-d_8, 23 °C) of a different sample of HPI **7** recorded before and after irradiation using 430 nm and 530 nm light. The NMR spectra are shown in the order of the experiments from top to bottom and isomeric composition of *Z* and *E* isomer at PSS are indicated. Unlabeled NMR spectra illustrate the isomer enrichment before reaching the PSS.

1.1.39 Photoisomerization of HPI 8

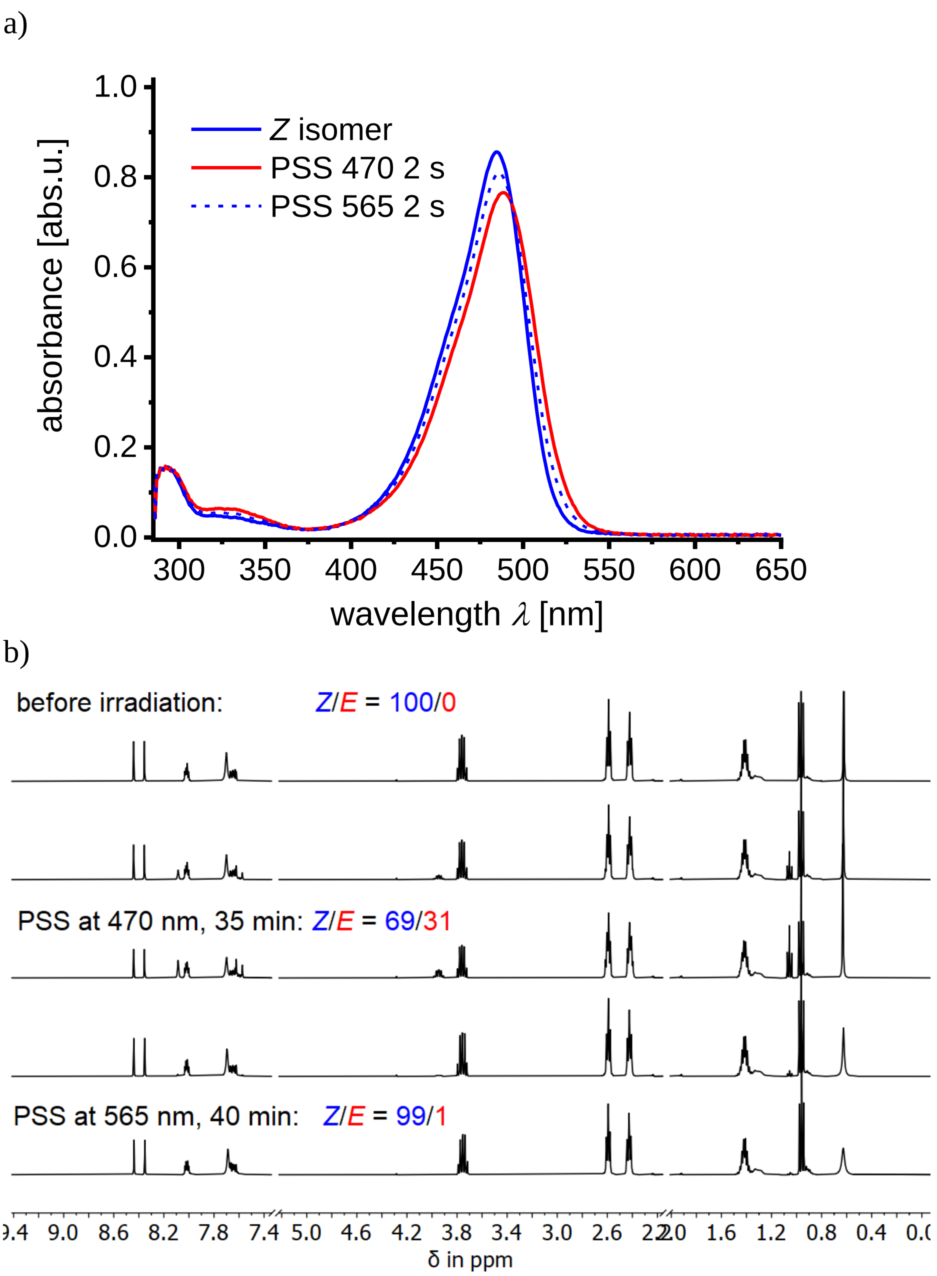

Supplementary Figure 76. Photoconversion of HPI **8** upon irradiation with 470 nm and 565 nm light. a) UV/Vis absorption spectra of HPI **8** in toluene at 23 °C recorded before (blue) and after irradiation with 470 nm (red) and 565 nm (blue dashed) light. The spectra after irradiation were recorded after reaching the PSS. b) ^{1}H NMR spectra (400 MHz, toluene-d_8, 23 °C) of a different sample of HPI **8** recorded before and after irradiation using 470 nm light. The NMR spectra are shown in the order of the experiments from top to bottom and isomeric composition of *Z* and *E* isomer at PSS are indicated. Unlabeled NMR spectra illustrate the isomer enrichment before reaching the PSS.

1.1.40 Photoisomerization of HPI 9

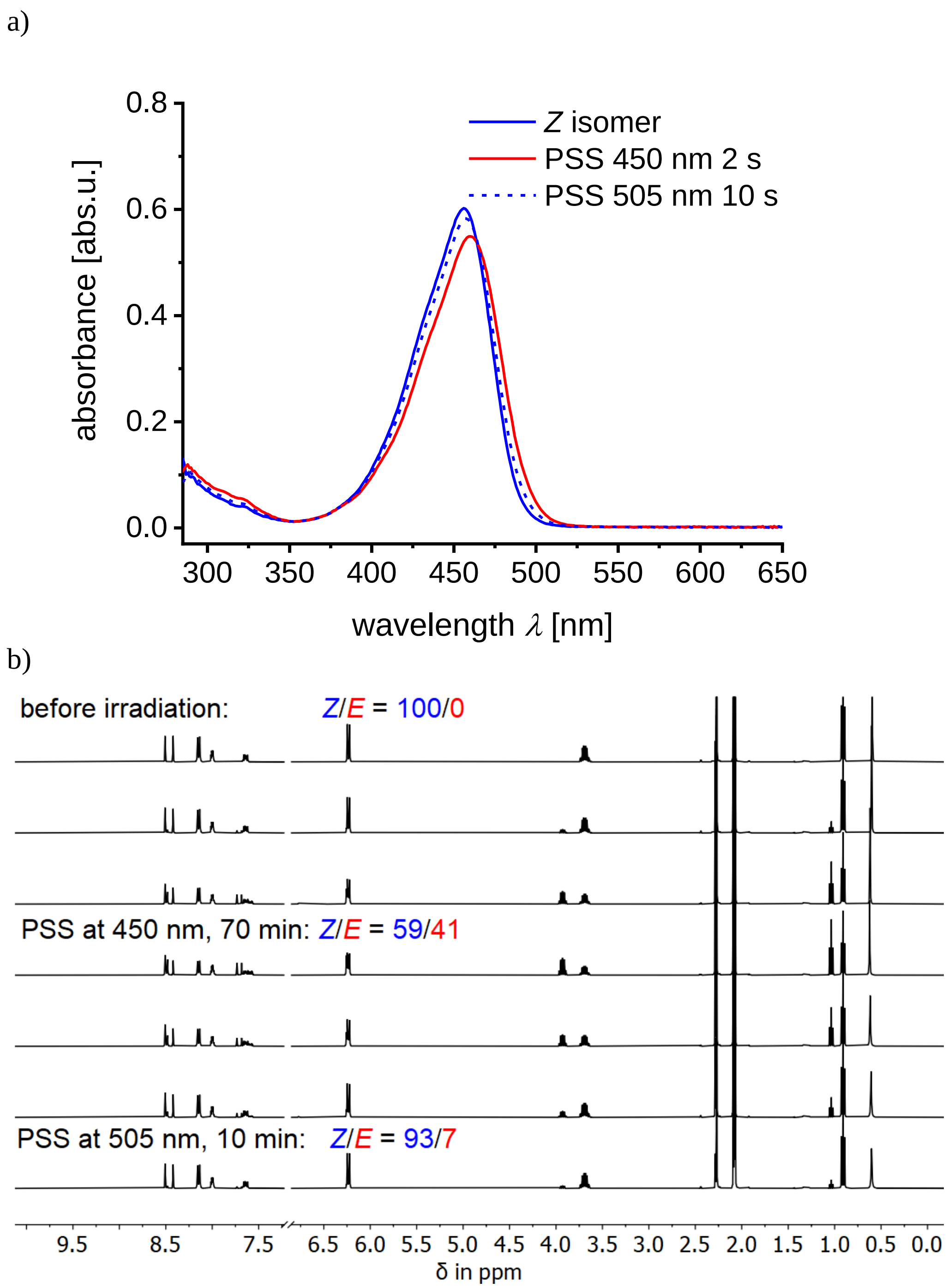

Supplementary Figure 77. Photoconversion of HPI **9** upon irradiation with 450 nm and 505 nm light. a) UV/Vis absorption spectra of HPI **9** in toluene at 23 °C recorded before (blue) and after irradiation with 450 nm (red) and 505 nm (blue dashed) light. The spectra after irradiation were recorded after reaching the PSS. b) ^{1}H NMR spectra (400 MHz, toluene-d_8, 23 °C) of a different sample of HPI **9** recorded before and after irradiation using 450 nm and 505 nm light. The NMR spectra are shown in the order of the experiments from top to bottom and isomeric composition of *Z* and *E* isomer at PSS are indicated. Unlabeled NMR spectra illustrate the isomer enrichment before reaching the PSS.

1.1.41 Photoisomerization of HPI 10

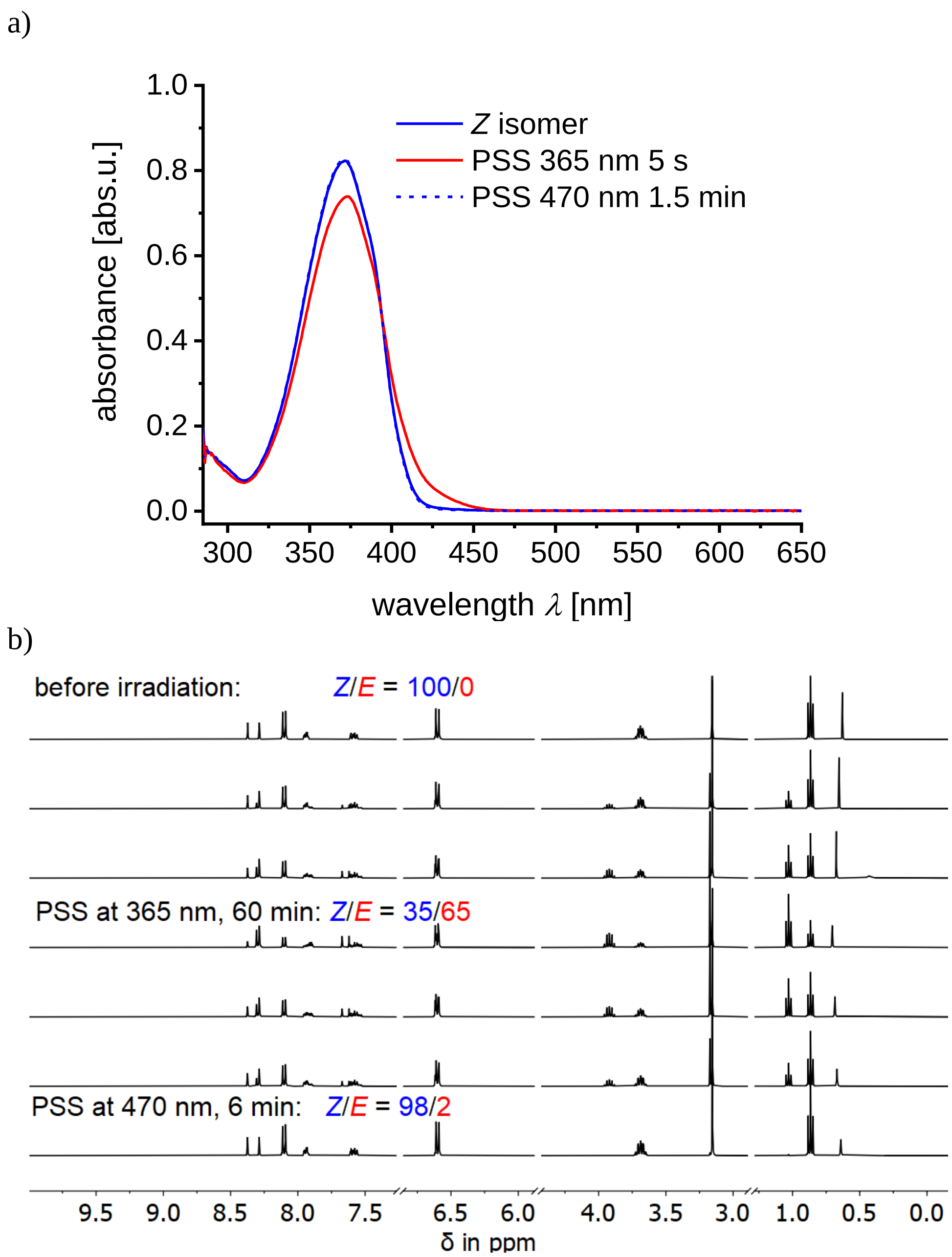

Supplementary Figure 78. Photoconversion of HPI **10** upon irradiation with 365 nm and 470 nm light. a) UV/Vis absorption spectra of HPI **10** in toluene at 23 °C recorded before (blue) and after irradiation with 365 nm (red) and 470 nm (blue dashed) light. The spectra after irradiation were recorded after reaching the PSS. b) ^{1}H NMR spectra (400 MHz, toluene-d_8, 23 °C) of a different sample of HPI **10** recorded before and after irradiation using 365 nm and 470 nm light. The NMR spectra are shown in the order of the experiments from top to bottom and isomeric composition of *Z* and *E* isomer at PSS are indicated. Unlabeled NMR spectra illustrate the isomer enrichment before reaching the PSS.

1.1.42 Photoisomerization of HPI 11

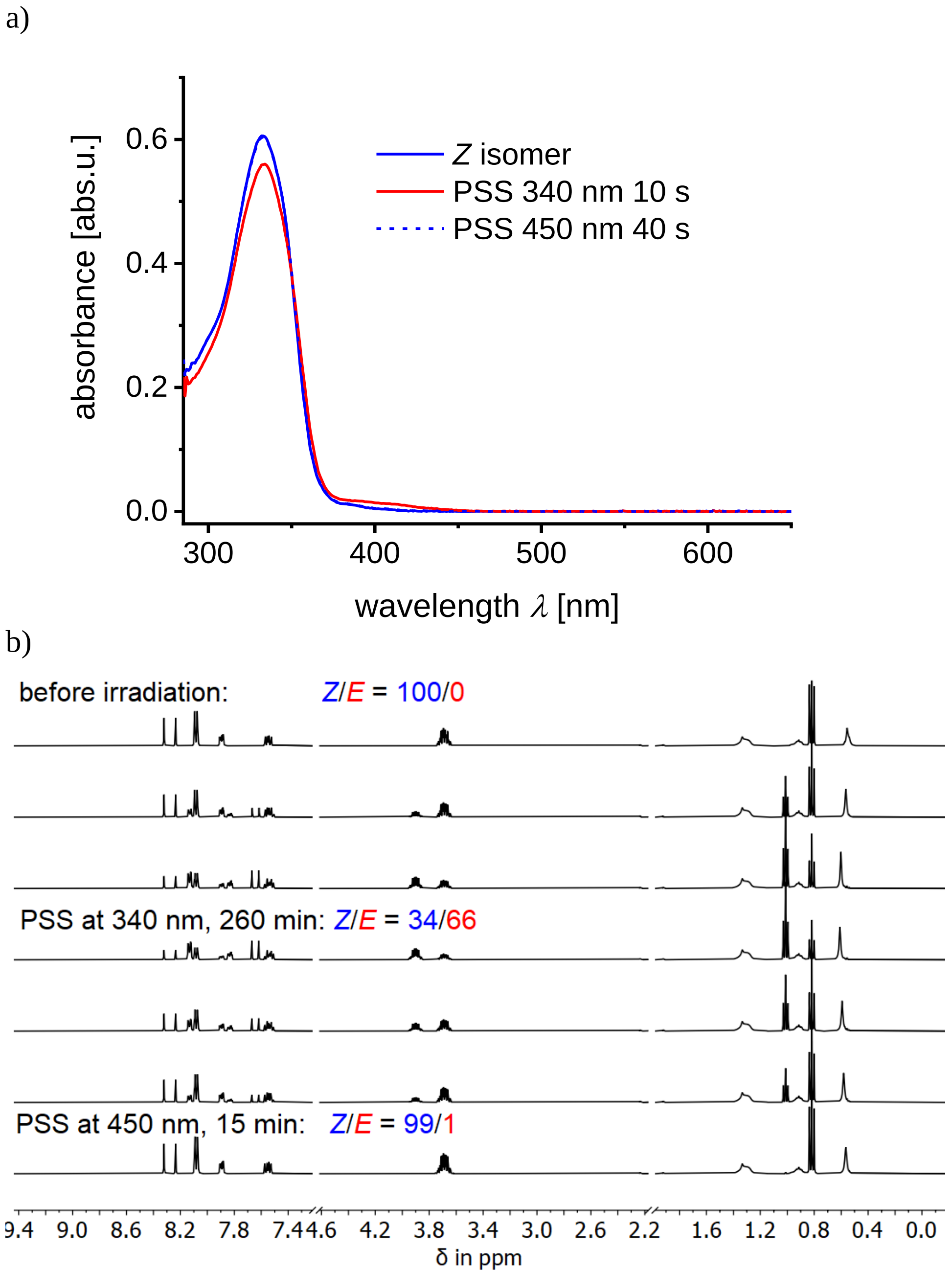

Supplementary Figure 79. Photoconversion of HPI **11** upon irradiation with 340 nm and 450 nm light. a) UV/Vis absorption spectra of HPI **11** in toluene at 23 °C recorded before (blue) and after irradiation with 365 nm (red) and 470 nm (blue dashed) light. The spectra after irradiation were recorded after reaching the PSS. b) ^{1}H NMR spectra (400 MHz, toluene-d_8, 23 °C) of a different sample of HPI **11** recorded before and after irradiation using 340 nm and 450 nm light. The NMR spectra are shown in the order of the experiments from top to bottom and isomeric composition of *Z* and *E* isomer at PSS are indicated. Unlabeled NMR spectra illustrate the isomer enrichment before reaching the PSS.

1.1.43 Photoisomerization of HPI 12

a)

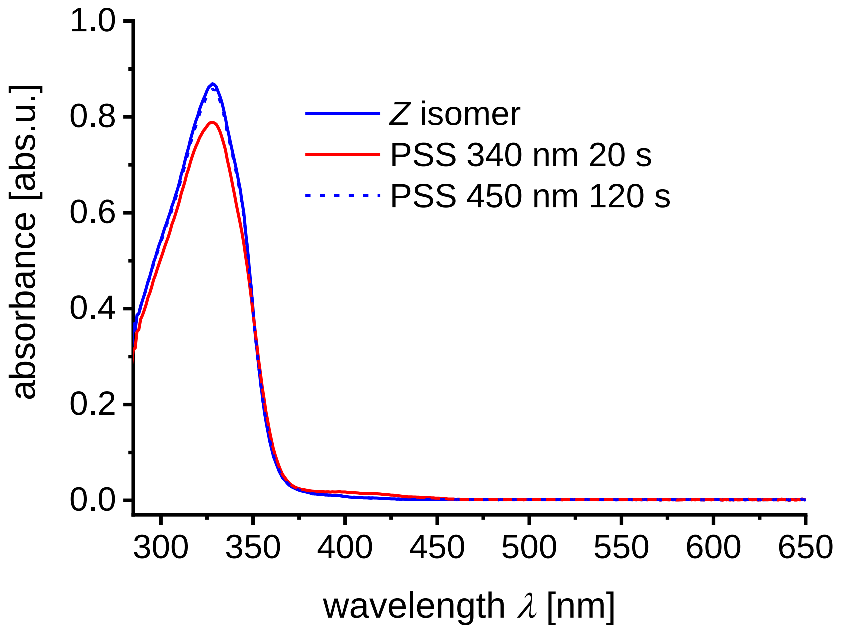

b)

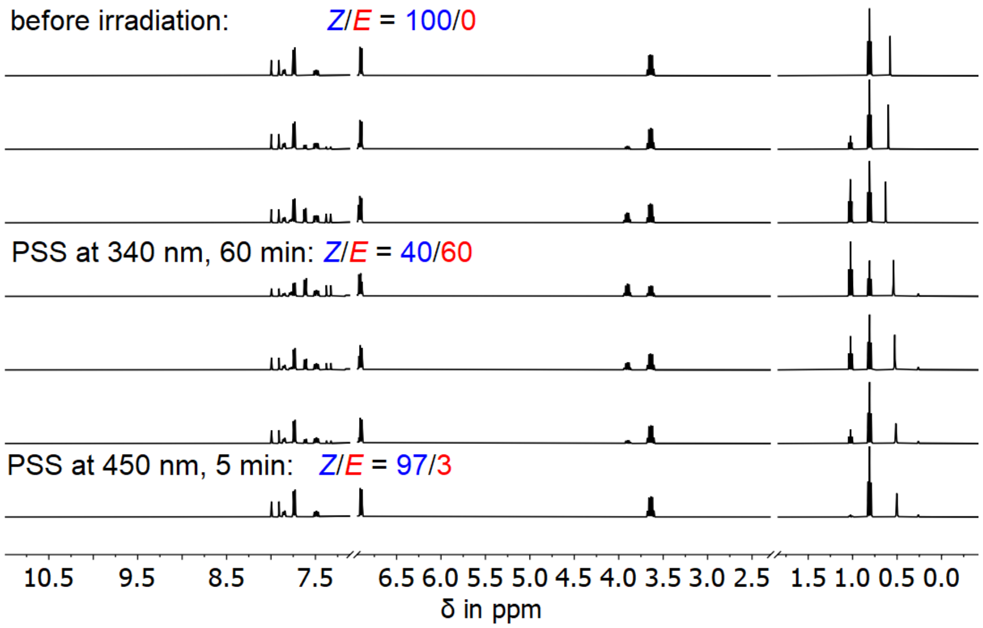

Supplementary Figure 80. Photoconversion of HPI **12** upon irradiation with 340 nm and 450 nm light. a) UV/Vis absorption spectra of HPI **12** in toluene at 23 °C recorded before (blue) and after irradiation with 340 nm (red) and 450 nm (blue dashed) light. The spectra after irradiation were recorded after reaching the PSS. b) ^{1}H NMR spectra (400 MHz, toluene-d_8, 23 °C) of a different sample of HPI **12** recorded before and after irradiation using 340 nm and 450 nm light. The NMR spectra are shown in the order of the experiments from top to bottom and isomeric composition of *Z* and *E* isomer at PSS are indicated. Unlabeled NMR spectra illustrate the isomer enrichment before reaching the PSS.

1.1.44 Photoisomerization of HPI 13

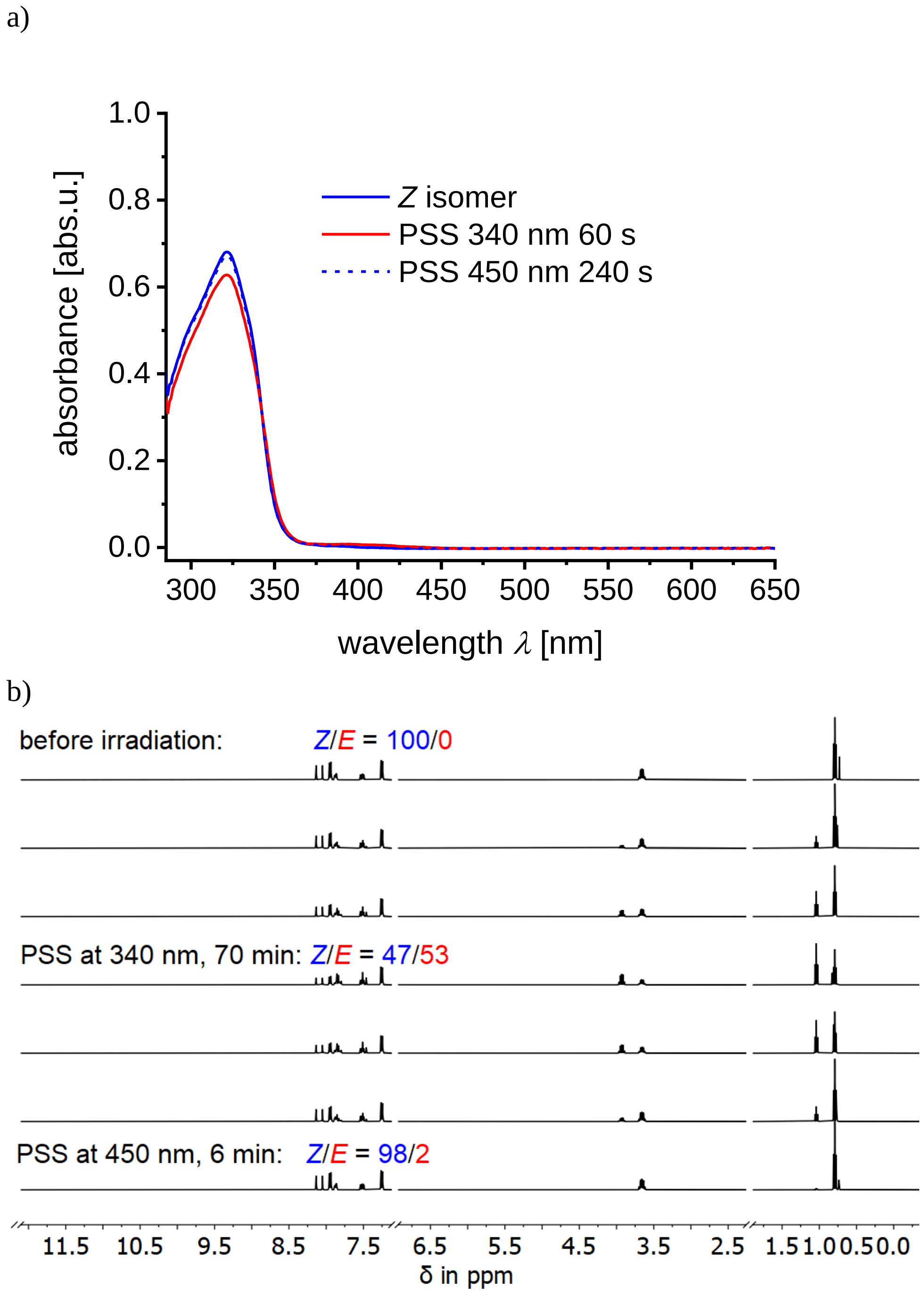

Supplementary Figure 81. Photoconversion of HPI **13** upon irradiation with 340 nm and 450 nm light. a) UV/Vis absorption spectra of HPI **13** in toluene at 23 °C recorded before (blue) and after irradiation with 340 nm (red) and 450 nm (blue dashed) light. The spectra after irradiation were recorded after reaching the PSS. b) ^{1}H NMR spectra (400 MHz, toluene-d_8, 23 °C) of a different sample of HPI **13** recorded before and after irradiation using 340 nm and 450 nm light. The NMR spectra are shown in the order of the experiments from top to bottom and isomeric composition of *Z* and *E* isomer at PSS are indicated. Unlabeled NMR spectra illustrate the isomer enrichment before reaching the PSS.

1.1.45 Photoisomerization of HPI 14

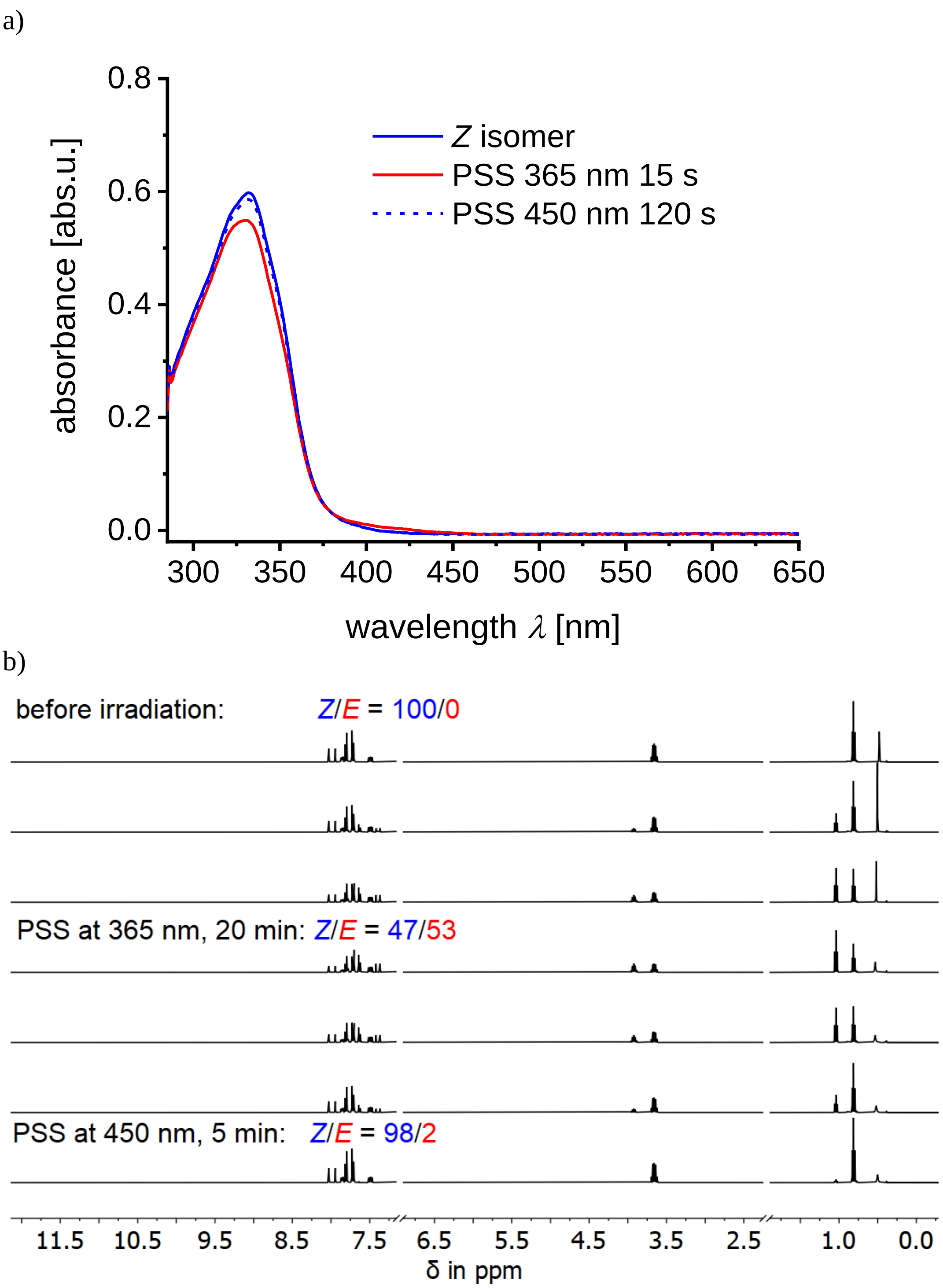

Supplementary Figure 82. Photoconversion of HPI **14** upon irradiation with 365 nm and 450 nm light. a) UV/Vis absorption spectra of HPI **14** in toluene at 23 °C recorded before (blue) and after irradiation with 365 nm (red) and 450 nm (blue dashed) light. The spectra after irradiation were recorded after reaching the PSS. b) ^{1}H NMR spectra (400 MHz, toluene-d_8, 23 °C) of a different sample of HPI **14** recorded before and after irradiation using 365 nm and 450 nm light. The NMR spectra are shown in the order of the experiments from top to bottom and isomeric composition of *Z* and *E* isomer at PSS are indicated. Unlabeled NMR spectra illustrate the isomer enrichment before reaching the PSS.

1.1.46 Photoisomerization of HPI 15

a)

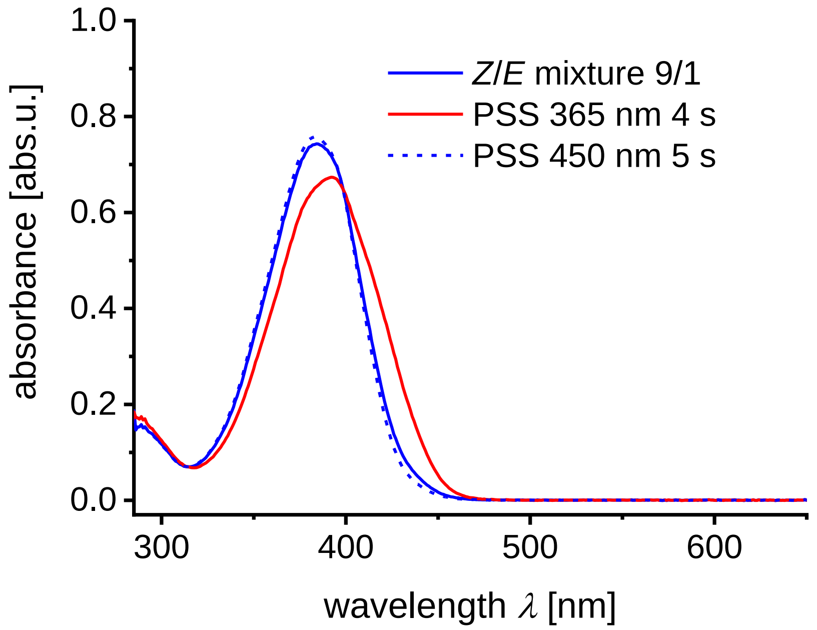

b)

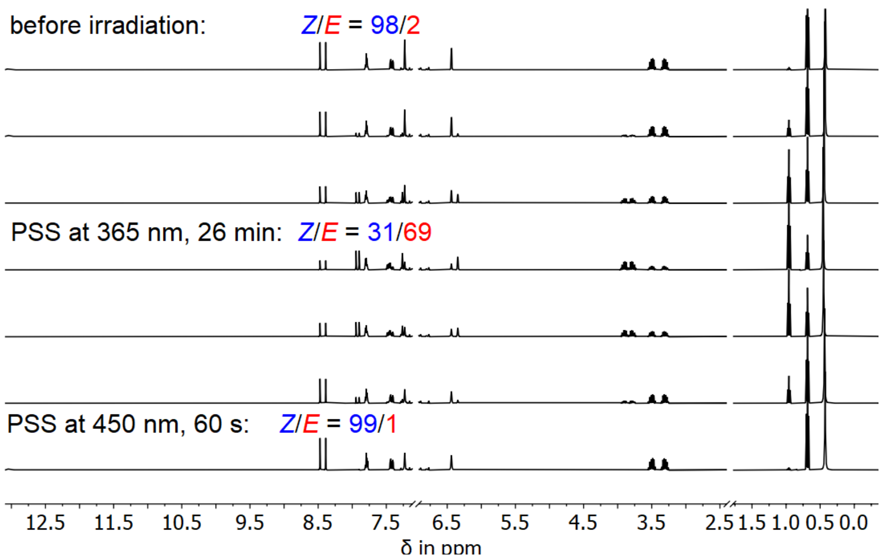

Supplementary Figure 83. Photoconversion of HPI **15** upon irradiation with 365 nm and 450 nm light. a) UV/Vis absorption spectra of HPI **15** in toluene at 23 °C recorded before (blue) and after irradiation with 365 nm (red) and 450 nm (blue dashed) light. The spectra after irradiation were recorded after reaching the PSS. b) ^{1}H NMR spectra (400 MHz, toluene-d_8, 23 °C) of a different sample of HPI **15** recorded before and after irradiation using 365 nm and 450 nm light. The NMR spectra are shown in the order of the experiments from top to bottom and isomeric composition of *Z* and *E* isomer at PSS are indicated. Unlabeled NMR spectra illustrate the isomer enrichment before reaching the PSS.

1.1.47 Photoisomerization of HPI 16

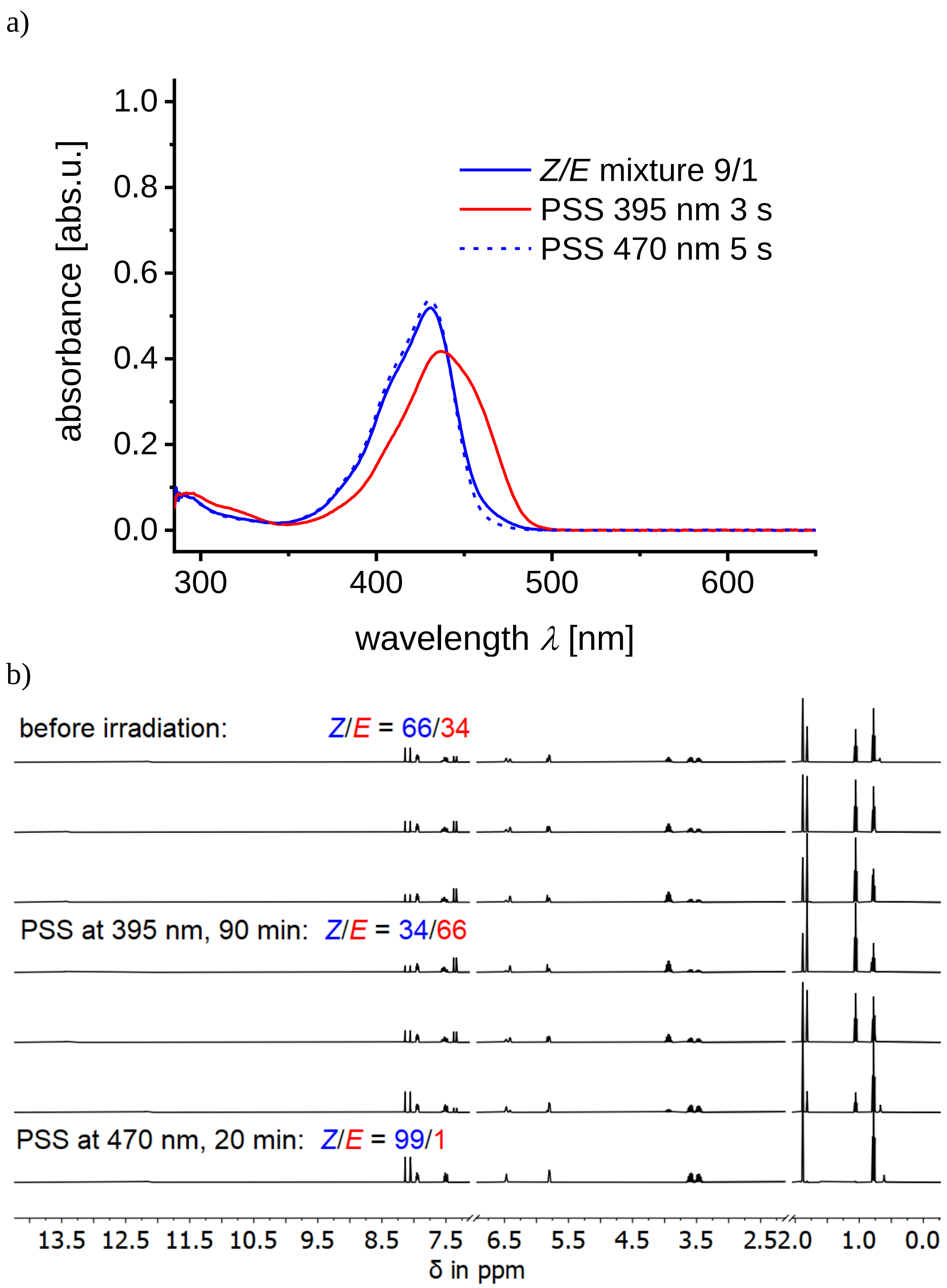

Supplementary Figure 84. Photoconversion of HPI **16** upon irradiation with 395 nm and 470 nm light. a) UV/Vis absorption spectra of HPI **16** in toluene at 23 °C recorded before (blue) and after irradiation with 395 nm (red) and 470 nm (blue dashed) light. The spectra after irradiation were recorded after reaching the PSS. b) ^{1}H NMR spectra (400 MHz, toluene-d_8, 23 °C) of a different sample of HPI **16** recorded before and after irradiation using 395 nm and 470 nm light. The NMR spectra are shown in the order of the experiments from top to bottom and isomeric composition of *Z* and *E* isomer at PSS are indicated. Unlabeled NMR spectra illustrate the isomer enrichment before reaching the PSS.

1.1.48 Photoisomerization of HPI 17

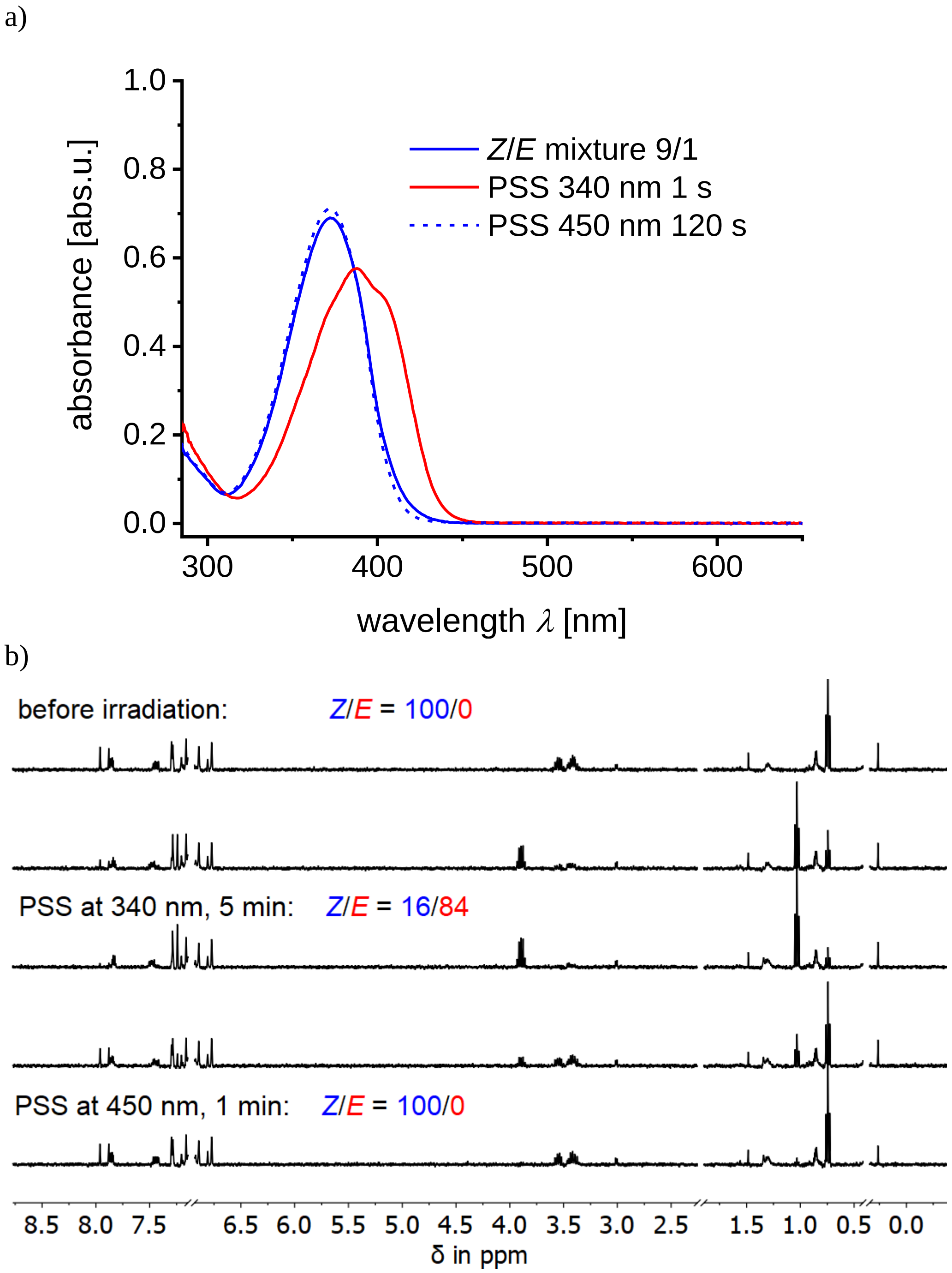

Supplementary Figure 85. Photoconversion of HPI **17** upon irradiation with 340 nm and 450 nm light. a) UV/Vis absorption spectra of HPI **17** in toluene at 23 °C recorded before (blue) and after irradiation with 340 nm (red) and 450 nm (blue dashed). The spectra after irradiation were recorded after reaching the PSS. b) ^{1}H NMR spectra (400 MHz, toluene-d_8, 23 °C) of a different sample of HPI **17** recorded before and after irradiation using 340 nm and 450 nm light. The NMR spectra are shown in the order of the experiments from top to bottom and isomeric composition of *Z* and *E* isomer at PSS are indicated. Unlabeled NMR spectra illustrate the isomer enrichment before reaching the PSS.

1.1.49 Photoisomerization of HPI 18

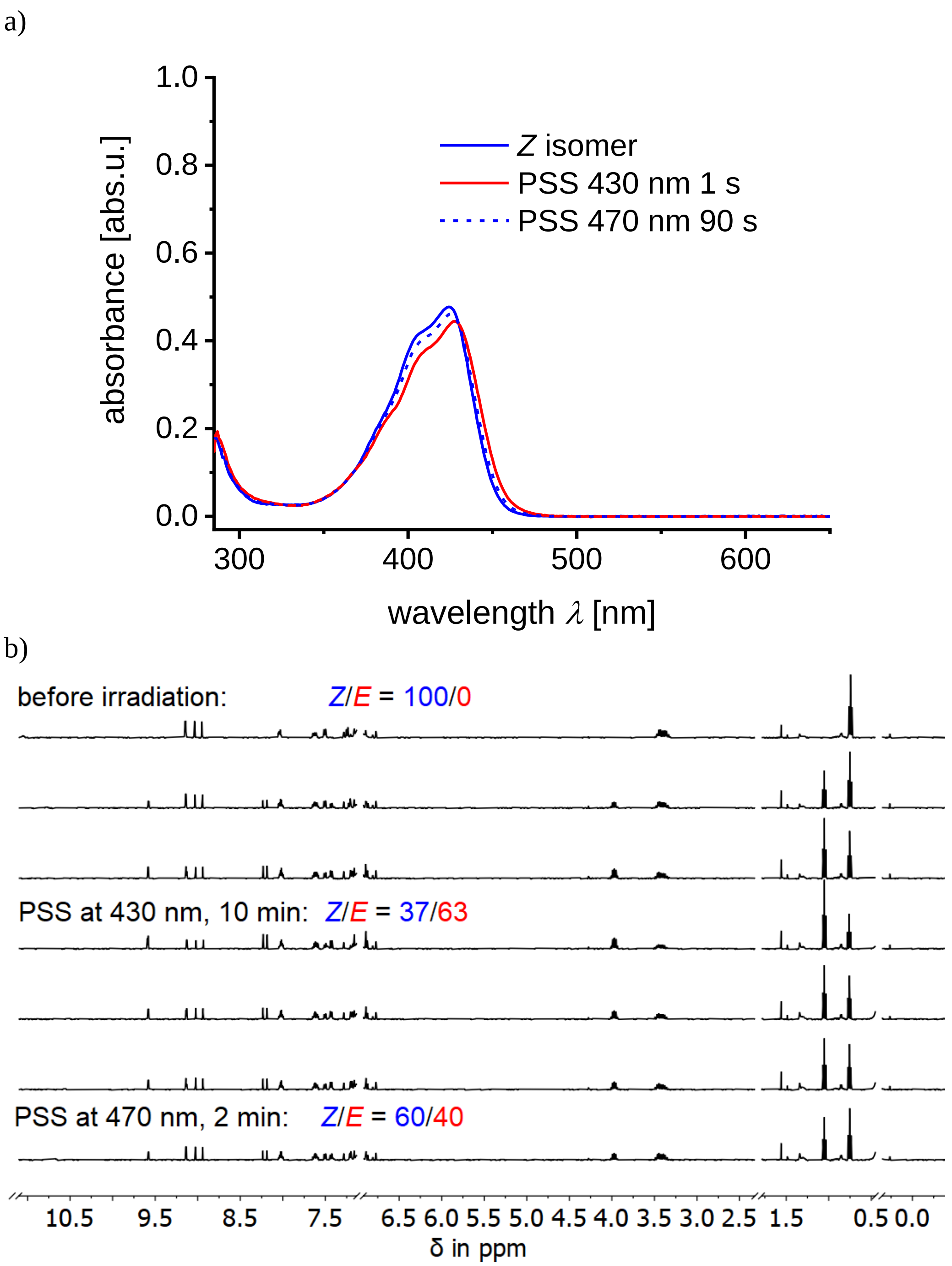

Supplementary Figure 86. Photoconversion of HPI **18** upon irradiation with 430 nm and 470 nm light. a) UV/Vis absorption spectra of HPI **18** in toluene at 23 °C recorded before (blue) and after irradiation with 430 nm (red) and 470 nm (blue dashed) light. The spectra after irradiation were recorded after reaching the PSS. b) ^{1}H NMR spectra (400 MHz, toluene-d_8, 23 °C) of a different sample of HPI **18** recorded before and after irradiation using 430 nm and 470 nm light. The NMR spectra are shown in the order of the experiments from top to bottom and isomeric composition of *Z* and *E* isomer at PSS are indicated. Unlabeled NMR spectra illustrate the isomer enrichment before reaching the PSS.

Supplementary Note 9: Photoisomerization reactions in water

For all derivatives, photoswitching in pure water has been investigated using UV/Vis spectroscopy. Saturated solutions have been prepared and were filtered before use. The absorption spectra of the saturated solutions were used for a qualitative comparison of the water solubility and selected derivatives were further analyzed via ^{1}H NMR spectroscopy.

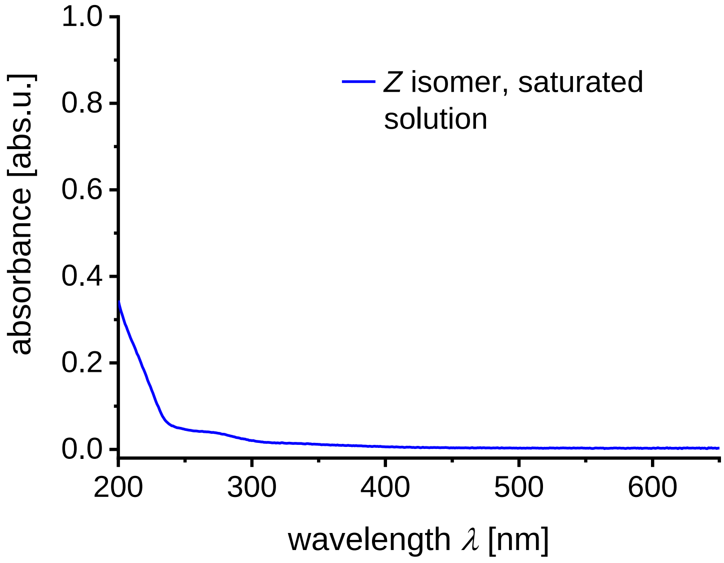

Supplementary Figure 87. UV/Vis absorption spectrum of a saturated solution of HPI **1** in water at 23 °C.

Comment: HPI **1** does not dissolve well enough in water and no spectral change occurs upon irradiation with 280 nm, 300 nm, 340 nm and 365 nm.

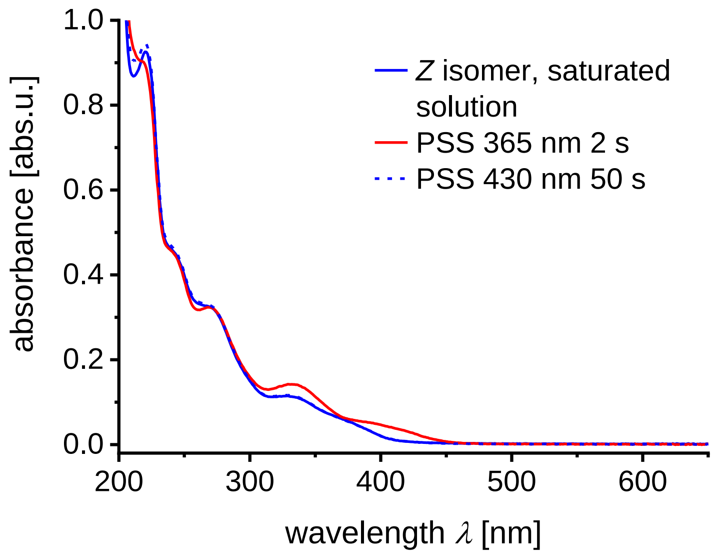

Supplementary Figure 88. UV/Vis absorption spectrum of a saturated solution of HPI **2** in water at 23 °C. Photoconversion of HPI **2** upon irradiation with 365 nm and 430 nm light. The spectra were recorded before (blue) and after irradiation with 365 nm (red) and 430 nm (blue dashed) light. The spectra after irradiation were recorded after reaching the PSS.

Comment: HPI **2** does not dissolve well enough for analysis via ^{1}H NMR spectroscopy.

a)

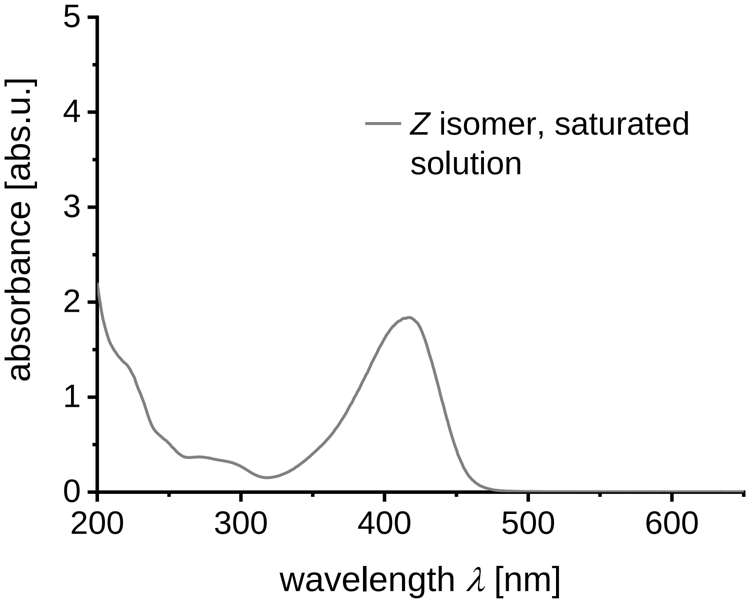

b)

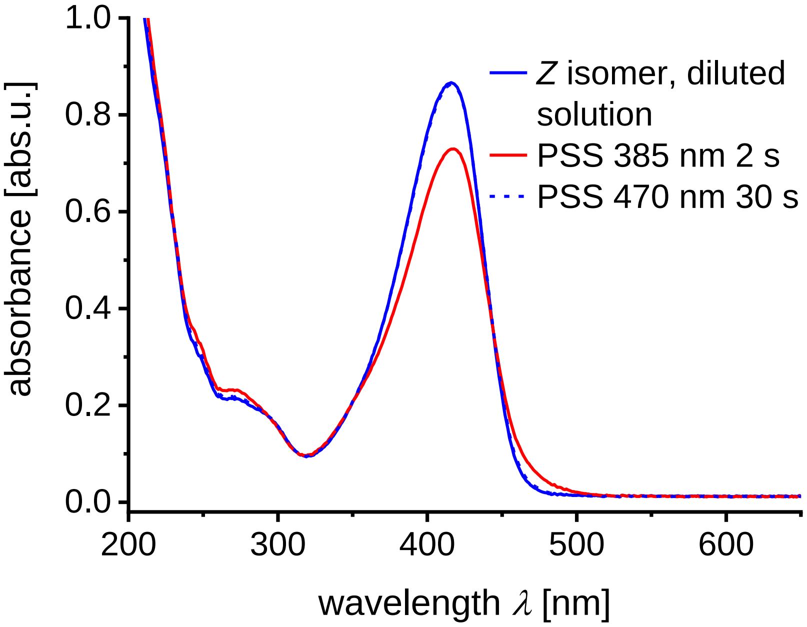

Supplementary Figure 89. a) UV/Vis absorption spectrum of a saturated solution of HPI **3** in water at 23 °C. b) Photoconversion of HPI **3** upon irradiation with 385 nm and 470 nm light in water 23 °C. The spectra were recorded before (blue) and after irradiation with 385 nm (red) and 470 nm (blue dashed) light. The spectra after irradiation were recorded after reaching the PSS.

Comment: Thermal back isomerization of HPI **3** is too fast for analysis via ^{1}H NMR spectroscopy.

a)

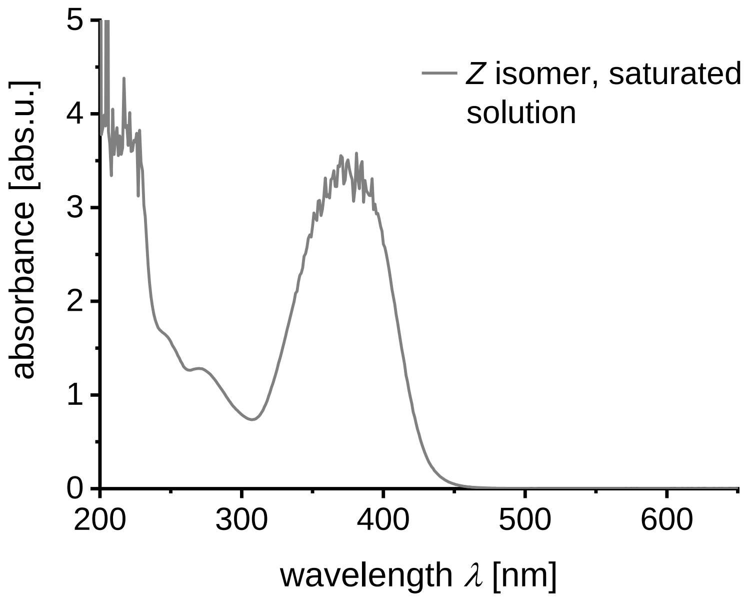

b)

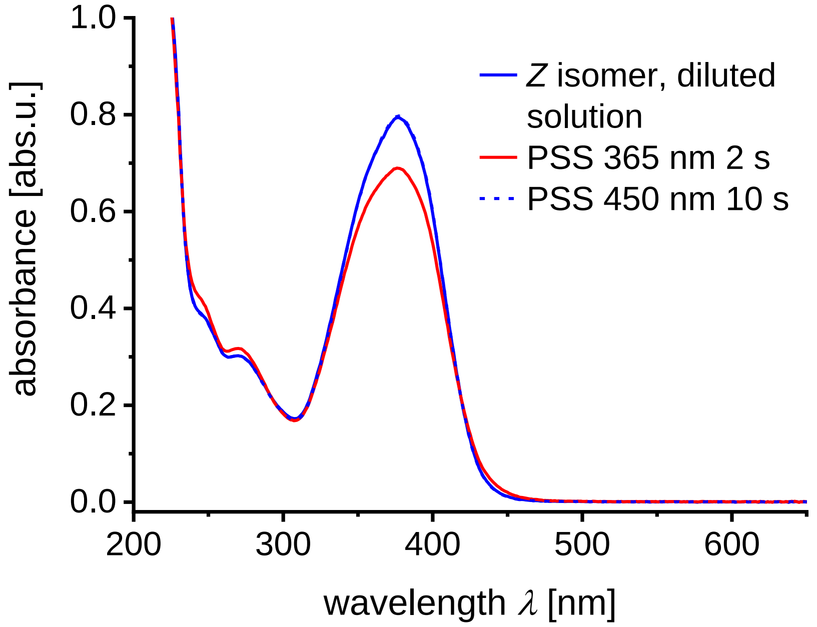

Supplementary Figure 90. a) UV/Vis absorption spectrum of a saturated solution of HPI **4** in water at 23 °C. b) Photoconversion of HPI **4** upon irradiation with 365 nm and 450 nm light in water 23 °C. The spectra were recorded before (blue) and after irradiation with 365 nm (red) and 450 nm (blue dashed) light. The spectra after irradiation were recorded after reaching the PSS.

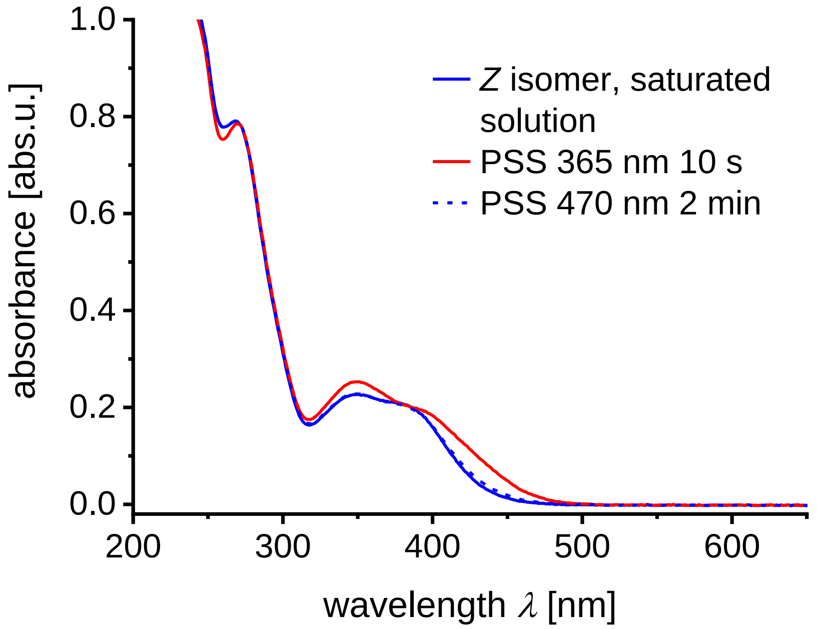

Supplementary Figure 91. UV/Vis absorption spectrum of a saturated solution of HPI **5** in water at 23 °C. Photoconversion of HPI **5** upon irradiation with 365 nm and 470 nm light. The spectra were recorded before (blue) and after irradiation with 365 nm (red) and 470 nm (blue dashed) light. The spectra after irradiation were recorded after reaching the PSS.

Comment: HPI **5** does not dissolve well enough for analysis via ^{1}H NMR spectroscopy.

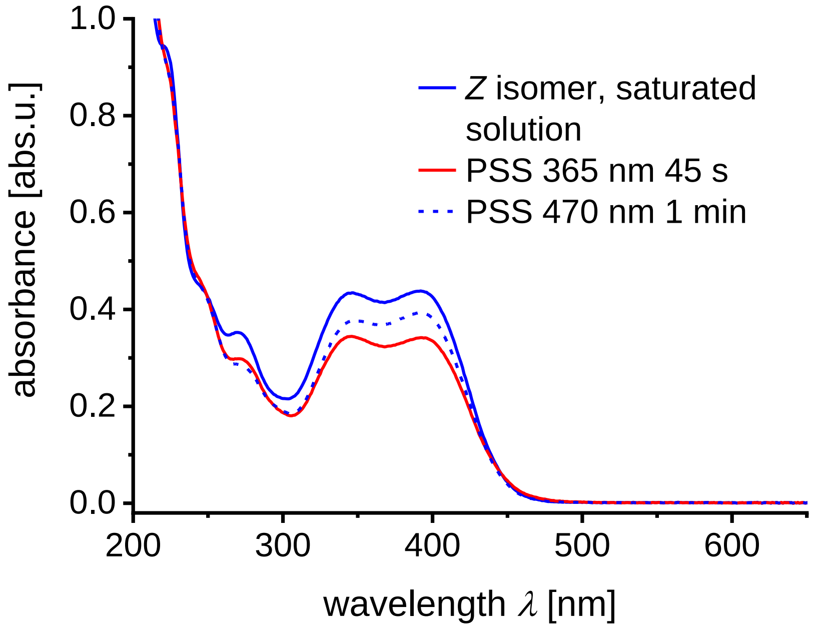

Supplementary Figure 92. UV/Vis absorption spectrum of a saturated solution of HPI **6** in water at 23 °C. Photoconversion of HPI **6** upon irradiation with 365 nm and 470 nm light. The spectra were recorded before (blue) and after irradiation with 365 nm (red) and 470 nm (blue dashed) light. The spectra after irradiation were recorded after reaching the PSS.

Comment: HPI **6** does not dissolve well enough for analysis via ^{1}H NMR spectroscopy.

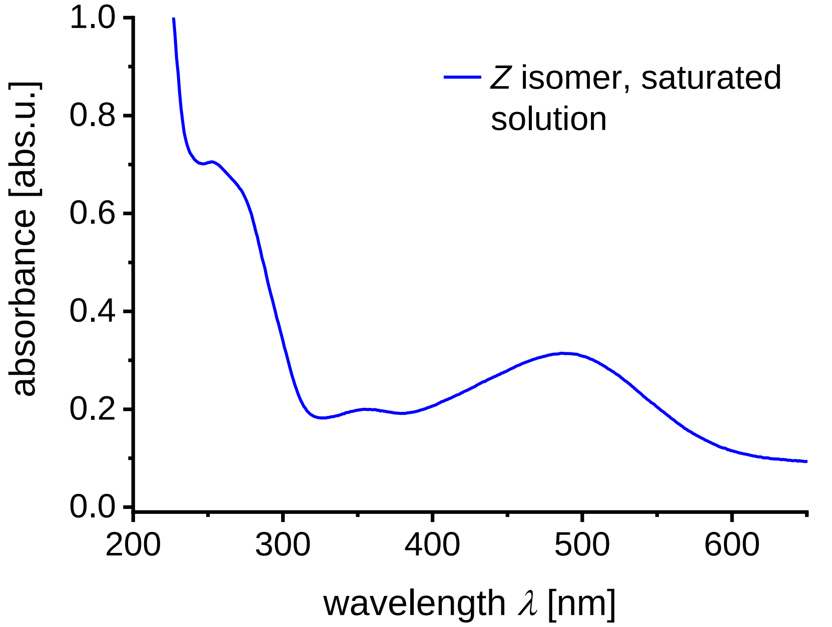

Supplementary Figure 93. UV/Vis absorption spectrum of a saturated solution of HPI **7** in water at 23 °C.

Comment: Shows strong fluorescence and no photoswitching upon irradiation with 300, 385, 395, 405, 420, 430, 450, 470, 515, 530, 565, 595 and 625 nm probably due to ICT (intramolecular charge transfer) effects

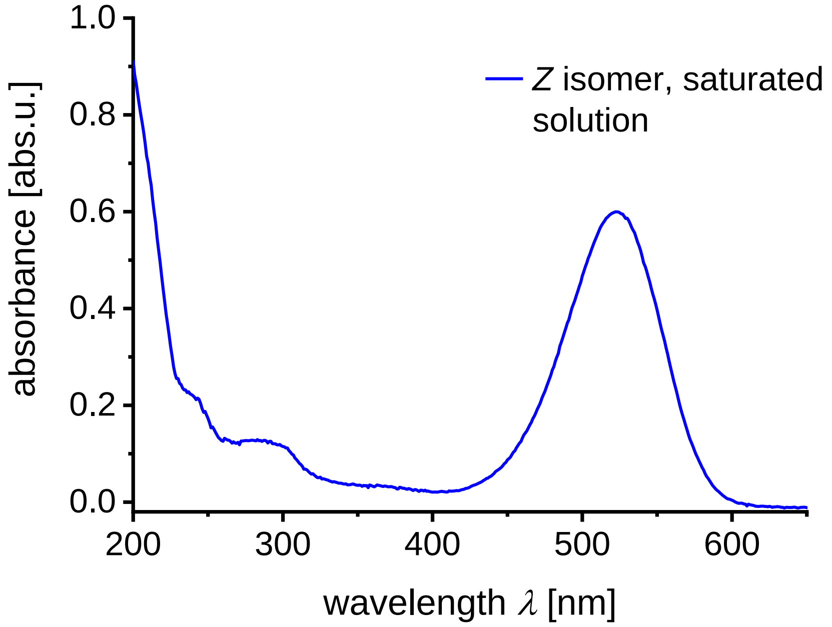

Supplementary Figure 94. UV/Vis absorption spectrum of a saturated solution of HPI **8** in water at 23 °C.

Comment: Shows strong fluorescence and no photoswitching upon irradiation with 420, 430, 450, 470, 515, 530, 565, 595 and 625 nm.

a)

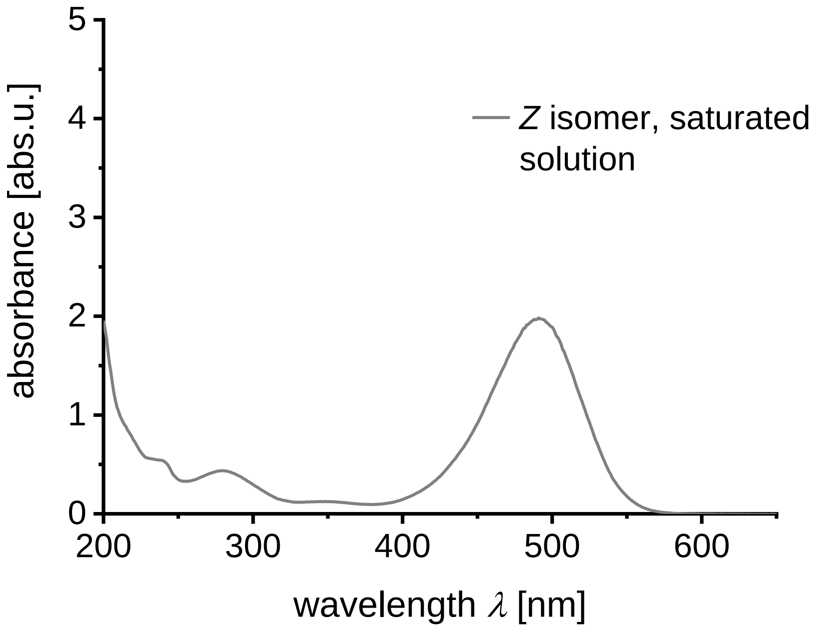

b)

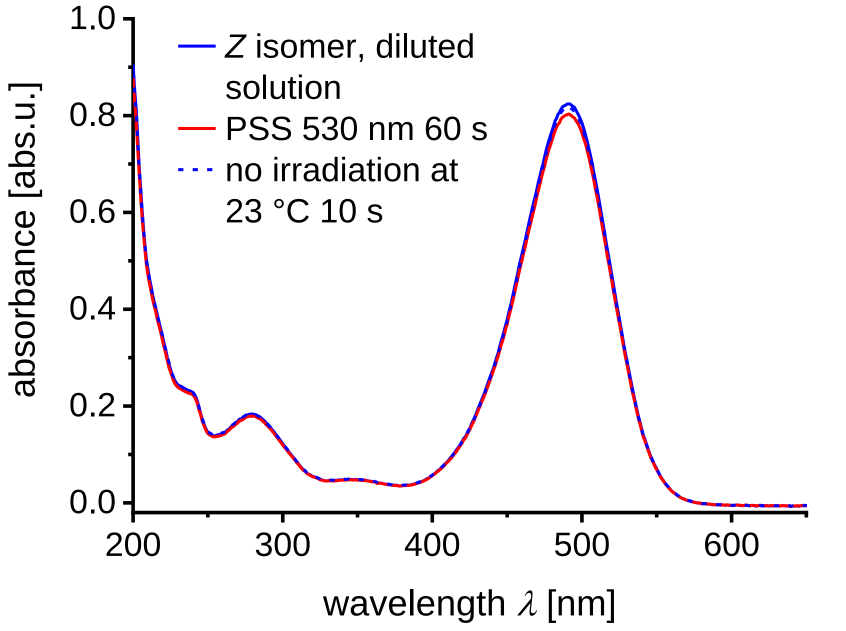

Supplementary Figure 95. a) UV/Vis absorption spectrum of a saturated solution of HPI **9** in water at 23 °C. b) Photoconversion of HPI **9** upon irradiation with 530 nm light in water 23 °C. The spectra were recorded before (blue) and after irradiation with 530 nm (red). The spectrum after irradiation was recorded after reaching the PSS.

Comment: Thermal back isomerization is very fast, not possible to determine the PSS for the *E* to *Z* reaction.

a)

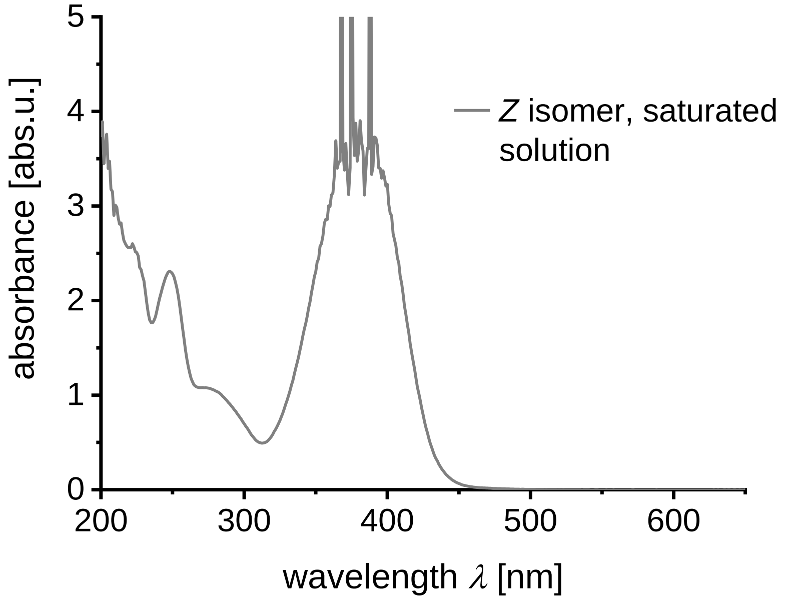

b)

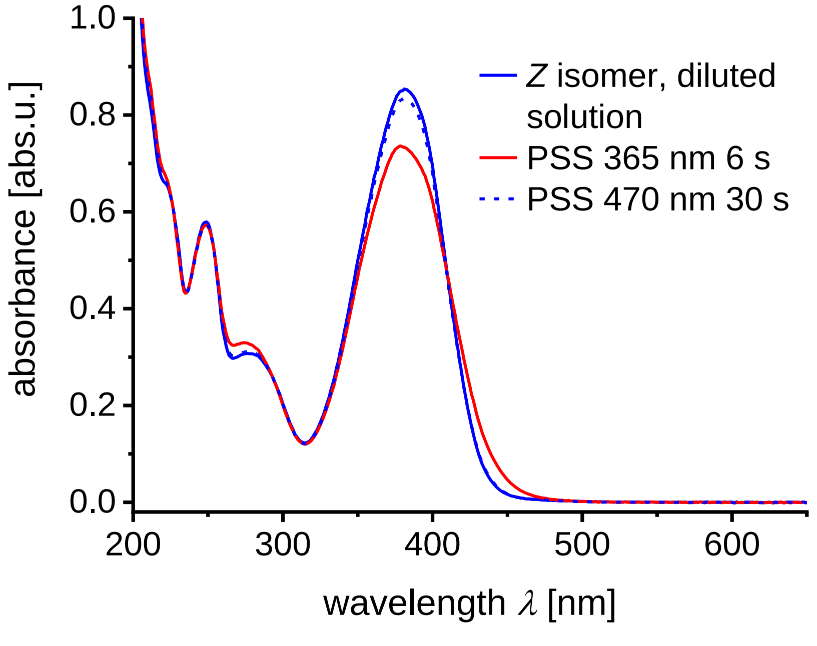

Supplementary Figure 96. a) UV/Vis absorption spectrum of a saturated solution of HPI **10** in water at 23 °C. b) Photoconversion of HPI **10** upon irradiation with 365 nm and 470 nm light in water 23 °C. The spectra were recorded before (blue) and after irradiation with 365 nm (red) and 470 nm (blue dashed) light. The spectrum after irradiation was recorded after reaching the PSS.

a)

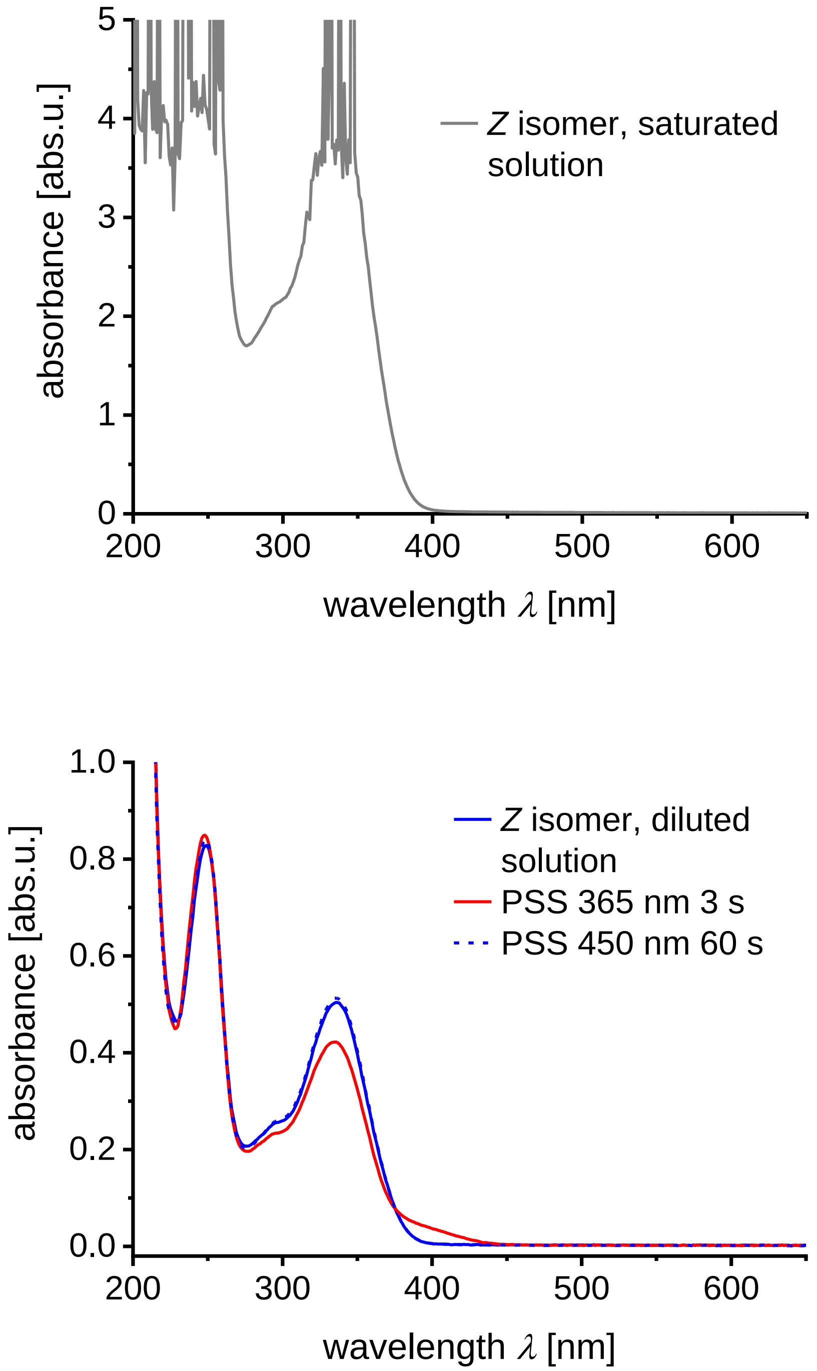

Supplementary Figure 97. a) UV/Vis absorption spectrum of a saturated solution of HPI **11** in water at 23 °C. b) Photoconversion of HPI **11** upon irradiation with 365 nm and 450 nm light in water 23 °C. The spectra were recorded before (blue) and after irradiation with 365 nm (red) and 450 nm (blue dashed) light. The spectrum after irradiation was recorded after reaching the PSS.

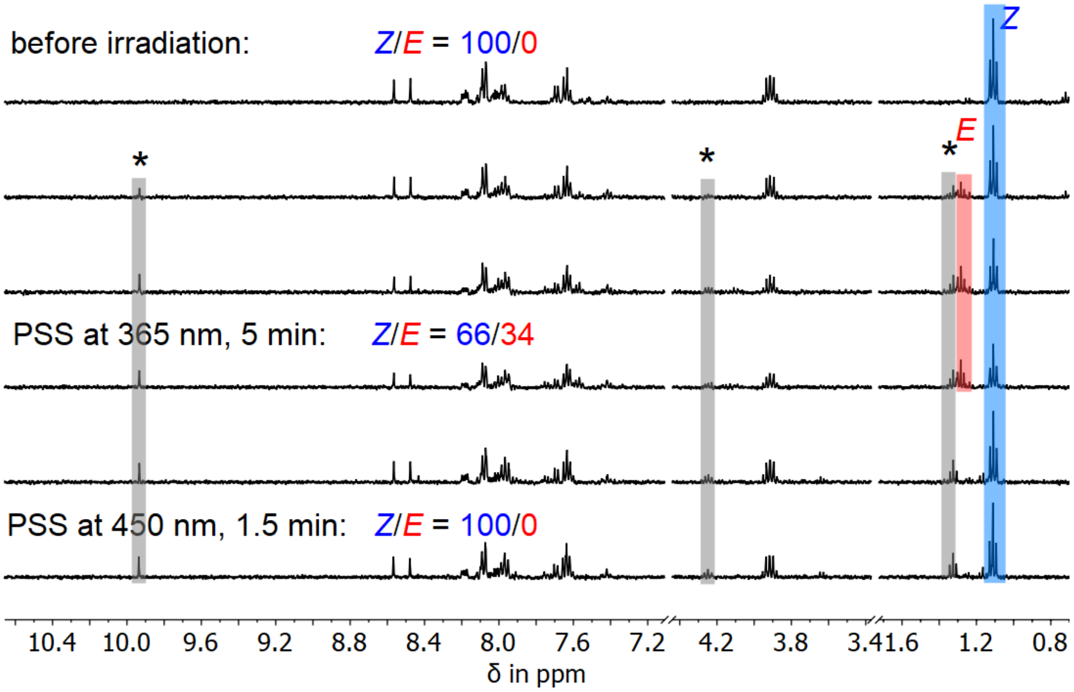

Supplementary Figure 98. Photoconversion of HPI **11** upon irradiation with 365 nm and 450 nm light followed by ^{1}H NMR spectroscopy (400 MHz, D_2O, 23 °C. The NMR spectra are shown in the order of the experiments from top to bottom and isomeric composition of *Z* and *E* isomer at PSS are indicated. Characteristic signals for both isomers are labeled in blue (*Z* isomer) and red (*E* isomer). Photodegradation is shown in grey and labeled with a star.

Comment: Most probably, fast thermal E to Z isomerization takes place leading to the need of prolonged irradiation times at NMR concentrations. The longer irradiation leads to visible photodegradation and reduced photoswitching performance under the higher concentrations.

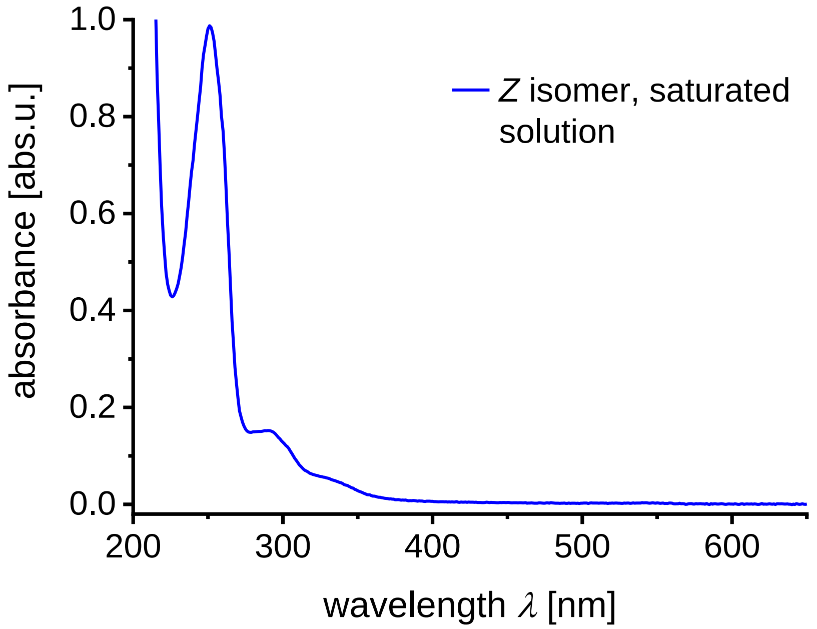

Supplementary Figure 99. UV/Vis absorption spectrum of a saturated solution of HPI **12** in water at 23 °C.

Comment: HPI **12** is not light addressable.

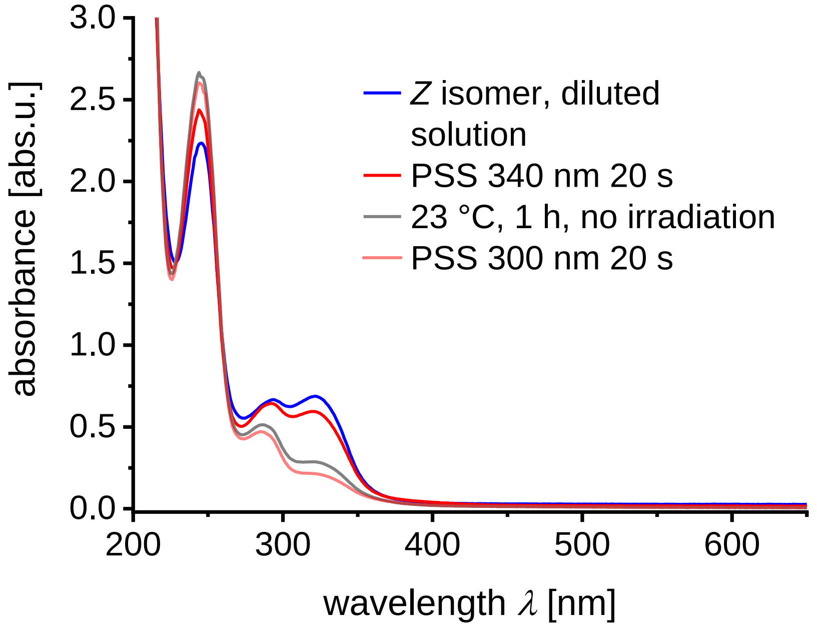

Supplementary Figure 100. UV/Vis absorption spectrum of a saturated solution of HPI **13** in water at 23 °C. Photoconversion of HPI **13** upon irradiation with 340 nm and 300 nm light. The spectra were recorded before (blue) and after irradiation with 340 nm (red) and 300 nm (light red) light. The spectra after irradiation were recorded after reaching the PSS. In between the measurements, the sample was not irradiated for 1 h, in order to showcase a thermal reaction occurring.

Comment: In this case photodegradation rather than productive photoisomerization occurs.

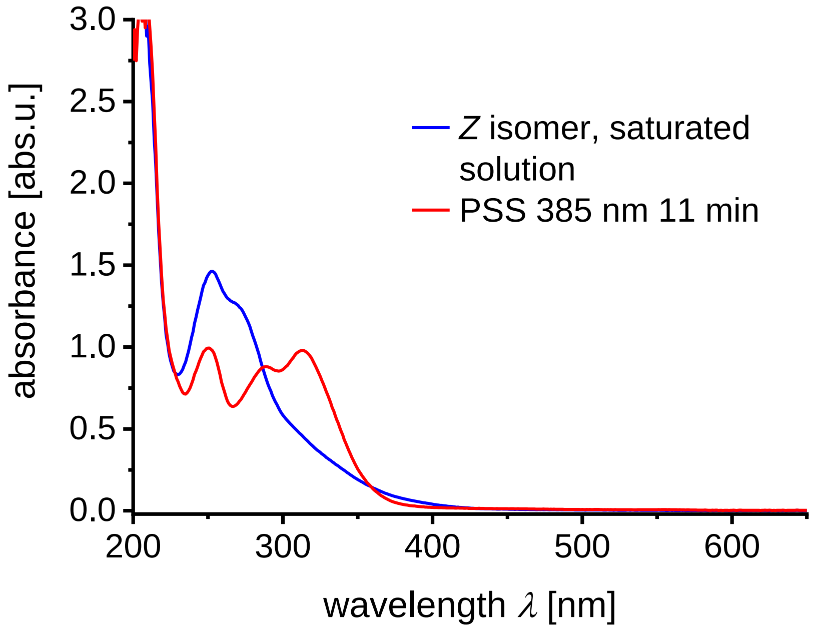

Supplementary Figure 101. UV/Vis absorption spectrum of a saturated solution of HPI **14** in water at 23 °C. Photoconversion of HPI **14** upon irradiation with 385 nm. The spectra were recorded before (blue) and after irradiation with 385 nm (red) light. The spectrum after irradiation was recorded after reaching the PSS.

Comment: The absorption spectrum after irradiation strongly differs from the one of the *Z* isomer, which is unusual for the planar HPIs in this study. It is more likely to assume that a different photoreaction than *Z* to *E* isomerization takes place and is supported by the fact that it is irreversible.

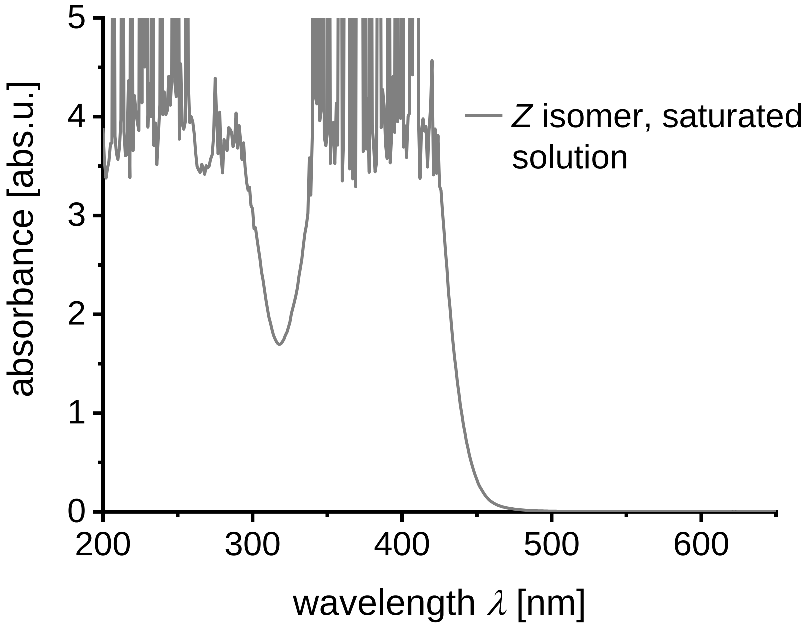

Supplementary Figure 102. UV/Vis absorption spectrum of a saturated solution of HPI **15** in water at 23 °C.

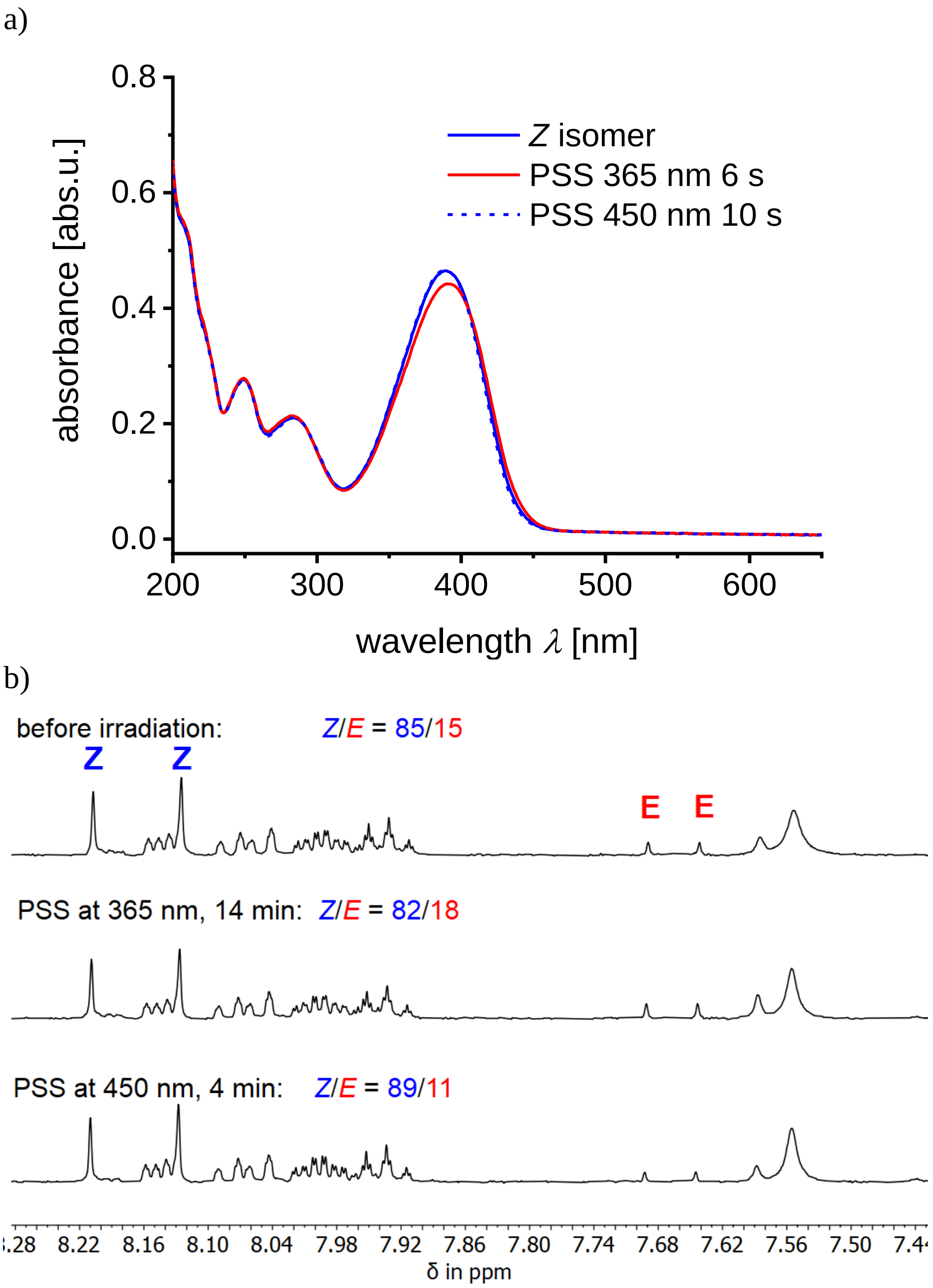

Supplementary Figure 103. Photoconversion of HPI **15** upon irradiation with 365 nm and 450 nm light. a) UV/Vis absorption spectra of HPI **15** in water at 23 °C recorded before (blue) and after irradiation with 365 nm (red) and 450 nm (blue dashed) light. The spectra after irradiation were recorded after reaching the PSS. b) ^{1}H NMR spectra (400 MHz, D_2O, 23 °C) of a different sample of HPI **15** recorded before and after irradiation using 365 nm and 450 nm light. The NMR spectra are shown in the order of the experiments from top to bottom and isomeric composition of *Z* and *E* isomer at PSS are indicated. The characteristic signal for the proton at the central double bond is labeled for both isomers.

a)

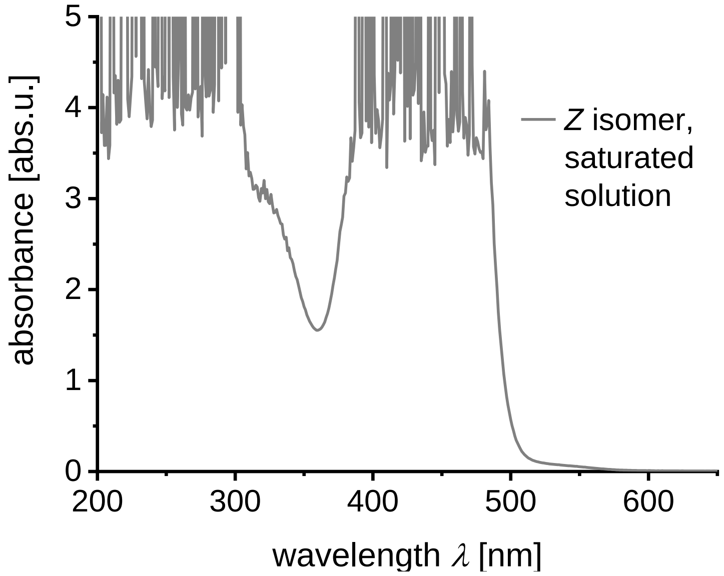

b)

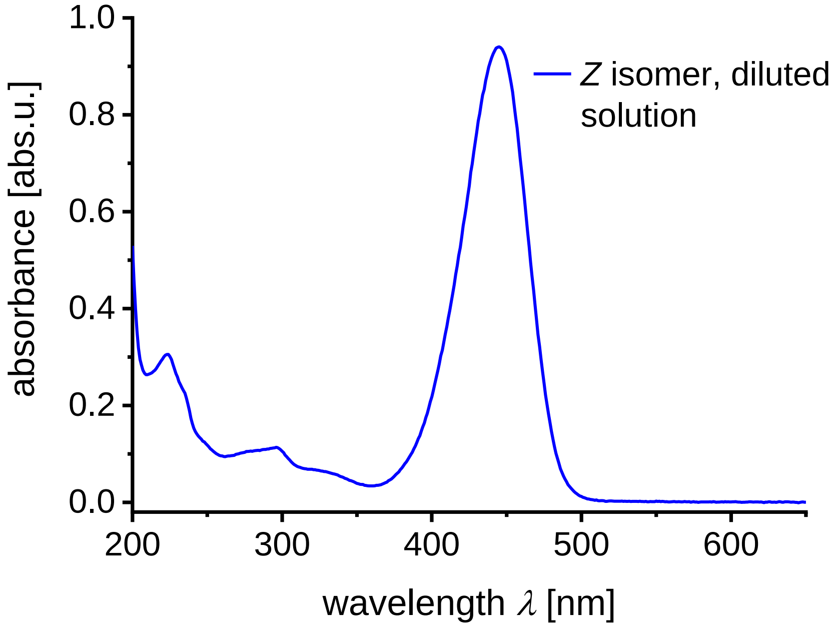

Supplementary Figure 104. a) UV/Vis absorption spectrum of a saturated solution of HPI **16** in water at 23 °C. b) UV/Vis absorption spectrum of a diluted solution of HPI **16** in water at 23 °C.

Comment: Fluorescence observable, HPI **16** is not light addressable, no change occurs upon irradiation with 365, 385, 395, 405, 420, 430, 450, 470, 490, 505, 515 and 530 nm light.

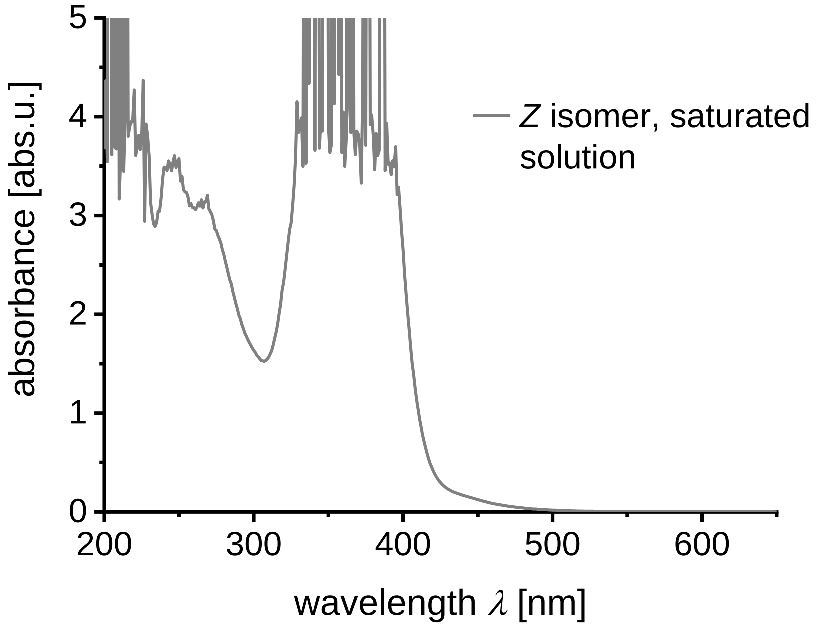

Supplementary Figure 105. UV/Vis absorption spectrum of a saturated solution of HPI **17** in water at 23 °C.

a)

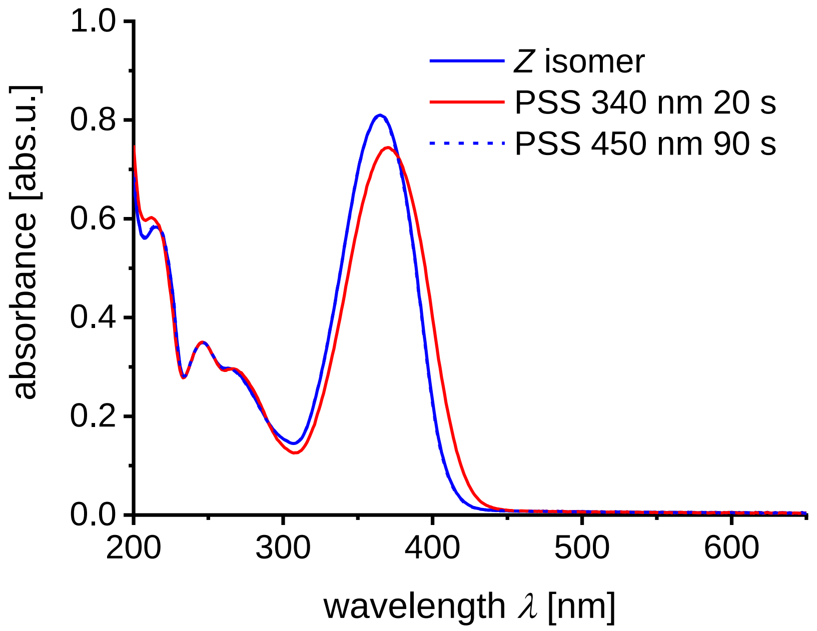

b)

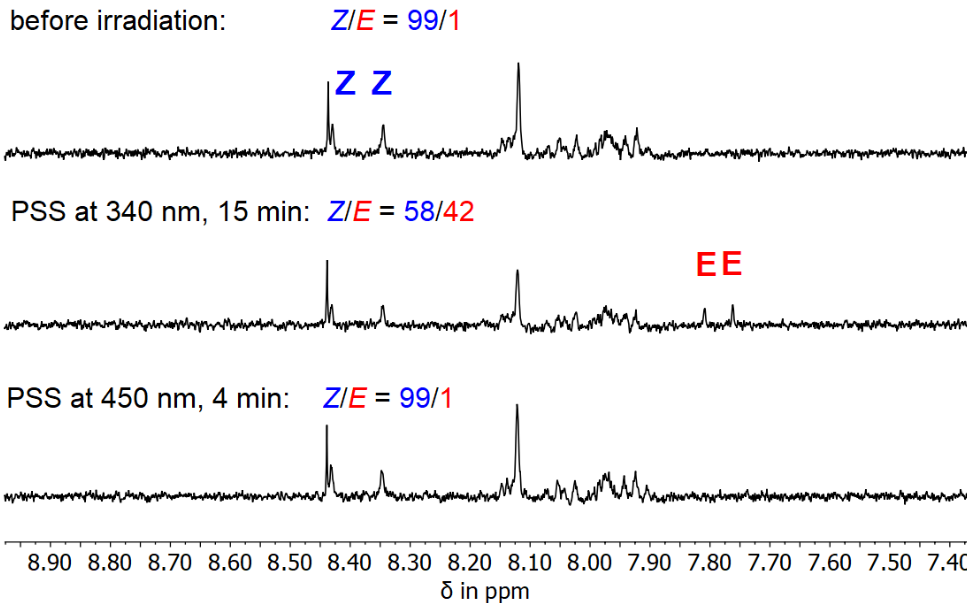

Supplementary Figure 106. Photoconversion of HPI **17** upon irradiation with 340 nm and 450 nm light. a) UV/Vis absorption spectra of HPI **17** in water at 23 °C recorded before (blue) and after irradiation with 340 nm (red) and 450 nm (blue dashed). The spectra after irradiation were recorded after reaching the PSS. b) ^{1}H NMR spectra (400 MHz, D_2O, 23 °C) of a different sample of HPI **17** recorded before and after irradiation using 340 nm and 450 nm light. The NMR spectra are shown in the order of the experiments from top to bottom and isomeric composition of *Z* and *E* isomer at PSS are indicated. The characteristic signal for the proton at the central double bond is labeled for both isomers.

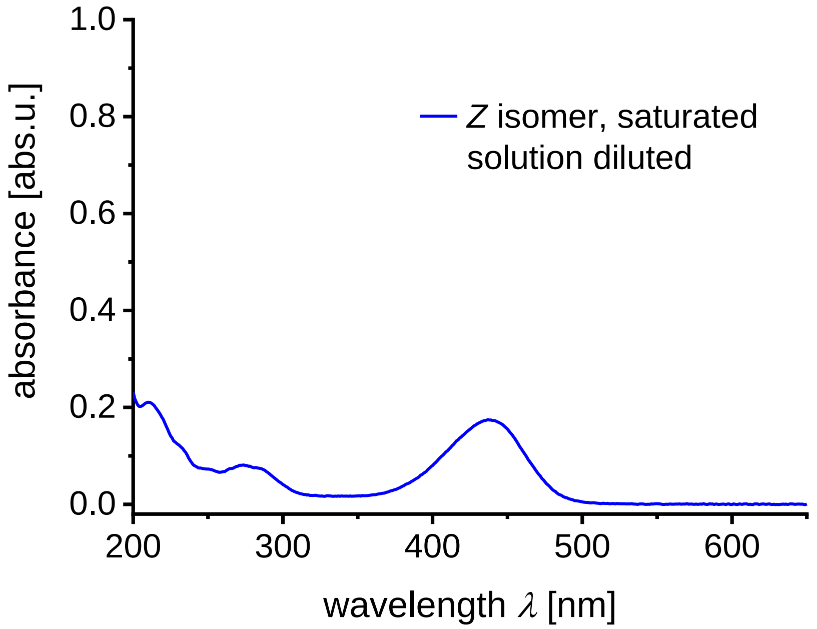

Supplementary Figure 107. UV/Vis absorption spectrum of a saturated solution of HPI **18** in water at 23 °C.

Comment: Fluorescence observable, HPI **18** is not light addressable, no change occurs upon irradiation with 365, 385, 395, 405, 420, 430, 450, 470, 490, 505, 515 and 530 nm light.

Supplementary Note 10: Photoisomerization reactions of hydrolyzed derivatives in water

Selected planar, twisted and heterocyclic HPIs (**1**, **2**, **3**, **11** and **17**) with good photoswitching properties were hydrolyzed to the corresponding phosphinic acids (**1**-OH, **2**-OH, **3**-OH, **11**-OH and **17**-OH) and photoswitching in water was investigated. For the difference in water solubility, see Supplementary Note 3. All derivatives were soluble enough for analysis via ^{1}H NMR spectroscopy. For the ^{1}H NMR measurements, saturated solutions were prepared that were filtered using a syringe filter before analysis.

a)

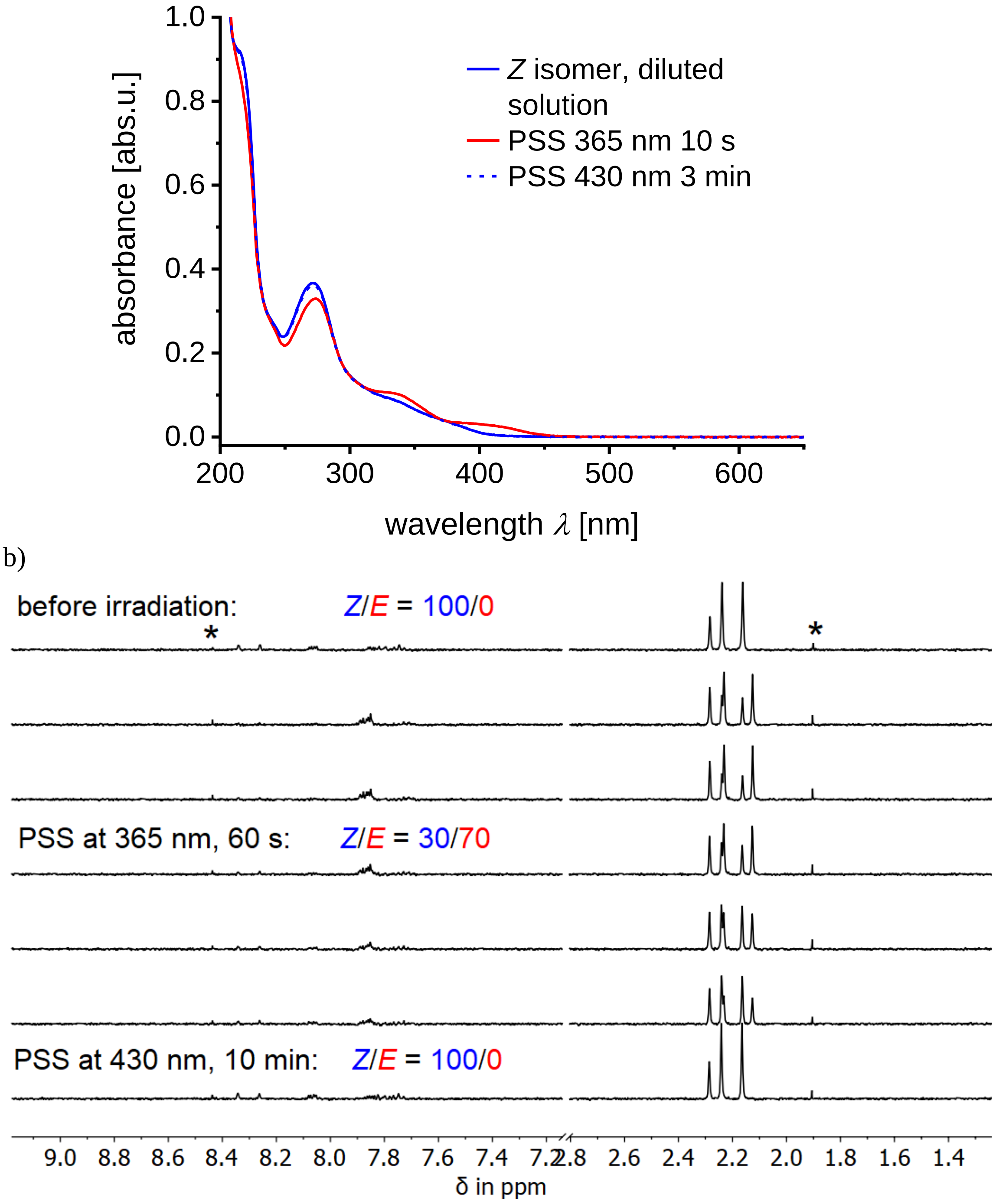

Supplementary Figure 108. Photoconversion of HPI **1**-OH upon irradiation with 365 nm and 430 nm light. a) UV/Vis absorption spectra of HPI **1**-OH in H_2O at 23 °C recorded before (blue) and after irradiation with 365 nm (red) and 430 nm (blue dashed) light. The spectra after irradiation were recorded after reaching the PSS. b) ^{1}H NMR spectra (400 MHz, D_2O, 23 °C) of a different sample of **1**-OH recorded before and after irradiation using 365 nm and 430 nm light. The NMR spectra are shown in the order of the experiments from top to bottom and isomeric composition of *Z* and *E* isomer at PSS are indicated. Unlabeled NMR spectra illustrate the isomer enrichment before reaching the PSS. Impurities originating from the syringe filter used for sample preparation are marked with a star.

a)

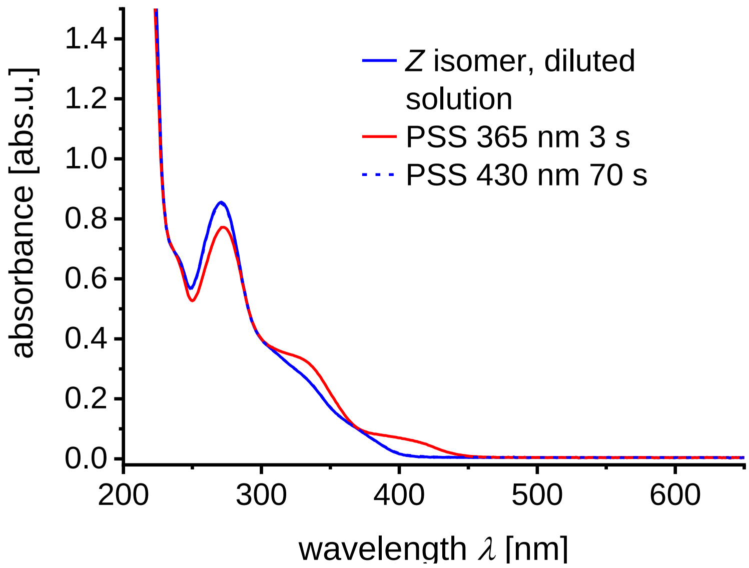

b)

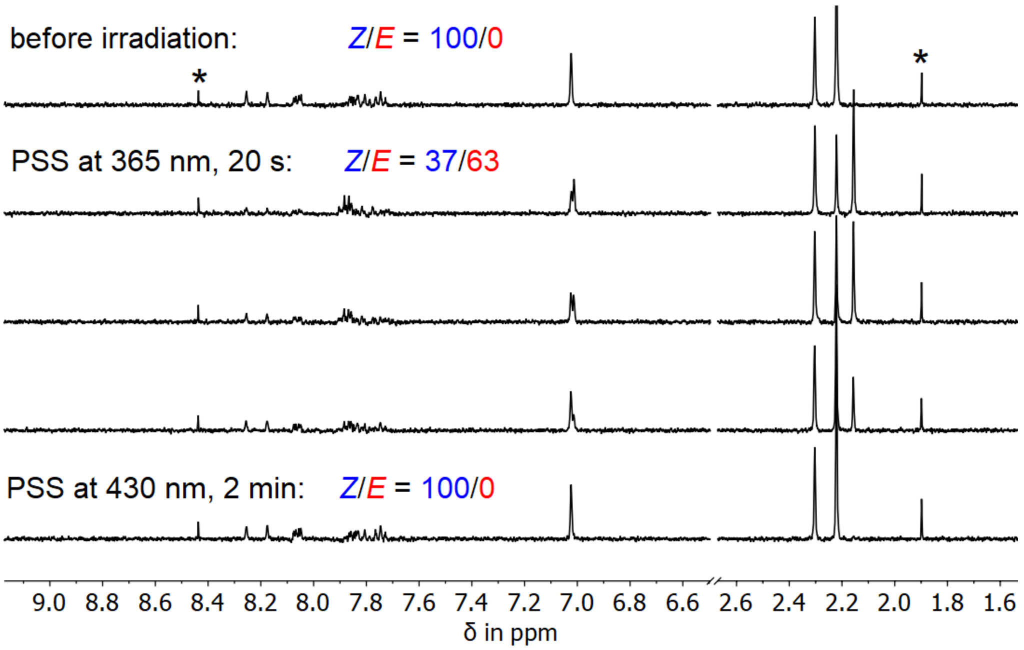

Supplementary Figure 109. Photoconversion of HPI **2**-OH upon irradiation with 365 nm and 430 nm light. a) UV/Vis absorption spectra of HPI **2**-OH in H_2O at 23 °C recorded before (blue) and after irradiation with 365 nm (red) and 430 nm (blue dashed) light. The spectra after irradiation were recorded after reaching the PSS. b) ^{1}H NMR spectra (400 MHz, D_2O, 23 °C) of a different sample of **2**-OH recorded before and after irradiation using 365 nm and 430 nm light. The NMR spectra are shown in the order of the experiments from top to bottom and isomeric composition of *Z* and *E* isomer at PSS are indicated. Unlabeled NMR spectra illustrate the isomer enrichment before reaching the PSS. Impurities originating from the syringe filter used for sample preparation are marked with a star.

a)

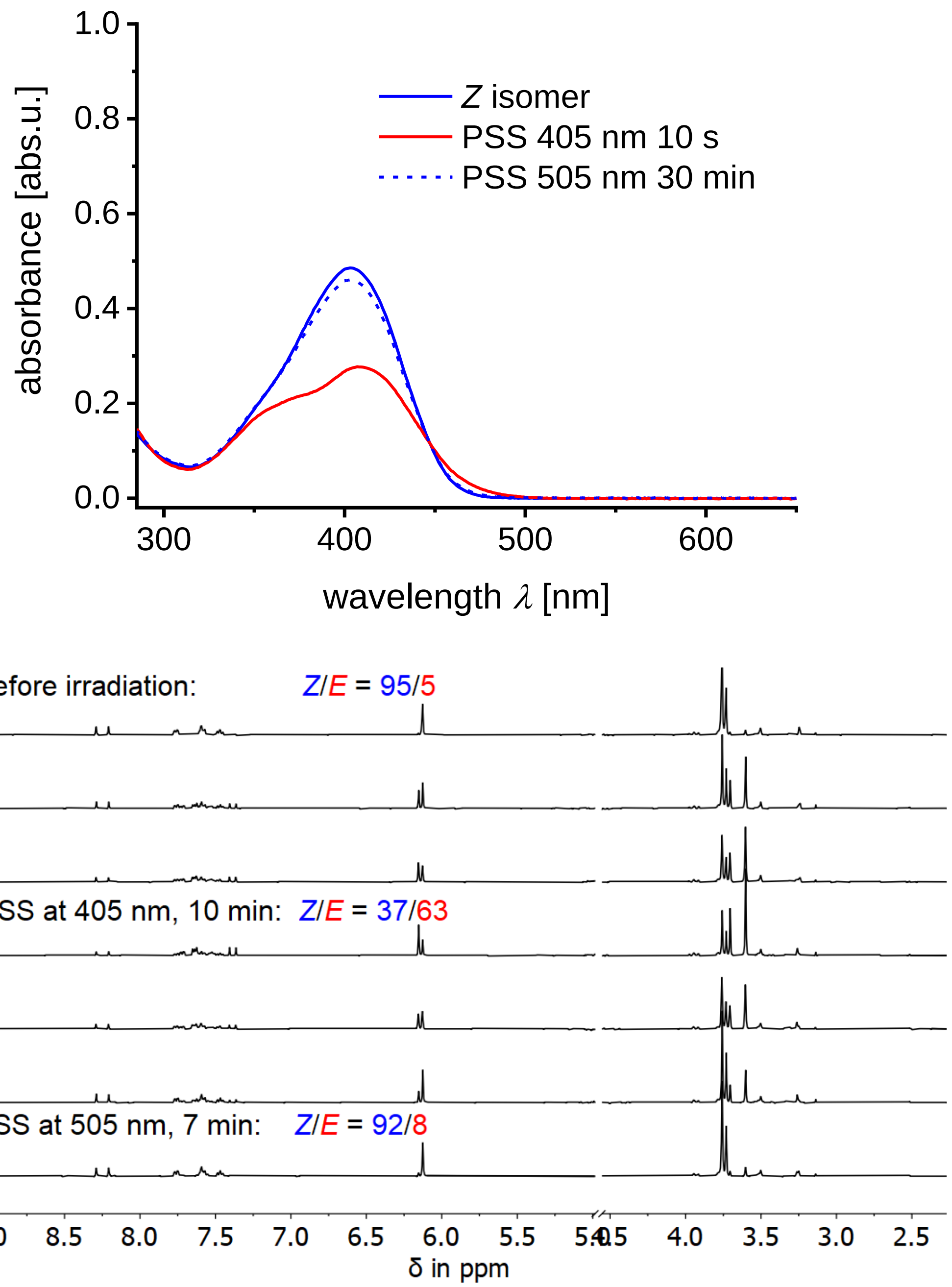

Supplementary Figure 110. Photoconversion of HPI **3**-OH upon irradiation with 405 nm and 505 nm light. a) UV/Vis absorption spectra of HPI **3**-OH in H_2O at 23 °C recorded before (blue) and after irradiation with 405 nm (red) and 505 nm (blue dashed) light. The spectra after irradiation were recorded after reaching the PSS. b) ^1H NMR spectra (400 MHz, D_2O, 6 °C) of a different sample of **3**-OH recorded before and after irradiation using 405 nm and 505 nm light at 6 °C. The NMR spectra are shown in the order of the experiments from top to bottom and isomeric composition of *Z* and *E* isomer at PSS are indicated. Unlabeled NMR spectra illustrate the isomer enrichment before reaching the PSS.

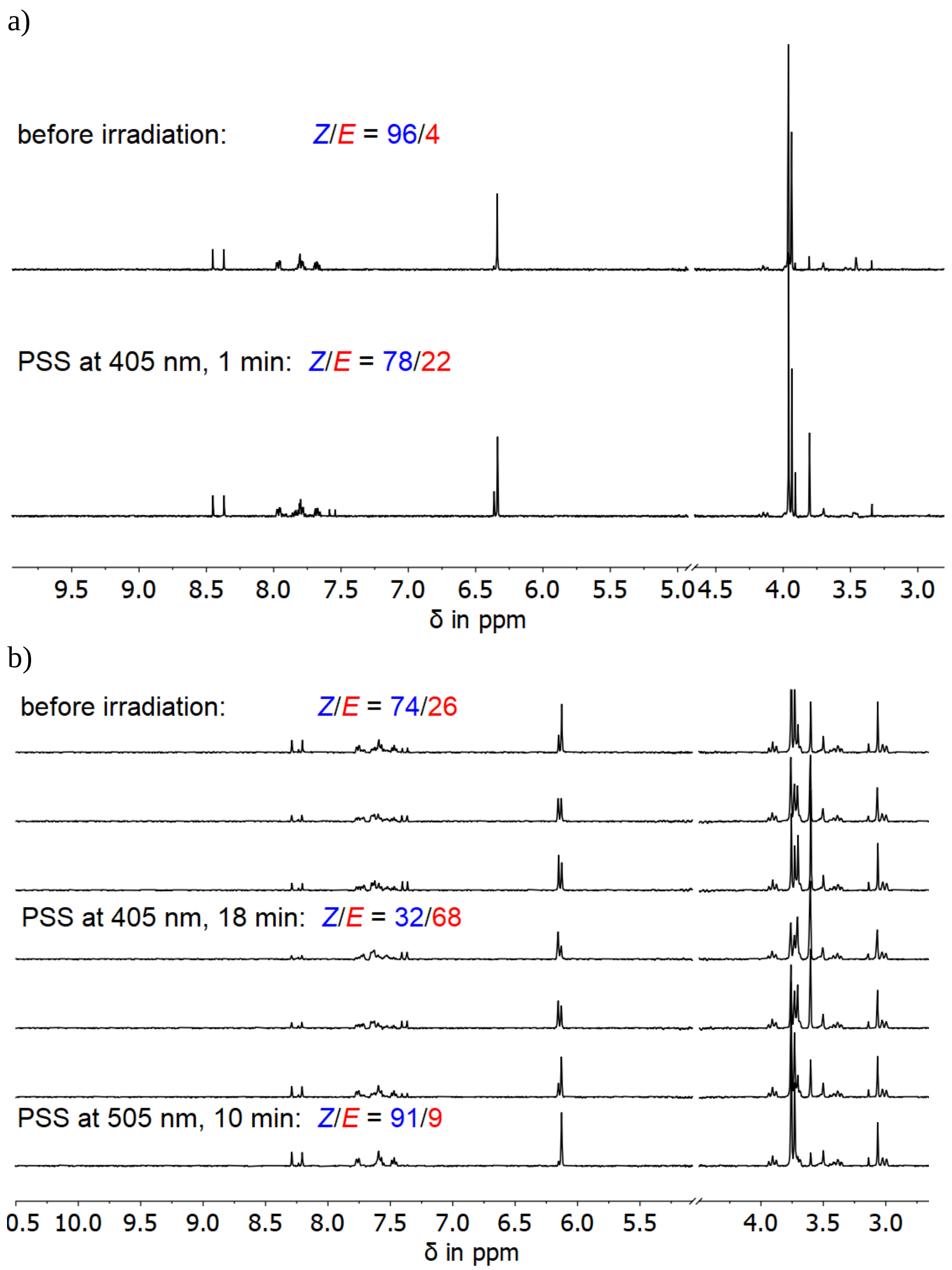

Supplementary Figure 111. Photoconversion of HPI **3**-OH upon irradiation with 405 nm and 505 nm light in aqueous solution. a) ^{1}H NMR spectra (400 MHz, D_2O, 23 °C) of **3**-OH recorded before and after irradiation using 405 nm light at 23 °C. b) ^{1}H NMR spectra (400 MHz, D_2O + K_2CO_3, 6 °C) of **3**-OH recorded before and after irradiation using 405 nm and 505 nm light at 6 °C. The NMR spectra are shown in the order of the experiments from top to bottom and isomeric composition of *Z* and *E* isomer at PSS are indicated. Unlabeled NMR spectra illustrate the isomer enrichment before reaching the PSS.

a)

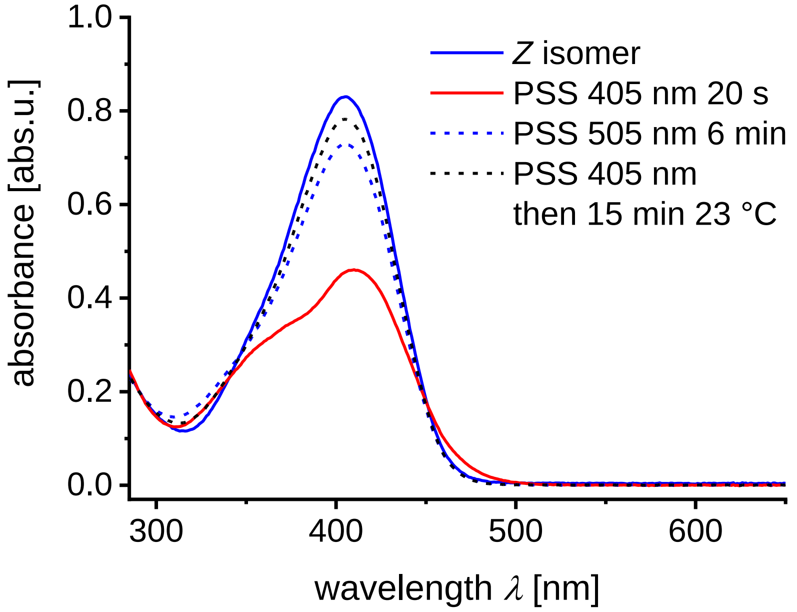

b)

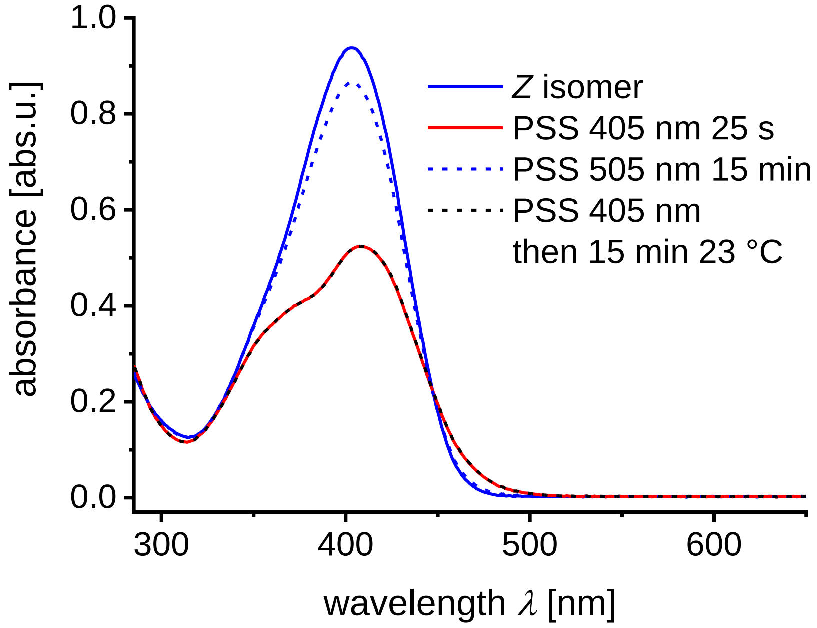

Supplementary Figure 112. UV/Vis absorption spectra of HPI **3**-OH upon irradiation with 405 nm and 505 nm light in different buffer solutions. The spectra after irradiation were recorded after reaching the PSS a) UV/Vis absorption spectra of HPI **3**-OH in Triz buffer pH=8.0 at 23 °C recorded before (blue) and after irradiation with 405 nm (red) and 505 nm (blue dashed) light. For an estimation of the magnitude of the thermal *E* to *Z* isomerization, the *E* enriched solution was measured again after 15 min at 23 °C (black). b) UV/Vis absorption spectra of HPI **3**-OH in PBS buffer pH=7.0 at 23 °C recorded before (blue) and after irradiation with 405 nm (red) and 505 nm (blue dashed) light. For an estimation of the magnitude of the thermal *E* to *Z* isomerization, the *E* enriched solution was measured again after 15 min at 23 °C (black).

a)

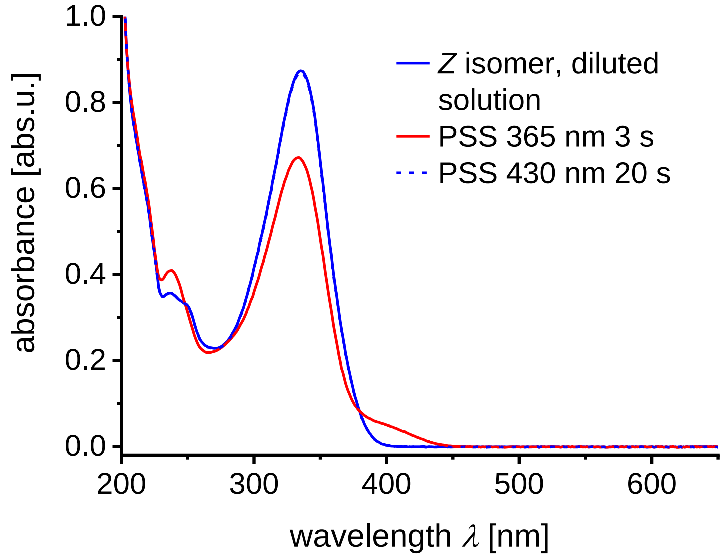

b)

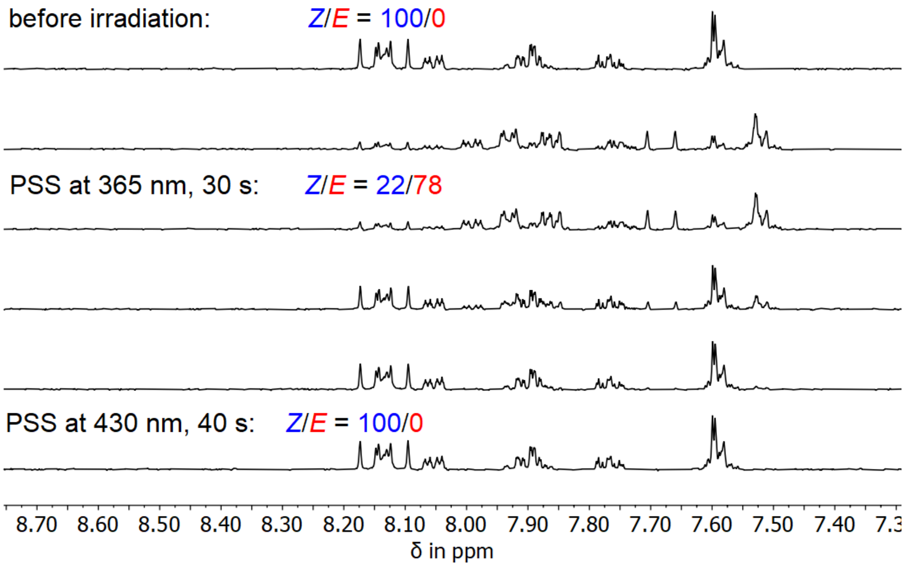

Supplementary Figure 113. Photoconversion of HPI **11**-OH upon irradiation with 365 nm and 430 nm light. a) UV/Vis absorption spectra of HPI **11**-OH in H_2O at 23 °C recorded before (blue) and after irradiation with 365 nm (red) and 430 nm (blue dashed) light. The spectra after irradiation were recorded after reaching the PSS. b) ^1H NMR spectra (400 MHz, D_2O, 23 °C) of a different sample of **11**-OH recorded before and after irradiation using 365 nm and 430 nm light. The NMR spectra are shown in the order of the experiments from top to bottom and isomeric composition of *Z* and *E* isomer at PSS are indicated. Unlabeled NMR spectra illustrate the isomer enrichment before reaching the PSS. Impurities originating from the syringe filter used for sample preparation are marked with a star.

a)

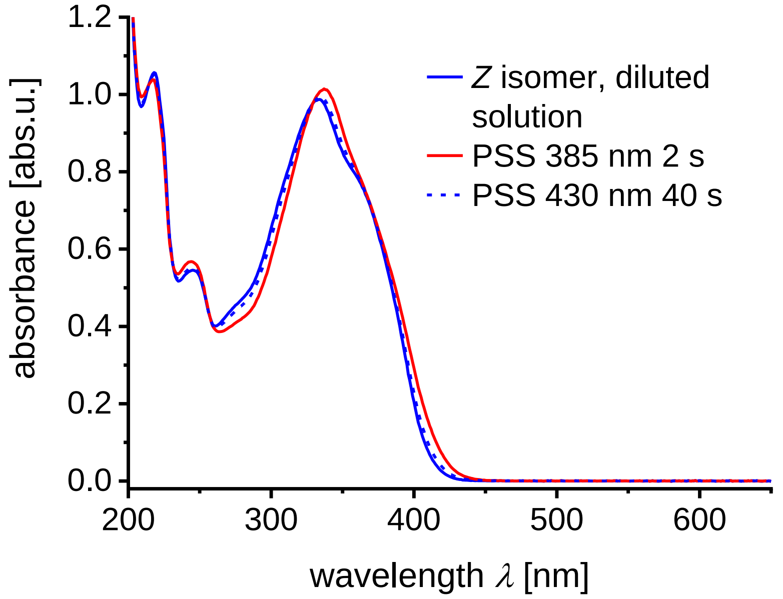

b)

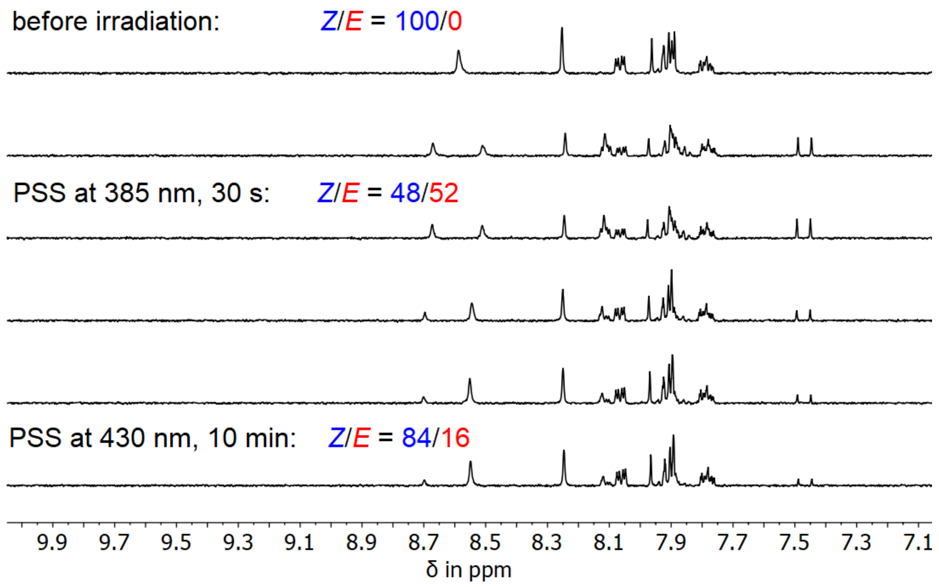

Supplementary Figure 114. Photoconversion of HPI **17**-OH upon irradiation with 385 nm and 430 nm light. a) UV/Vis absorption spectra of HPI **17**-OH in H_2O at 23 °C recorded before (blue) and after irradiation with 385 nm (red) and 430 nm (blue dashed) light. The spectra after irradiation were recorded after reaching the PSS. b) ^{1}H NMR spectra (400 MHz, D_2O, 23 °C) of a different sample of **17**-OH recorded before and after irradiation using 385 nm and 430 nm light. The NMR spectra are shown in the order of the experiments from top to bottom and isomeric composition of *Z* and *E* isomer at PSS are indicated. Unlabeled NMR spectra illustrate the isomer enrichment before reaching the PSS. Impurities originating from the syringe filter used for sample preparation are marked with a star.

Supplementary Note 11: Summary of all photoisomerization experiments

Supplementary Table 9. Summary of all photoisomerization experiments of HPIs **1-18** and selected hydrolyzed derivatives in toluene and water.

HPI	Solvent	T [°C]	λ for *Z* to *E* isomerization [nm]	[*E*] at PSS [%]	λ for *E* to *Z* isomerization [nm]	[*Z*] at PSS [%]	Comment
**1**	toluene-d_8	23	395	88	450	98	-
**1**	water	-	-	-	-	-	not soluble and addressable
**1-OH**	D_2O	23	365	70	430	100	-
**2**	toluene-d_8	23	300	80	450	100	-
**2**	water	23	365	-	430	-	not soluble enough for NMR
**2-OH**	D_2O	23	365	63	430	100	-
**3**	toluene-d_8	23	395	82	470	98	-
**3**	water	23	385	-	470	-	not soluble enough for NMR
**3-OH**	D_2O	6	405	63	505	92	-
**3-OH**	D_2O + K_2CO_3	6	405	68	505	91	-
**4**	toluene-d_8	23	395	79	450	99	-
**4**	water	23	365	n.d.	450	n.d.	-
**5**	toluene-d_8	23	395	68	450	98	-
**5**	water	23	365	n.d.	470	n.d.	-
**6**	toluene-d_8	23	395	81	470	98	-
**6**	water	23	365	n.d.	470	n.d	-
**7**	toluene-d_8	23	430	47	530	91	
**7**	water	23	-	-	-	-	no photoswitching
**8**	toluene-d_8	23	470	31	565	91	-
**8**	water	23	-	-	-	-	no photoswitching

**9**	toluene-*d*$_8$	23	450	41	505	93	-
**9**	water	23	530	-	-	-	thermal isomerization too fast
**10**	toluene-*d*$_8$	23	365	65	470	98	-
**10**	water	23	365	n.d.	470	n.d.	-
**11**	toluene-*d*$_8$	23	340	66	450	99	-
**11**	D_2O	23	365	-	450	-	degradation
**11-**OH	D_2O	23	365	78	430	100	
**12**	toluene-*d*$_8$	23	340	60	450	97	
**12**	water	23	-	-	-	-	no photoswitching
**13**	toluene-*d*$_8$	23	340	53	450	98	-
**13**	water	23	-	-	-	-	different thermal reaction
**14**	toluene-*d*$_8$	23	365	53	450	98	-
**14**	water	23	-	-	-	-	irreversible photoreaction
**15**	toluene-*d*$_8$	23	365	69	450	99	-
**15**	D_2O	23	365	18	450	89	-
**16**	toluene-*d*$_8$	23	395	66	470	99	-
**16**	water	23	-	-	-	-	no photoswitching
**17**	toluene-*d*$_8$	23	340	84	450	100	-
**17**	D_2O	23	340	42	450	99	-
**17-**OH	D_2O	23	385	52	430	84	
**18**	toluene-*d*$_8$	23	430	63	470	60	-
**18**	water	23	-	-	-	-	no photoswitching

Supplementary Note 12: Photofatigue experiments

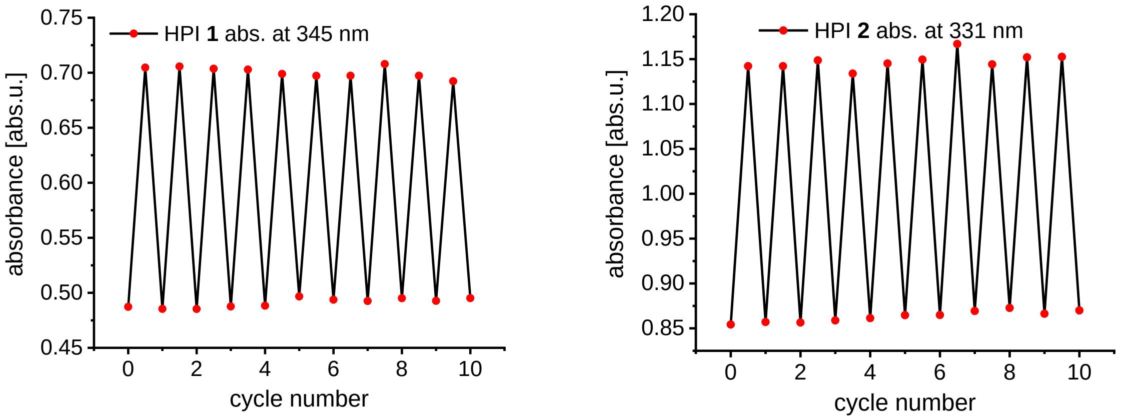

Supplementary Figure 115. Photostability experiments of HPI **1** and **2** in toluene. (left) Absorption of HPI **1** at 345 nm, monitored over 10 irradiation cycles with 395 nm and 450 nm *via* UV/Vis spectroscopy. (right) Absorption of HPI **2** at 331 nm, monitored over 10 irradiation cycles with 300 nm and 450 nm *via* UV/Vis spectroscopy.

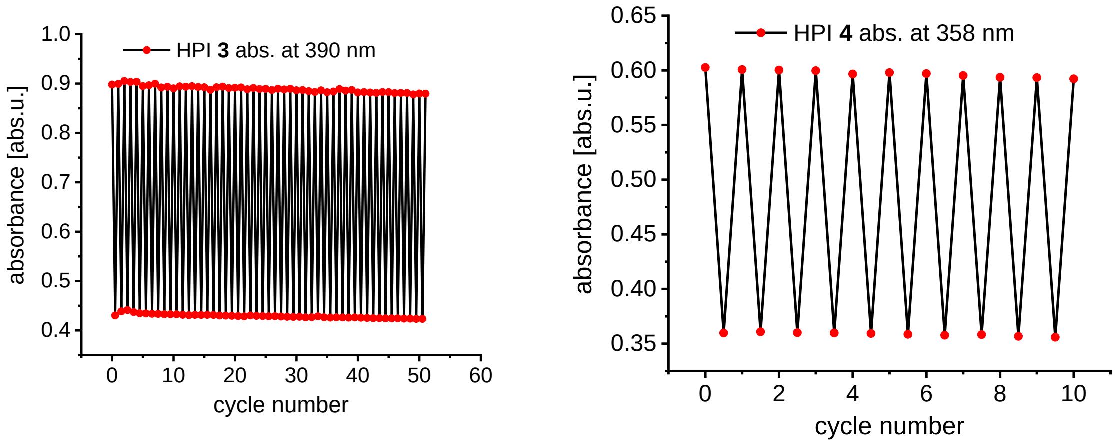

Supplementary Figure 116. Photostability experiments of HPI **3** and **4** in toluene. (left) Absorption of HPI **3** at 390 nm, monitored over 50 irradiation cycles with 395 nm and 470 nm *via* UV/Vis spectroscopy. (right) Absorption of HPI **4** at 358 nm, monitored over 10 irradiation cycles with 395 nm and 450 nm *via* UV/Vis spectroscopy.

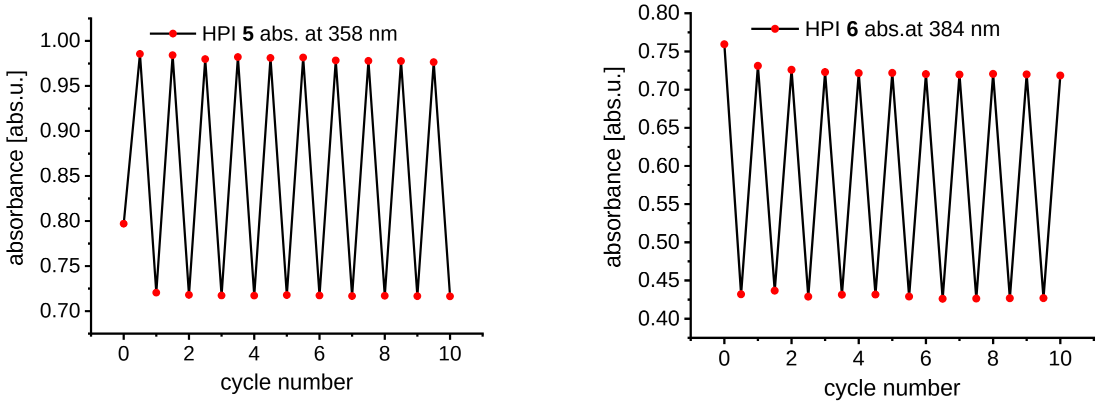

Supplementary Figure 117. Photostability experiments of HPI **5** and **6** in toluene. (left) Absorption of HPI **5** at 358 nm, monitored over 10 irradiation cycles with 395 nm and 450 nm *via* UV/Vis spectroscopy. (right) Absorption of HPI **6** at 358 nm, monitored over 10 irradiation cycles with 395 nm and 470 nm *via* UV/Vis spectroscopy.

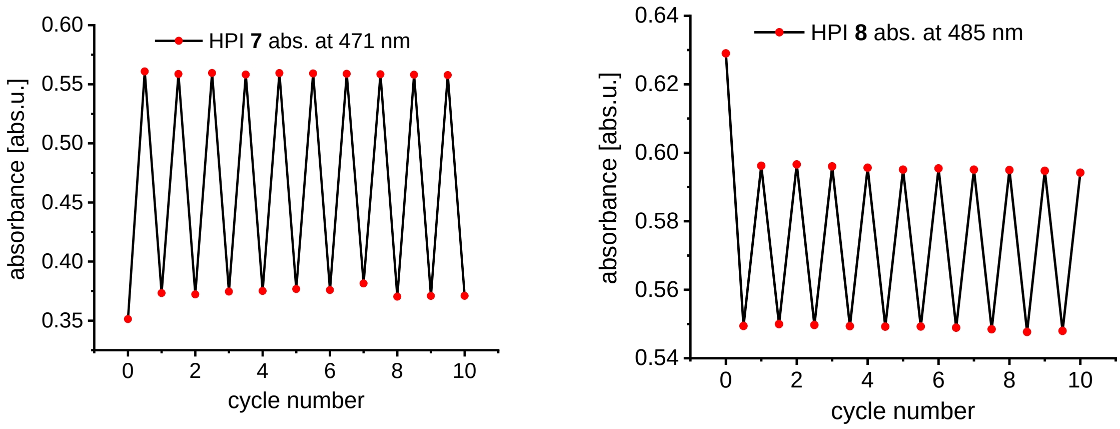

Supplementary Figure 118. Photostability experiments of HPI **7** and **8** in toluene. (left) Absorption of HPI **7** at 471 nm, monitored over 10 irradiation cycles with 430 nm and 530 nm *via* UV/Vis spectroscopy. (right) Absorption of HPI **8** at 485 nm, monitored over 10 irradiation cycles with 470 nm and 565 nm *via* UV/Vis spectroscopy.

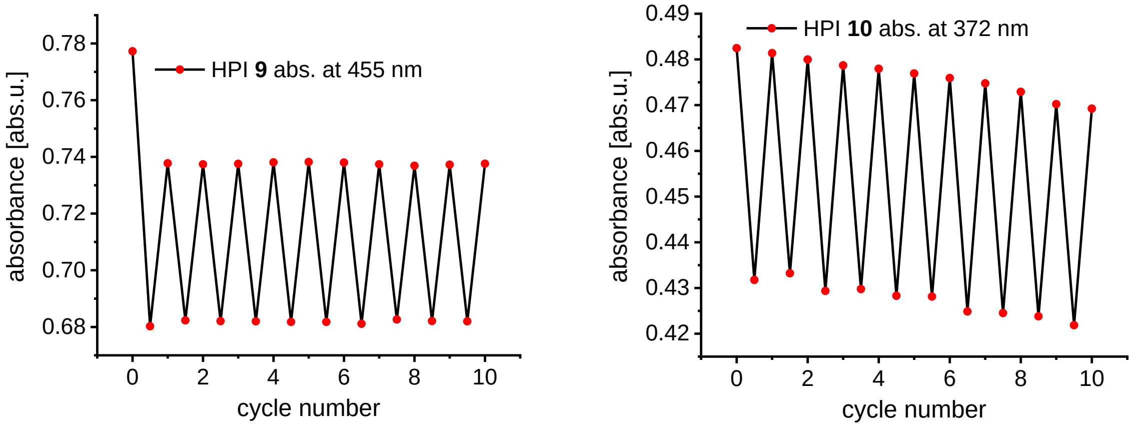

Supplementary Figure 119. Photostability experiments of HPI **9** and **10** in toluene. (left) Absorption of HPI **9** at 455 nm, monitored over 10 irradiation cycles with 450 nm and 505 nm *via* UV/Vis spectroscopy. (right) Absorption of HPI **10** at 372 nm, monitored over 10 irradiation cycles with 365 nm and 470 nm *via* UV/Vis spectroscopy.

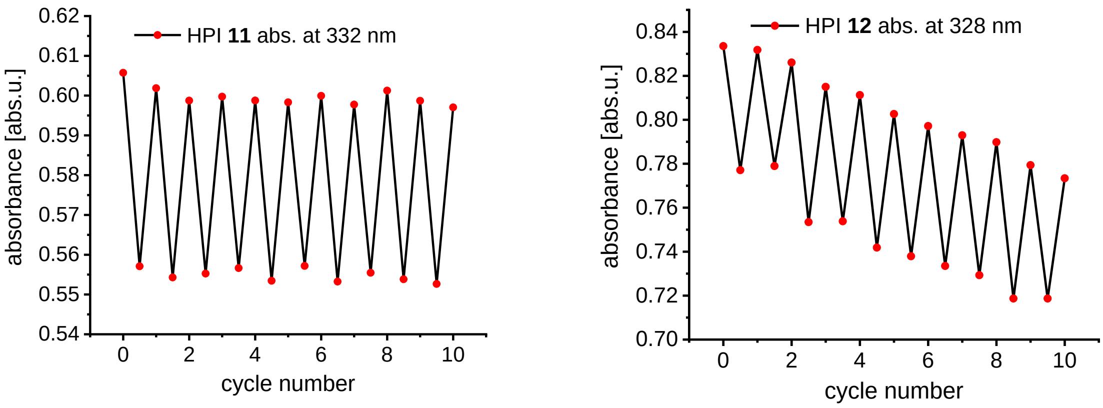

Supplementary Figure 120. Photostability experiments of HPI **11** and **12** in toluene. (left) Absorption of HPI **11** at 332 nm, monitored over 10 irradiation cycles with 340 nm and 450 nm *via* UV/Vis spectroscopy. (right) Absorption of HPI **12** at 328 nm, monitored over 10 irradiation cycles with 340 nm and 450 nm *via* UV/Vis spectroscopy.

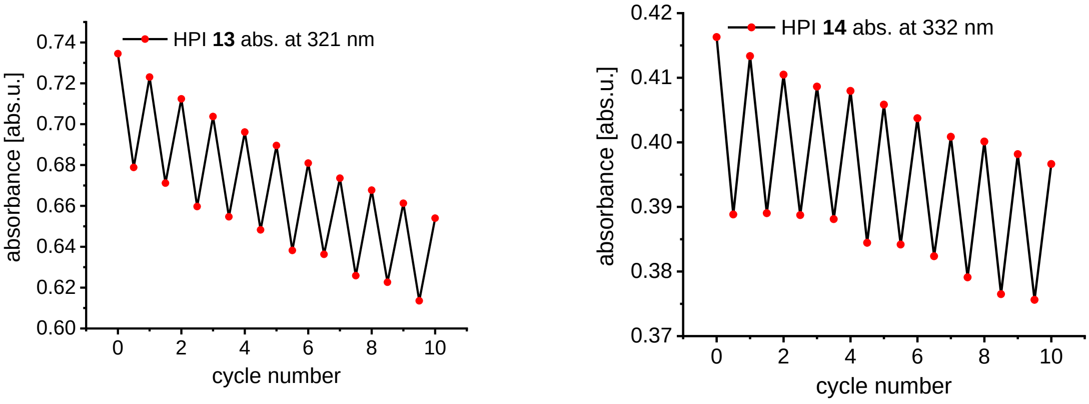

Supplementary Figure 121. Photostability experiments of HPI **13** and **14** in toluene. (left) Absorption of HPI **13** at 321 nm, monitored over 10 irradiation cycles with 340 nm and 450 nm *via* UV/Vis spectroscopy. (right) Absorption of HPI **14** at 332 nm, monitored over 10 irradiation cycles with 365 nm and 450 nm *via* UV/Vis spectroscopy.

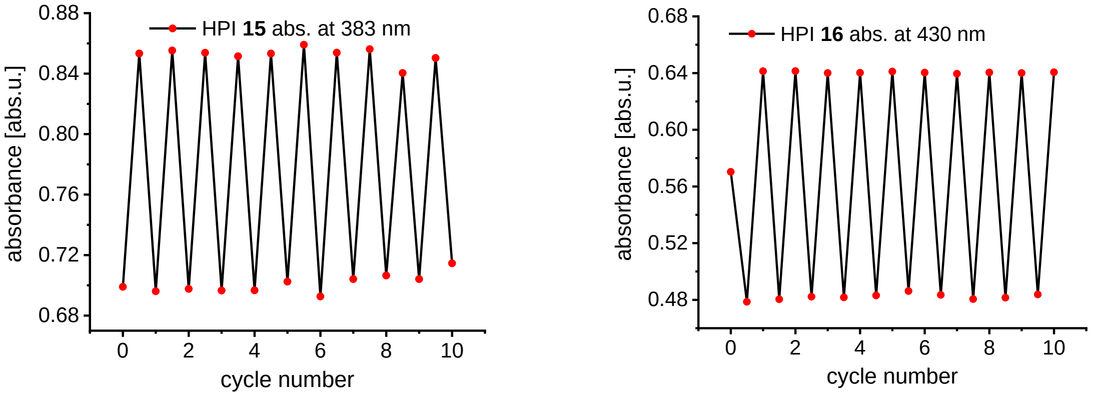

Supplementary Figure 122. Photostability experiments of HPI **15** and **16** in toluene. (left) Absorption of HPI **15** at 383 nm, monitored over 10 irradiation cycles with 365 nm and 450 nm *via* UV/Vis spectroscopy. (right) Absorption of HPI **16** at 430 nm, monitored over 10 irradiation cycles with 395 nm and 450 nm *via* UV/Vis spectroscopy.

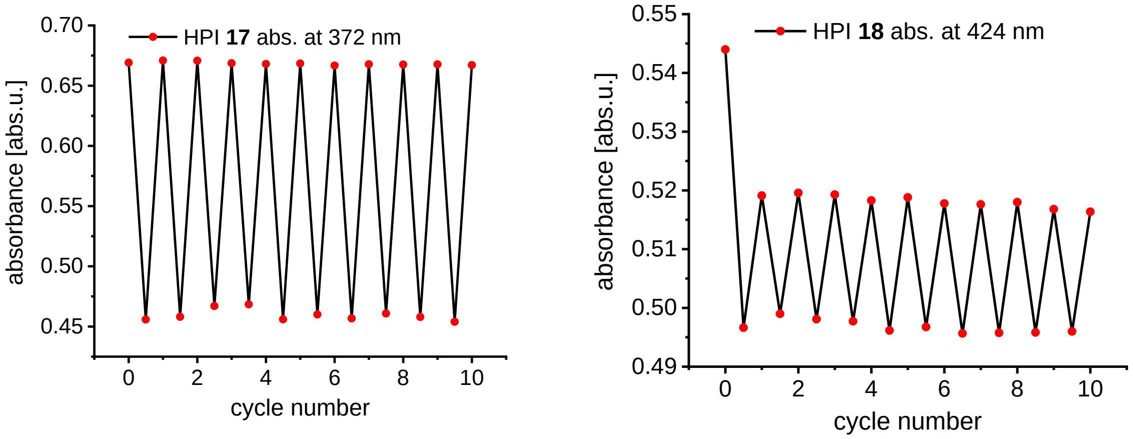

Supplementary Figure 123. Photostability experiments of HPI **17** and **18** in toluene. (left) Absorption of HPI **17** at 372 nm, monitored over 10 irradiation cycles with 340 nm and 450 nm *via* UV/Vis spectroscopy. (right) Absorption of HPI **18** at 424 nm, monitored over 10 irradiation cycles with 430 nm and 470 nm *via* UV/Vis spectroscopy.

Supplementary Note 13: Quantum yield determination

The quantum yield ϕ for a photochemical reaction is the ratio of the number of isomerized product molecules and the total number of absorbed photons.

$$\phi = \frac{N(isomerized\ molecules)}{N(absorbed\ photons)} \tag{18}$$

The quantum yields for both photoisomerization reactions (*Z* to *E* and *E* to *Z)* were determined using the instrumental setup of *Riedle*.[18] Samples were prepared in 2.02-2.14 mL toluene (filtered through basic Al_2O_3 and Na_2SO_4) or water with absorptions ranging from 0.7 to 1.4. The samples were irradiated with light of wavelength close to the isosbestic point. After each irradiation step, the power of the solar cell detector and a UV/Vis absorption spectrum were recorded. Irradiation was continued until the photostationary state was reached. The setup allows for determination of both isomerization reactions (forwards and backwards) in one measurement by calculating the concentration changes from the previously determined molar absorption coefficients and the absorption spectra and performing a kinetic fit. Supplementary Table 10 summarizes all calculated photoisomerization quantum yields.

Supplementary Table 10. Summary of all photoreaction quantum yields of HPIs **1-18** and HPI **3**-OH in the corresponding solvent.

**HPI**	**Solvent**	**Irradiation wavelength [nm]**	$\phi_{Z\rightarrow E}$	$\phi_{E\rightarrow Z}$
**1**	toluene	340	50%	1%
**2**	toluene	365	40%	10%
**3**	toluene	340	52%	24%
**3-OH**	H_2O	340	9%	7%
**4**	toluene	340	47%	21%
**5**	toluene	340	37%	9%
**6**	toluene	340	45%	23%
**7**	toluene	425	3%	2%
**8**	toluene	470	21%	22%
**9**	toluene	470	26%	23%
**10**	toluene	365	51%	18%
**11**	toluene	340	46%	30%
**12**	toluene	340	48%	30%
**13**	toluene	340	51%	49%
**14**	toluene	365	36%	59%
**15**	toluene	365	29%	33%
**16**	toluene	425	12%	14%
**17**	toluene	340	30%	12%
**18**	toluene	425	35%	11%

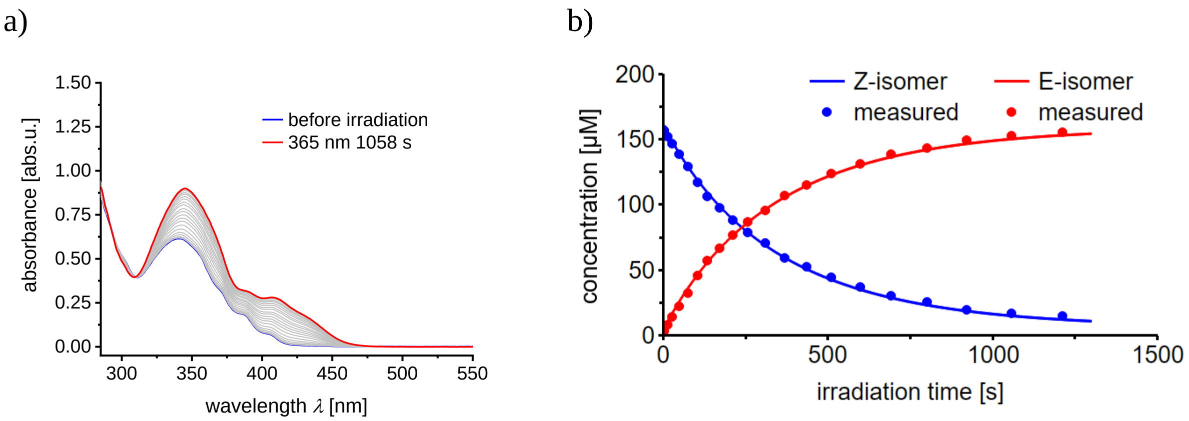

Supplementary Figure 124. Photoisomerization quantum yield determination of HPI **1** in toluene at 23 °C. a) UV/Vis absorption spectra recorded after each 365 nm irradiation step, starting with *Z*-isomer enriched solution. Irradiation was continued until the photostationary state (PSS) was reached. b) Measured and fitted changes (blue and red lines) in concentration of *Z* and *E* isomers during the photoreaction.

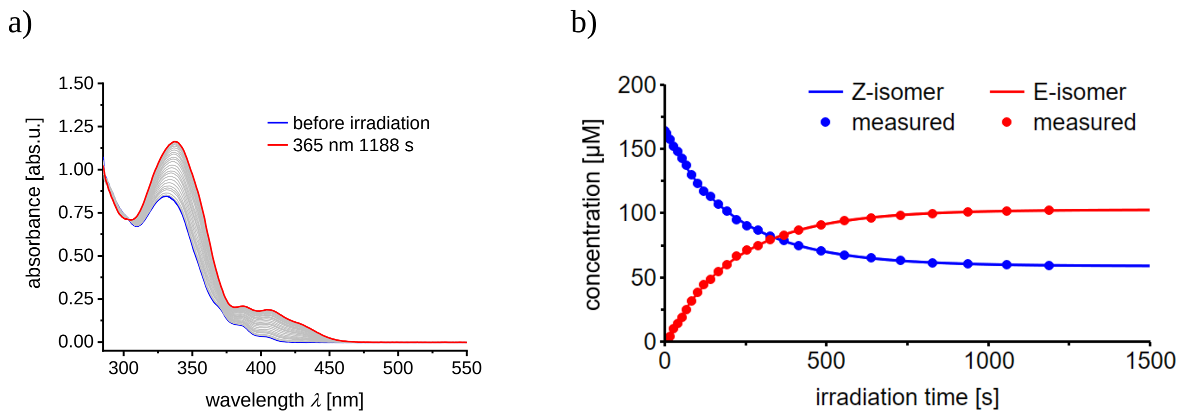

Supplementary Figure 125. Photoisomerization quantum yield determination of HPI **2** in toluene at 23 °C. a) UV/Vis absorption spectra recorded after each 365 nm irradiation step, starting with pure *Z*-isomer. Irradiation was continued until the photostationary state (PSS) was reached. b) Measured and fitted changes (blue and red lines) in concentration of *Z* and *E* isomers during the photoreaction.

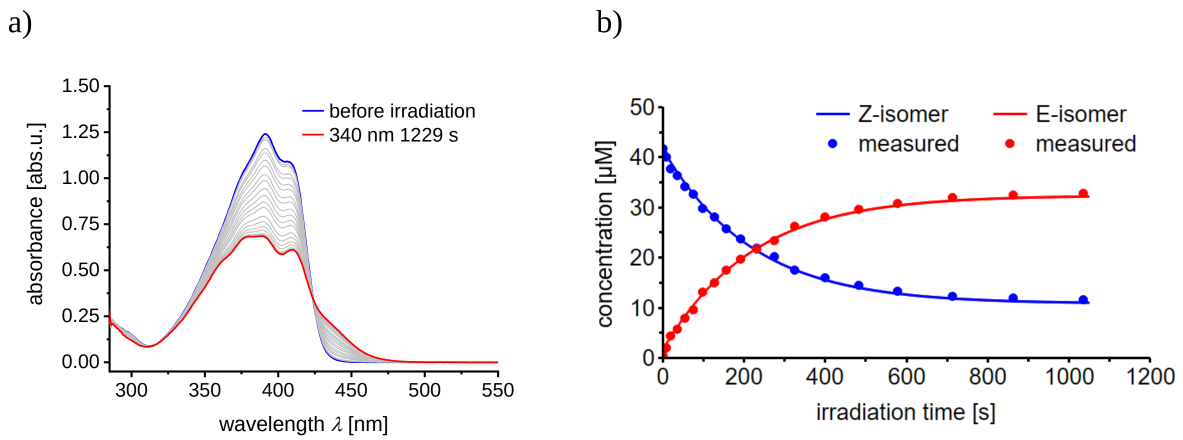

Supplementary Figure 126. Photoisomerization quantum yield determination of HPI **3** in toluene at 23 °C. a) UV/Vis absorption spectra recorded after each 340 nm irradiation step, starting with pure *Z*-isomer. Irradiation was continued until the photostationary state (PSS) was reached. b) Measured and fitted changes (blue and red lines) in concentration of *Z* and *E* isomers during the photoreaction.

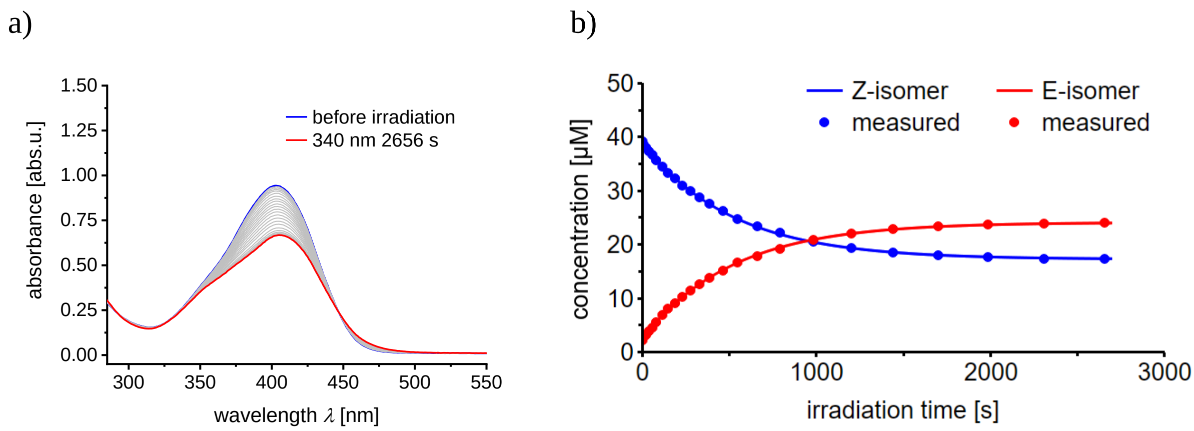

Supplementary Figure 127. Photoisomerization quantum yield determination of HPI **3-OH** in H_2O at 23 °C. a) UV/Vis absorption spectra recorded after each 340 nm irradiation step, starting with *Z*-isomer enriched solution. Irradiation was continued until the photostationary state (PSS) was reached. b) Measured and fitted changes (blue and red lines) in concentration of *Z* and *E* isomers during the photoreaction.

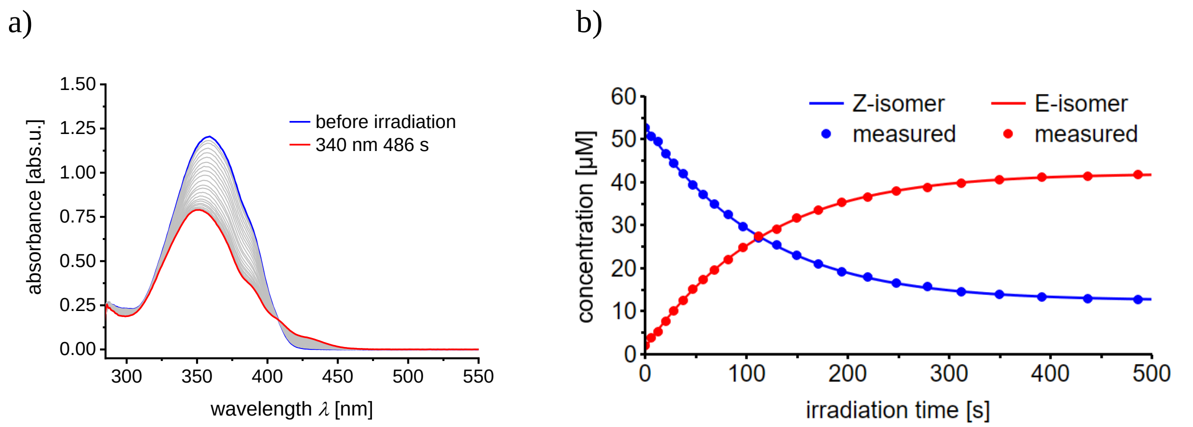

Supplementary Figure 128. Photoisomerization quantum yield determination of HPI **4** in toluene at 23 °C. a) UV/Vis absorption spectra recorded after each 340 nm irradiation step, starting with *Z*-isomer enriched solution. Irradiation was continued until the photostationary state (PSS) was reached. b) Measured and fitted changes (blue and red lines) in concentration of *Z* and *E* isomers during the photoreaction.

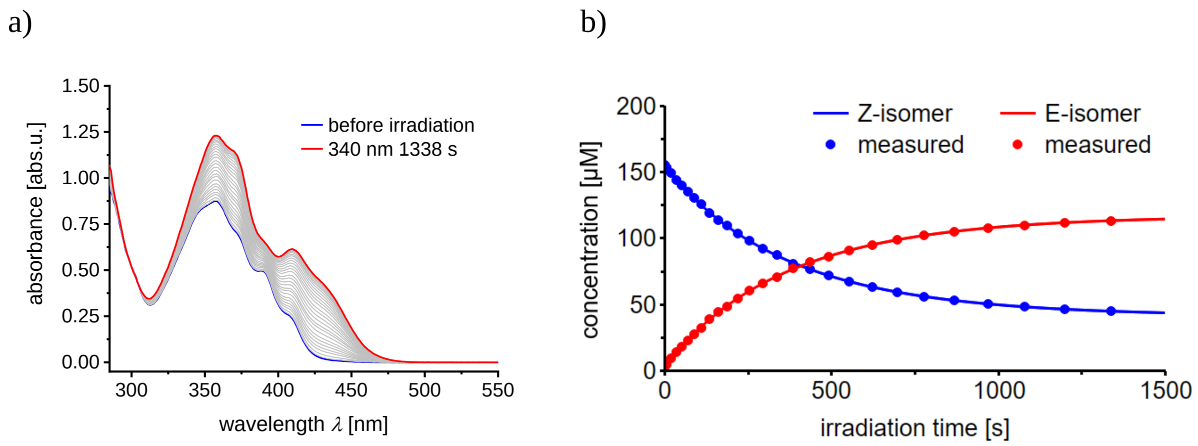

Supplementary Figure 129. Photoisomerization quantum yield determination of HPI **5** in toluene at 23 °C. a) UV/Vis absorption spectra recorded after each 340 nm irradiation step, starting with pure *Z*-isomer. Irradiation was continued until the photostationary state (PSS) was reached. b) Measured and fitted changes (blue and red lines) in concentration of *Z* and *E* isomers during the photoreaction.

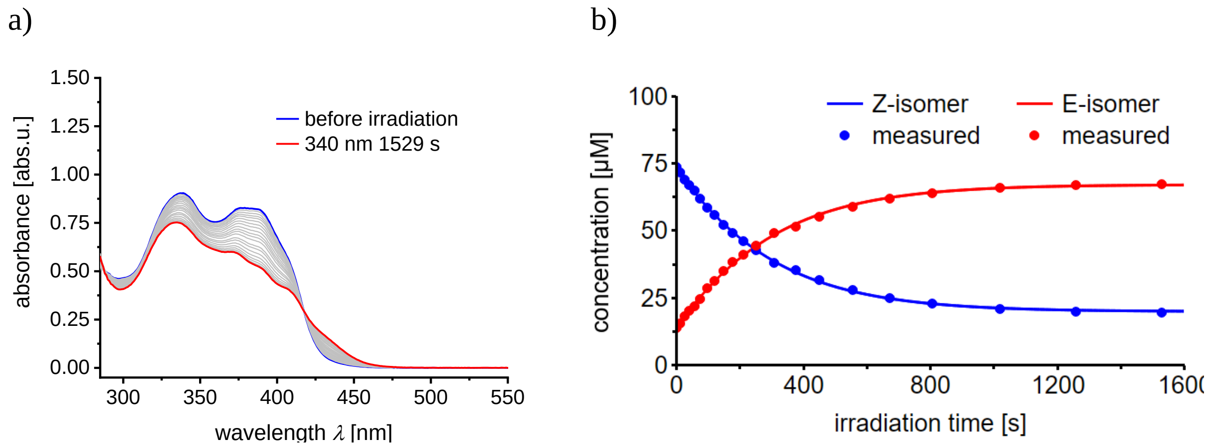

Supplementary Figure 130. Photoisomerization quantum yield determination of HPI **6** in toluene at 23 °C. a) UV/Vis absorption spectra recorded after each 340 nm irradiation step, starting with *Z*-isomer enriched solution. Irradiation was continued until the photostationary state (PSS) was reached. b) Measured and fitted changes (blue and red lines) in concentration of *Z* and *E* isomers during the photoreaction.

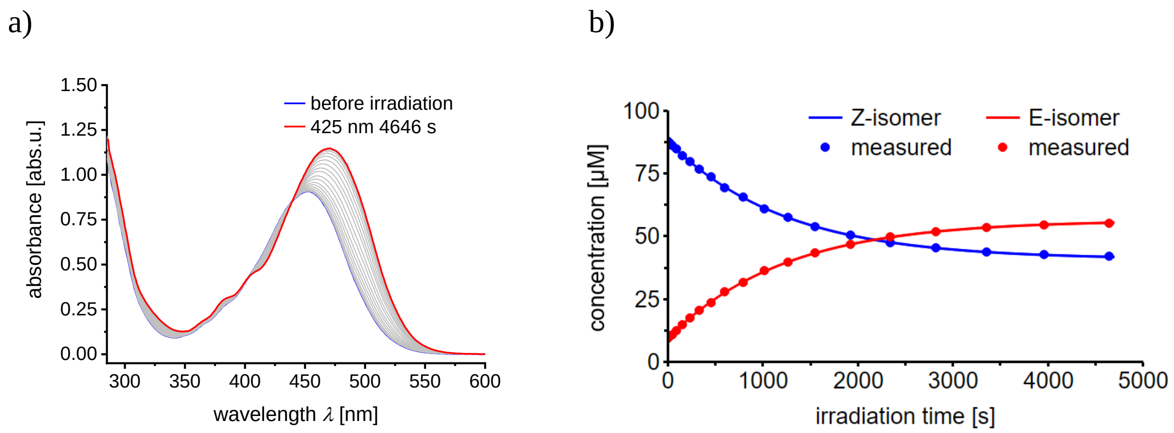

Supplementary Figure 131. Photoisomerization quantum yield determination of HPI **7** in toluene at 23 °C. a) UV/Vis absorption spectra recorded after each 425 nm irradiation step, starting with pure *Z*-isomer. Irradiation was continued until the photostationary state (PSS) was reached. b) Measured and fitted changes (blue and red lines) in concentration of *Z* and *E* isomers during the photoreaction.

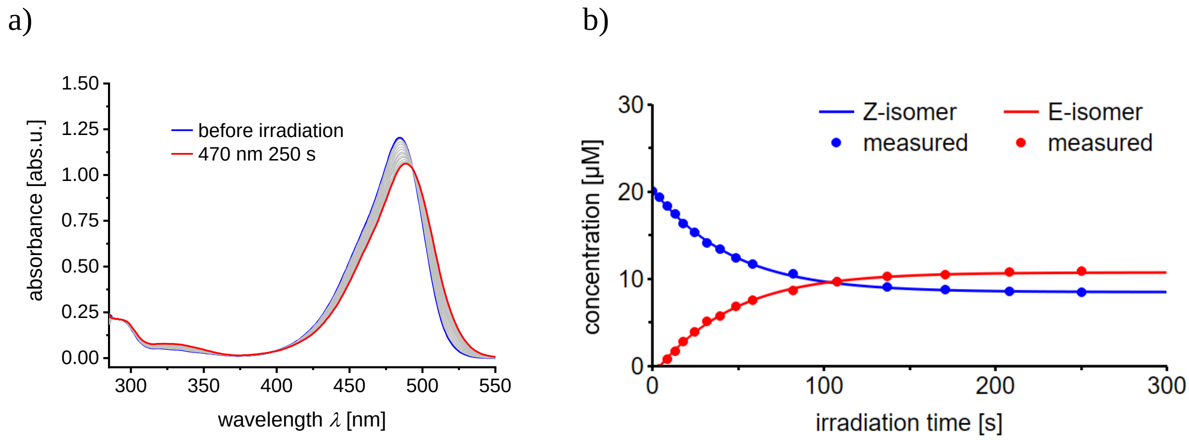

Supplementary Figure 132. Photoisomerization quantum yield determination of HPI **8** in toluene at 23 °C. a) UV/Vis absorption spectra recorded after each 470 nm irradiation step, starting with pure *Z*-isomer. Irradiation was continued until the photostationary state (PSS) was reached. b) Measured and fitted changes (blue and red lines) in concentration of *Z* and *E* isomers during the photoreaction.

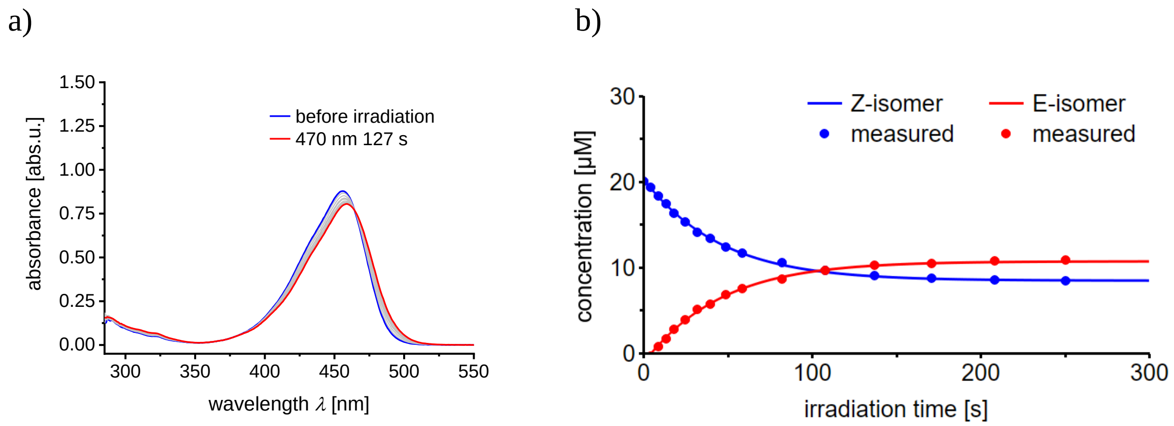

Supplementary Figure 133. Photoisomerization quantum yield determination of HPI **9** in toluene at 23 °C. a) UV/Vis absorption spectra recorded after each 470 nm irradiation step, starting with pure *Z*-isomer. Irradiation was continued until the photostationary state (PSS) was reached. b) Measured and fitted changes (blue and red lines) in concentration of *Z* and *E* isomers during the photoreaction.

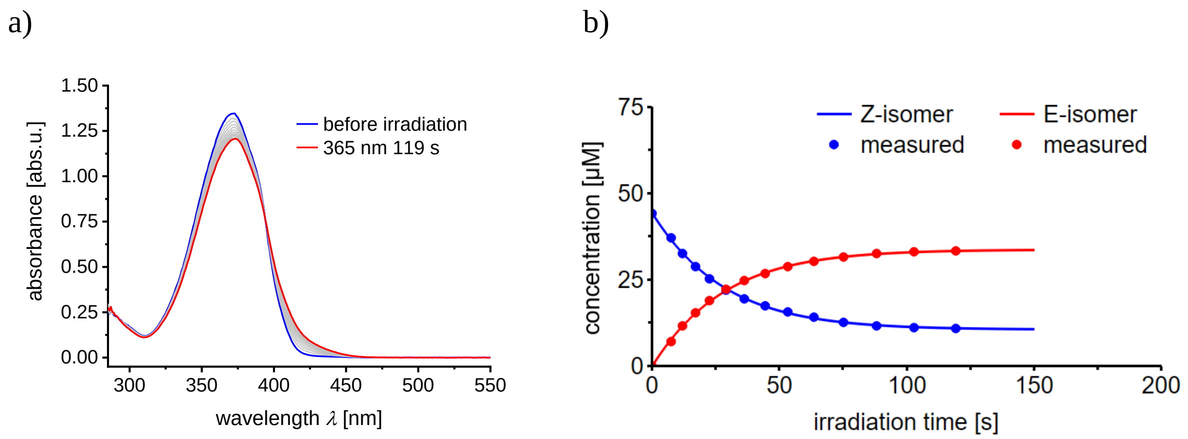

Supplementary Figure 134. Photoisomerization quantum yield determination of HPI **10** in toluene at 23 °C. a) UV/Vis absorption spectra recorded after each 365 nm irradiation step, starting with pure *Z*-isomer. Irradiation was continued until the photostationary state (PSS) was reached. b) Measured and fitted changes (blue and red lines) in concentration of *Z* and *E* isomers during the photoreaction.

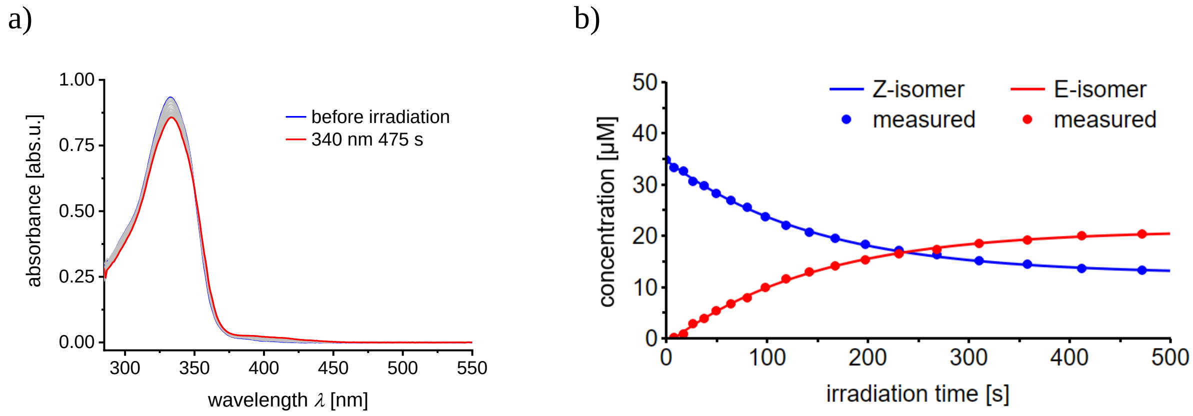

Supplementary Figure 135. Photoisomerization quantum yield determination of HPI **11** in toluene at 23 °C. a) UV/Vis absorption spectra recorded after each 340 nm irradiation step, starting with pure *Z*-isomer. Irradiation was continued until the photostationary state (PSS) was reached. b) Measured and fitted changes (blue and red lines) in concentration of *Z* and *E* isomers during the photoreaction.

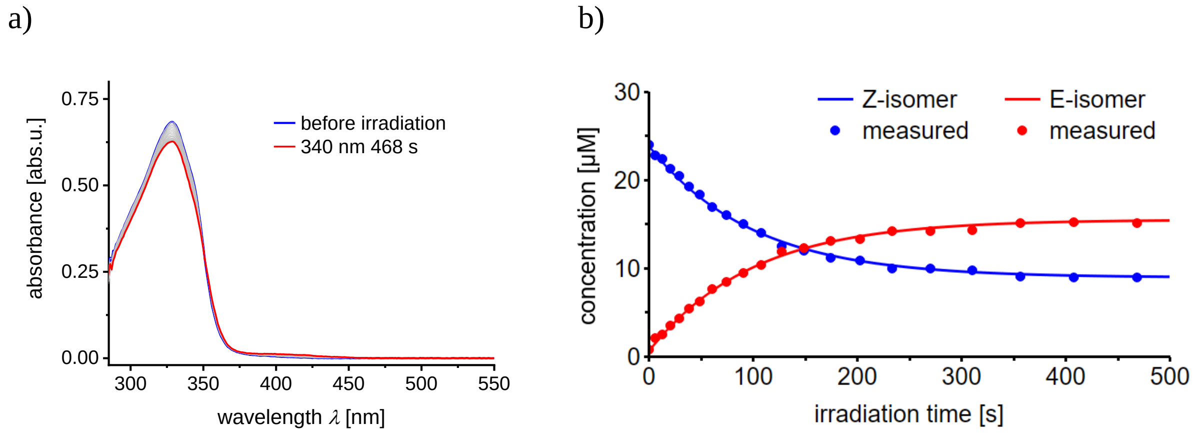

Supplementary Figure 136. Photoisomerization quantum yield determination of HPI **12** in toluene at 23 °C. a) UV/Vis absorption spectra recorded after each 340 nm irradiation step, starting with pure *Z*-isomer. Irradiation was continued until the photostationary state (PSS) was reached. b) Measured and fitted changes (blue and red lines) in concentration of *Z* and *E* isomers during the photoreaction.

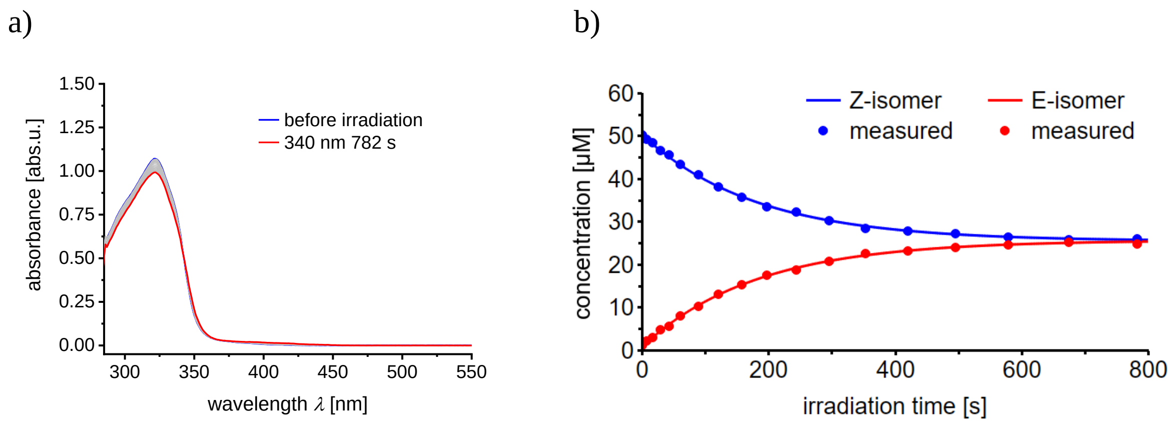

Supplementary Figure 137. Photoisomerization quantum yield determination of HPI **13** in toluene at 23 °C. a) UV/Vis absorption spectra recorded after each 340 nm irradiation step, starting with pure *Z*-isomer. Irradiation was continued until the photostationary state (PSS) was reached. b) Measured and fitted changes (blue and red lines) in concentration of *Z* and *E* isomers during the photoreaction.

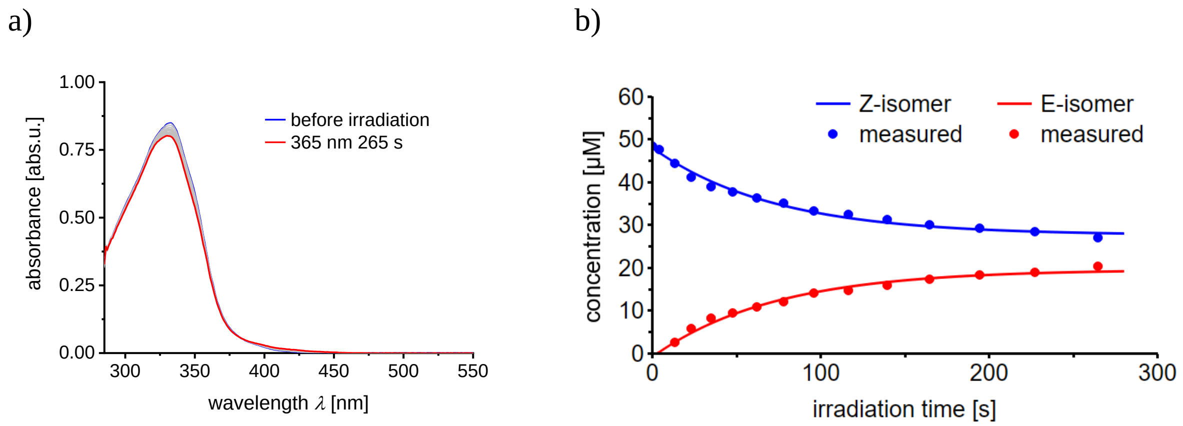

Supplementary Figure 138. Photoisomerization quantum yield determination of HPI **14** in toluene at 23 °C. a) UV/Vis absorption spectra recorded after each 365 nm irradiation step, starting with pure *Z*-isomer. Irradiation was continued until the photostationary state (PSS) was reached. b) Measured and fitted changes (blue and red lines) in concentration of *Z* and *E* isomers during the photoreaction.

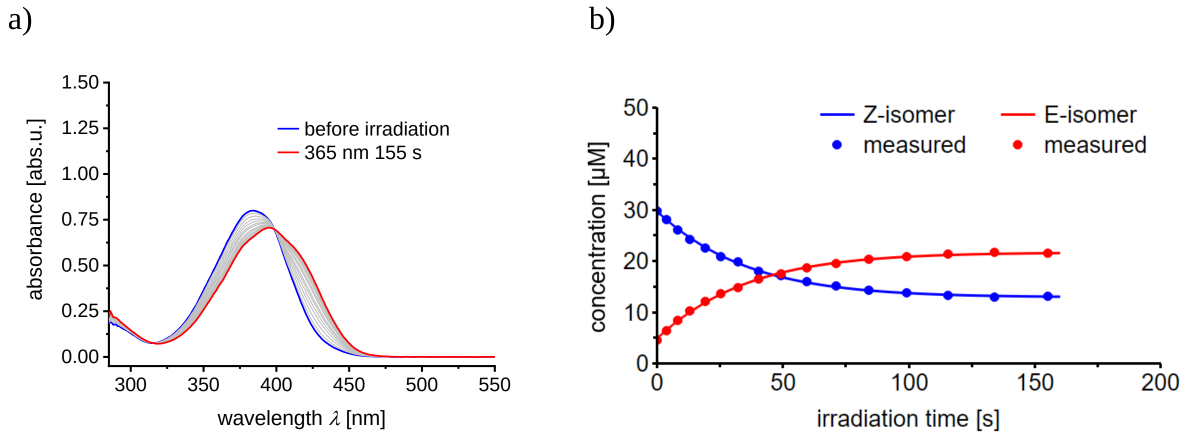

Supplementary Figure 139. Photoisomerization quantum yield determination of HPI **15** in toluene at 23 °C. a) UV/Vis absorption spectra recorded after each 365 nm irradiation step, starting with pure *Z*-isomer. Irradiation was continued until the photostationary state (PSS) was reached. b) Measured and fitted changes (blue and red lines) in concentration of *Z* and *E* isomers during the photoreaction.

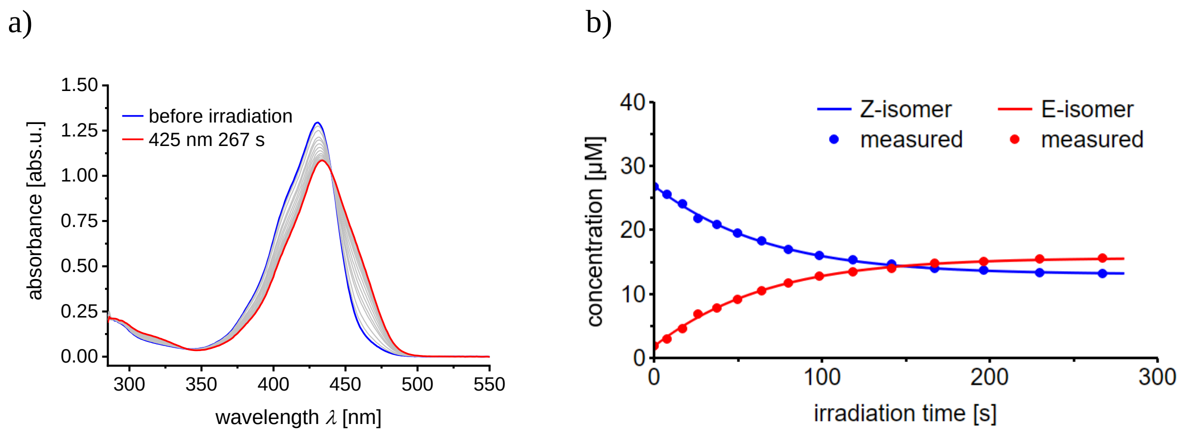

Supplementary Figure 140. Photoisomerization quantum yield determination of HPI **16** in toluene at 23 °C. a) UV/Vis absorption spectra recorded after each 425 nm irradiation step, starting with *Z*-isomer enriched solution. Irradiation was continued until the photostationary state (PSS) was reached. b) Measured and fitted changes (blue and red lines) in concentration of *Z* and *E* isomers during the photoreaction.

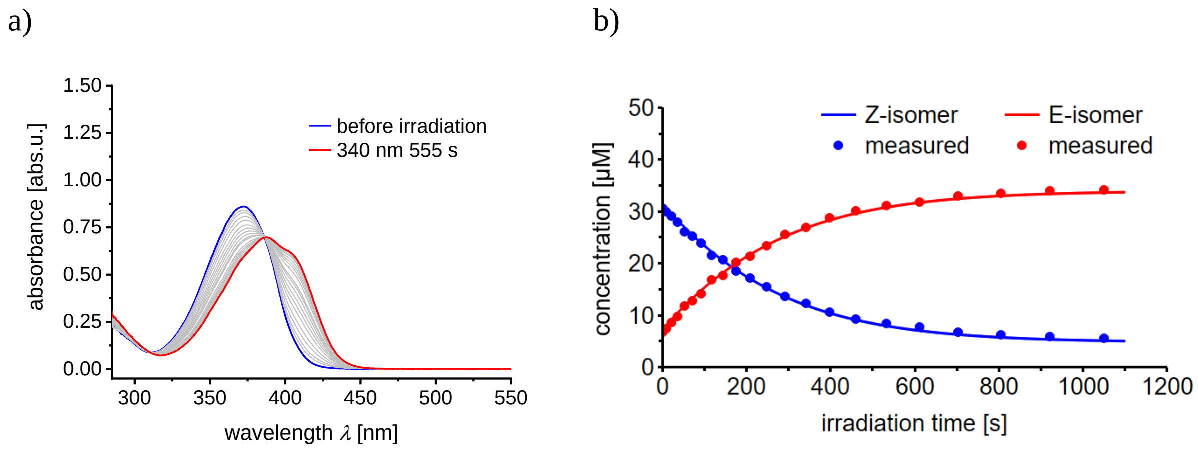

Supplementary Figure 141. Photoisomerization quantum yield determination of HPI **17** in toluene at 23 °C. a) UV/Vis absorption spectra recorded after each 340 nm irradiation step, starting with *Z*-isomer enriched solution. Irradiation was continued until the photostationary state (PSS) was reached. b) Measured and fitted changes (blue and red lines) in concentration of *Z* and *E* isomers during the photoreaction.

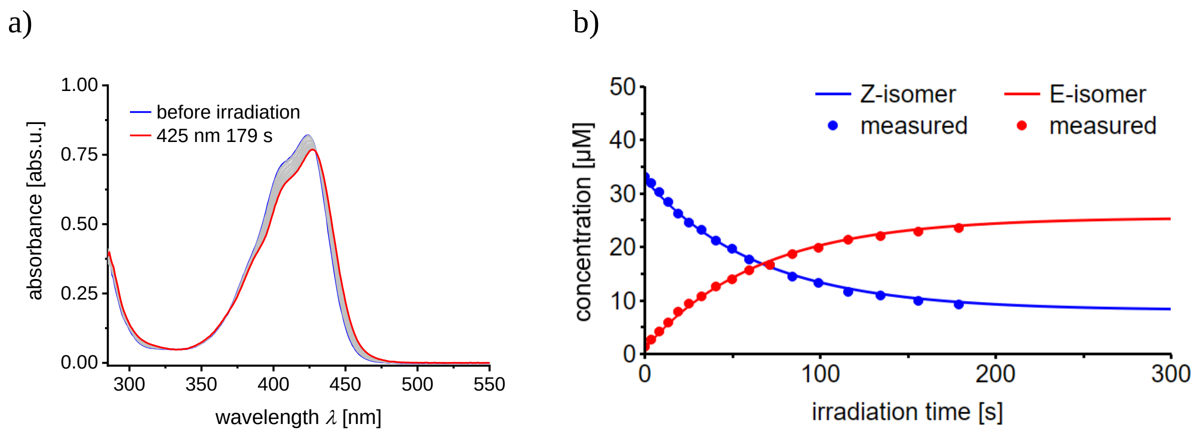

Supplementary Figure 142. Photoisomerization quantum yield determination of HPI **18** in toluene at 23 °C. a) UV/Vis absorption spectra recorded after each 425 nm irradiation step, starting with pure *Z*-isomer. Irradiation was continued until the photostationary state (PSS) was reached. b) Measured and fitted changes (blue and red lines) in concentration of *Z* and *E* isomers during the photoreaction.

Supplementary Note 14: Error determination

The order of magnitude of the error in the measurement and calculation of the molar absorption coefficients, isomer concentrations during NMR irradiations, and thermal stabilities was determined for HPI **3**. For all measurements, 3 different samples were prepared as described in the chapters above and the sample standard deviation *s* was calculated using equation (19).

$$s = \sqrt{\frac{\sum(x_i - \bar{x})^2}{n-1}} \tag{19}$$

With:

x_i = data point *i*

$\bar{x}$ = mean of the data

n = sample set size (number of measurements)

The results are summarized in the following figures and tables. The errors for the molar absorption coefficients (± 1-3%) and the isomer concentrations during NMR irradiation are typically low (± 0-1%), while the values for the thermal isomerization barriers have larger errors, most probably due to the varying water content in the deuterated toluene. All measurements display reasonably small errors, hence the conclusions drawn from the experimental data are consistent. For this reason, we assume similar small errors for all other measurements of all other HPIs.

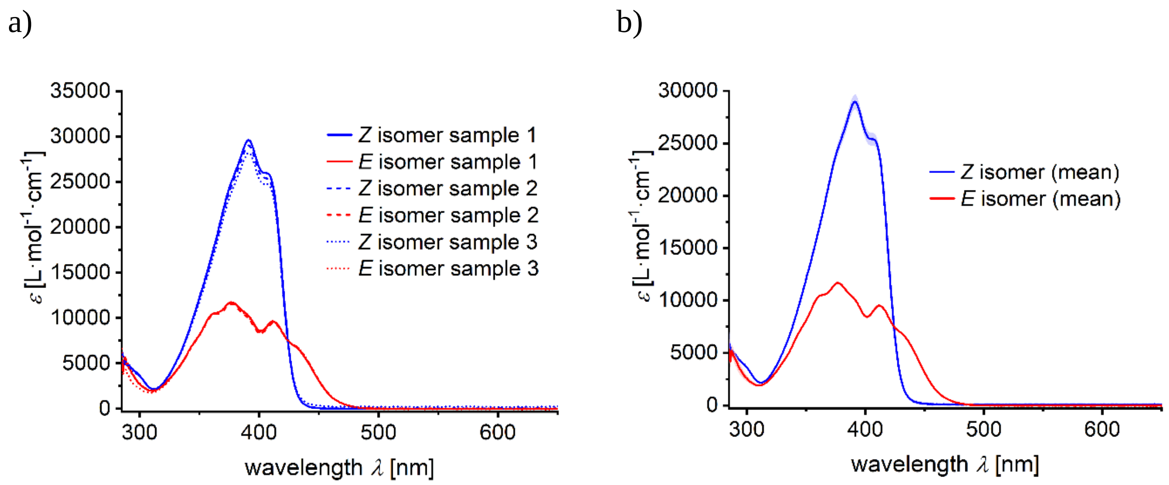

Supplementary Figure 143. Experimentally determined molar absorption coefficients for the pure *Z* (blue) and *E* (red) isomers of HPI **3** in toluene at 23 °C. a) Molar absorption coefficients for both isomers determined from three different samples. b) Corresponding mean molar absorption coefficients for both isomers, the blue and red shadows represent the sample standard deviation. Errors are in the range of 1-3%.

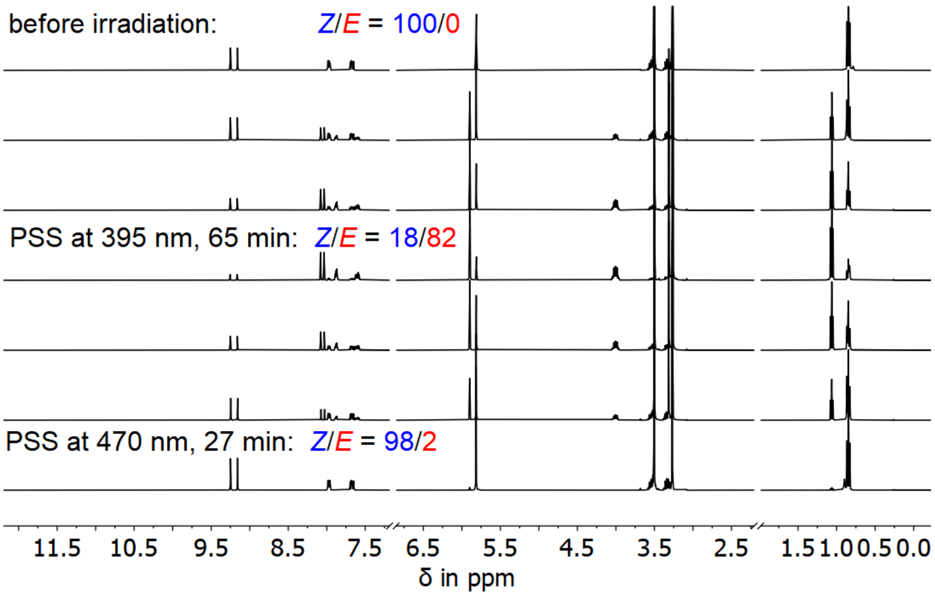

Supplementary Figure 144. Exemplary photoconversion experiments for the first sample of HPI **3** upon irradiation with 395 nm and 470 nm light. ^{1}H NMR spectra (400 MHz, toluene-d_8, 23 °C) of HPI **3** are recorded before and after irradiation using 395 nm and 470 nm light. The NMR spectra are shown in the order of the experiments from top to bottom and isomeric composition of *Z* and *E* isomer at PSS are indicated. Unlabeled NMR spectra illustrate the isomer enrichment before reaching the PSS. The C-H signals at 5.80 and 5.89 ppm were used for integration.

Supplementary Table 11. Determined isomer concentrations of HPI **3** during irradiation with 395 nm and 470 nm light of three different samples. The isomerization reactions were followed with ^{1}H NMR spectroscopy (e.g. Supplementary Figure 144). The sample standard deviation was calculated relative to the average isomer concentrations.

Sample Number	Solvent	[*E*] at PSS [%] (395 nm)	Average [*E*] at PSS [%] (395 nm)	Sample standard deviation [%]	[*Z*] at PSS [%] (470 nm)	Average [*Z*] at PSS [%] (470 nm)	Sample standard deviation [%]
**1**	toluene	81.9			97.5		
**2**	toluene	82.6	82.3	0.4%	98.9	98.5	0.9%
**3**	toluene	82.4			99.0		

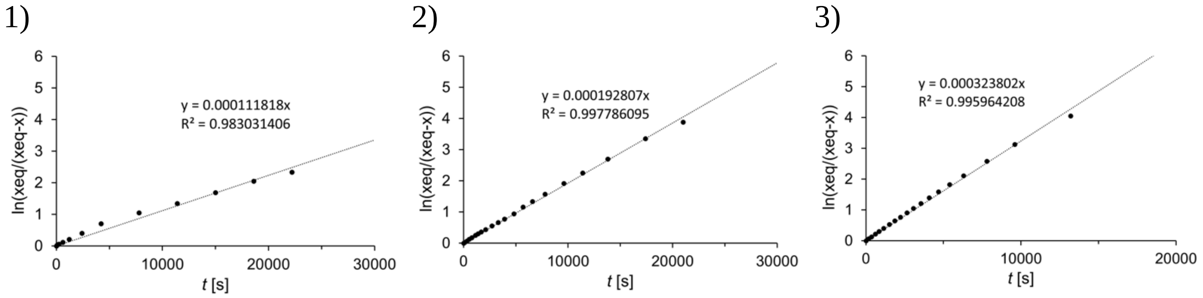

Supplementary Figure 145. Thermal *E* to *Z* isomerization of HPI **3** in toluene-d_8 in the dark starting from *E* enriched solution, which was obtained *via* irradiation with 395 nm light. The first order kinetic analysis of the thermal isomerization of *E* to *Z* proceeding towards a thermal equilibrium for three different samples (1, 2 and 3) are shown.

Supplementary Table 12. Thermal isomerization behavior of 3 different samples of HPI **3** in toluene-d_8 at 100 °C. The values for the *Gibbs* energies of activation for the *E* to *Z* isomerization were determined, averaged and the sample standard deviation was calculated relative to the average energy of activation.

Sample Number	Temperature T [°C]	Half-life of *E* isomer at indicated T	k_E [s^{-1}] at indicated T	$\Delta G^{‡}_{E\rightarrow Z}$ [kcal/mol] at indicated T	Average $\Delta G^{‡}_{E\rightarrow Z}$ [kcal/mol] at indicated T	$\Delta G^{‡}_{E\rightarrow Z}$ sample standard deviation [kcal/mol]	Half-life range of *E* isomer at 298 K (lowest and highest possible value)
**1**	100 °C	1.7 h	1.07×10^{-4}	28.8			
**2**	100 °C	1.0 h	1.87×10^{-4}	28.4	28.4	0.35	1.3-4.3 a
**3**	101 °C	0.6 h	3.14×10^{-4}	28.1			

Supplementary Note 15: NMR-spectra

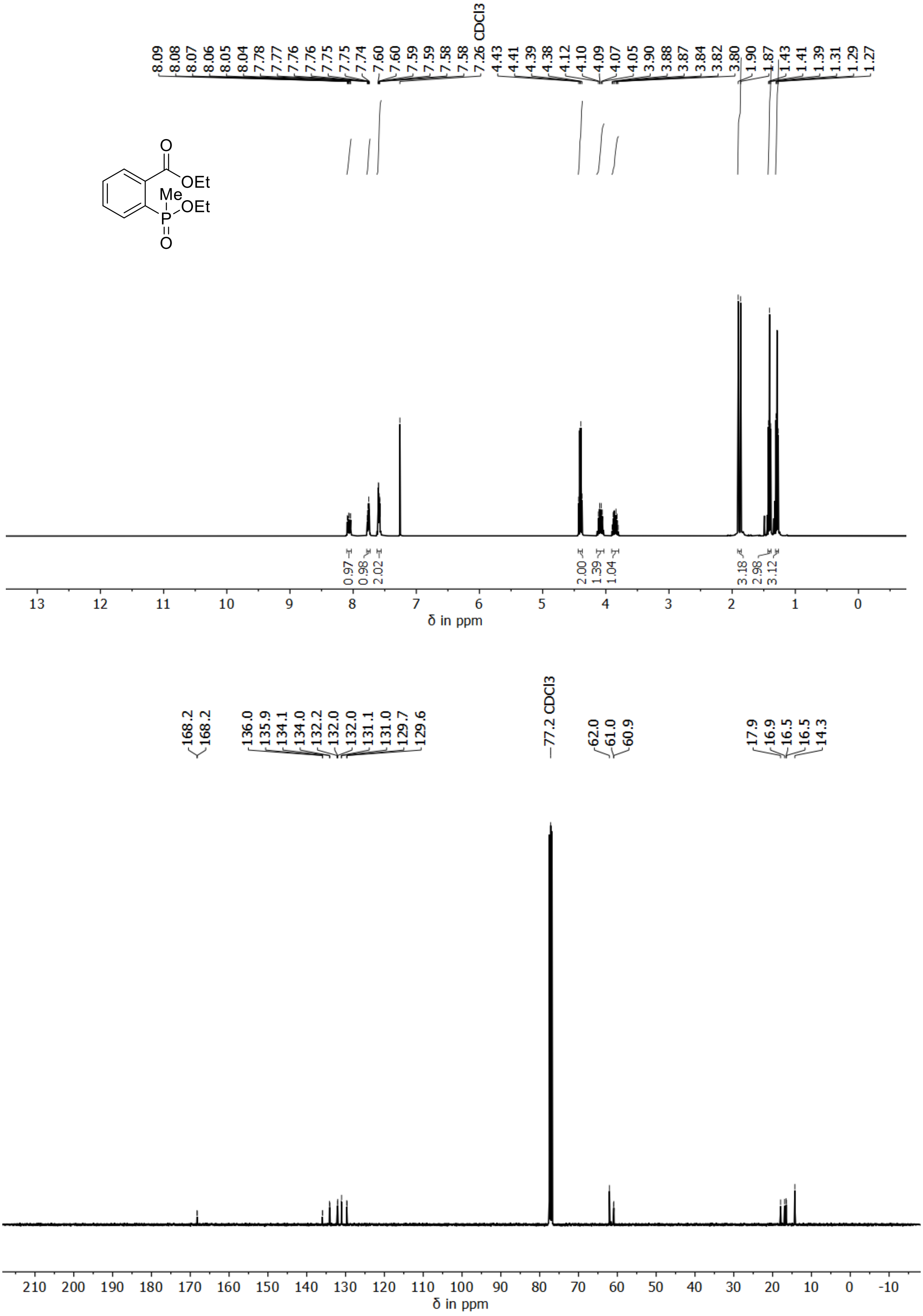

Supplementary Figure 146. NMR spectra of **19** in $CDCl_3$ at 25 °C. (top) 400 MHz ^1H NMR spectrum. (bottom) 101 MHz ^{13}C NMR spectrum.

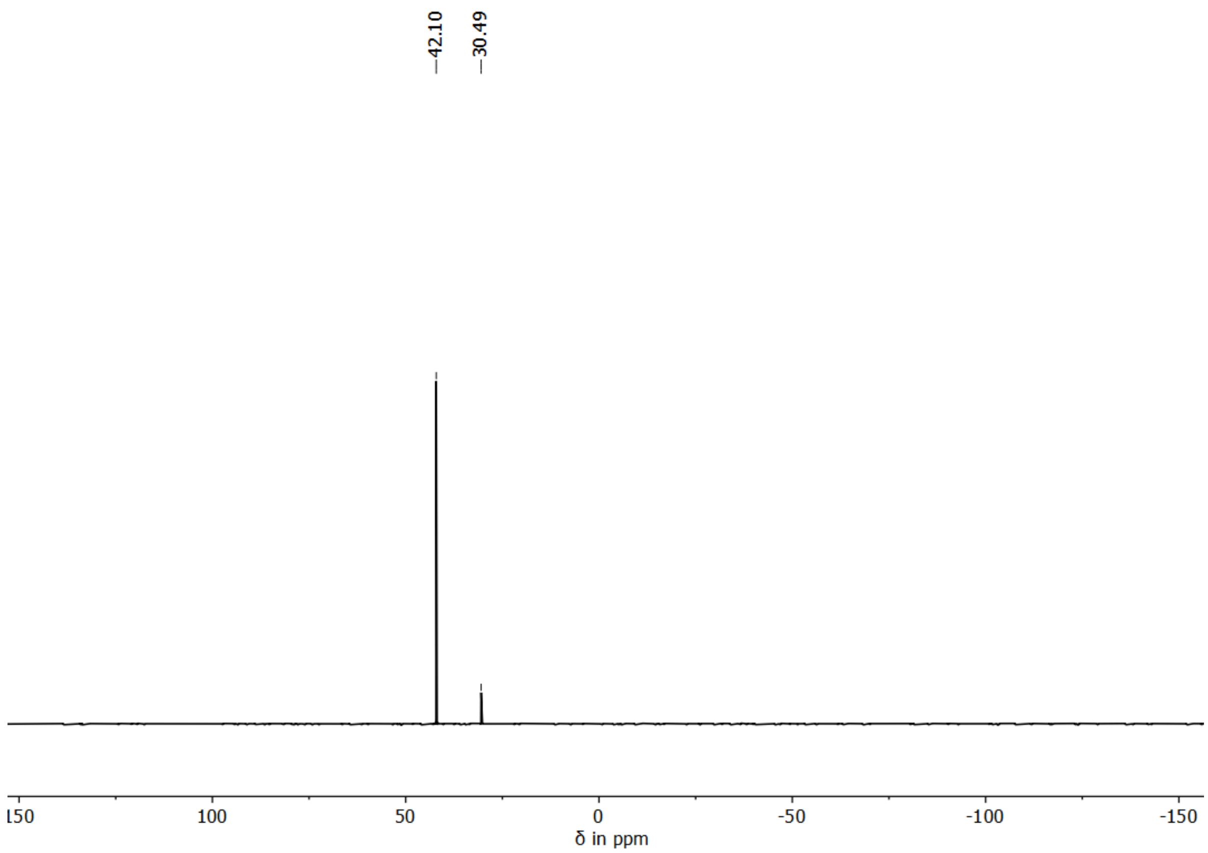

Supplementary Figure 147. 162 MHz ^{31}P NMR spectrum of **19** in $CDCl_3$ at 25 °C (Impurity at 30.49 ppm).

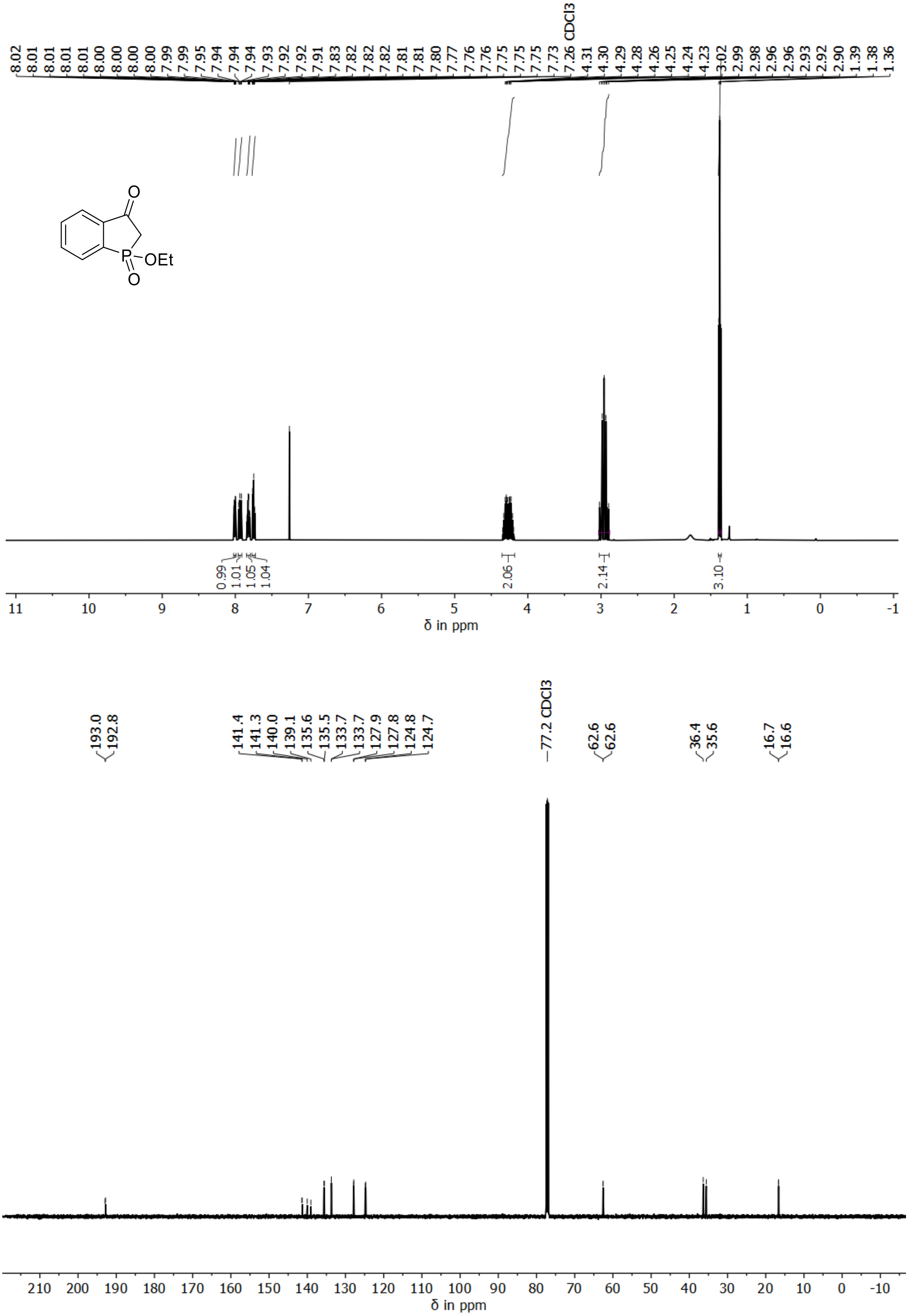

Supplementary Figure 148. NMR spectra of **20** in $CDCl_3$ at 25 °C. (top) 500 MHz ^1H NMR spectrum. (bottom) 126 MHz ^{13}C NMR spectrum.

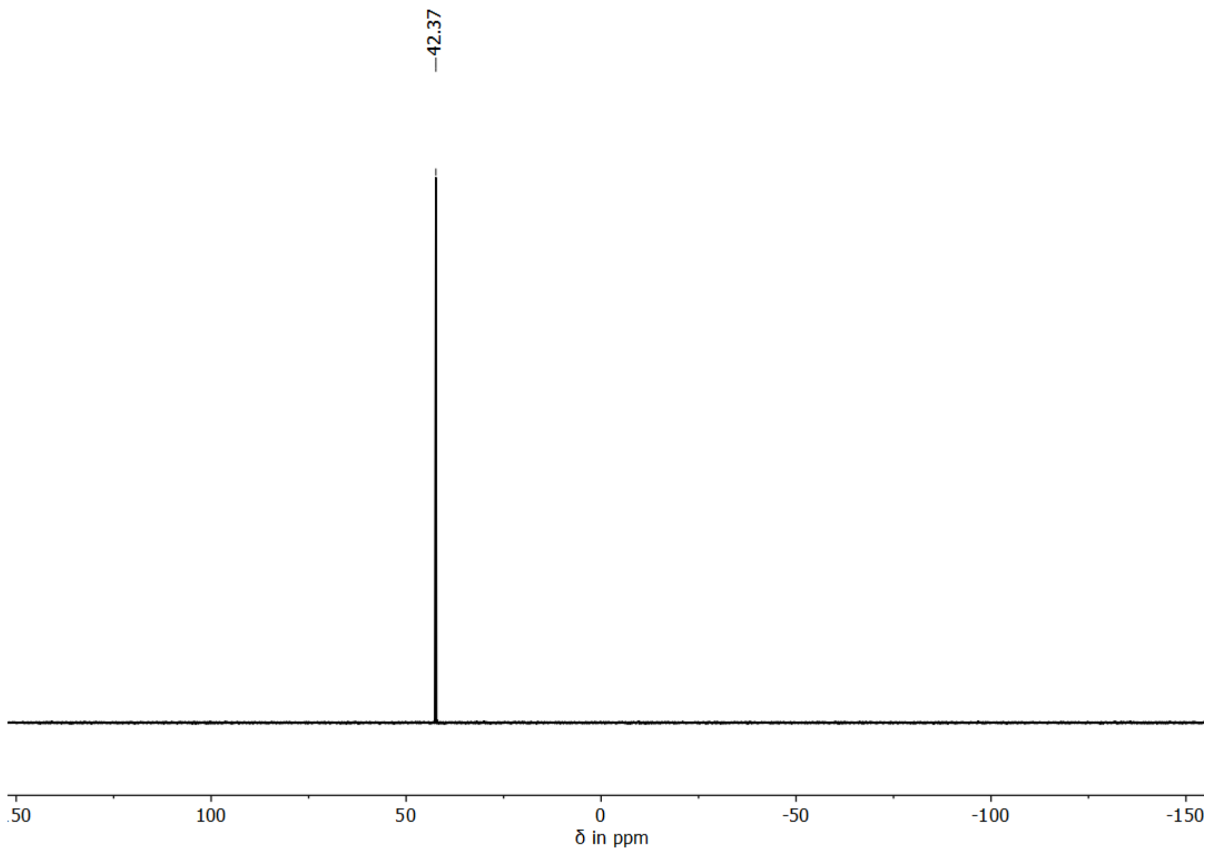

Supplementary Figure 149. 203 MHz ^{31}P NMR spectrum of **20** in $CDCl_3$ at 25 °C.

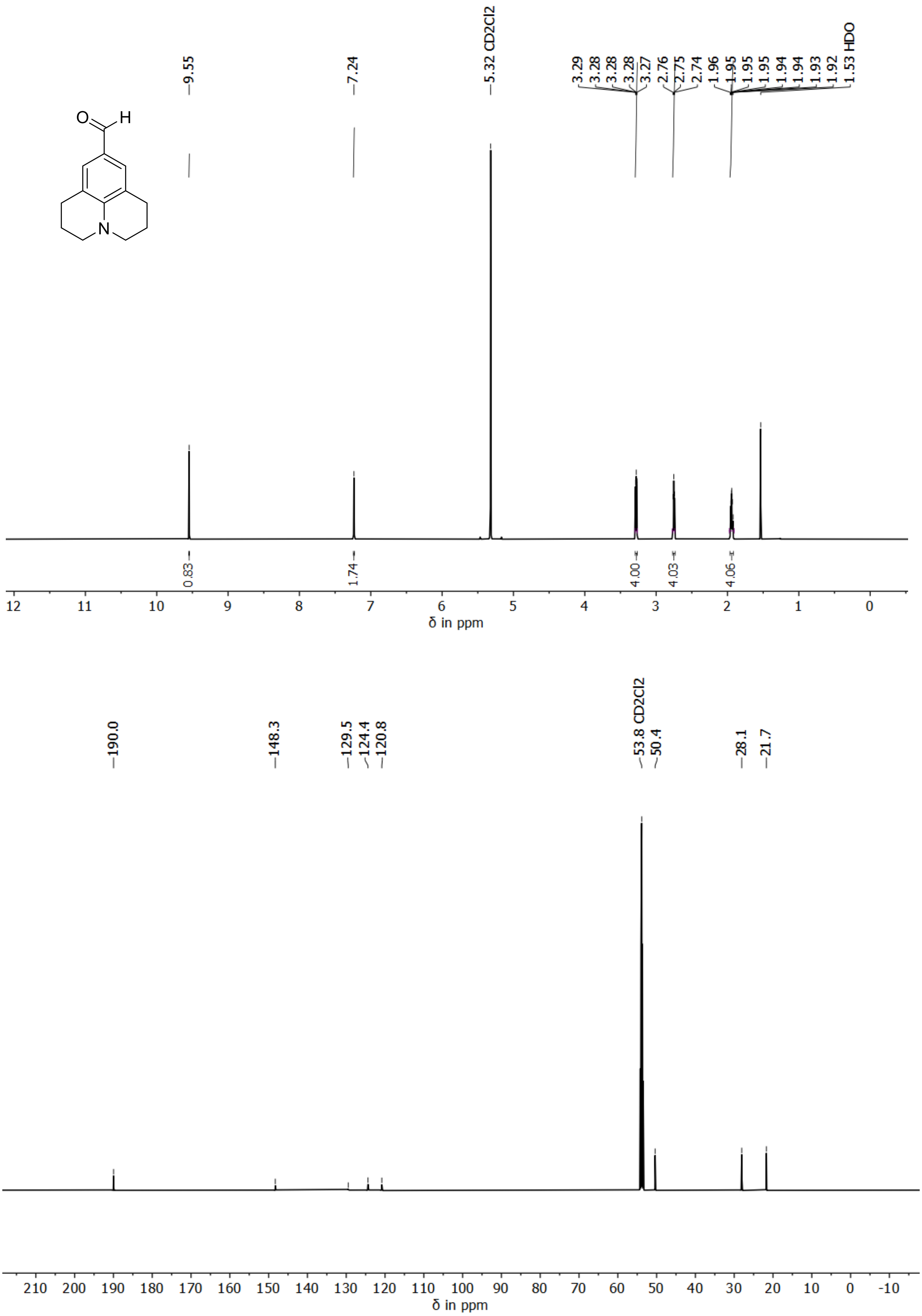

Supplementary Figure 150. NMR spectra of **24** in CD_2Cl_2 at 25 °C. (top) 600 MHz ^1H NMR spectrum. (bottom) 151 MHz ^{13}C NMR spectrum.

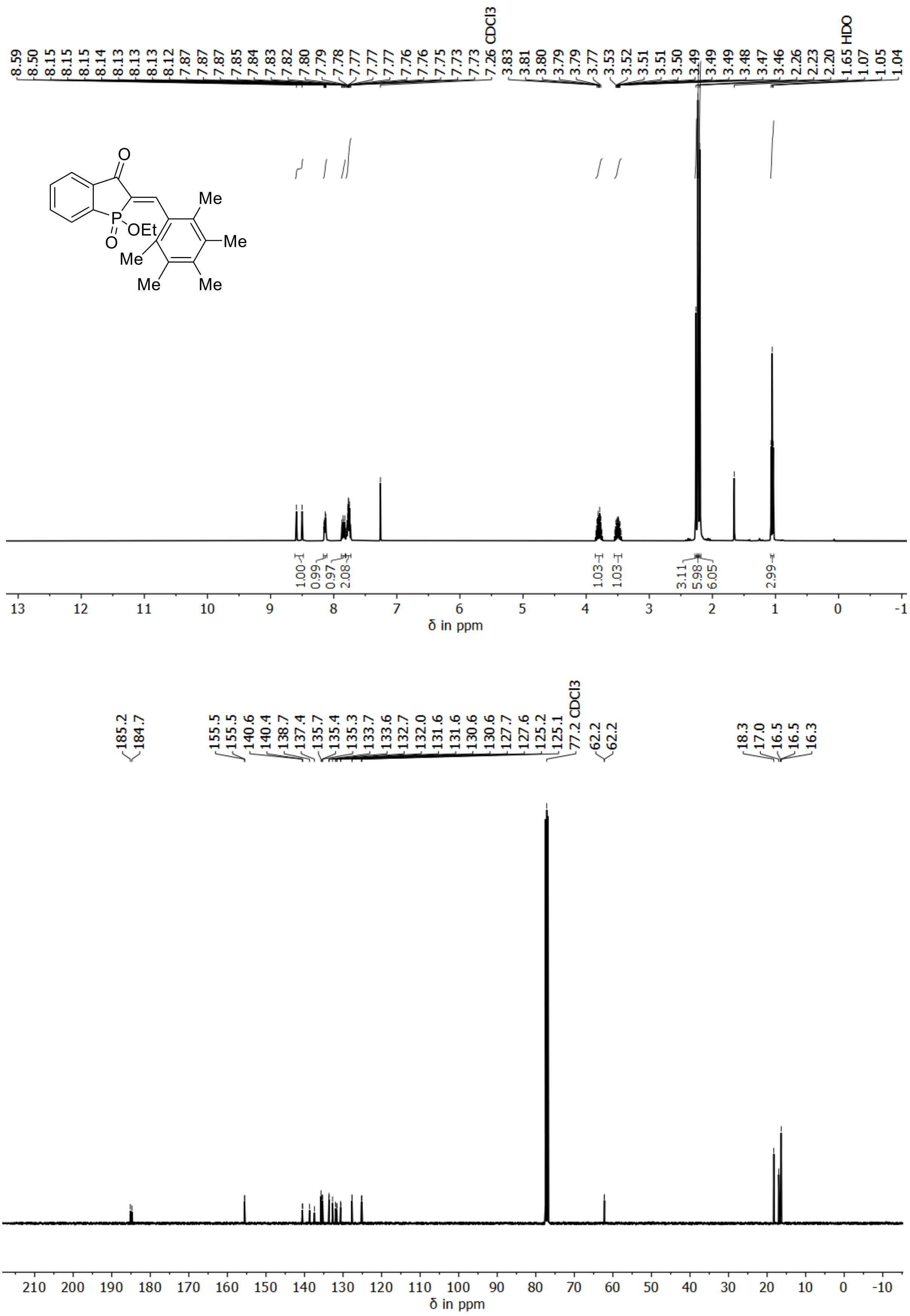

Supplementary Figure 151. NMR spectra of *Z*-**1** in $CDCl_3$ at 25 °C. (top) 400 MHz ^{1}H NMR spectrum. (bottom) 101 MHz ^{13}C NMR spectrum.

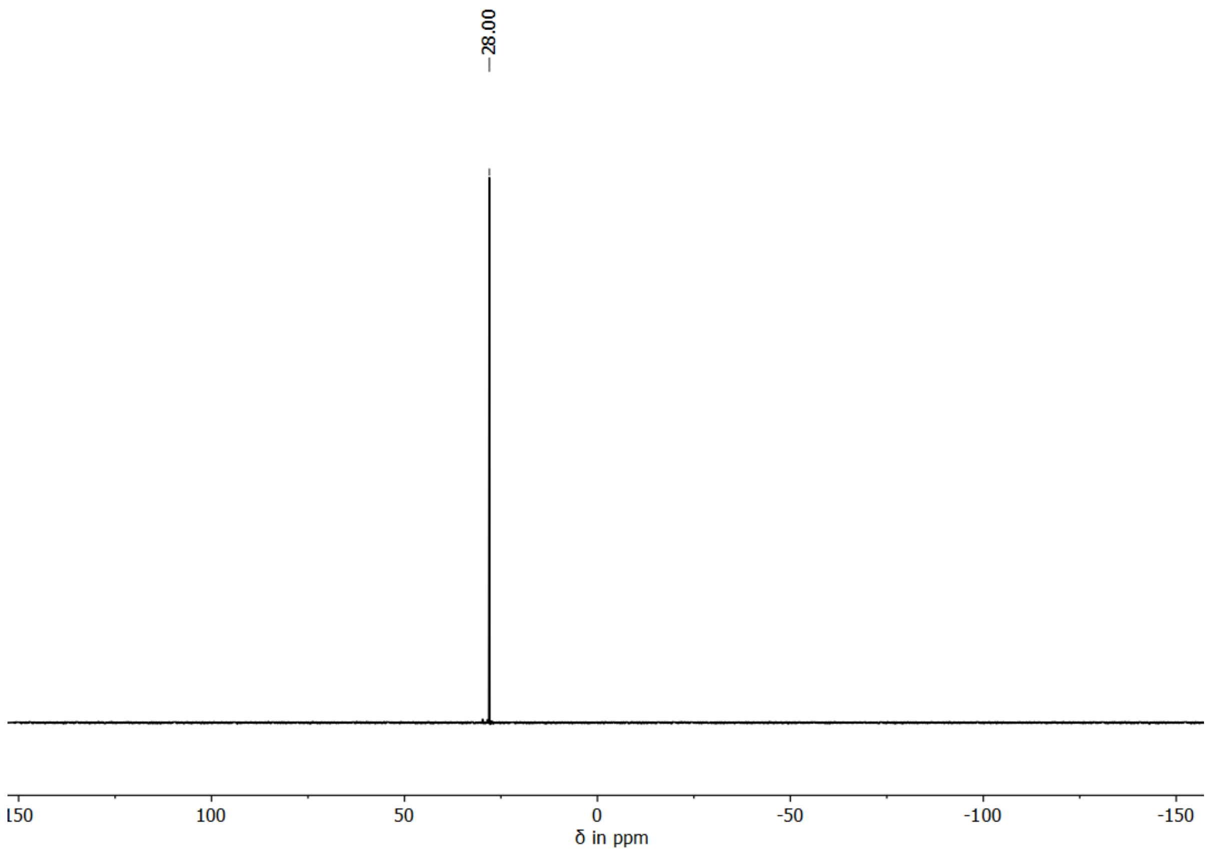

Supplementary Figure 152. 162 MHz ^{31}P NMR spectrum of *Z*-**1** in $CDCl_3$ at 25 °C.

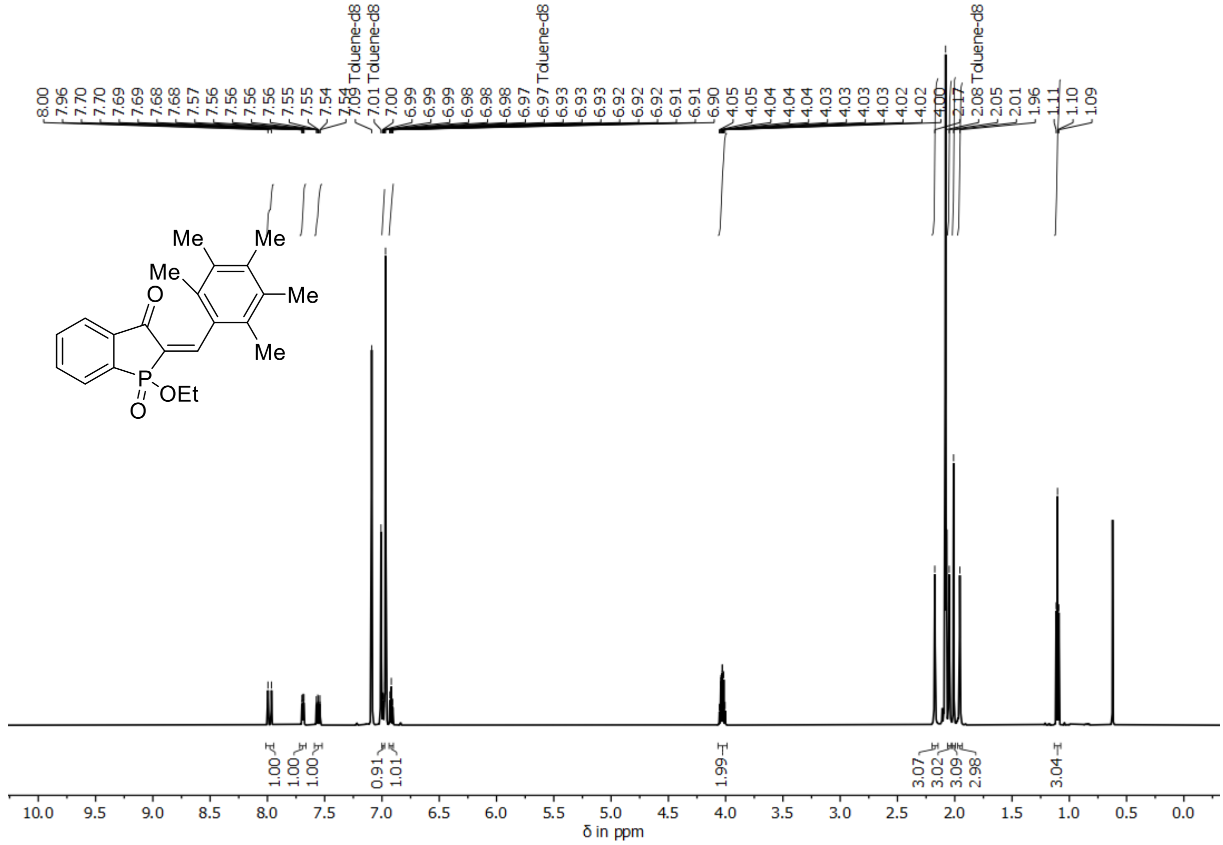

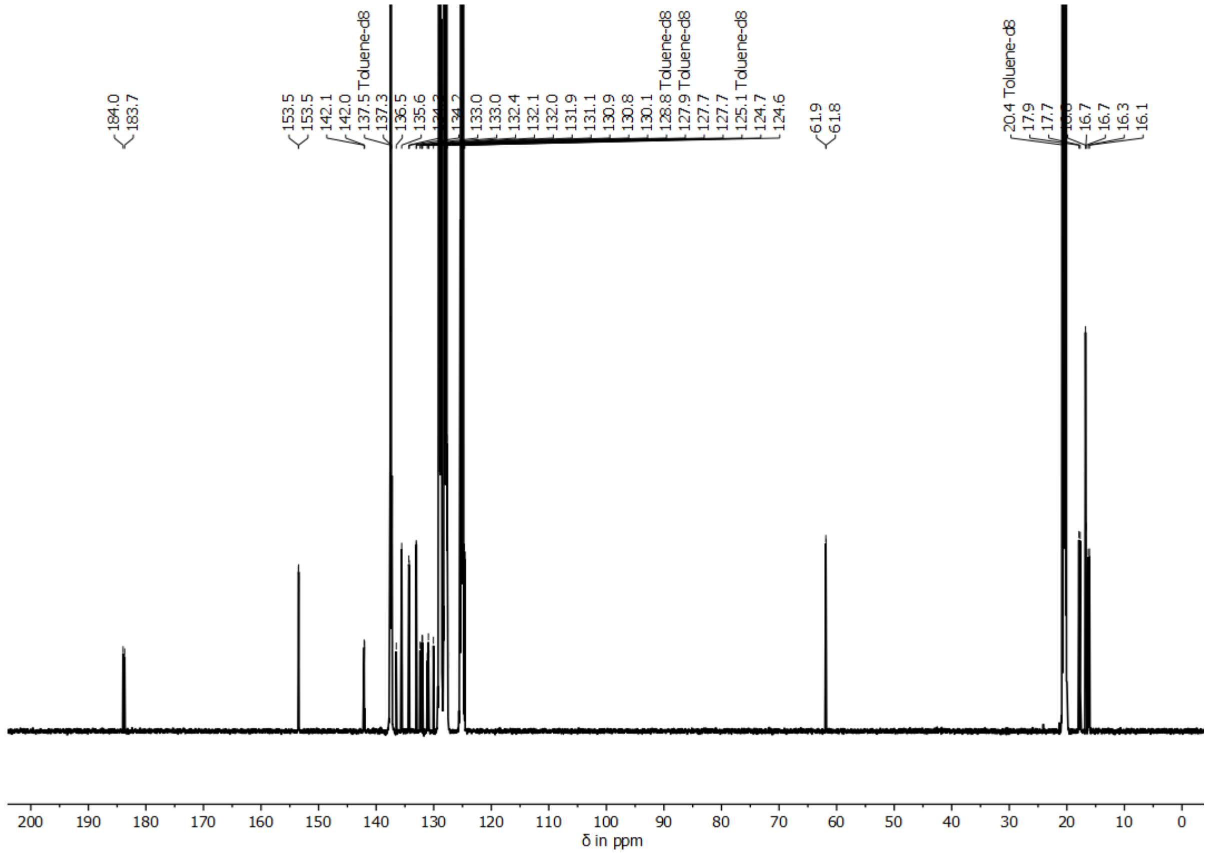

Supplementary Figure 153. NMR spectra of *E*-**1** in Tol-d_8 at 25 °C. (top) 600 MHz ^{1}H NMR spectrum. (bottom) 151 MHz ^{13}C NMR spectrum.

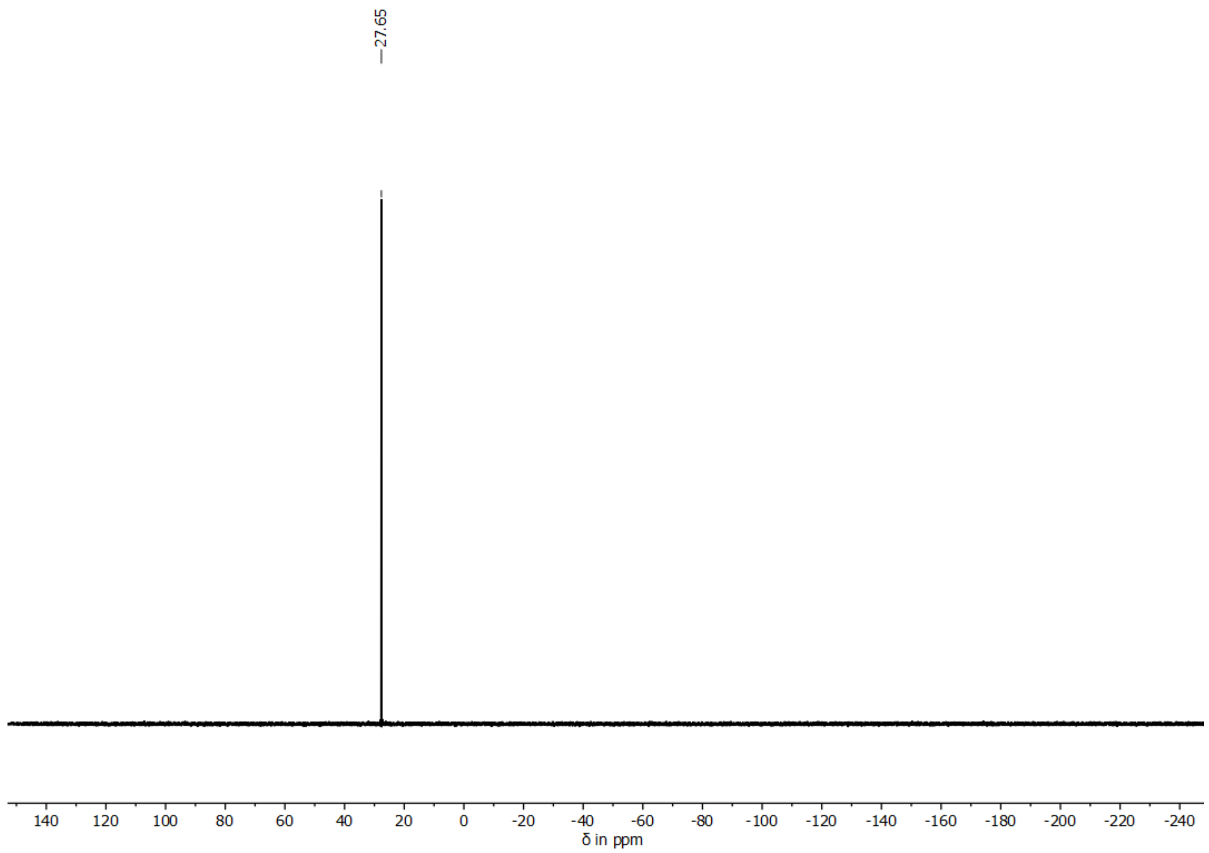

Supplementary Figure 154. 162 MHz ^{31}P NMR spectrum of *E*-**1** in $CDCl_3$ at 25 °C.

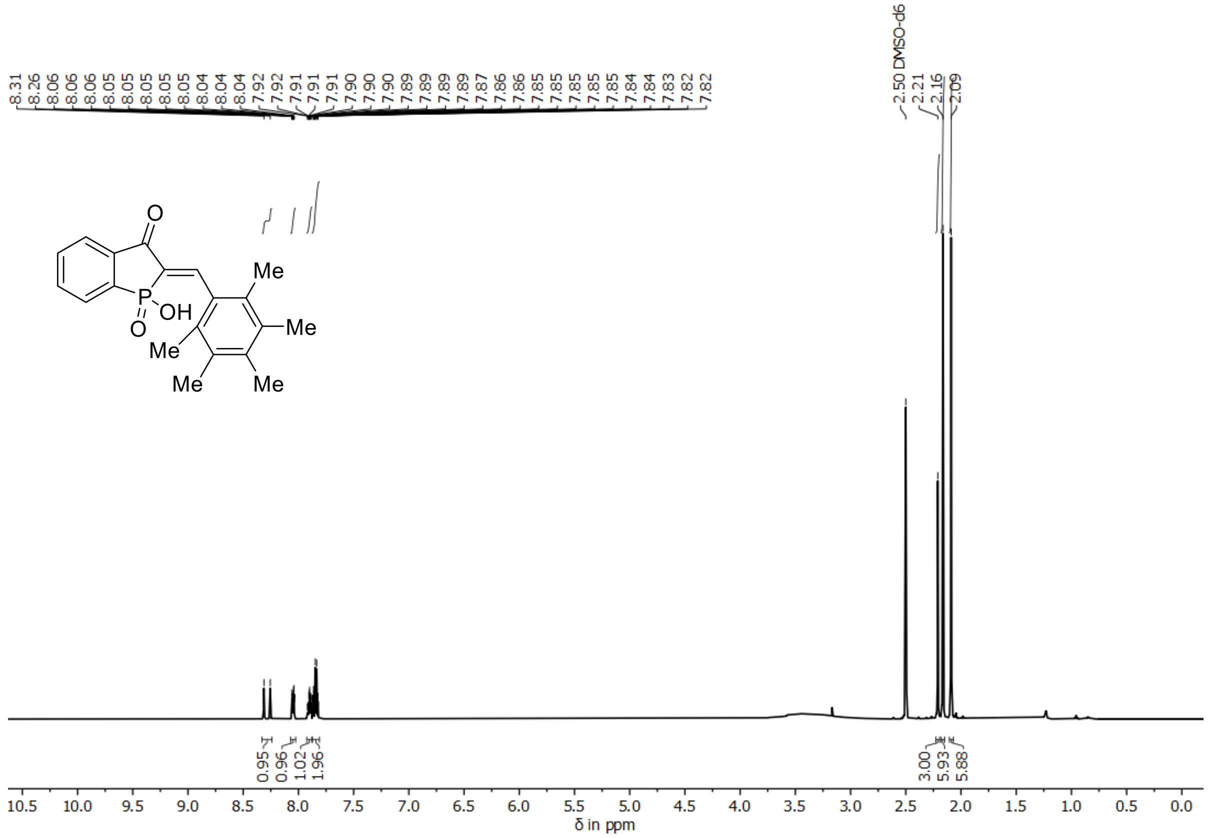

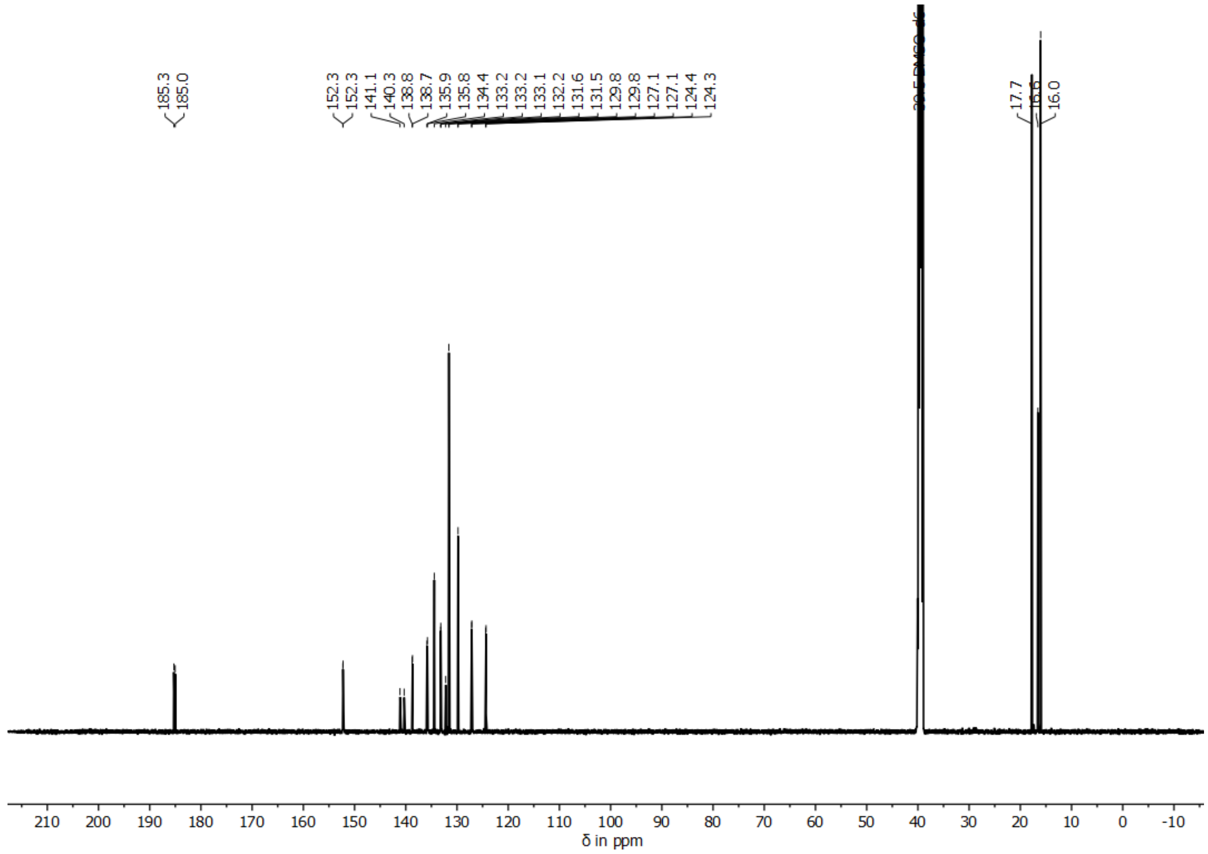

Supplementary Figure 155. NMR spectra of **1**-OH in DMSO-d_6 at 25 °C. (top) 600 MHz ^{1}H NMR spectrum. (bottom) 151 MHz ^{13}C NMR spectrum.

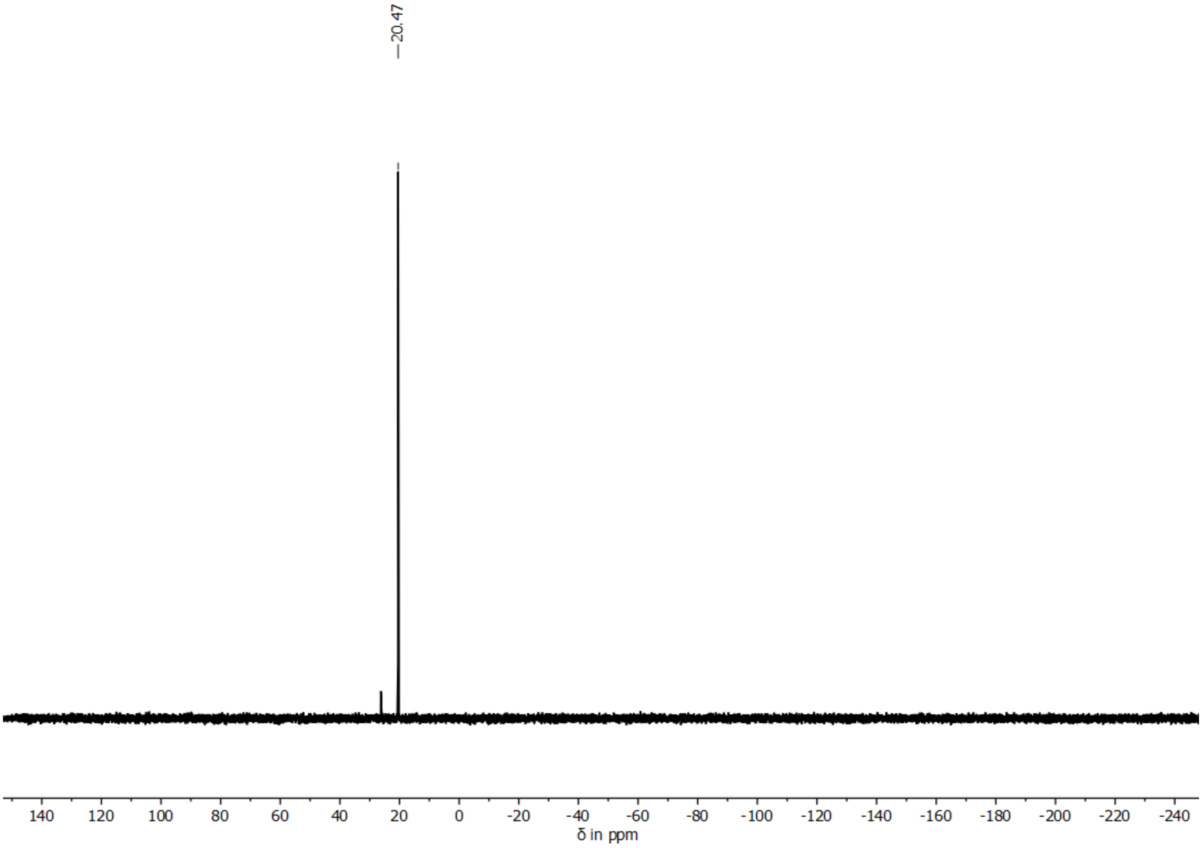

Supplementary Figure 156. 162 MHz ^{31}P NMR spectrum of **1**-OH in DMSO-d_6 at 25 °C.

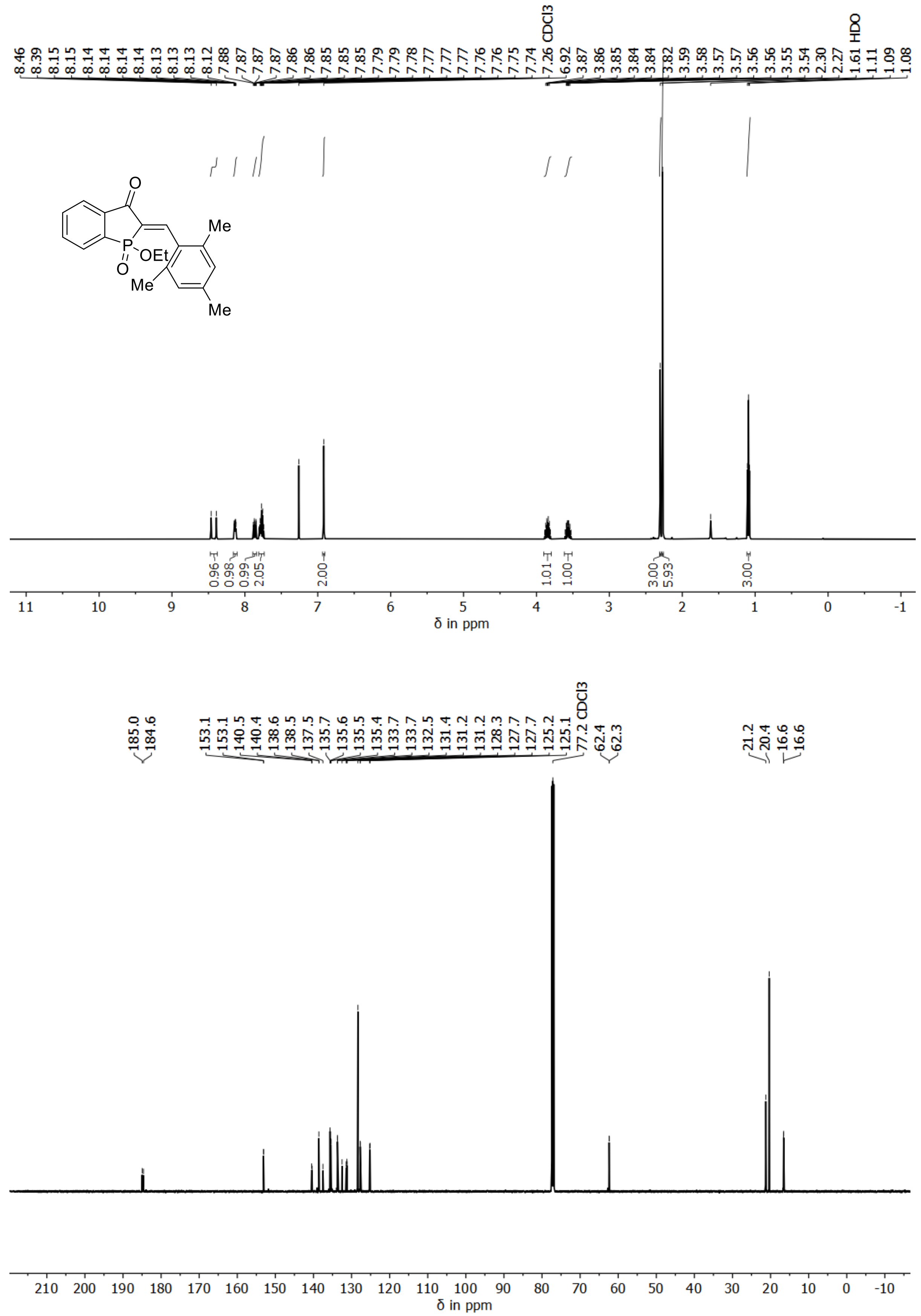

Supplementary Figure 157. NMR spectra of **2** in $CDCl_3$ at 25 °C. (top) 500 MHz ^1H NMR spectrum. (bottom) 126 MHz ^{13}C NMR spectrum.

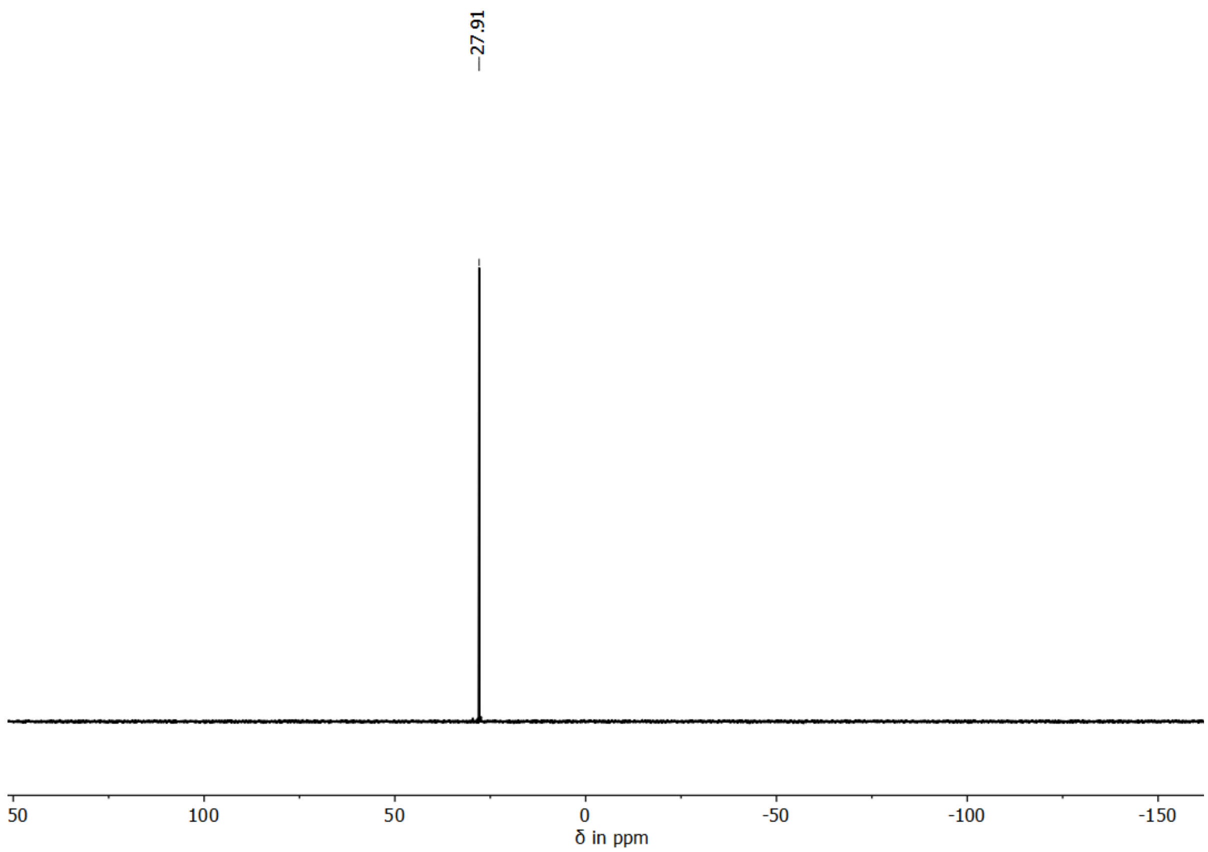

Supplementary Figure 158. 203 MHz ^{31}P NMR spectrum of **2** in $CDCl_3$ at 25 °C.

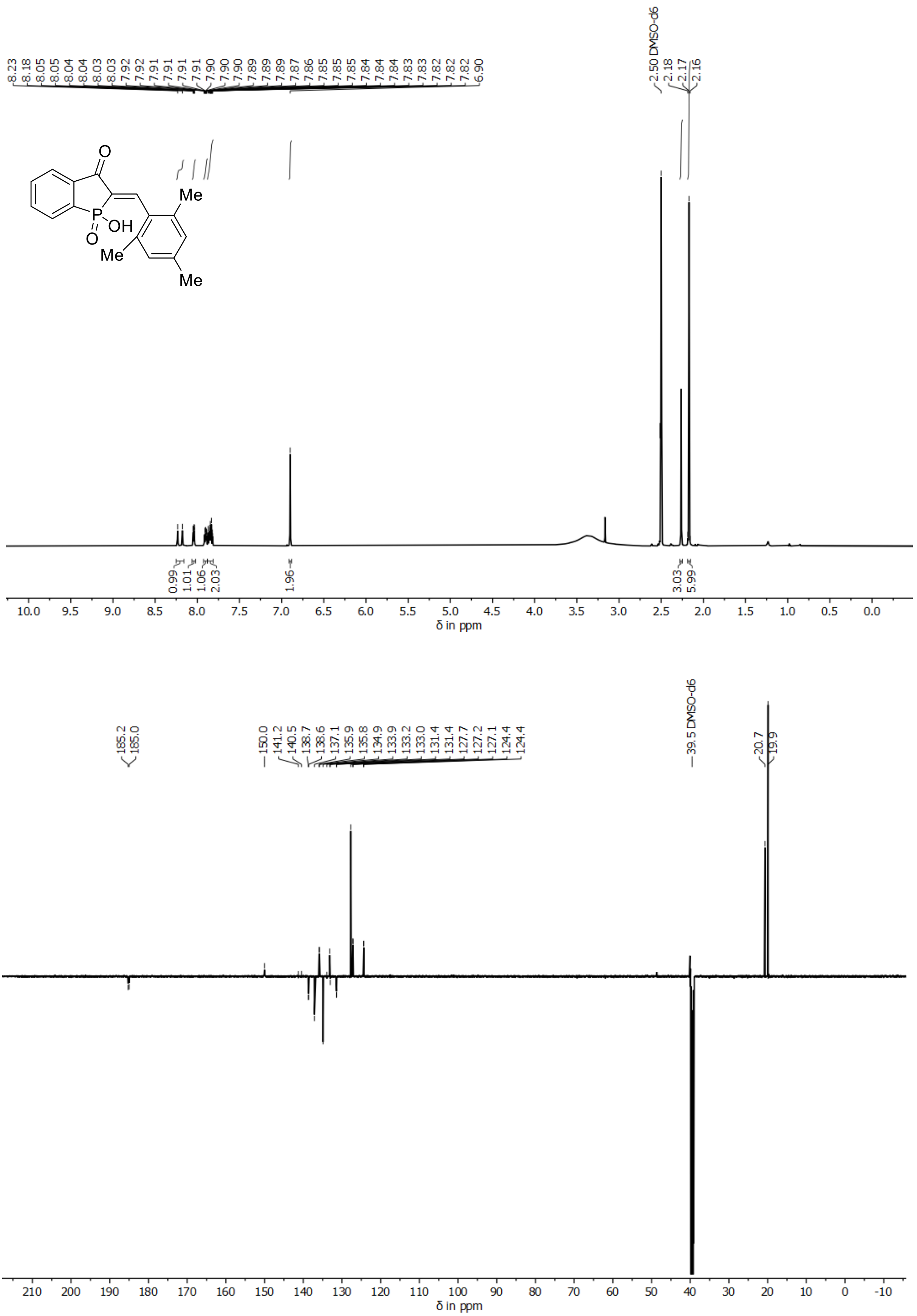

Supplementary Figure 159. NMR spectra of **2-**OH in DMSO-d_6 at 25 °C. (top) 600 MHz ^{1}H NMR spectrum. (bottom) 151 MHz DEPTq spectrum.

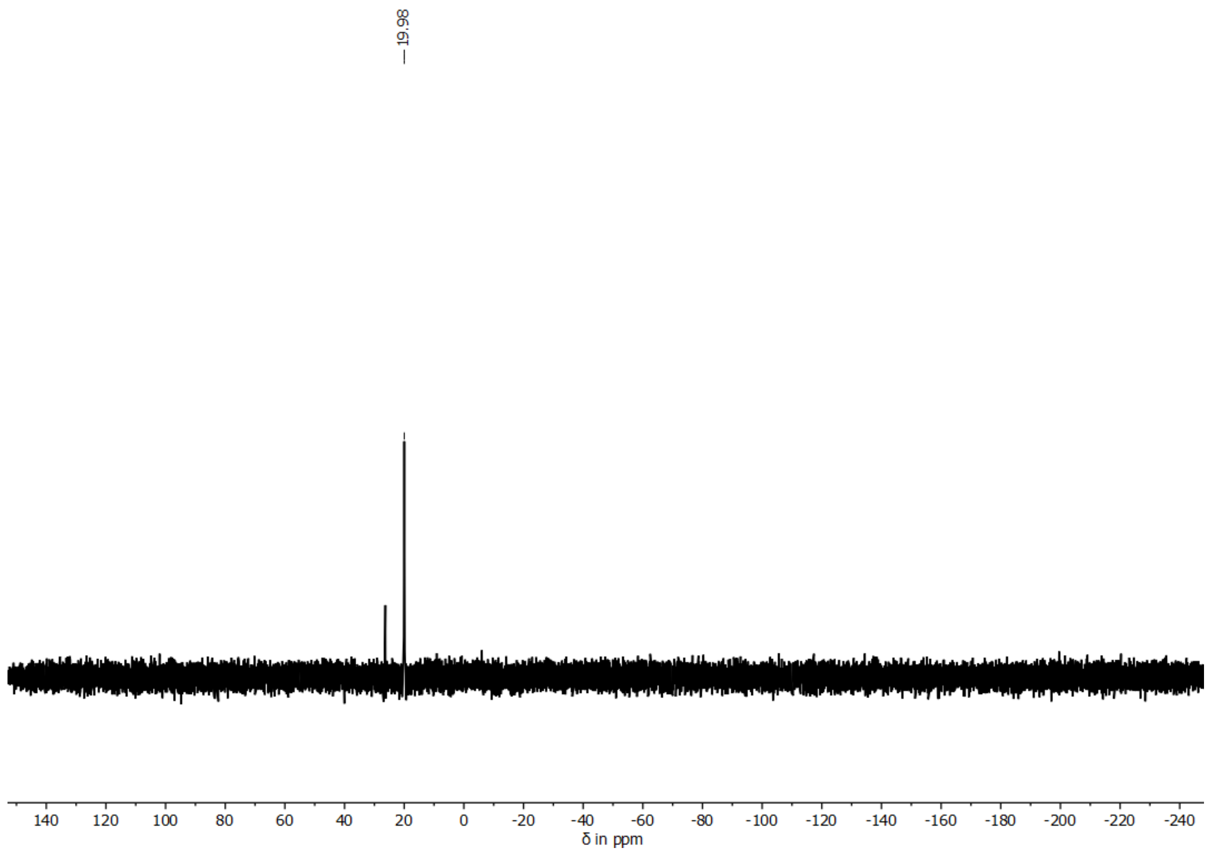

Supplementary Figure 160. 162 MHz ^{31}P NMR spectrum of **2**-OH in DMSO-d_6 at 25 °C.

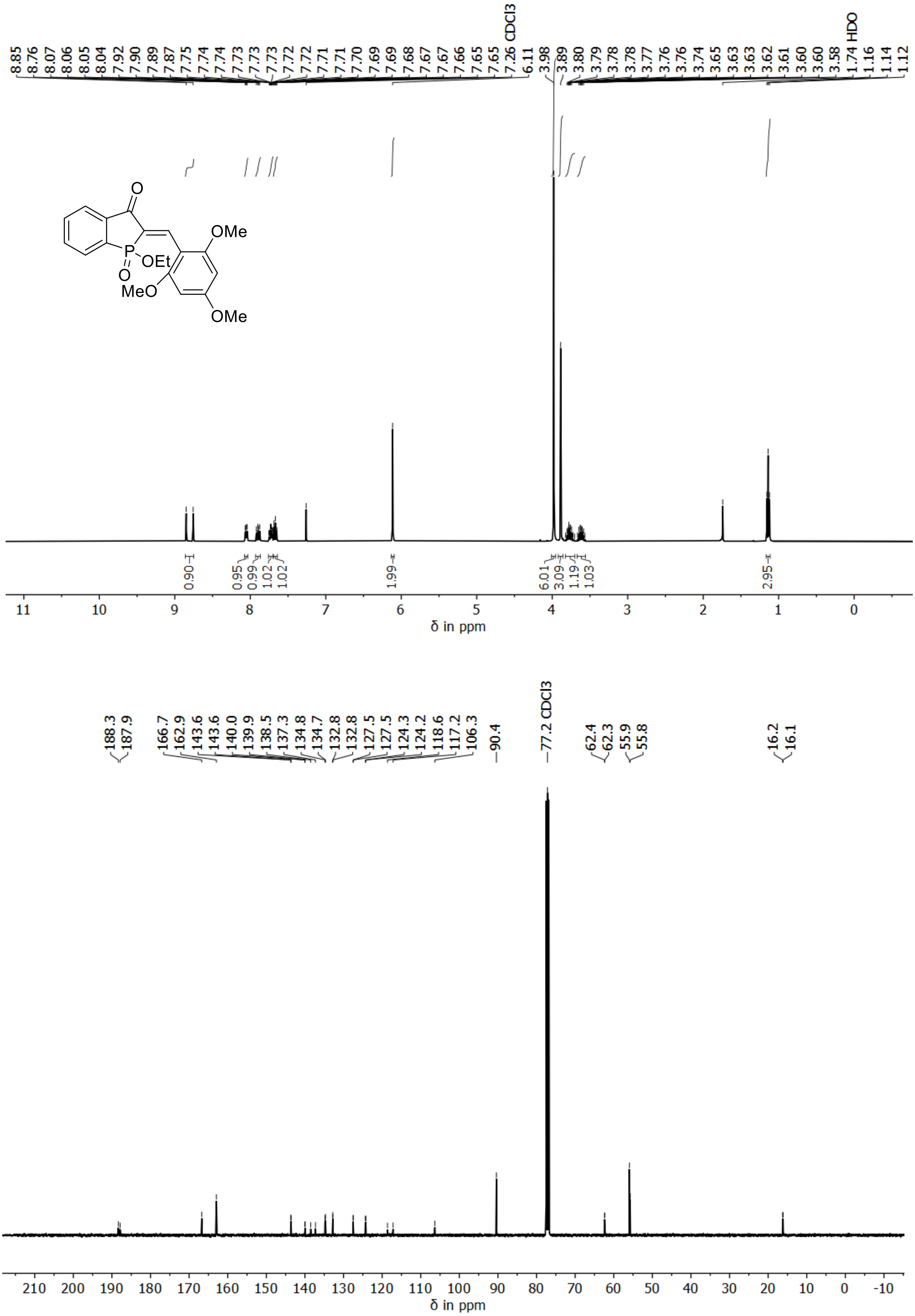

Supplementary Figure 161. NMR spectra of **3** in $CDCl_3$ at 25 °C. (top) 400 MHz ^1H NMR spectrum. (bottom) 101 MHz ^{13}C NMR spectrum.

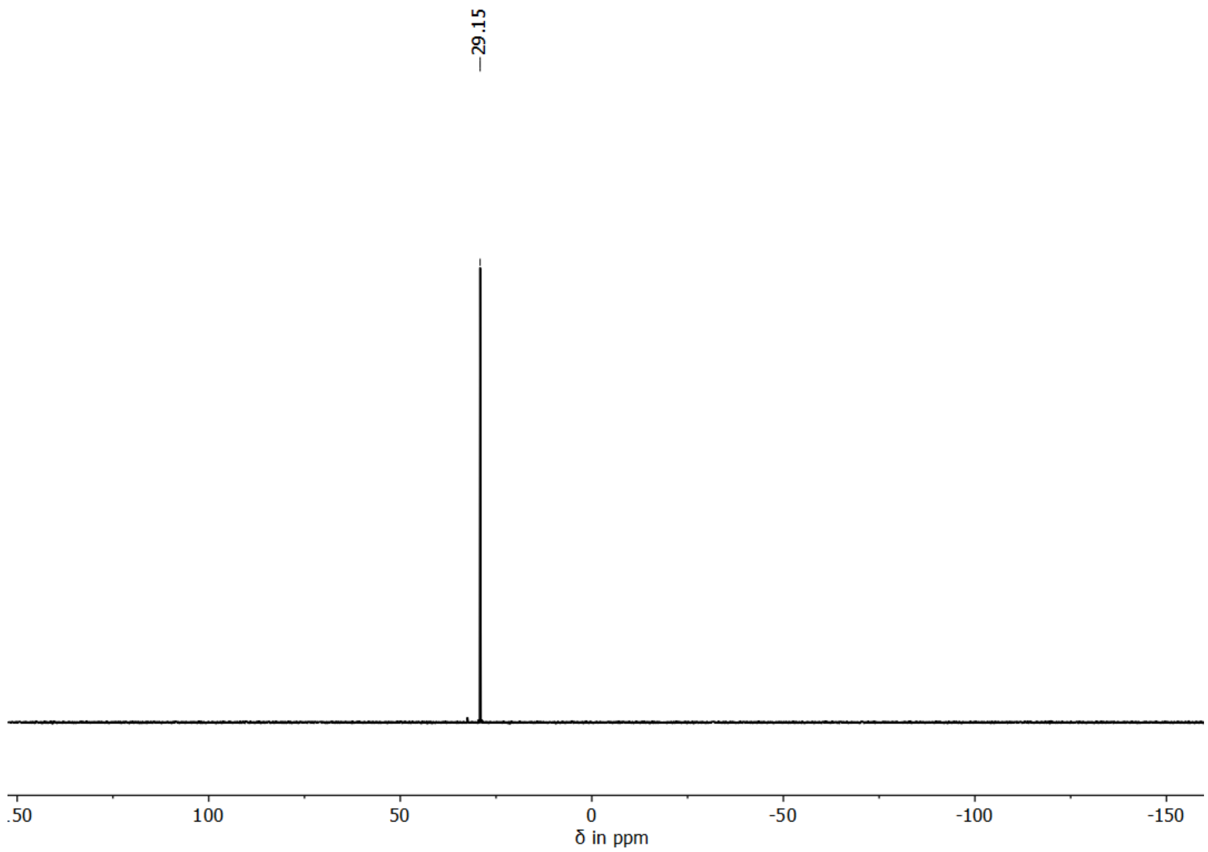

Supplementary Figure 162. 162 MHz ^{31}P NMR spectrum of **3** in $CDCl_3$ at 25 °C.

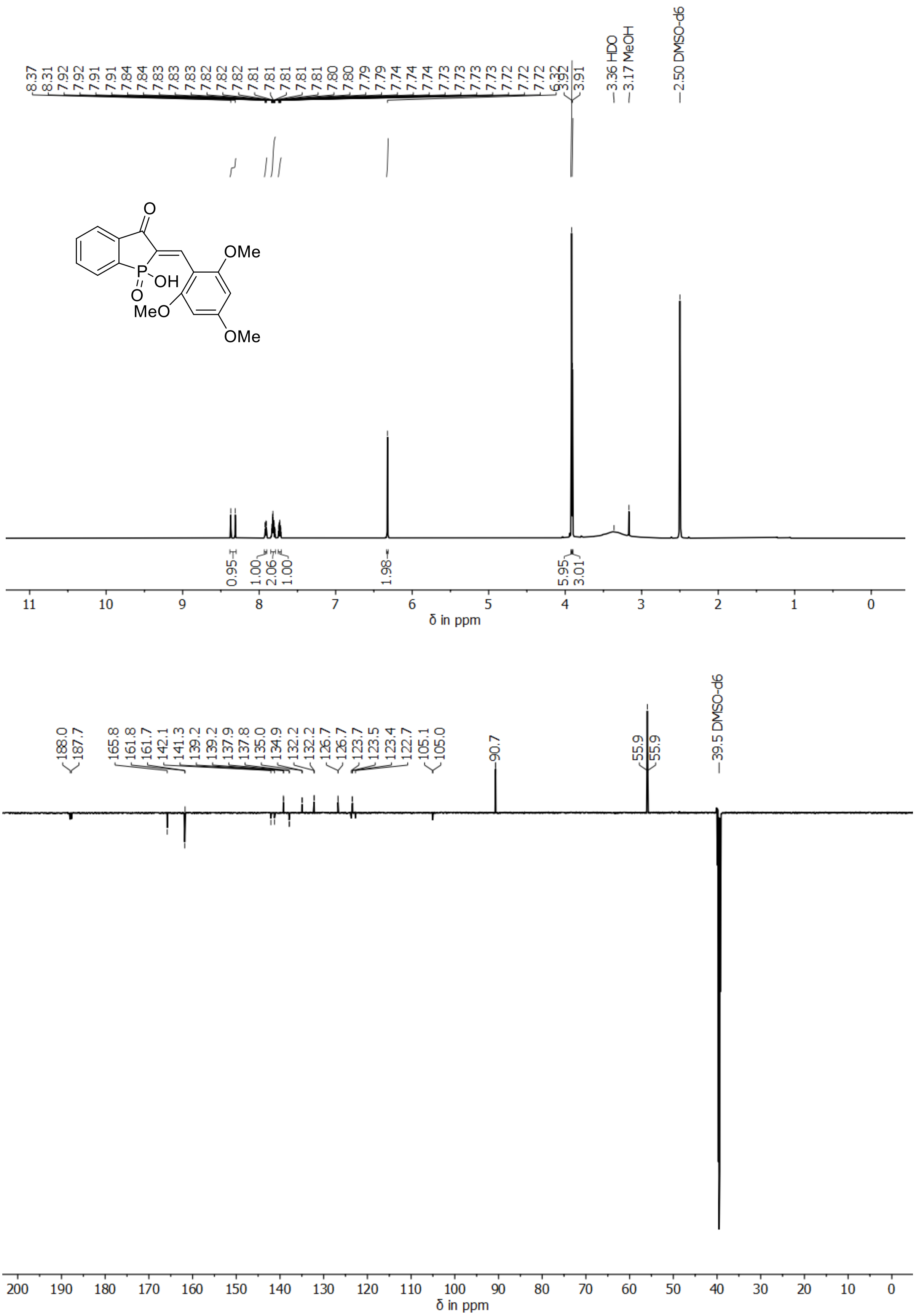

Supplementary Figure 163. NMR spectra of **3-**OH in DMSO-d_6 at 25 °C. (top) 600 MHz ^{1}H NMR spectrum. (bottom) 151 MHz DEPTq spectrum.

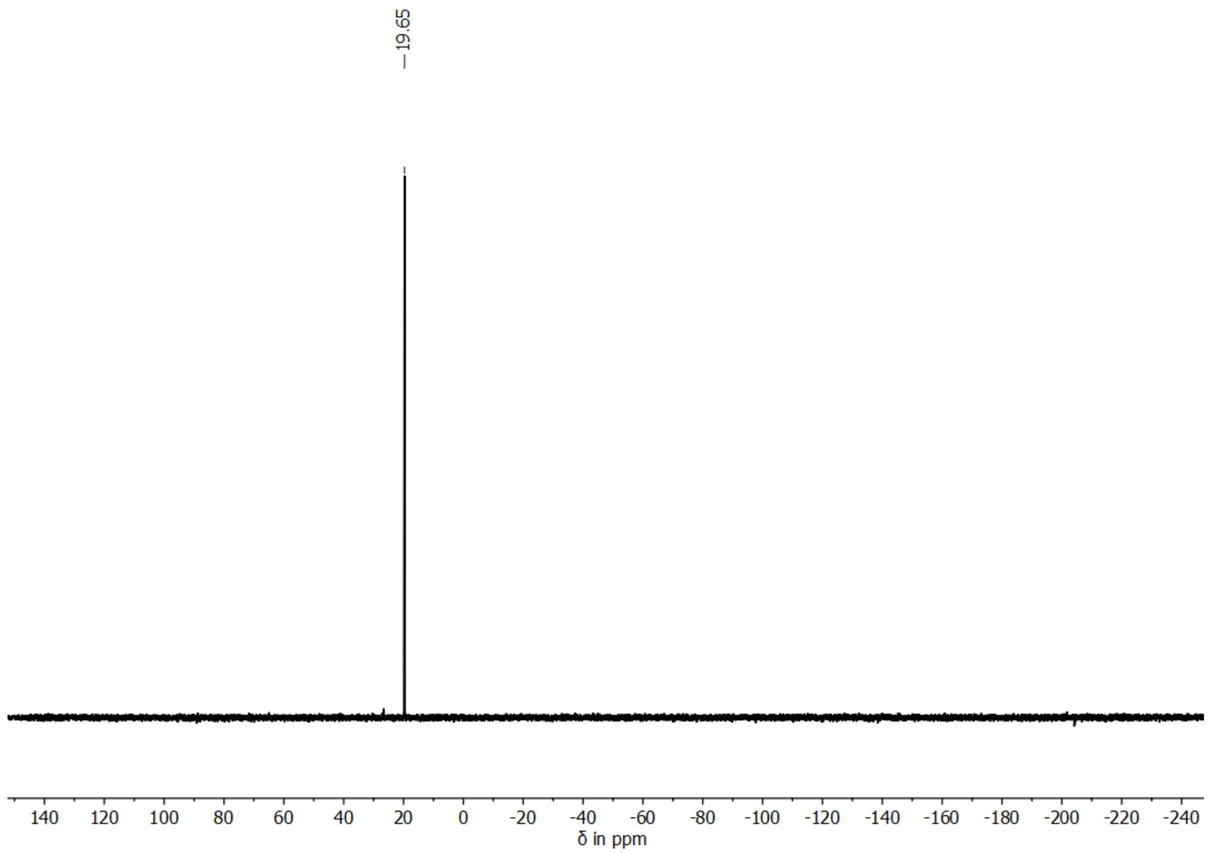

Supplementary Figure 164. 203 MHz ^{31}P NMR spectrum of **3**-OH in DMSO-d_6 at 25 °C.

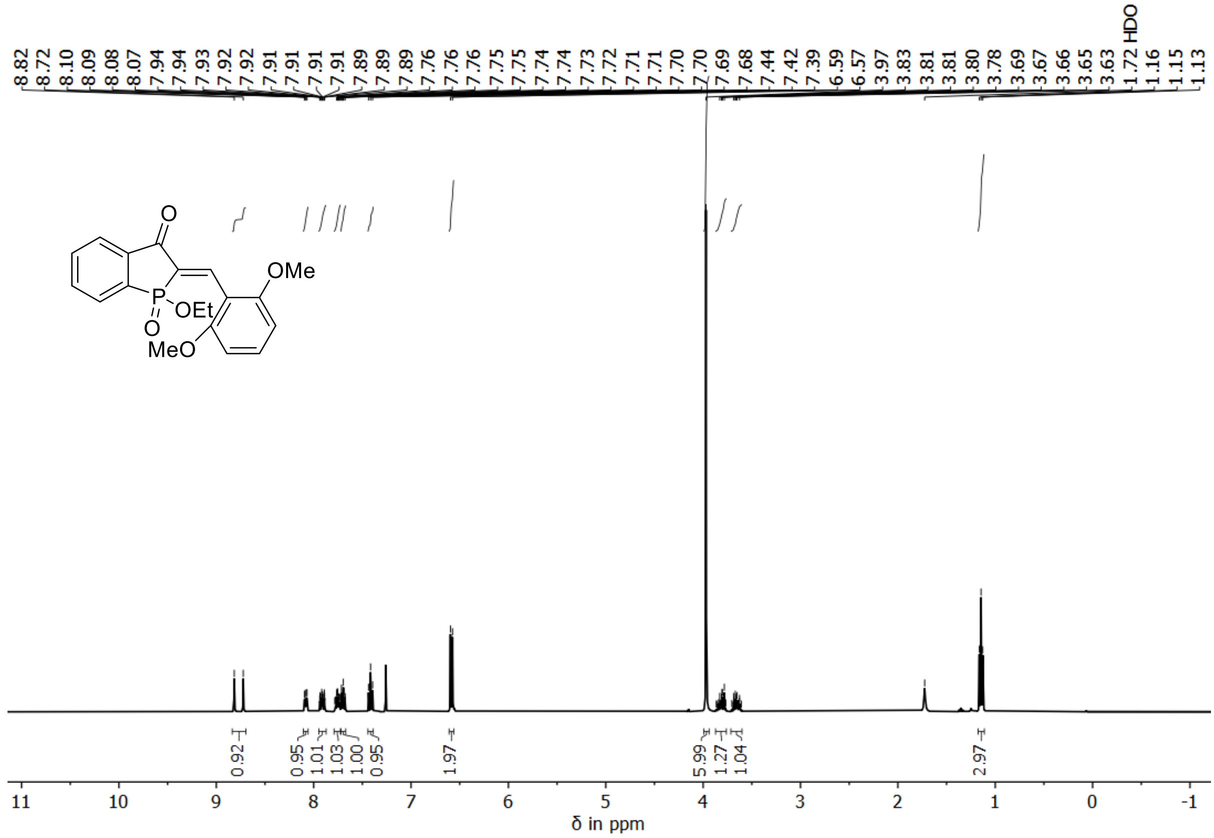

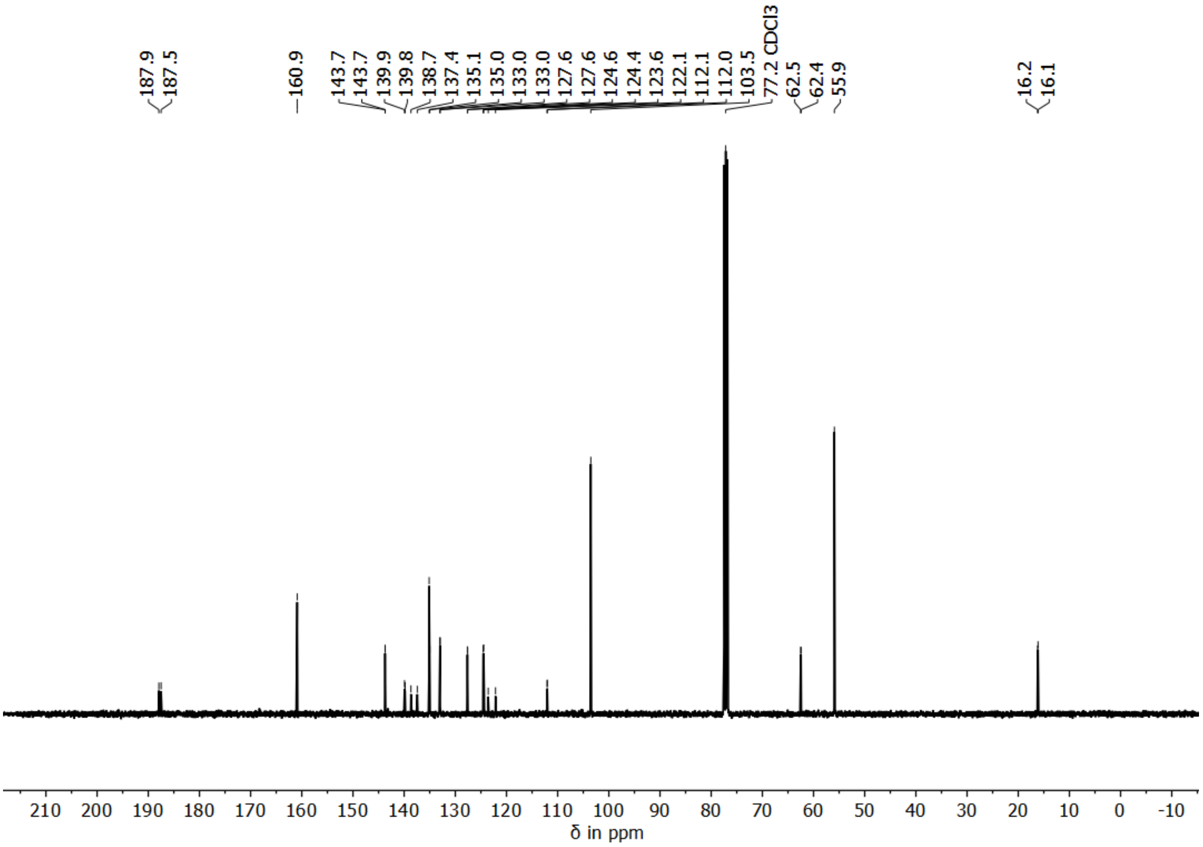

Supplementary Figure 165. NMR spectra of **4** in $CDCl_3$ at 25 °C. (top) 400 MHz ^1H NMR spectrum. (bottom) 101 MHz ^{13}C NMR spectrum.

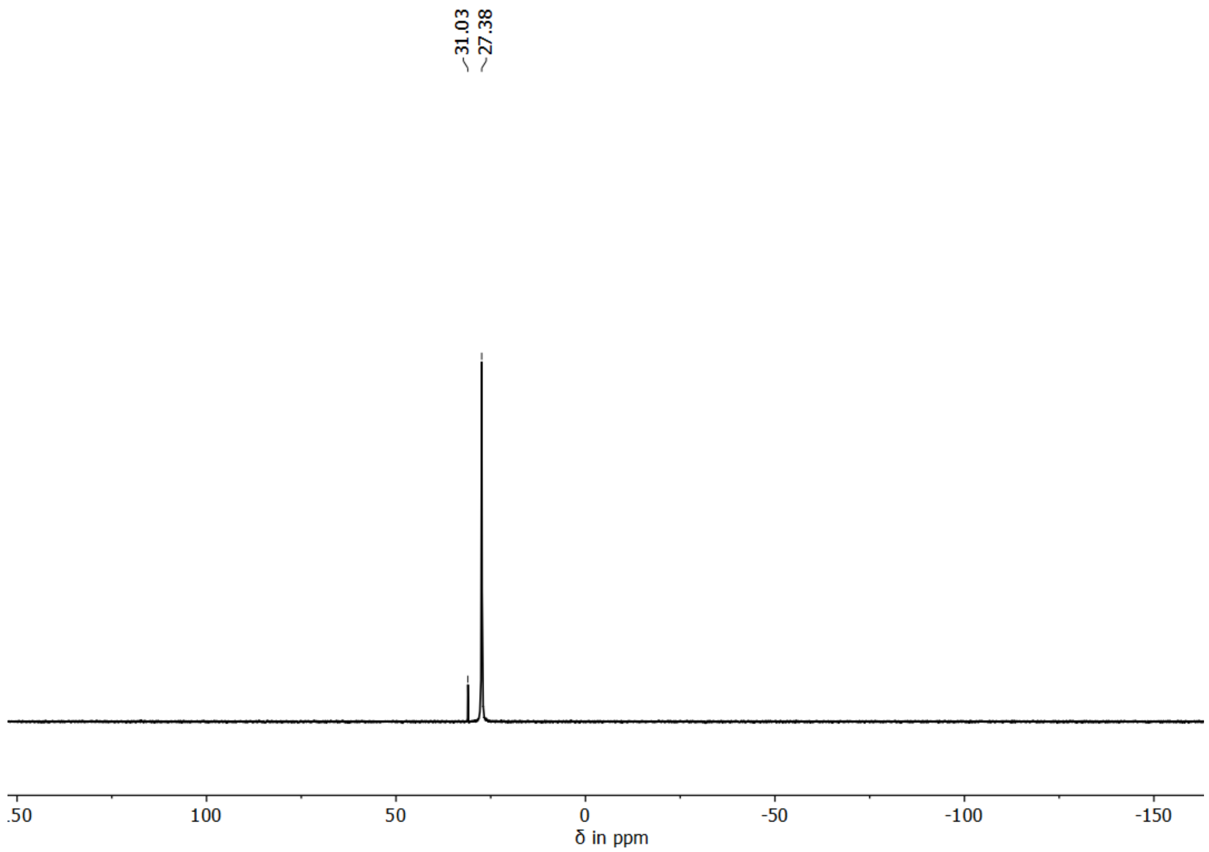

Supplementary Figure 166. 162 MHz ^{31}P NMR spectrum of **4** in $CDCl_3$ at 25 °C.

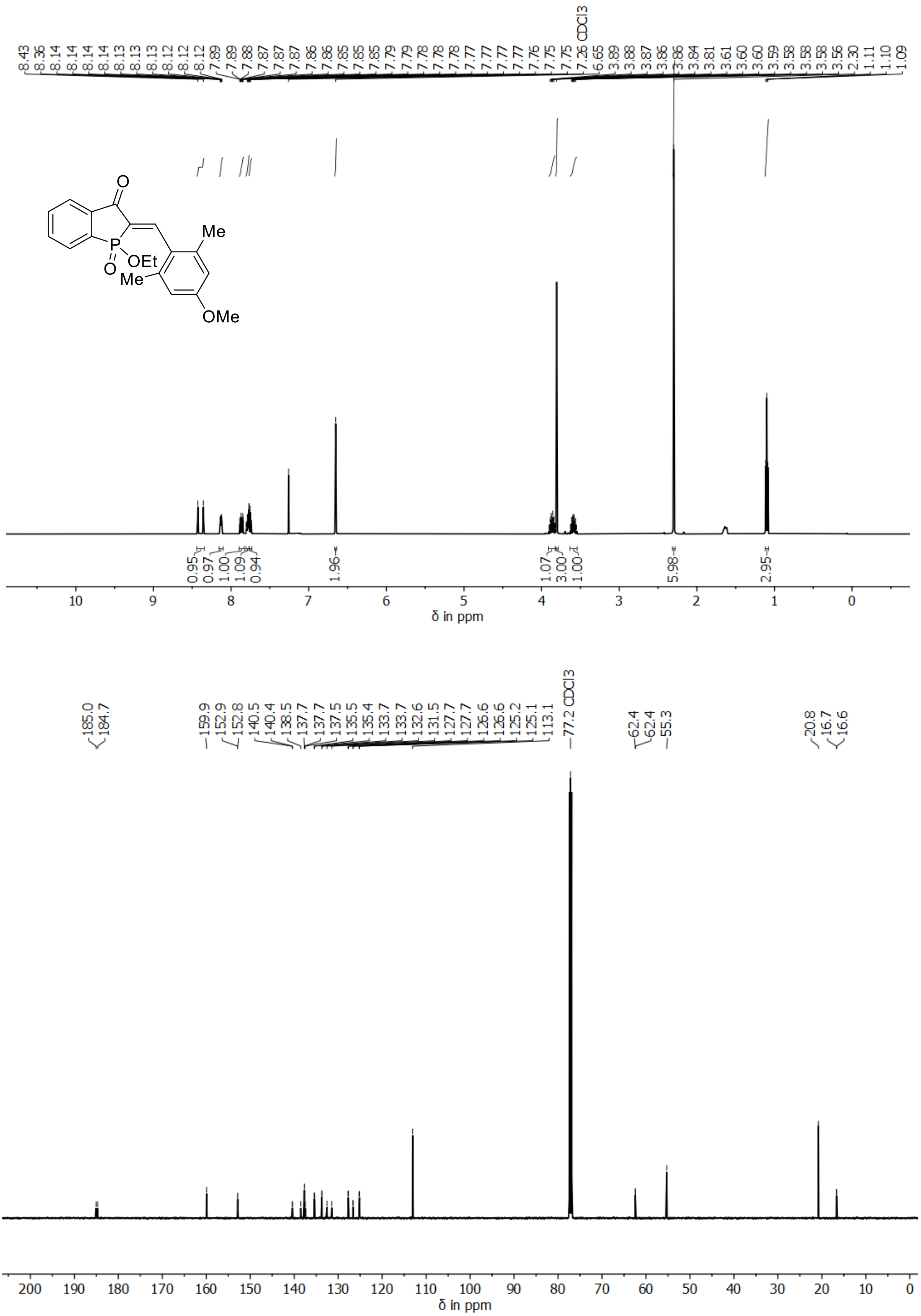

Supplementary Figure 167. NMR spectra of **5** in $CDCl_3$ at 25 °C. (top) 500 MHz ^1H NMR spectrum. (bottom) 126 MHz ^{13}C NMR spectrum.

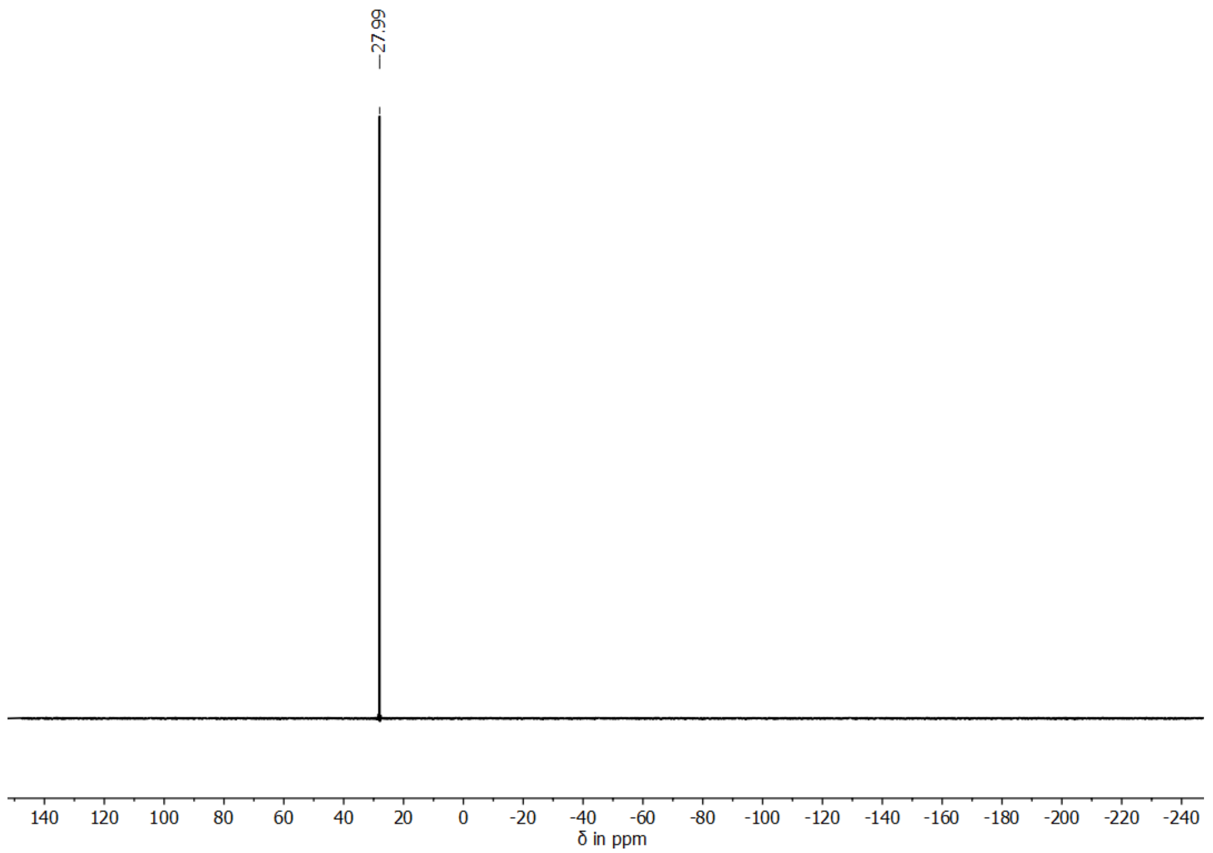

Supplementary Figure 168. 203 MHz ^{31}P NMR spectrum of **5** in $CDCl_3$ at 25 °C.

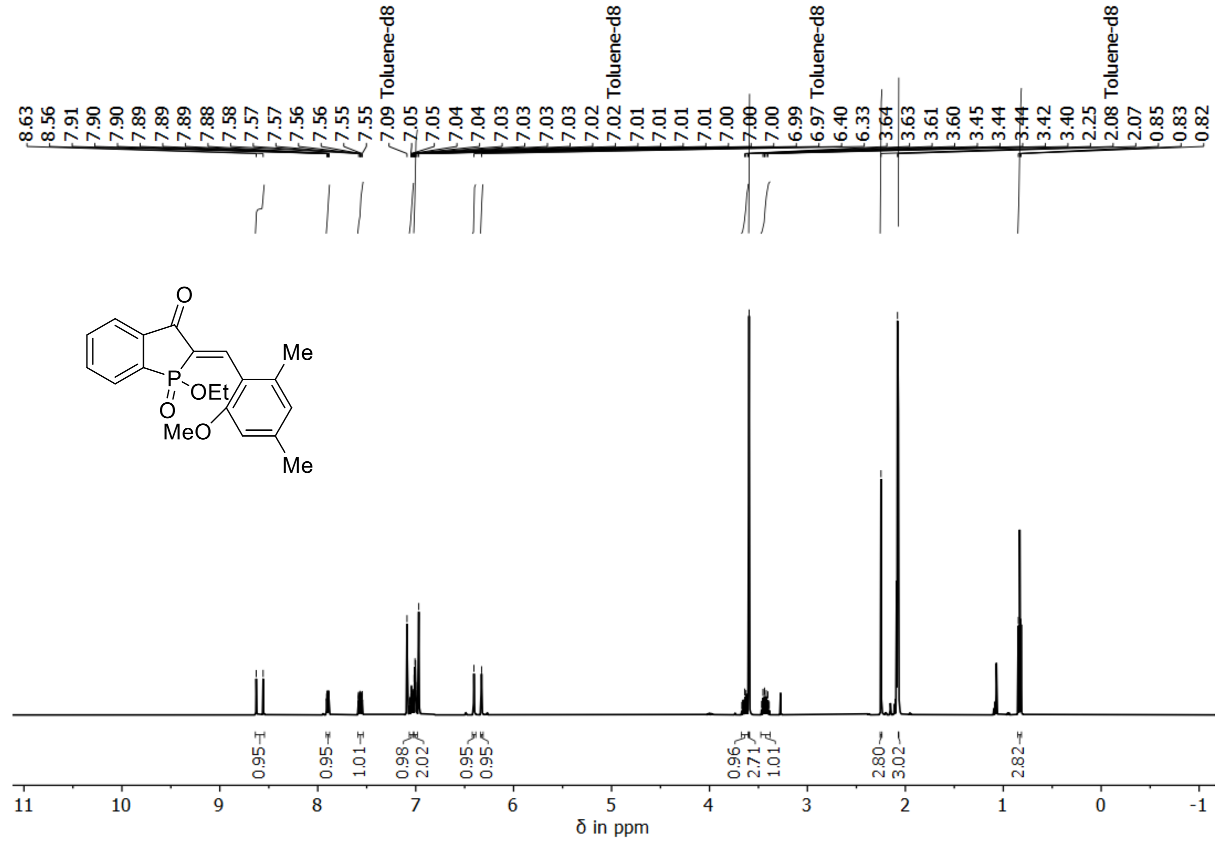

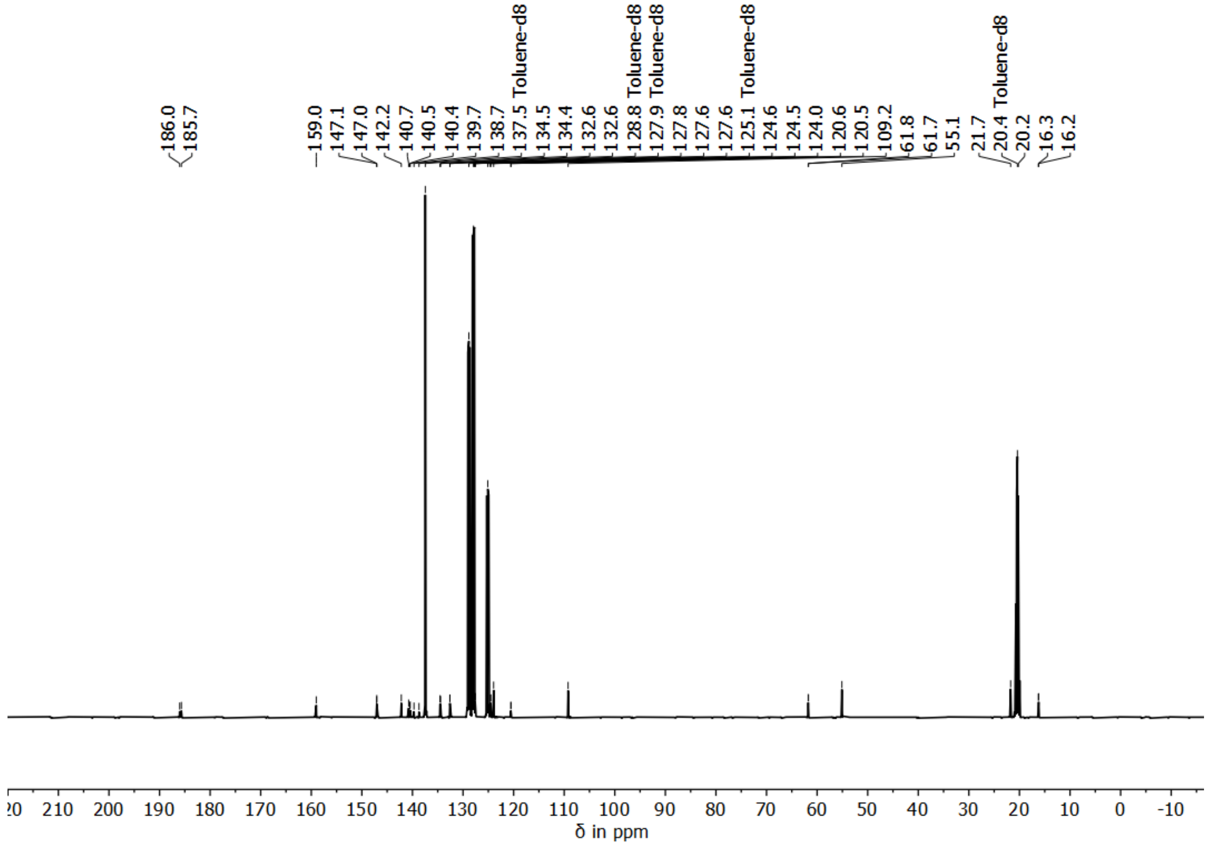

Supplementary Figure 169. NMR spectra of **6** in toluene-d_8 at 25 °C. (top) 500 MHz ^{1}H NMR spectrum. (bottom) 126 MHz ^{13}C NMR spectrum.

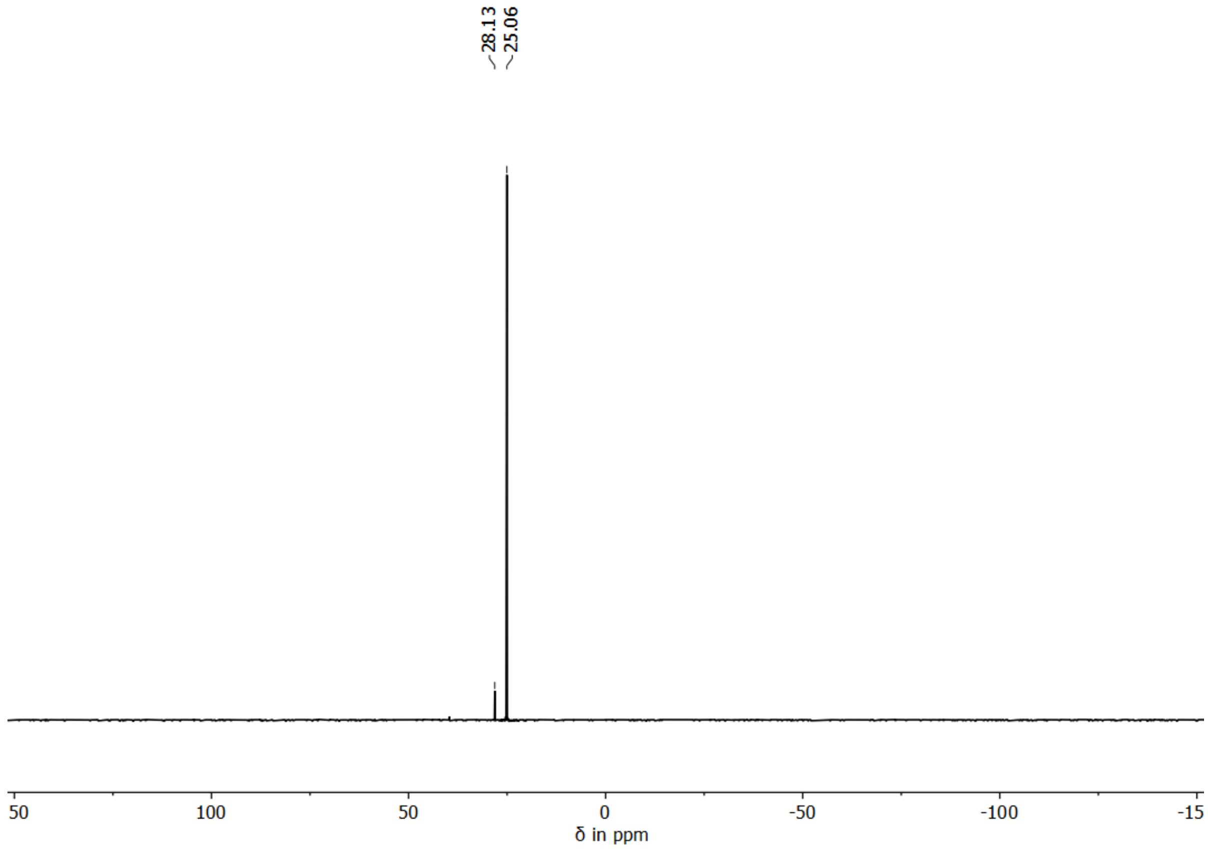

Supplementary Figure 170. 203 MHz ^{31}P NMR spectrum of **6** in toluene-d_8 at 25 °C.

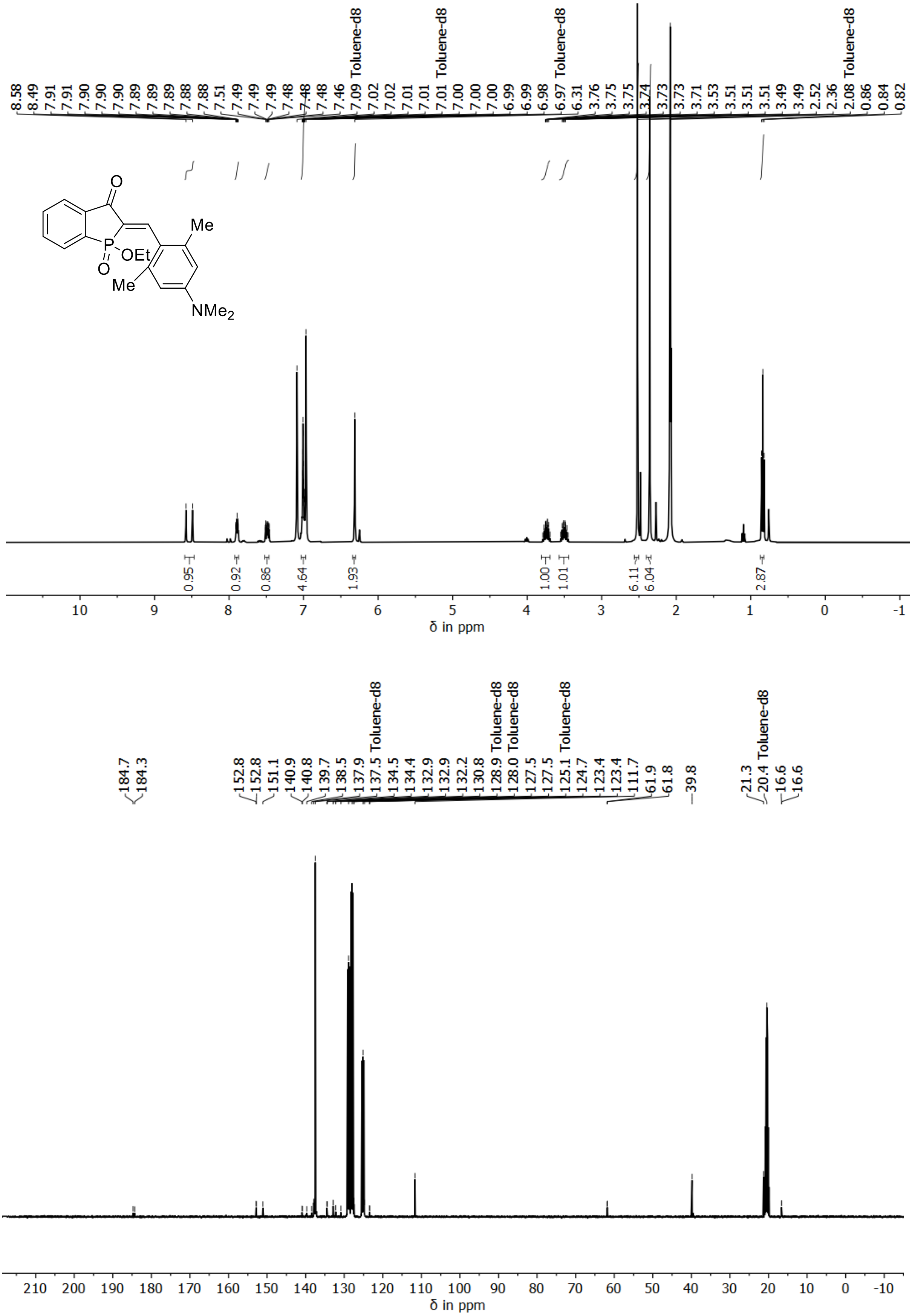

Supplementary Figure 171. NMR spectra of **7** in toluene-d_8 at 25 °C. (top) 400 MHz ^{1}H NMR spectrum. (bottom) 101 MHz ^{13}C NMR spectrum.

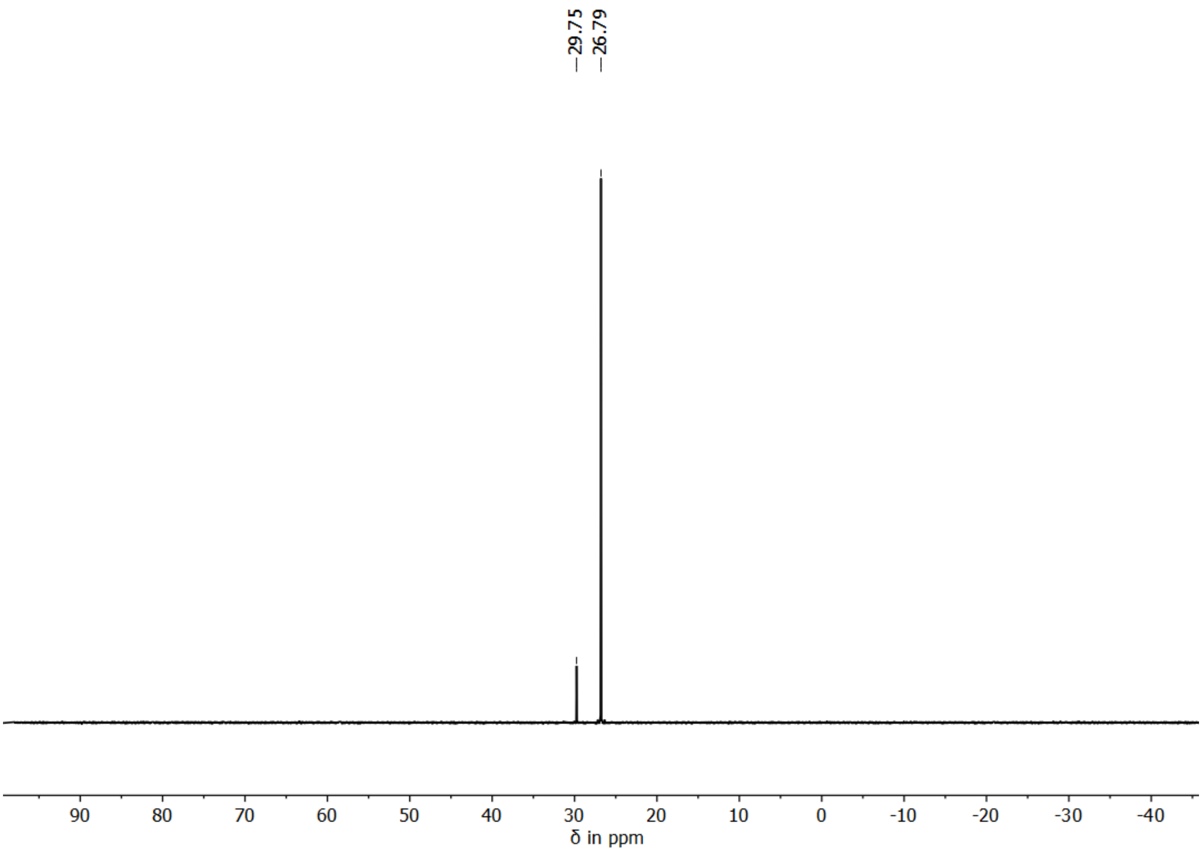

Supplementary Figure 172. 162 MHz ^{31}P NMR spectrum of **7** in toluene-d_8 at 25 °C.

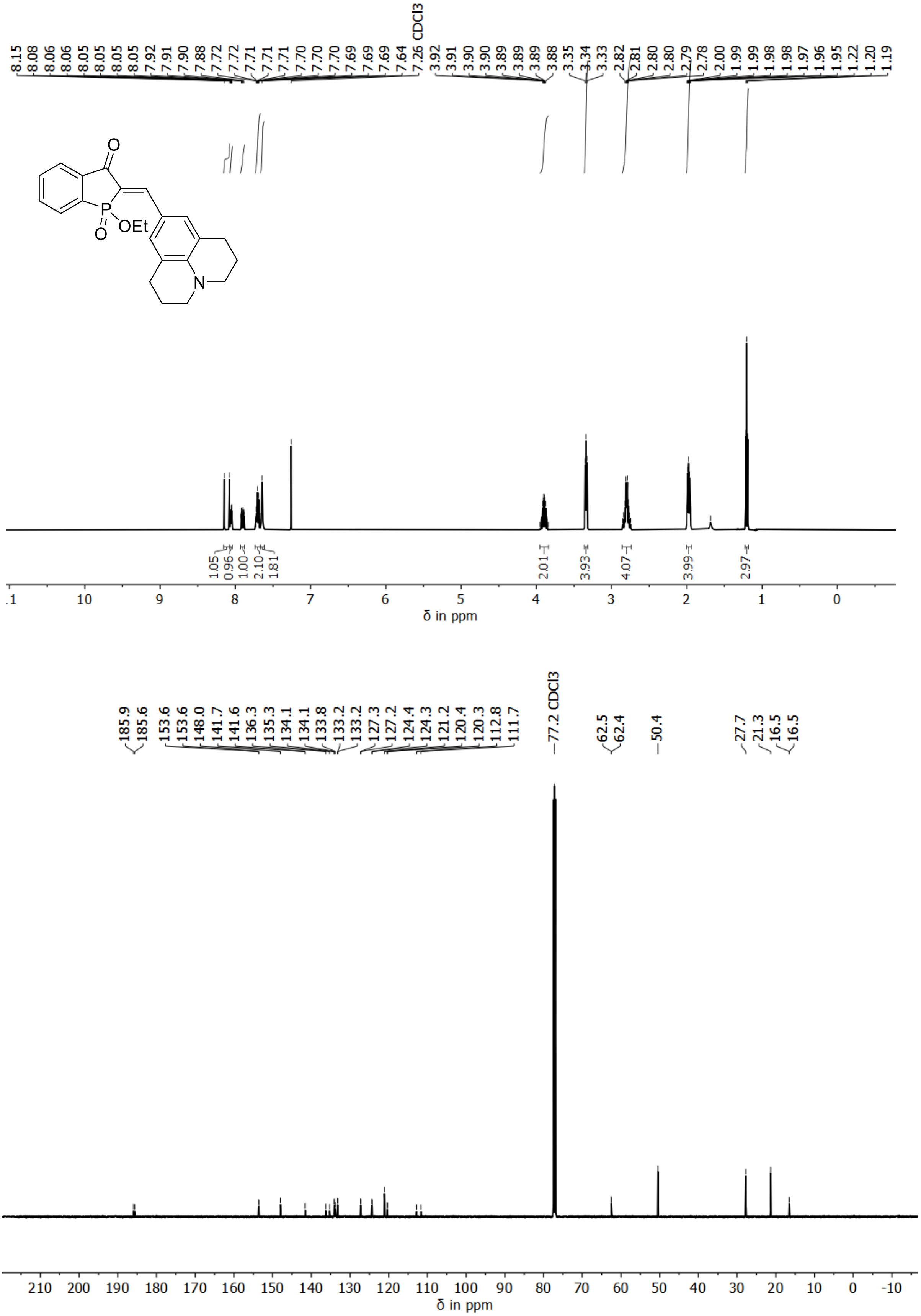

Supplementary Figure 173. NMR spectra of **8** in $CDCl_3$ at 25 °C. (top) 500 MHz ^1H NMR spectrum. (bottom) 126 MHz ^{13}C NMR spectrum.

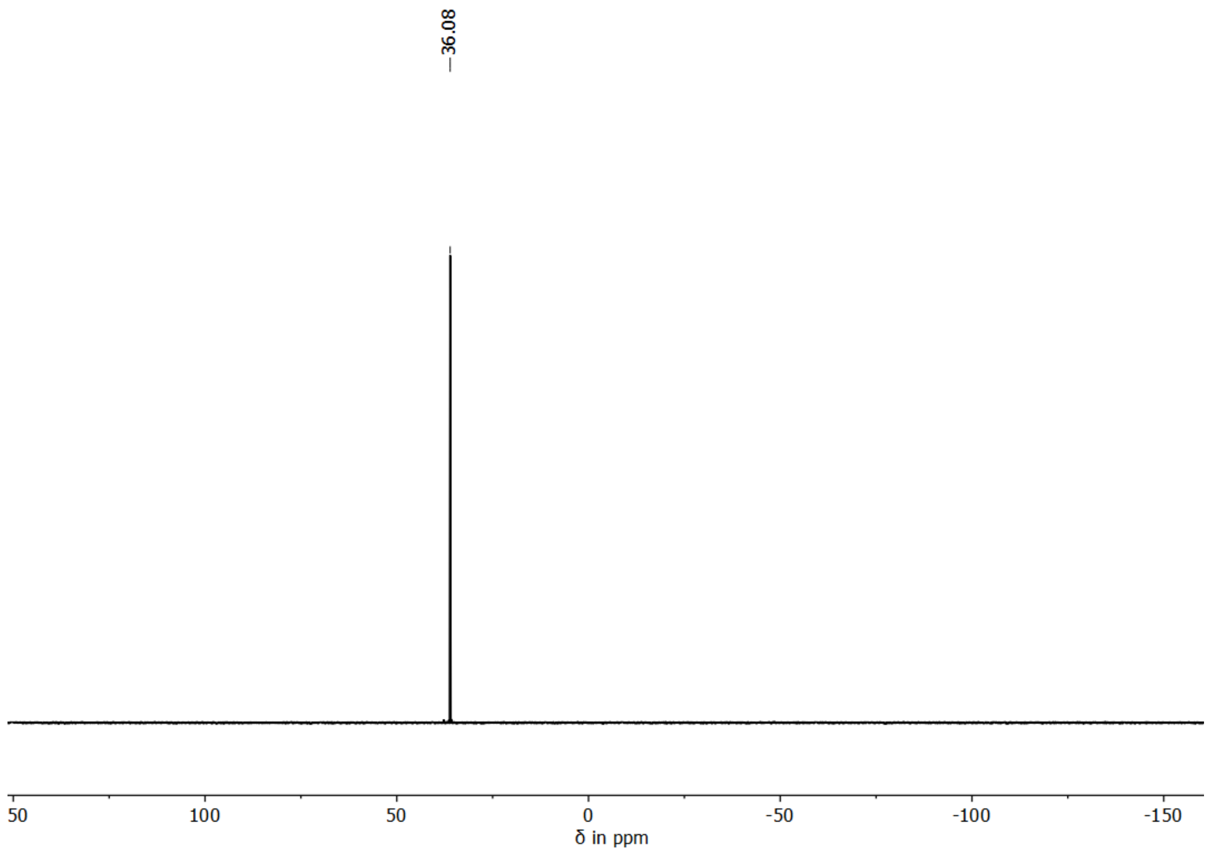

Supplementary Figure 174. 203 MHz ^{31}P NMR spectrum of **8** in $CDCl_3$ at 25 °C.

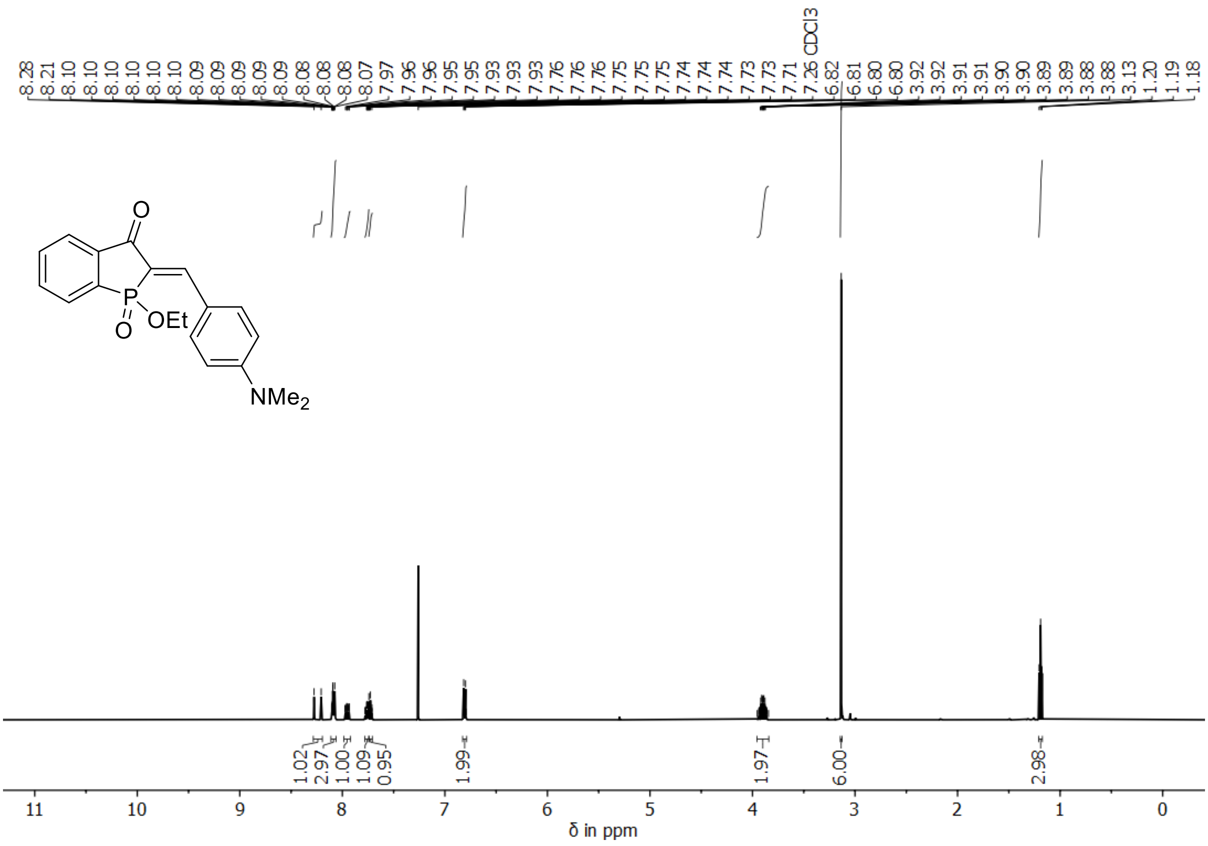

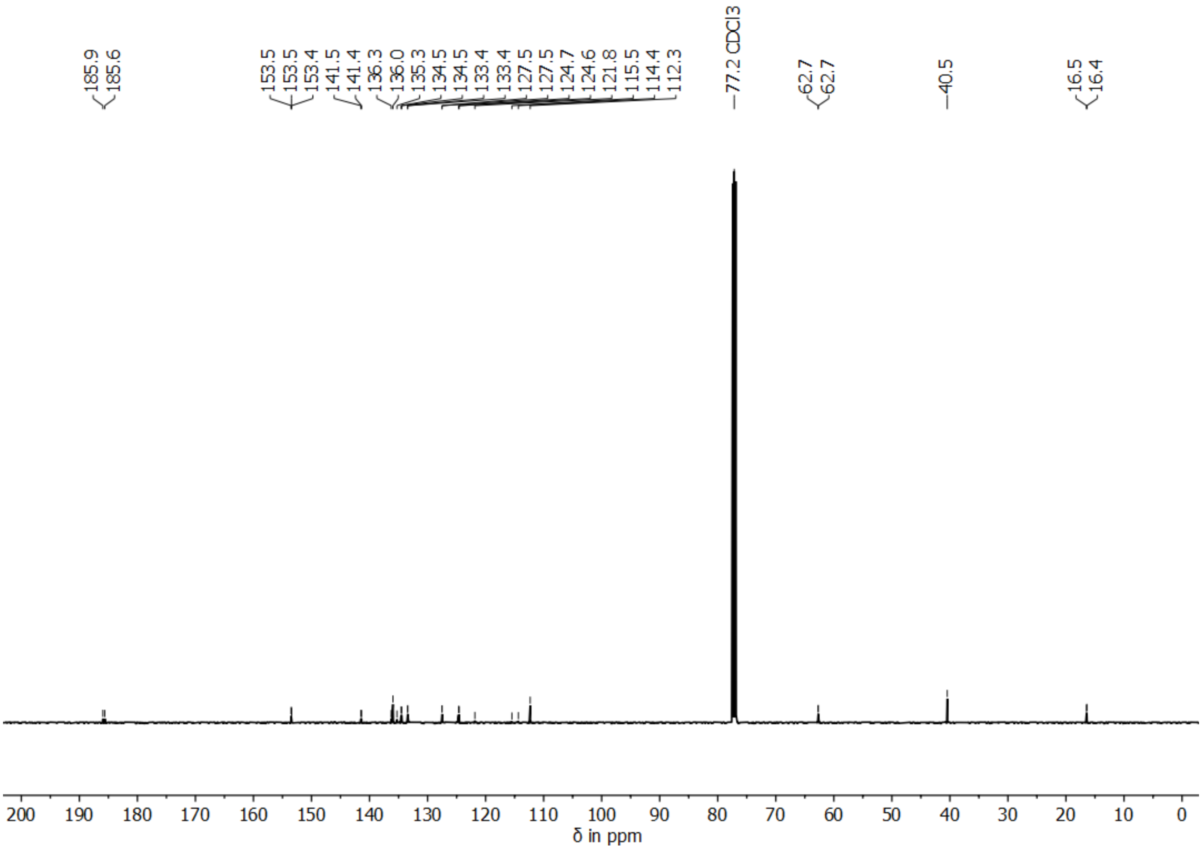

Supplementary Figure 175. NMR spectra of **9** in $CDCl_3$ at 25 °C. (top) 500 MHz ^1H NMR spectrum. (bottom) 126 MHz ^{13}C NMR spectrum.

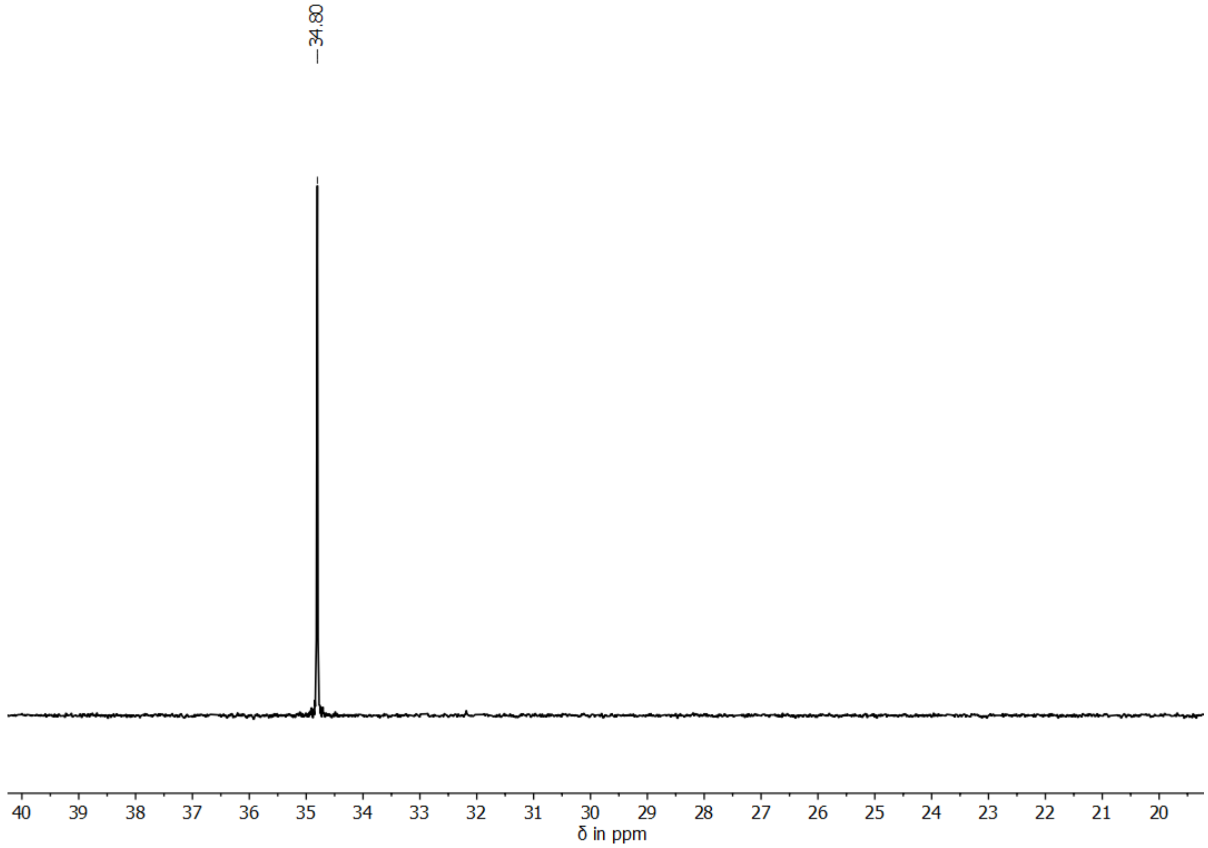

Supplementary Figure 176. 203 MHz ^{31}P NMR spectrum of **9** in $CDCl_3$ at 25 °C.

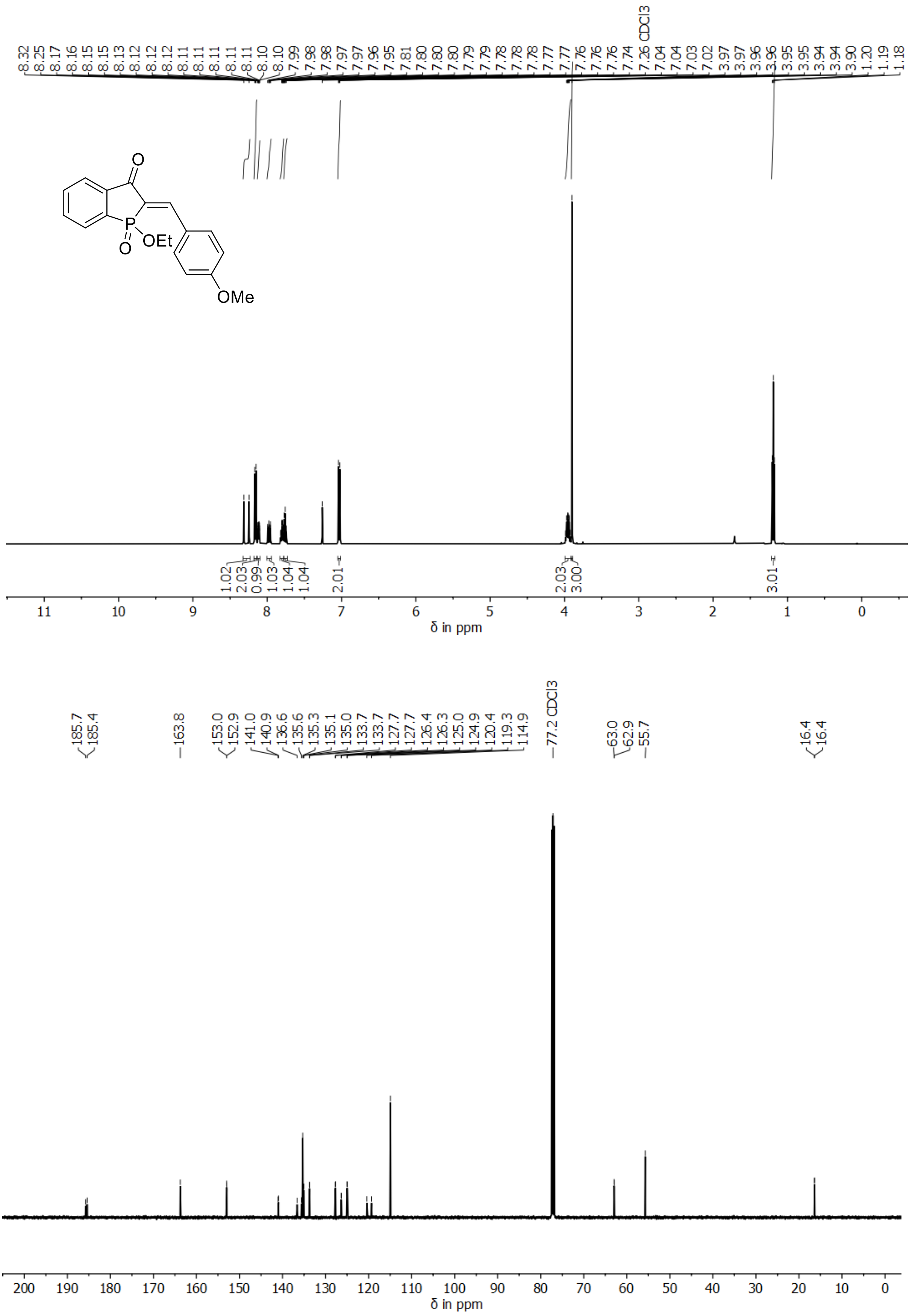

Supplementary Figure 177. NMR spectra of **10** in $CDCl_3$ at 25 °C. (top) 500 MHz ^1H NMR spectrum. (bottom) 126 MHz ^{13}C NMR spectrum.

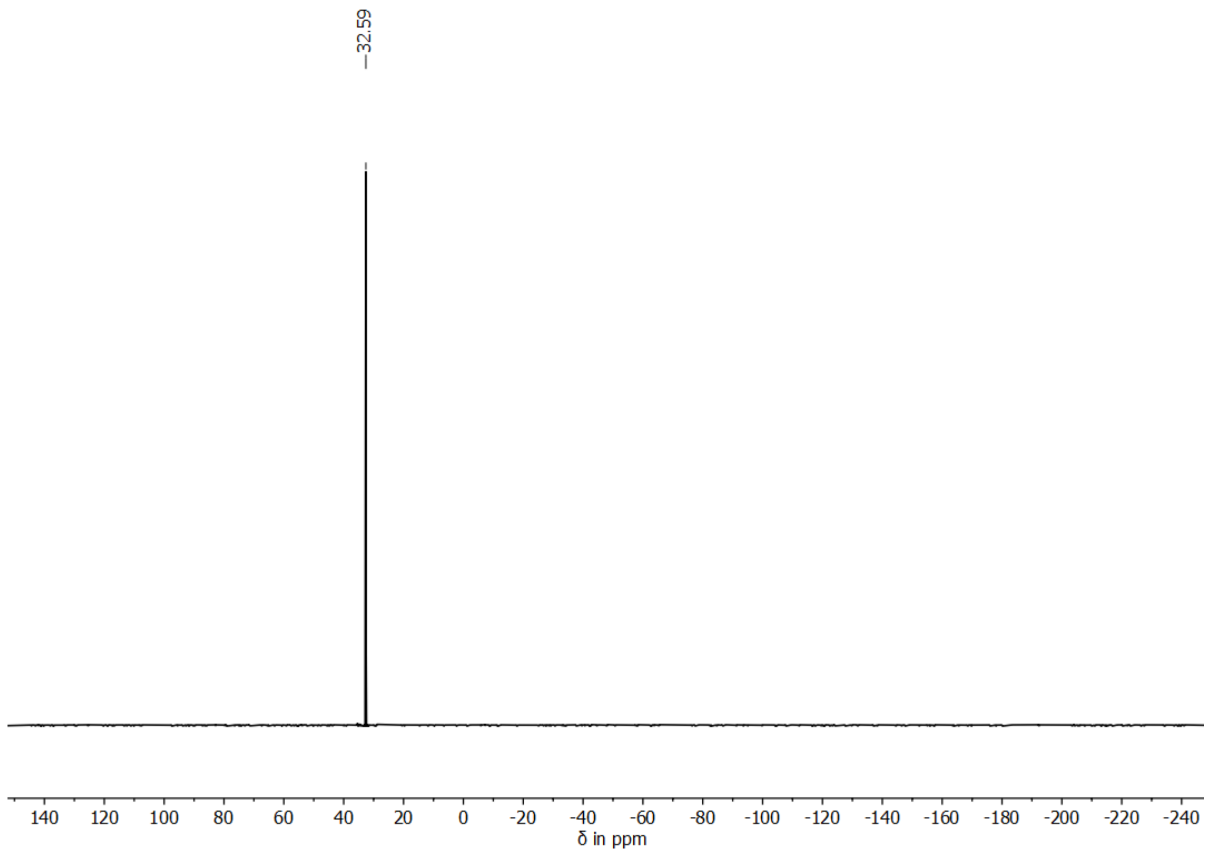

Supplementary Figure 178. 203 MHz ^{31}P NMR spectrum of **10** in $CDCl_3$ at 25 °C.

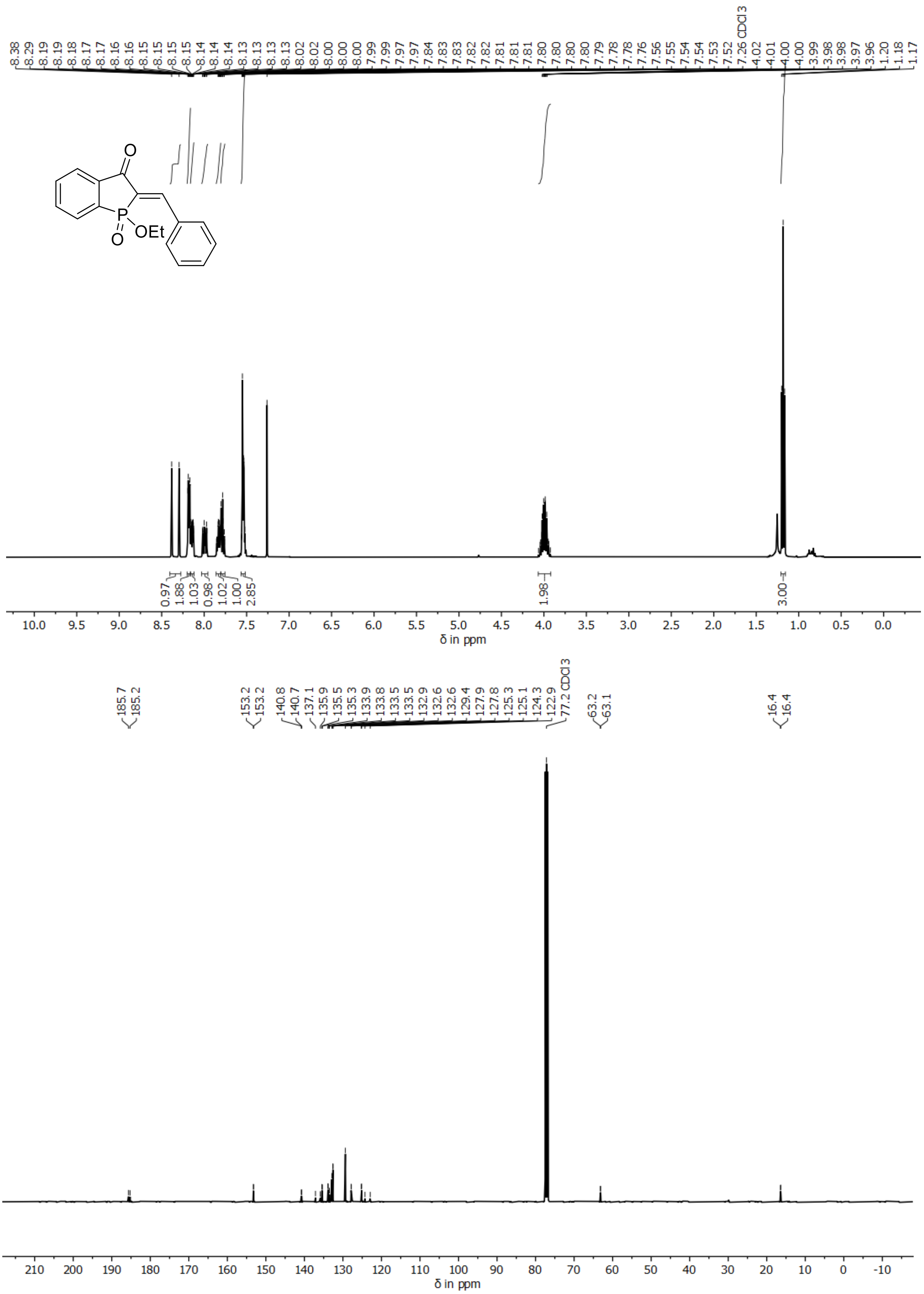

Supplementary Figure 179. NMR spectra of **11** in $CDCl_3$ at 25 °C. (top) 400 MHz ^1H NMR spectrum. (bottom) 101 MHz ^{13}C NMR spectrum.

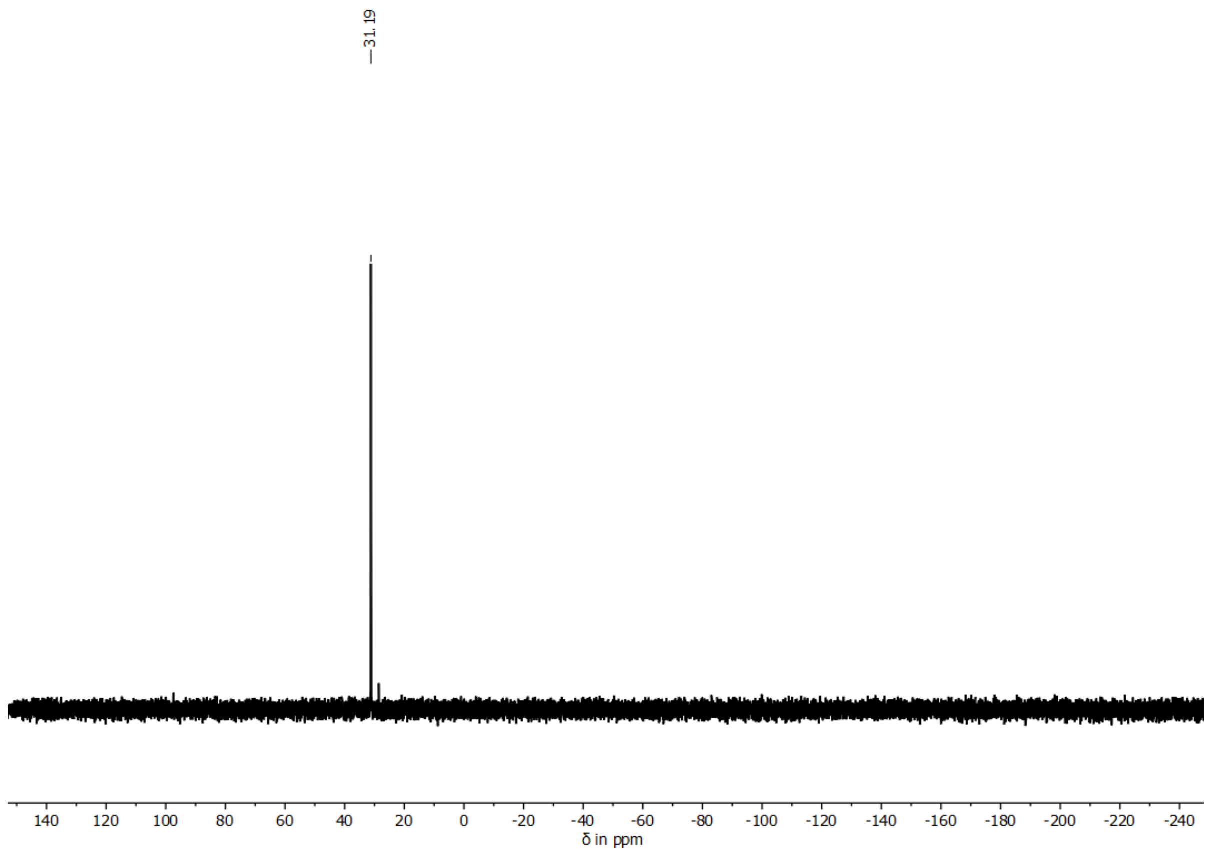

Supplementary Figure 180. 162 MHz ^{31}P NMR spectrum of **11** in $CDCl_3$ at 25 °C.

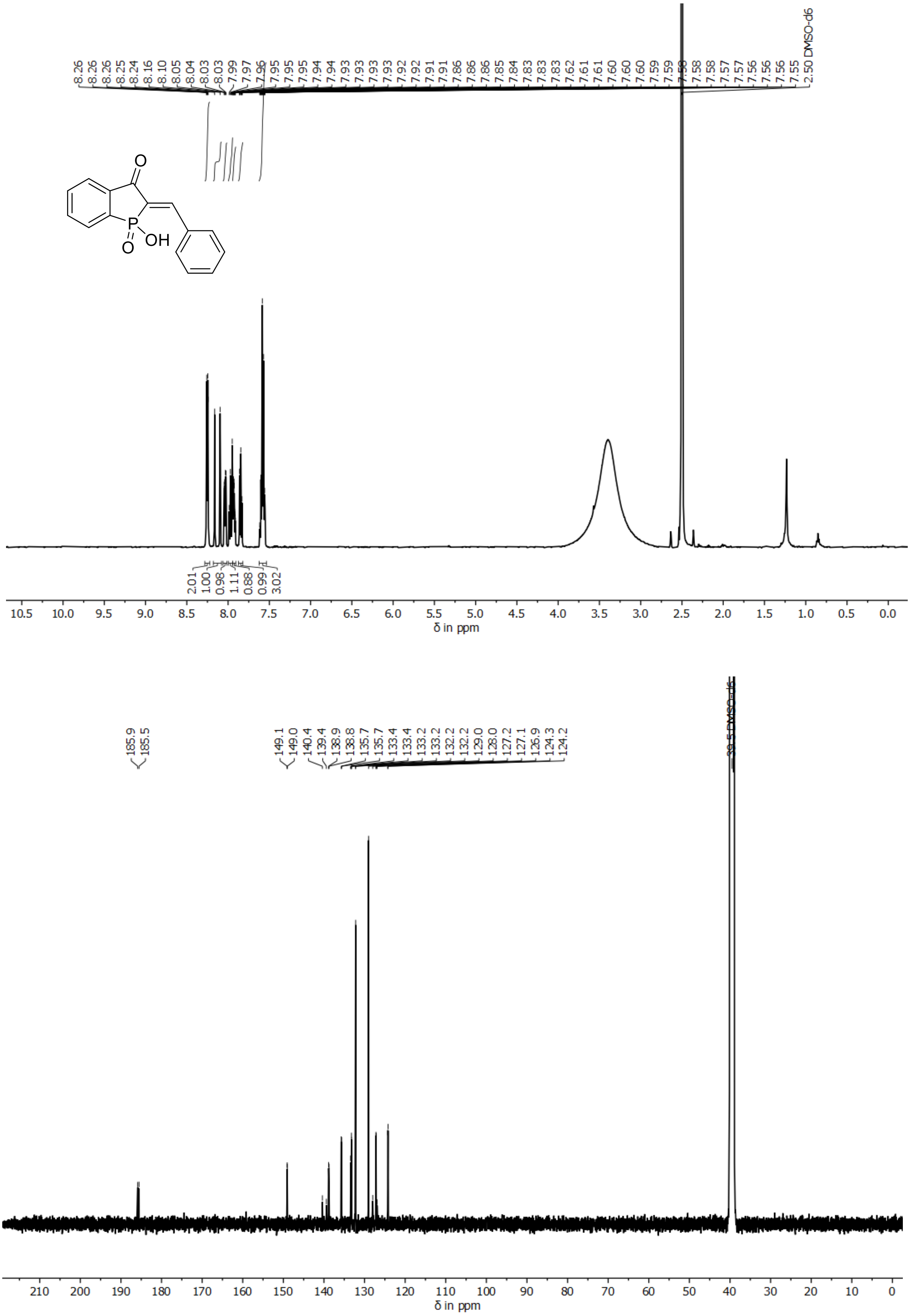

Supplementary Figure 181. NMR spectra of **11-**OH in DMSO-d_6 at 25 °C. (top) 500 MHz ^{1}H NMR spectrum. (bottom) 126 MHz ^{13}C NMR spectrum.

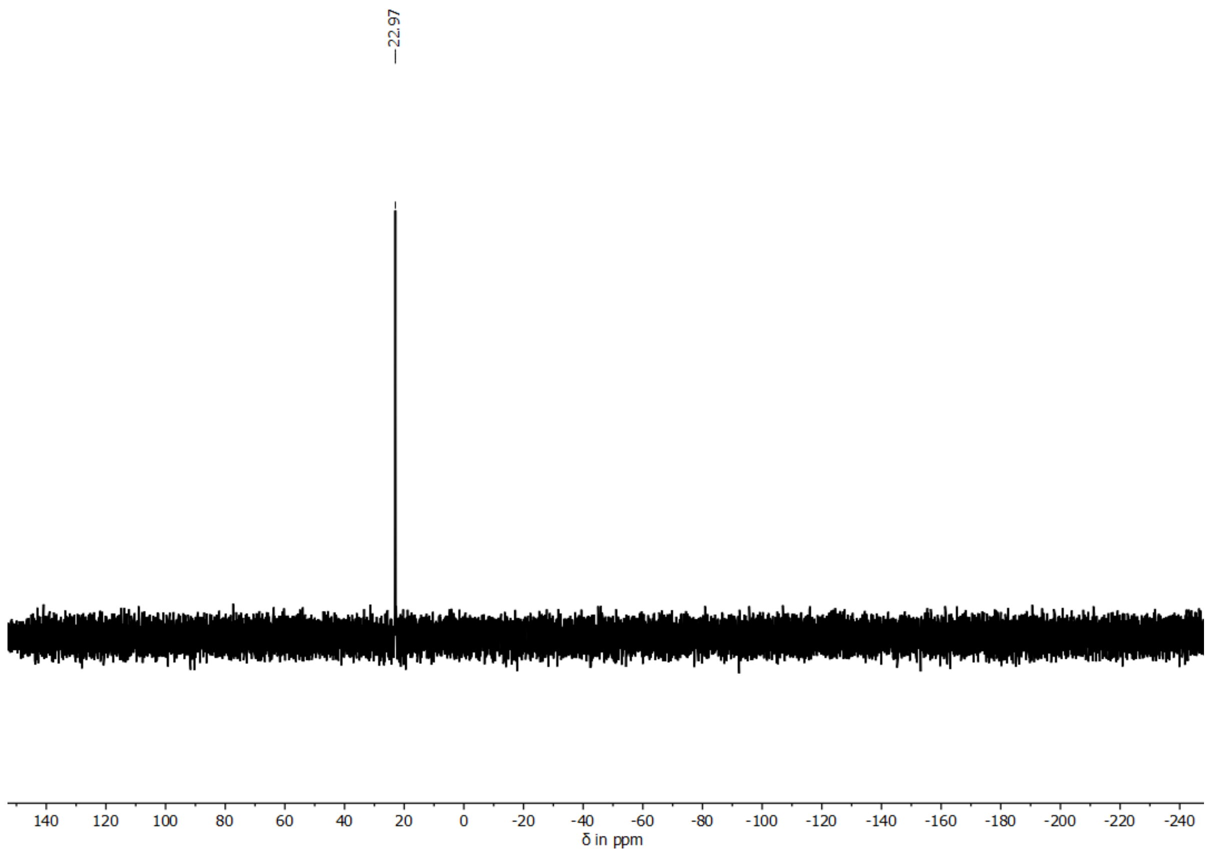

Supplementary Figure 182. 162 MHz ^{31}P NMR spectrum of **11**-OH in DMSO-d_6 at 25 °C.

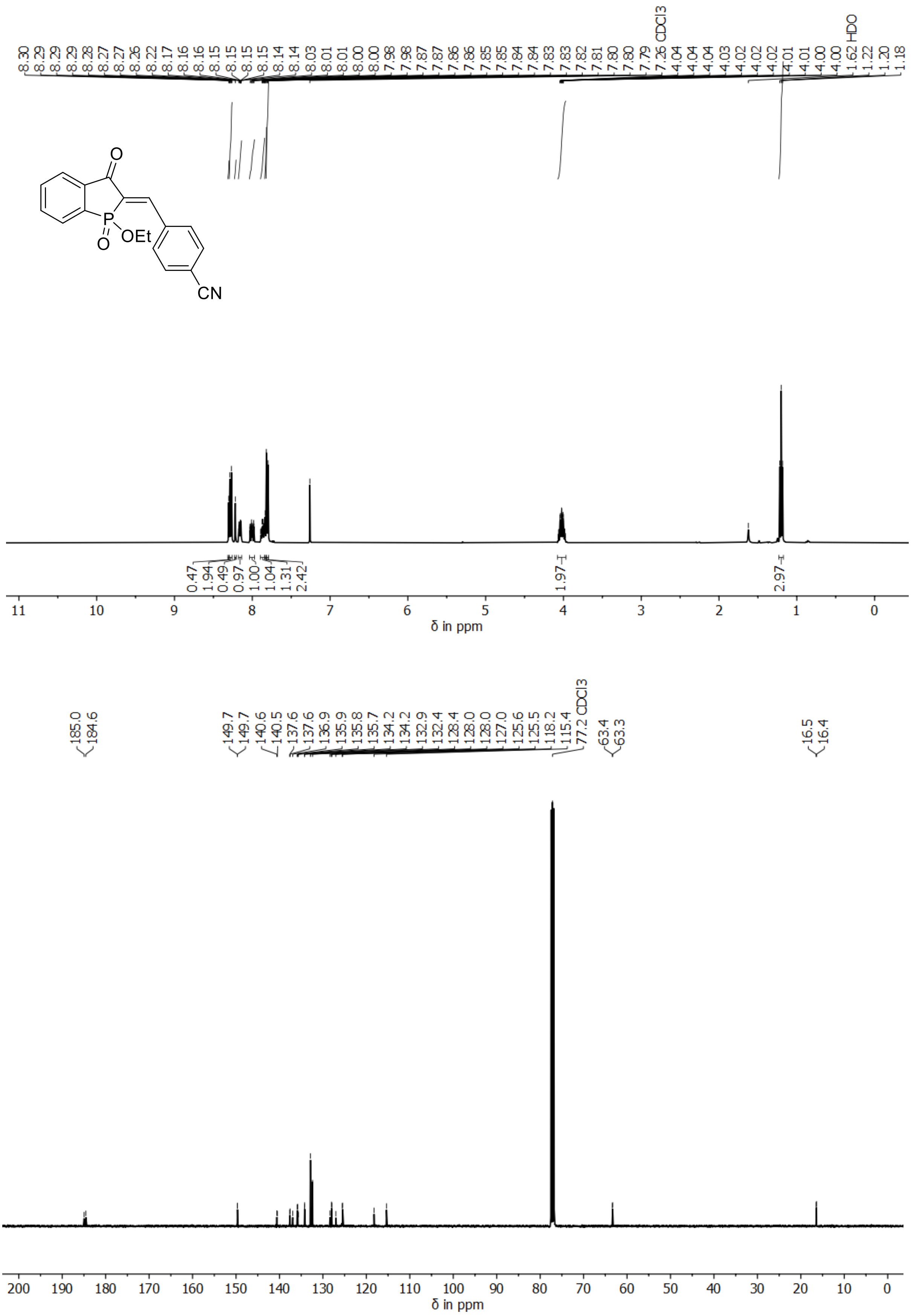

Supplementary Figure 183. NMR spectra of **12** in $CDCl_3$ at 25 °C. (top) 500 MHz ^{1}H NMR spectrum. (bottom) 126 MHz ^{13}C NMR spectrum.

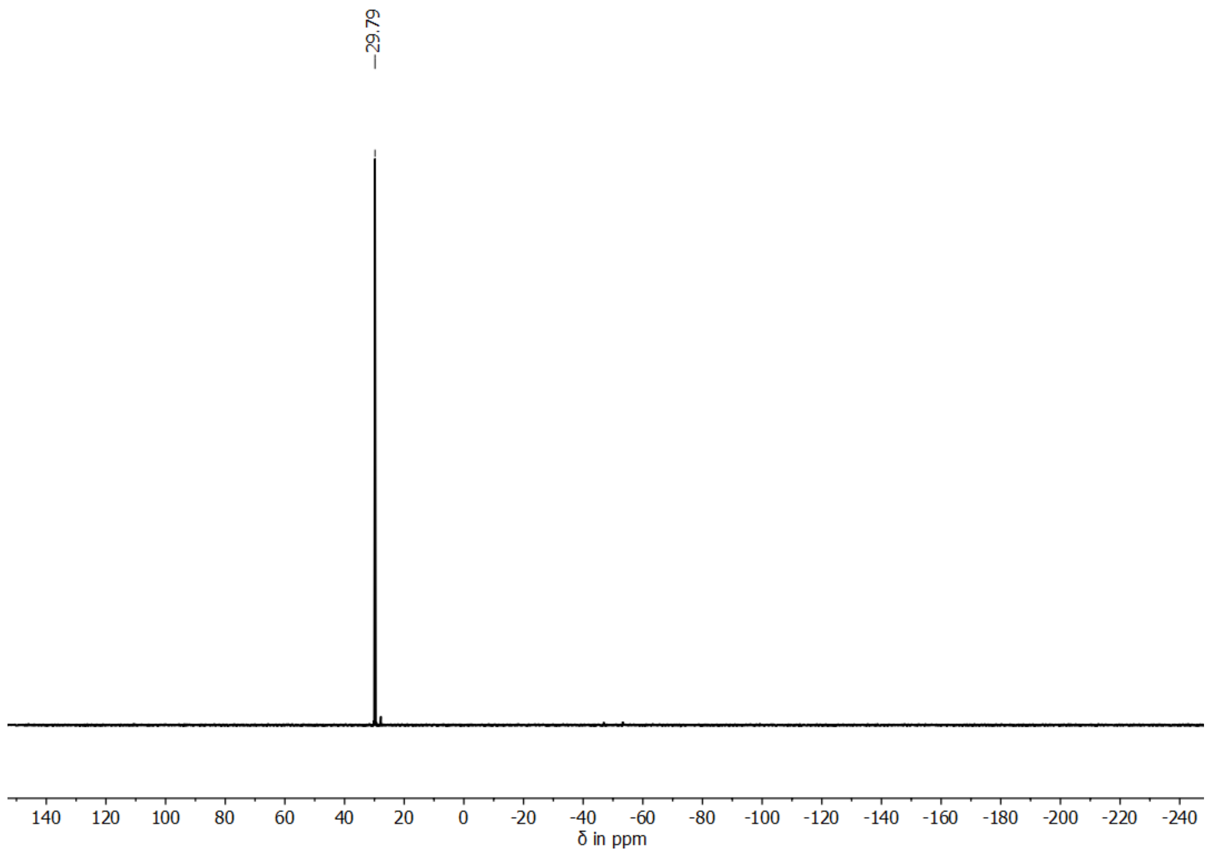

Supplementary Figure 184. 203 MHz ^{31}P NMR spectrum of **12** in $CDCl_3$ at 25 °C.

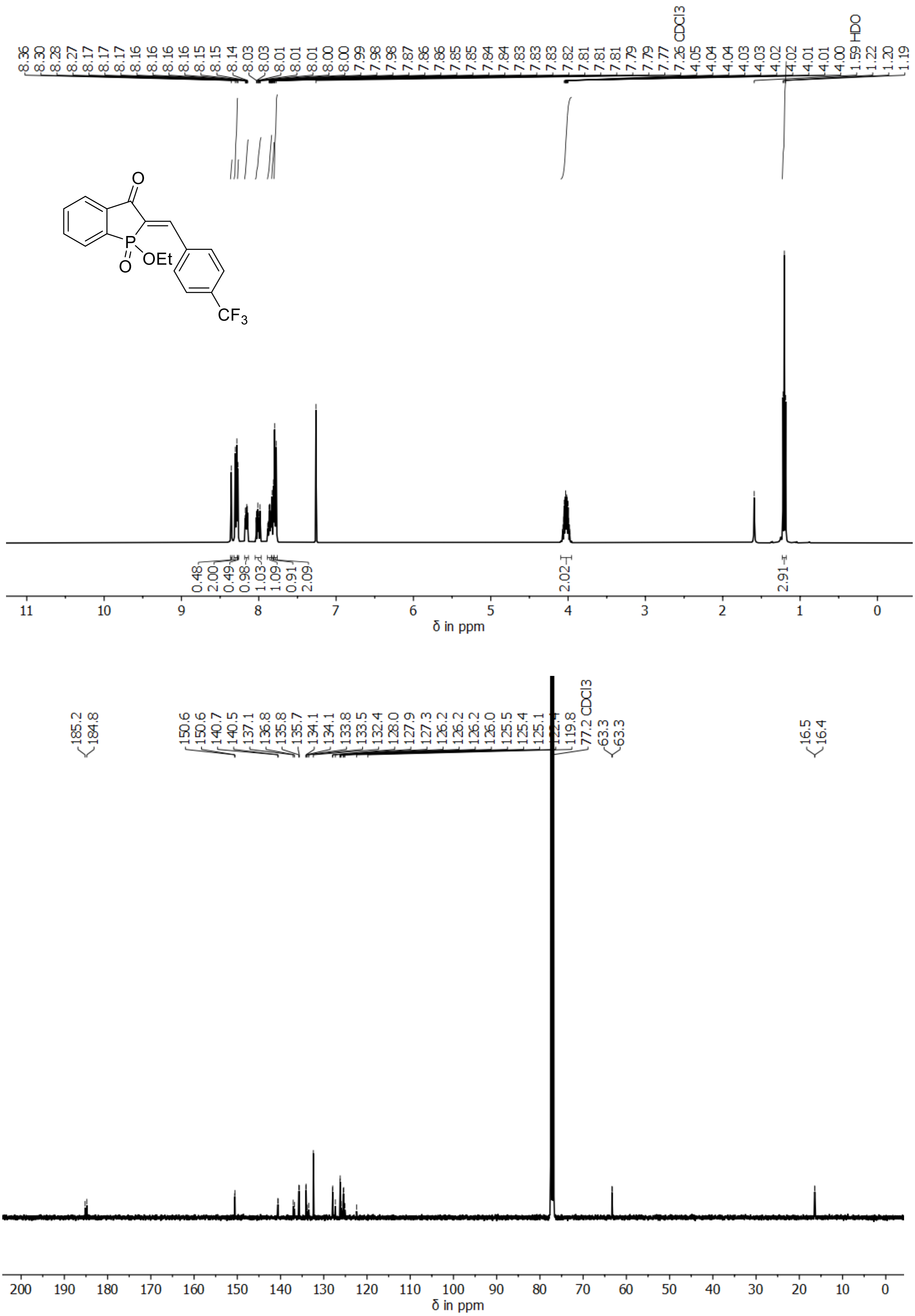

Supplementary Figure 185. NMR spectra of **13** in $CDCl_3$ at 25 °C. (top) 400 MHz ^{1}H NMR spectrum. (bottom) 101 MHz ^{13}C NMR spectrum.

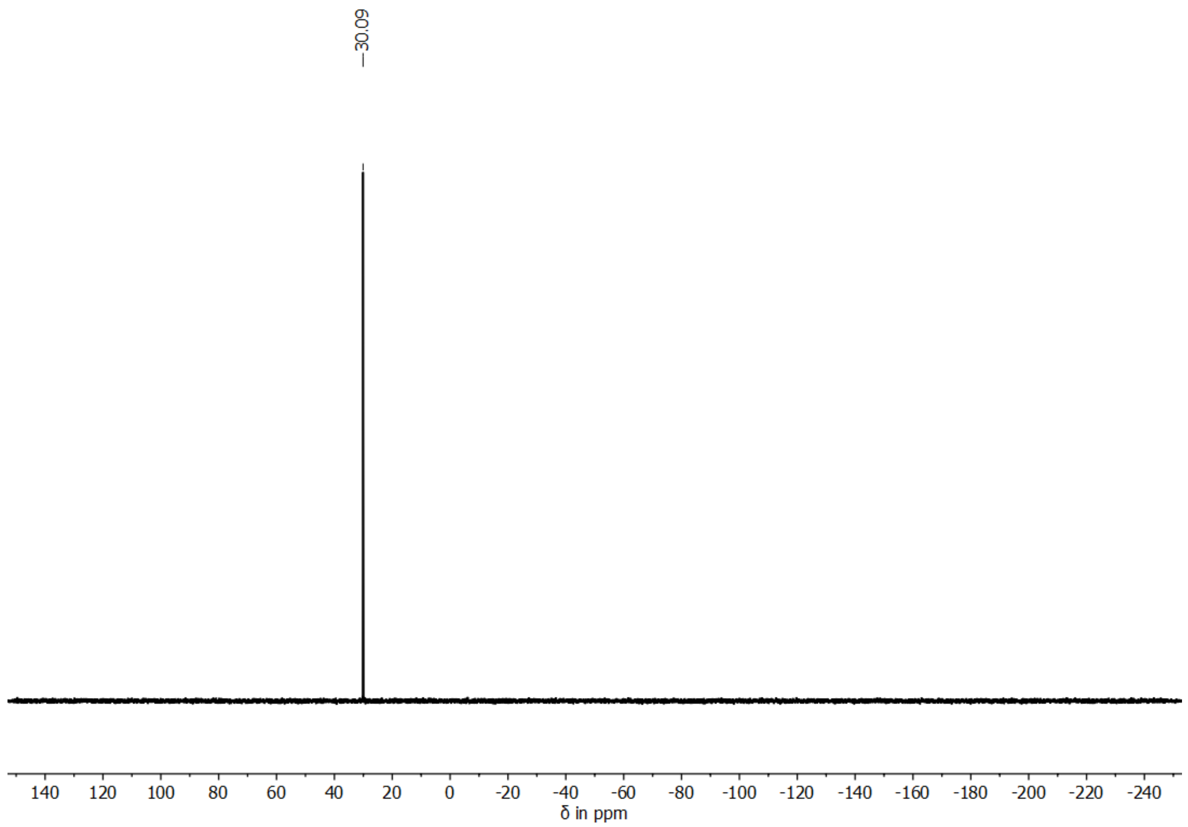

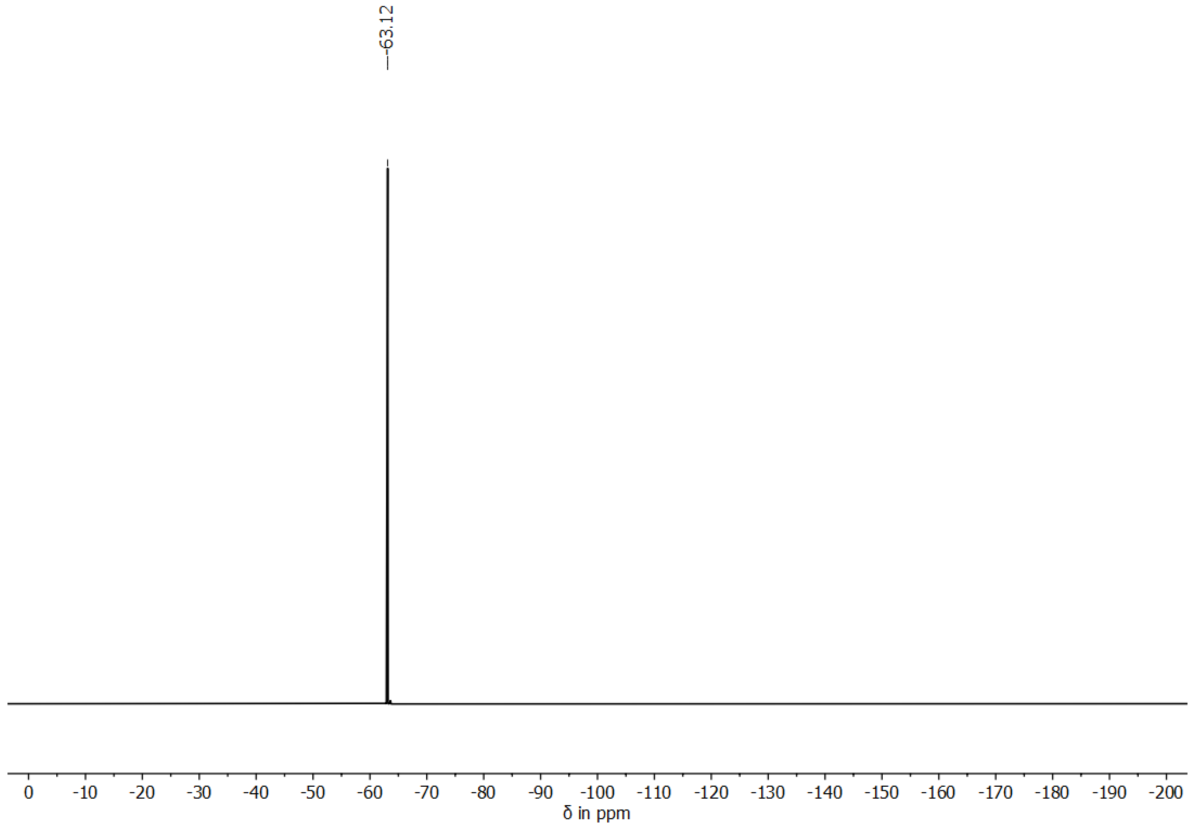

Supplementary Figure 186. NMR spectra of **13** in $CDCl_3$ at 25 °C. (top) 162 MHz ^{31}P NMR spectrum. (bottom) 377 MHz ^{19}F NMR spectrum.

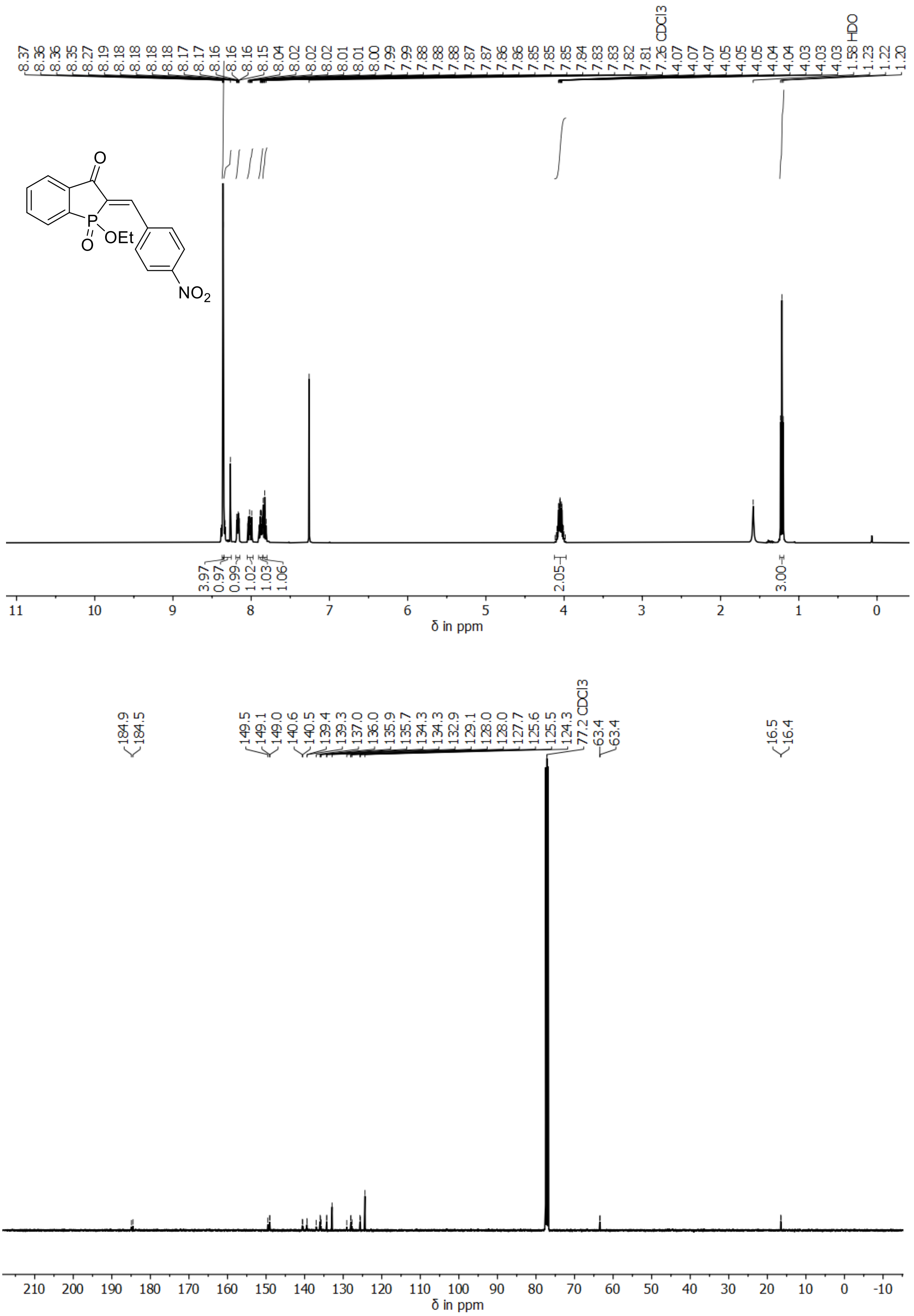

Supplementary Figure 187. NMR spectra of **14** in $CDCl_3$ at 25 °C. (top) 400 MHz ^1H NMR spectrum. (bottom) 101 MHz ^{13}C NMR spectrum.

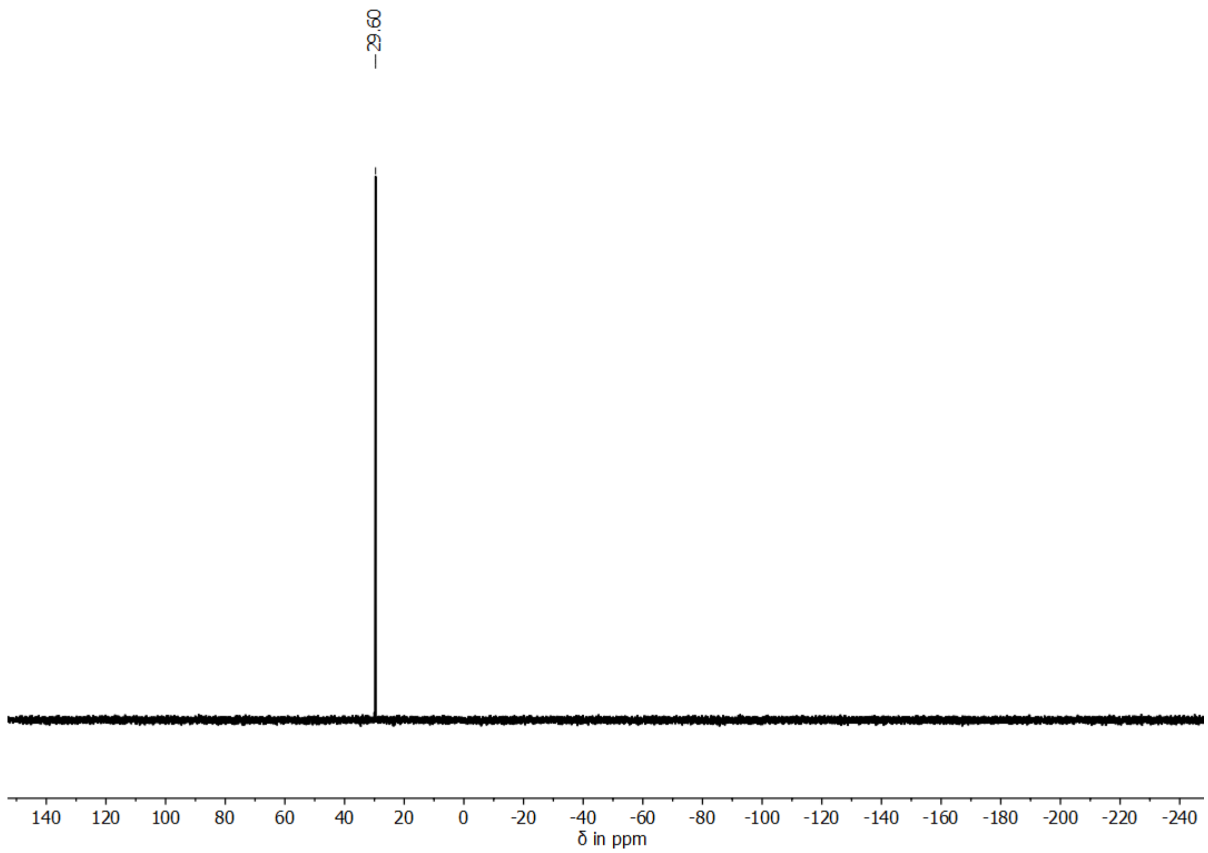

Supplementary Figure 188. 162 MHz ^{31}P NMR spectrum of **14** in $CDCl_3$ at 25 °C.

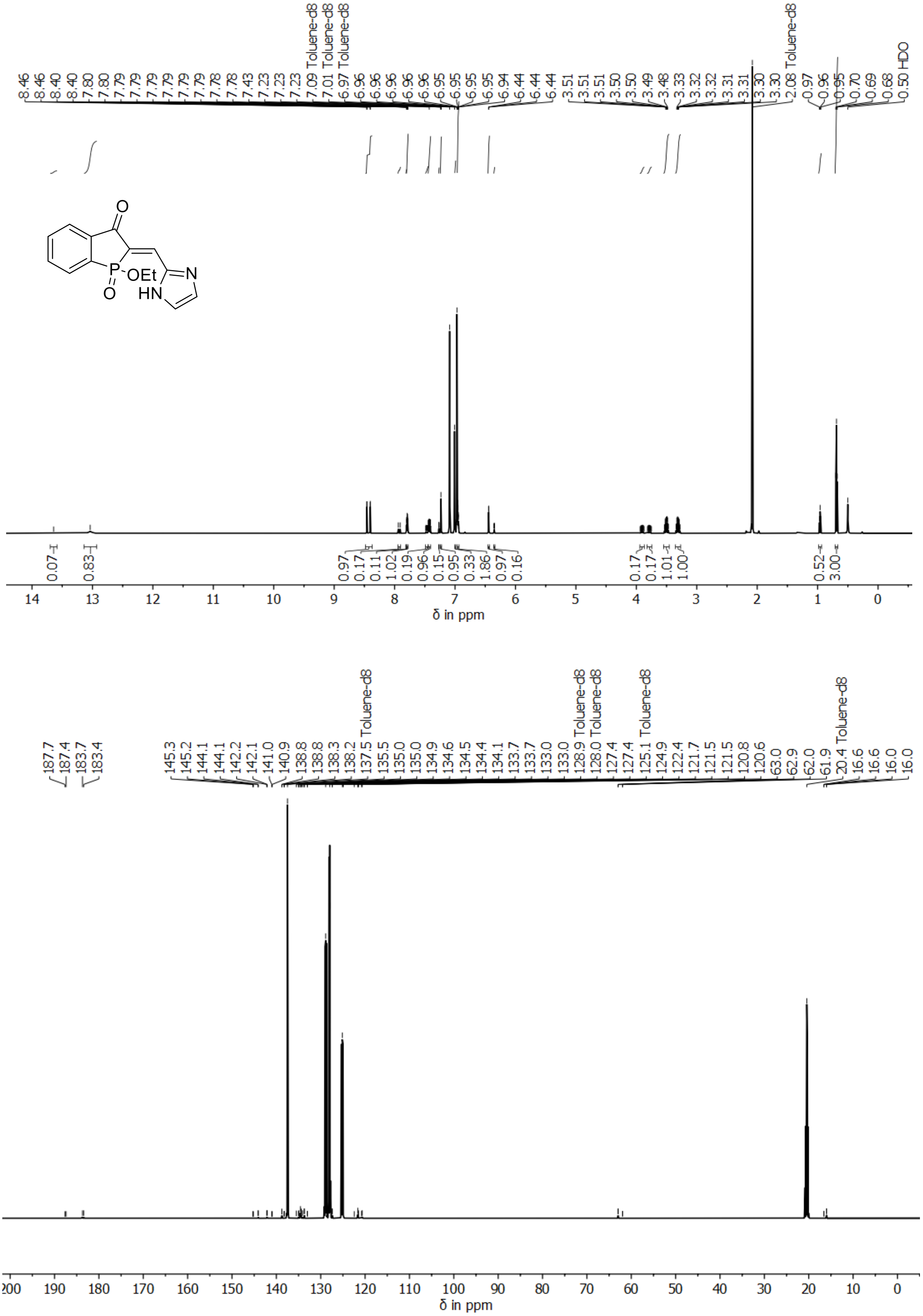

Supplementary Figure 189. NMR spectra of **15** in toluene-d_8 at 25 °C. (top) 600 MHz ^{1}H NMR spectrum. (bottom) 151 MHz ^{13}C NMR spectrum.

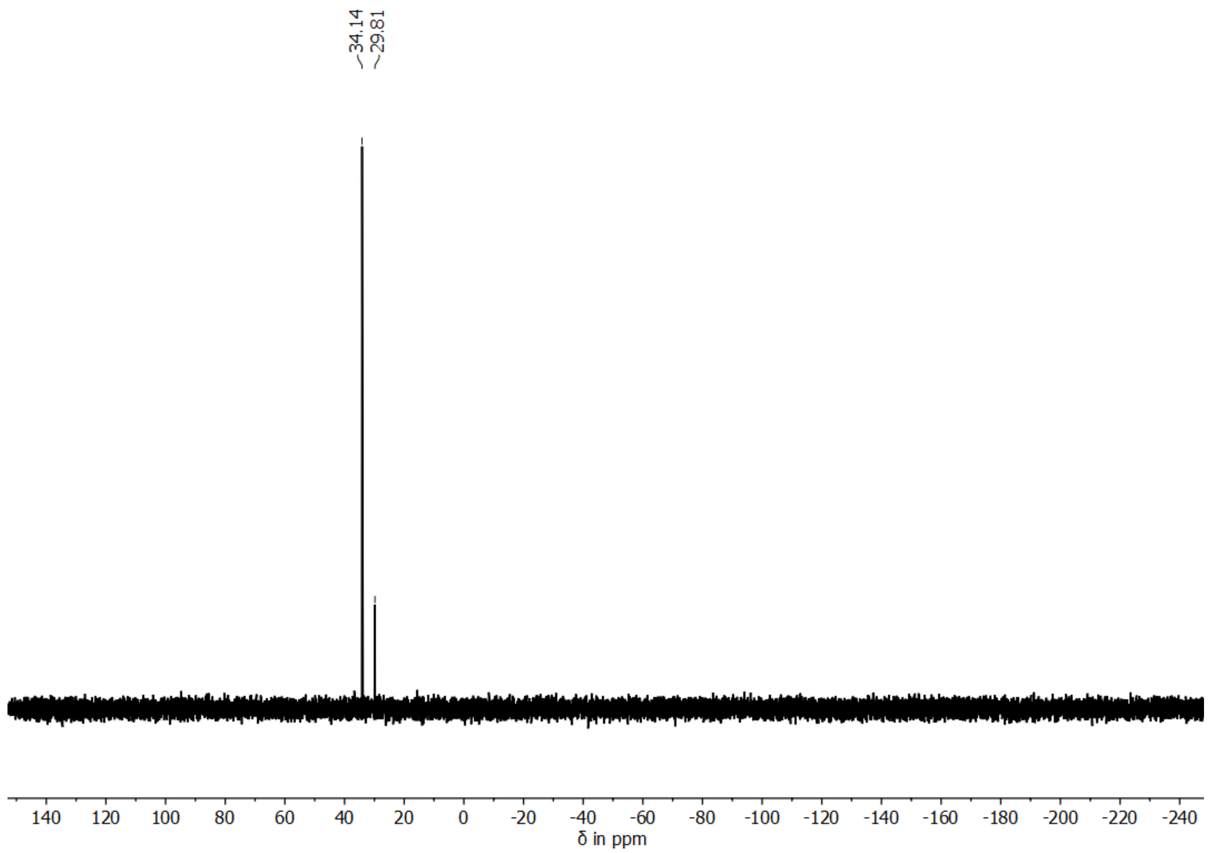

Supplementary Figure 190. 162 MHz ^{31}P NMR spectrum of **15** in toluene-d_8 at 25 °C.

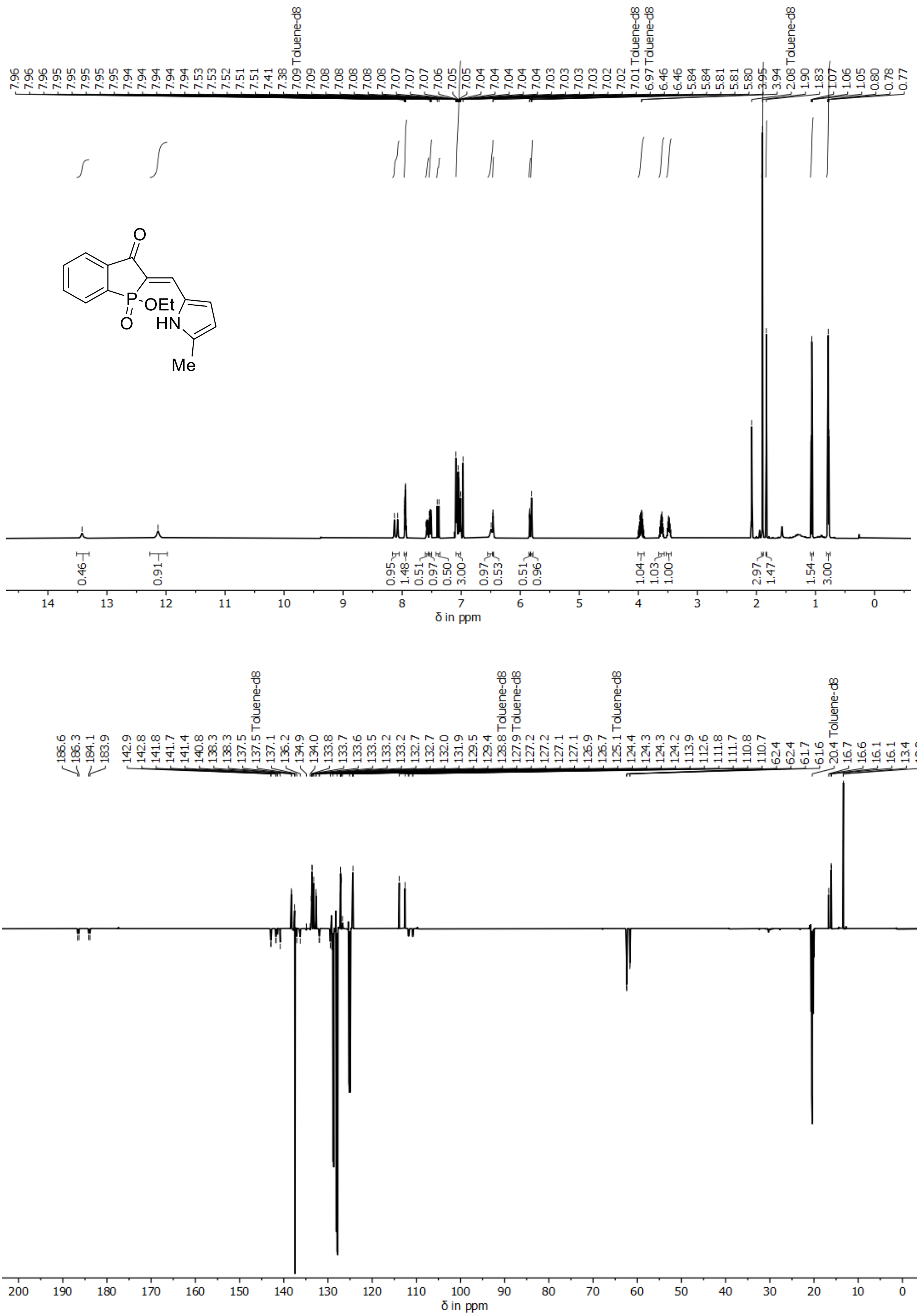

Supplementary Figure 191. NMR spectra of **16** in toluene-d_8 at 25 °C. (top) 600 MHz ^{1}H NMR spectrum. (bottom) 151 MHz DEPTq spectrum.

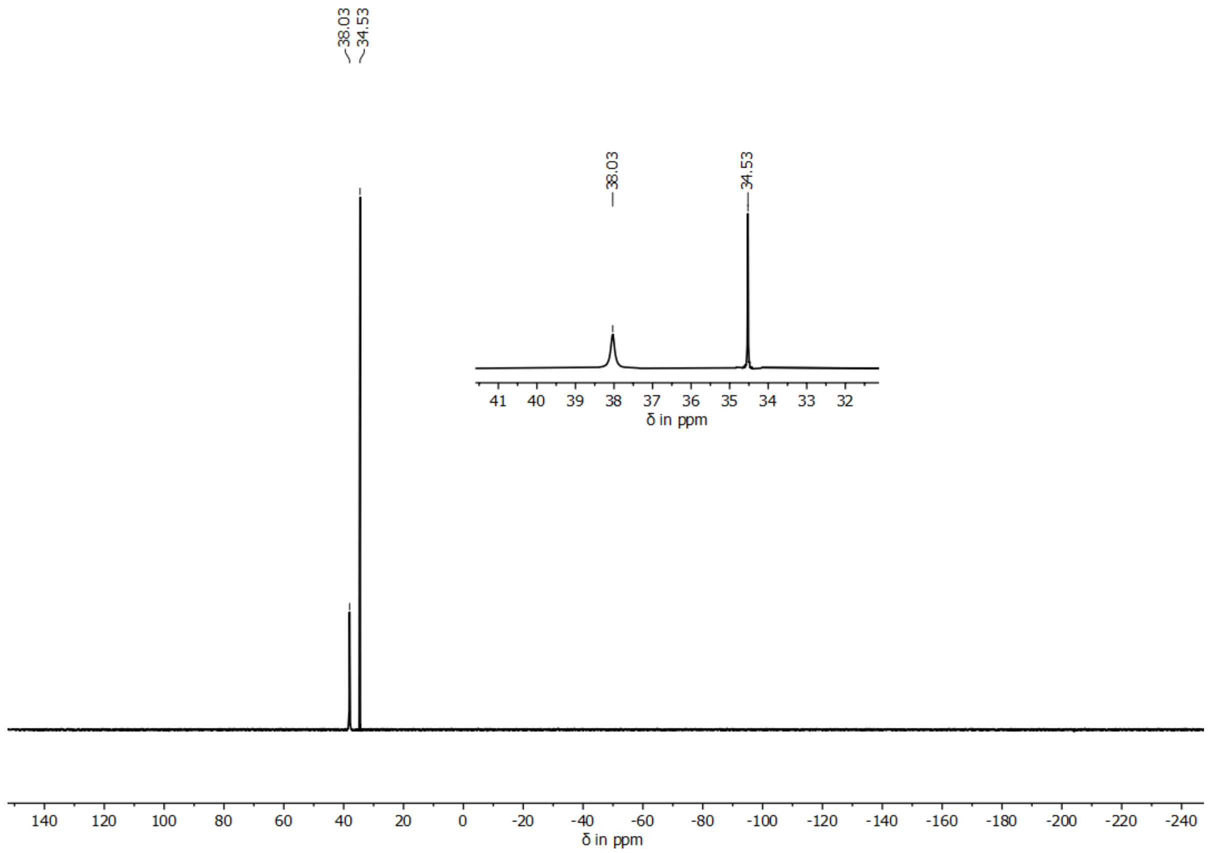

Supplementary Figure 192. 202 MHz ^{31}P NMR spectrum of **16** in toluene-d_8 at 25 °C.

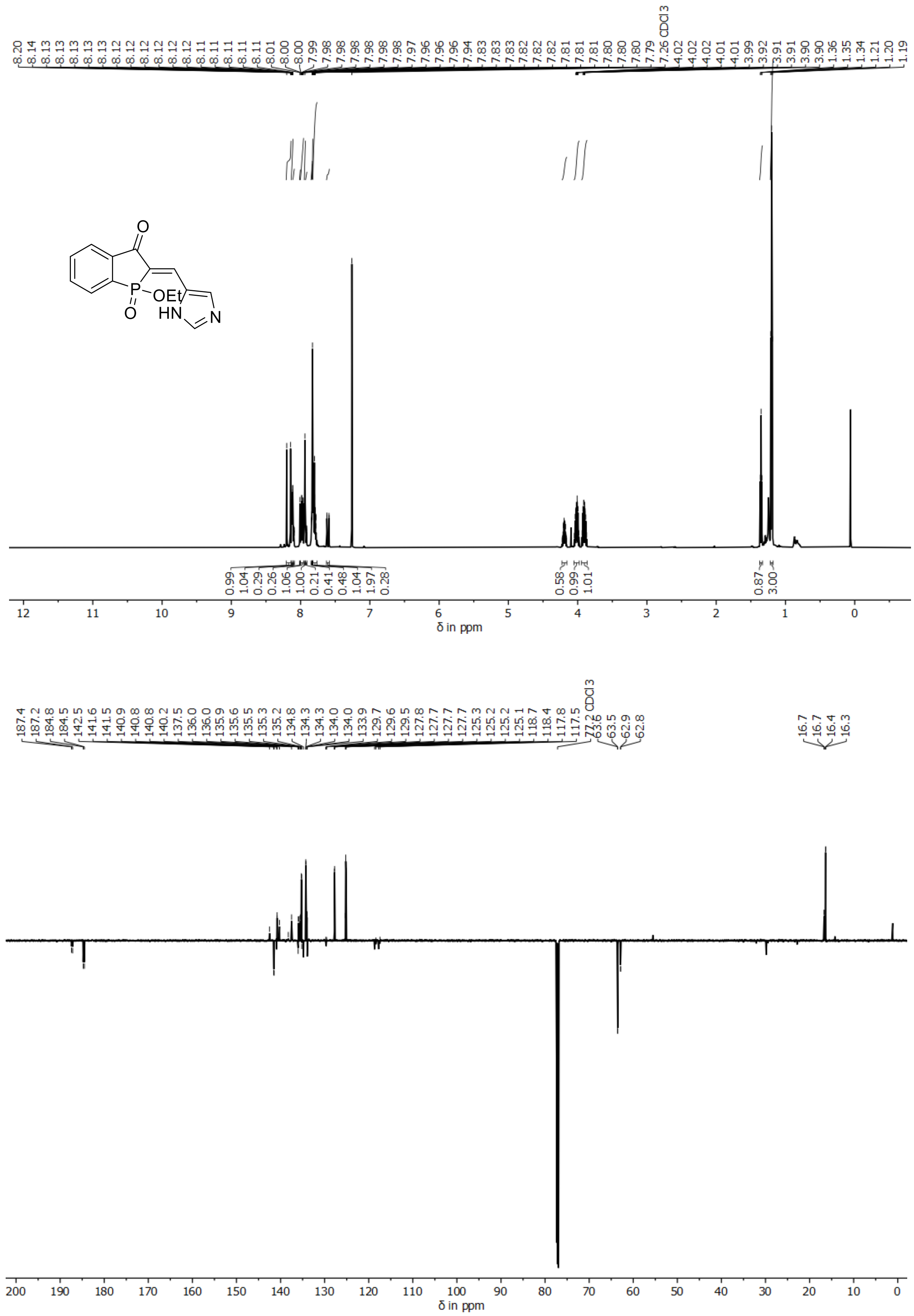

Supplementary Figure 193. NMR spectra of **17** in $CDCl_3$ at 25 °C. (top) 600 MHz ^1H NMR spectrum. (bottom) 151 MHz DEPTq spectrum.

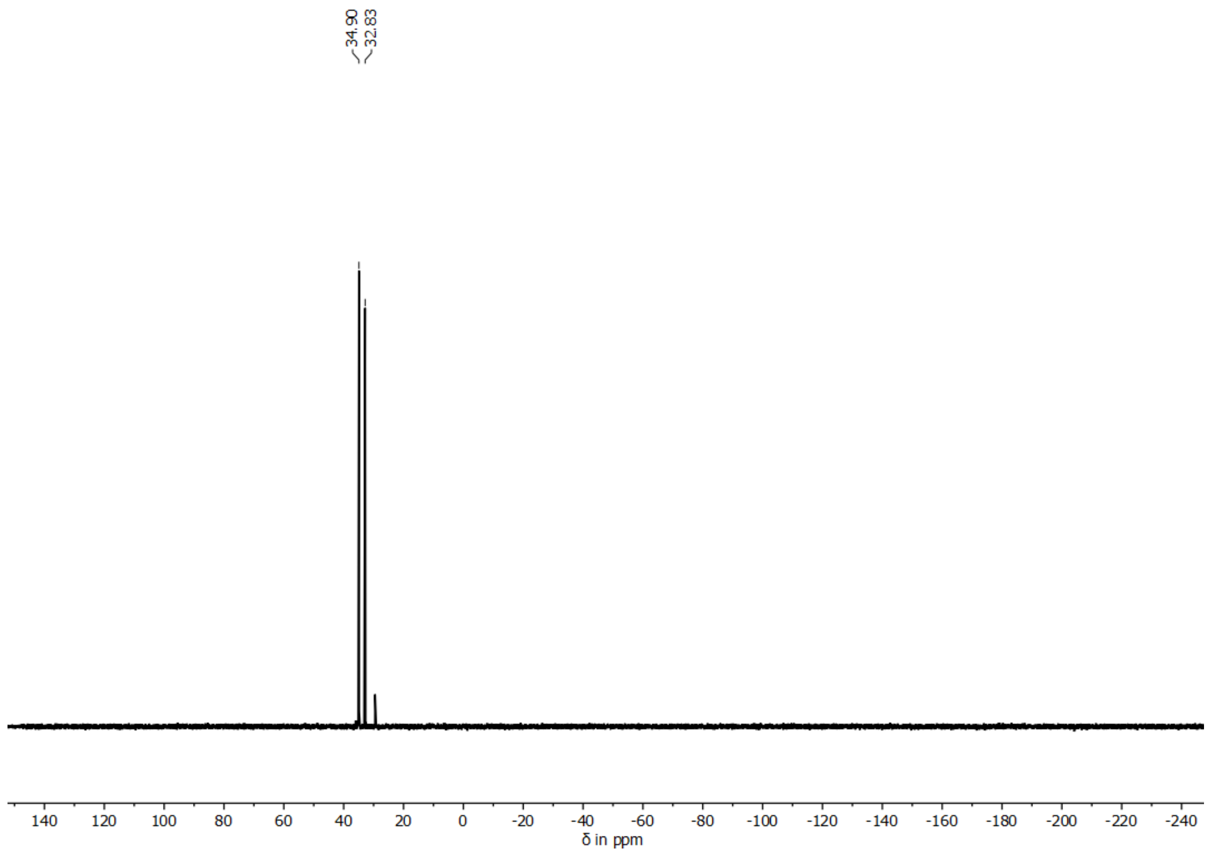

Supplementary Figure 194. 202 MHz ^{31}P NMR spectrum of **17** in $CDCl_3$ at 25 °C.

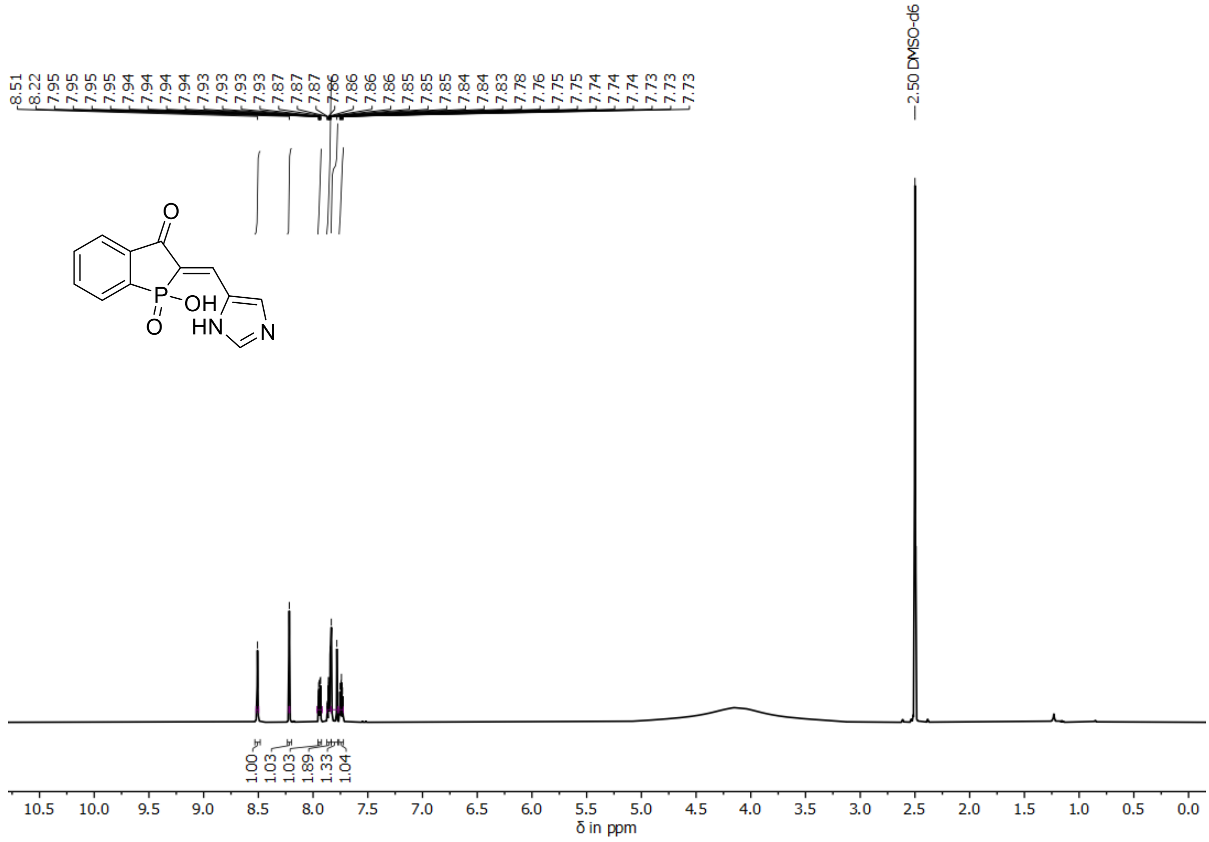

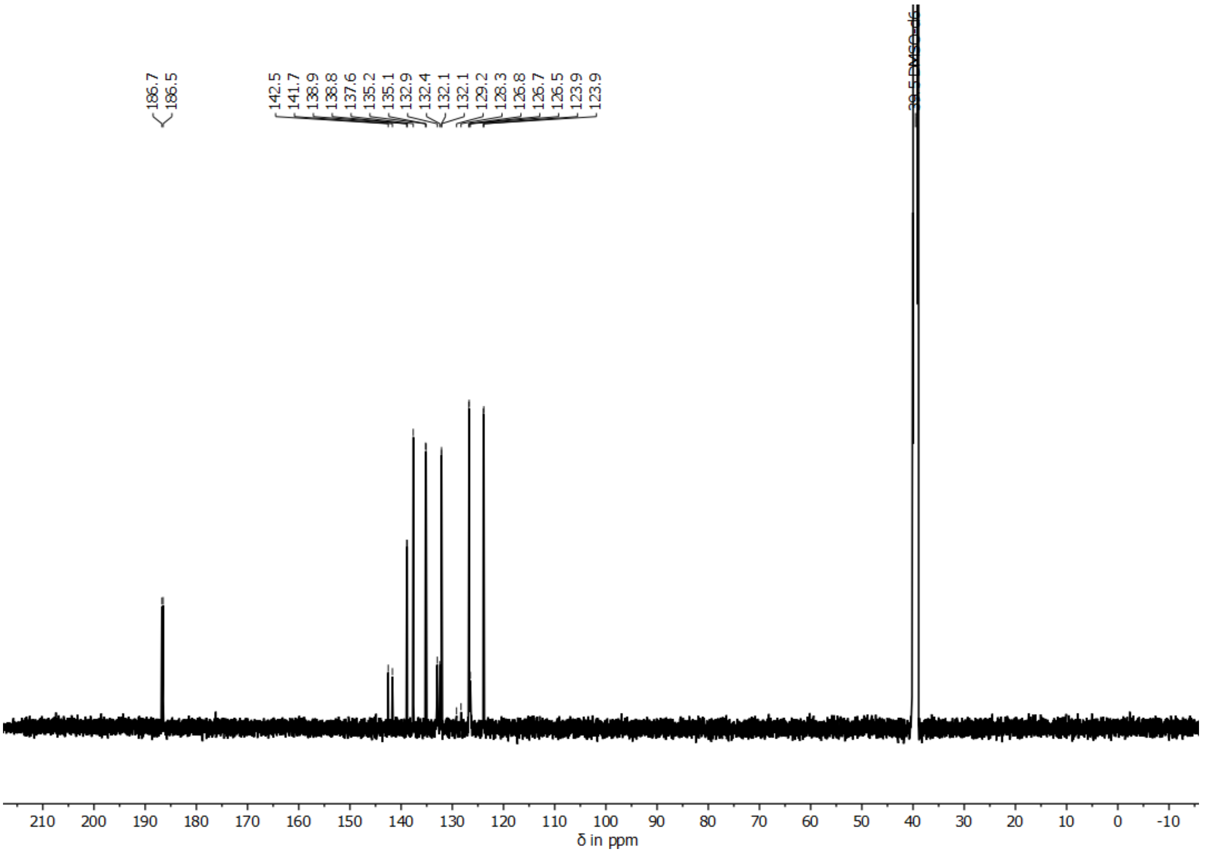

Supplementary Figure 195. NMR spectra of **17-**OH in DMSO-d_6 at 25 °C. (top) 600 MHz ^{1}H NMR spectrum. (bottom) 151 MHz ^{13}C NMR spectrum.

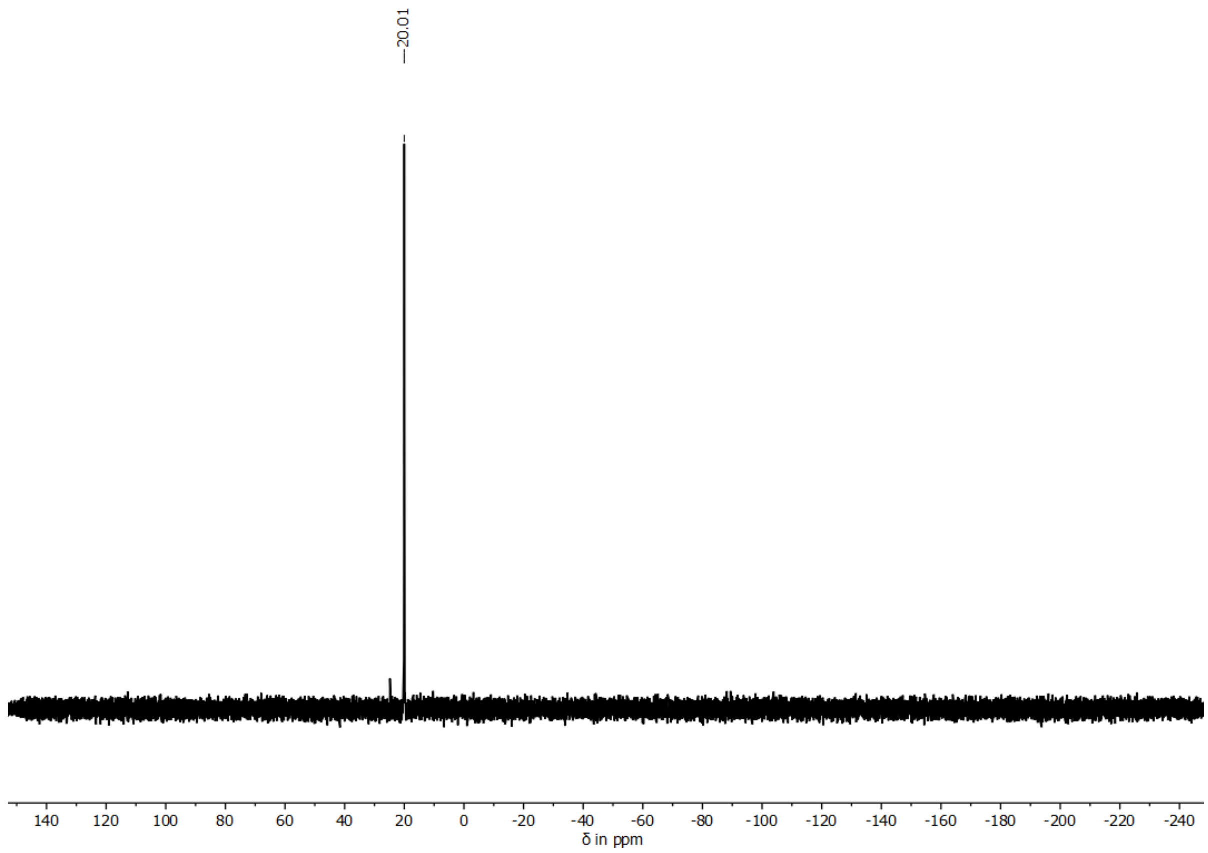

Supplementary Figure 196. 162 MHz ^{31}P NMR spectrum of **17**-OH in DMSO-d_6 at 25 °C.

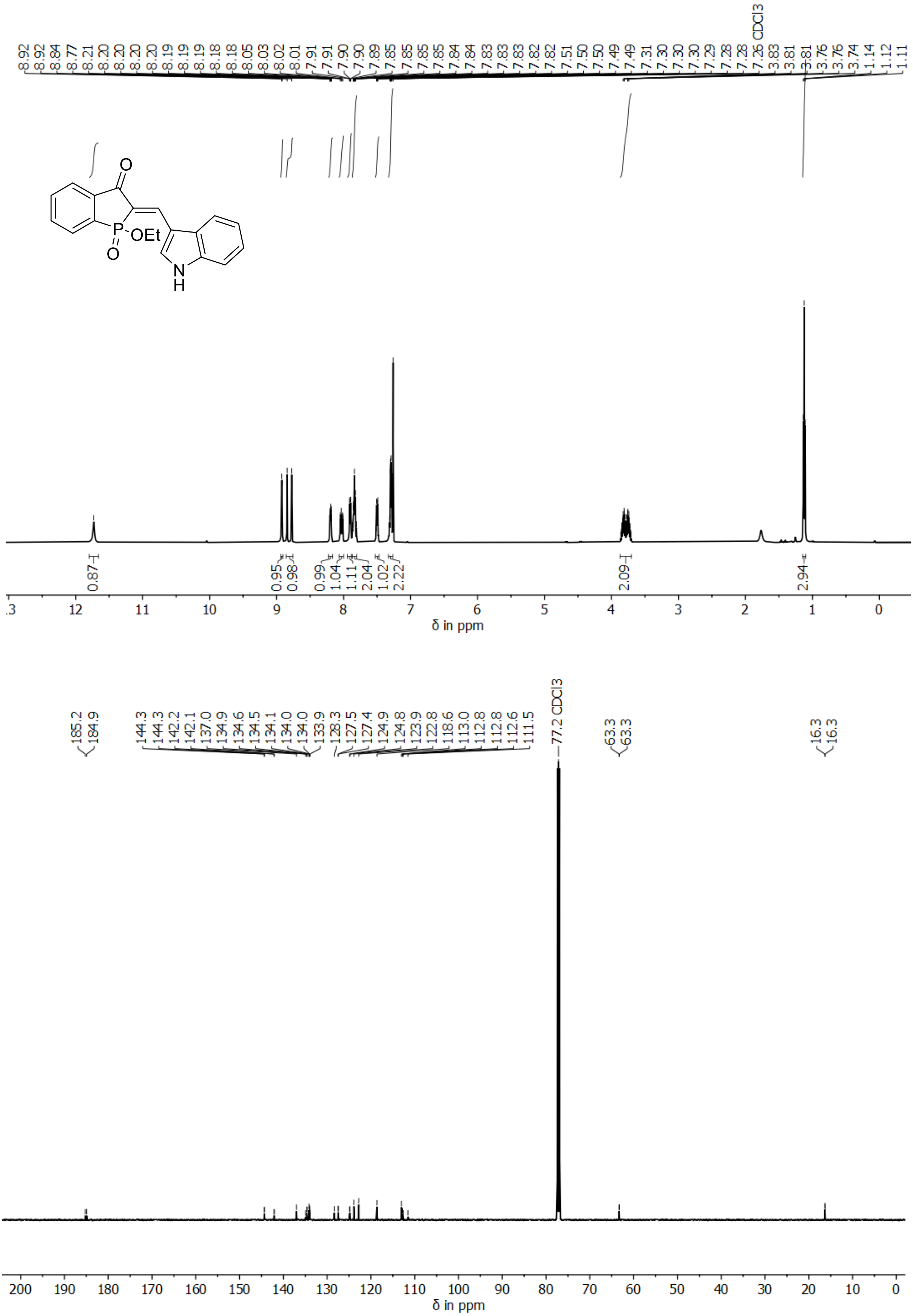

Supplementary Figure 197. NMR spectra of **18** in $CDCl_3$ at 25 °C. (top) 500 MHz ^{1}H NMR spectrum. (bottom) 126 MHz ^{13}C NMR spectrum.

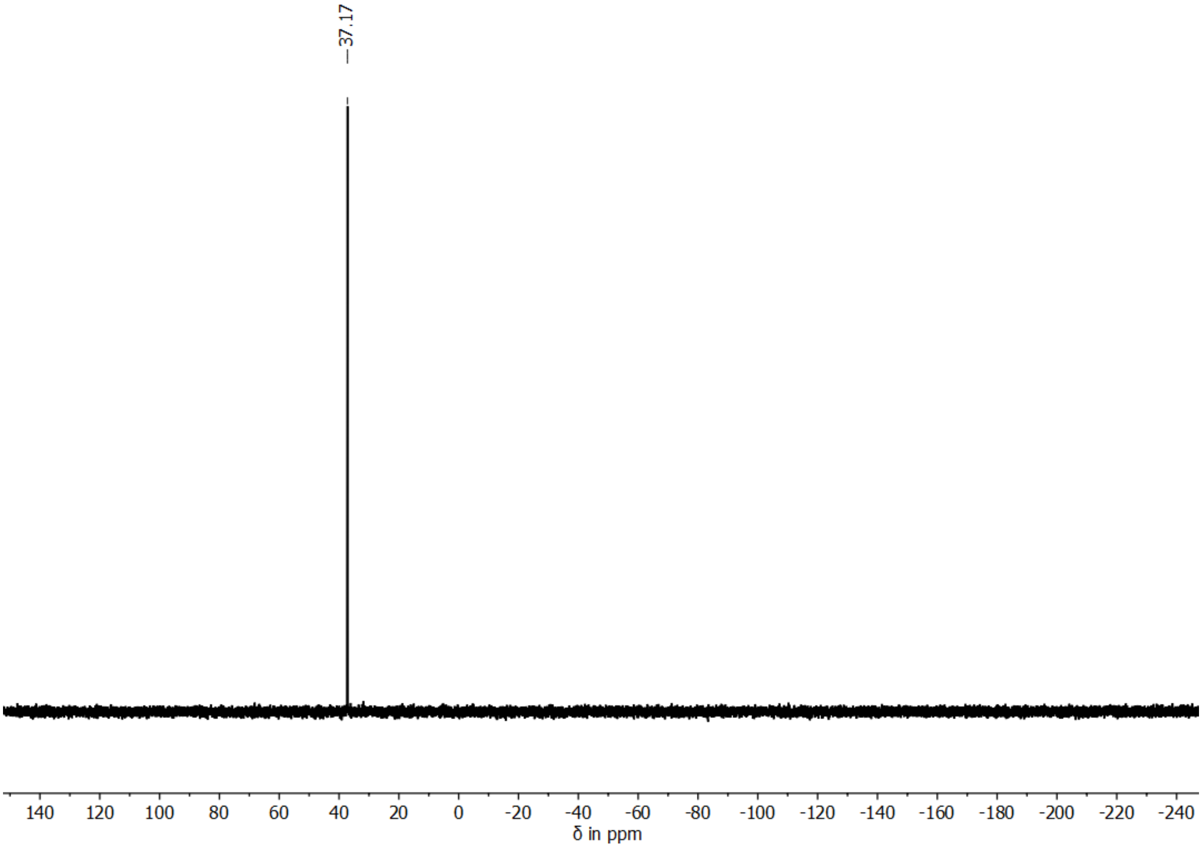

Supplementary Figure 198. 203 MHz ^{31}P NMR spectrum of **18** in $CDCl_3$ at 25 °C.

Supplementary Note 16: Crystal structure analysis

HPI **1**

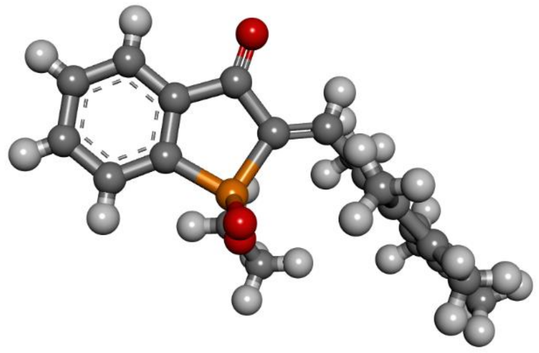

HPI **2**

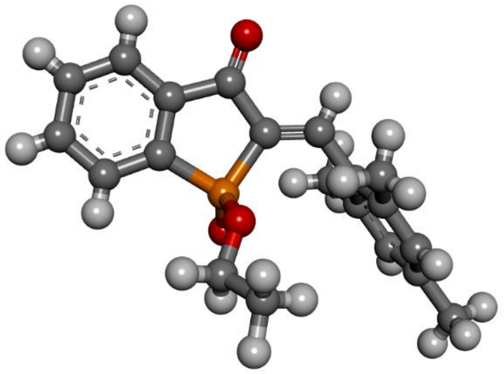

HPI **3**

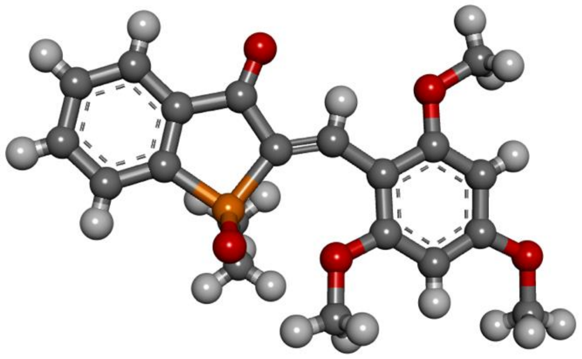

HPI **4**

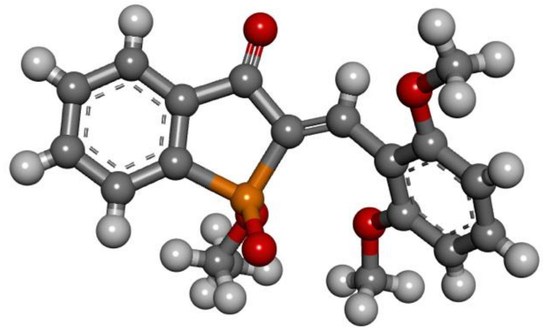

HPI **5**

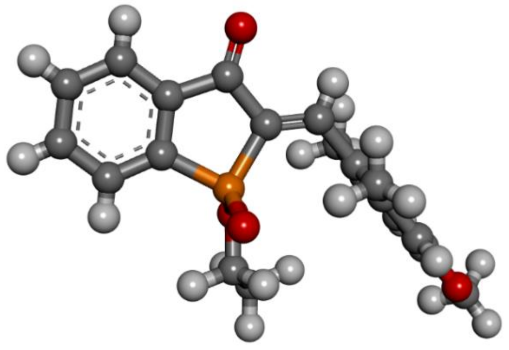

HPI **6**

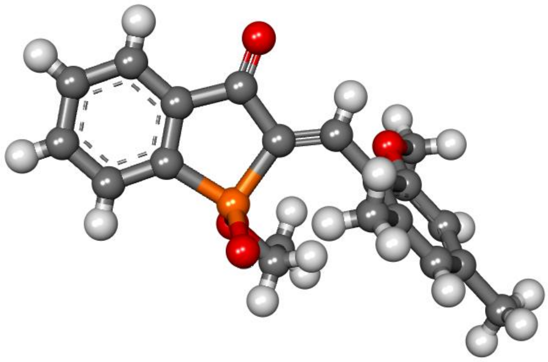

HPI **8**

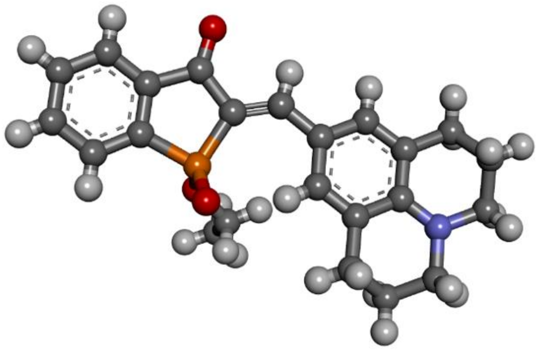

HPI **9**

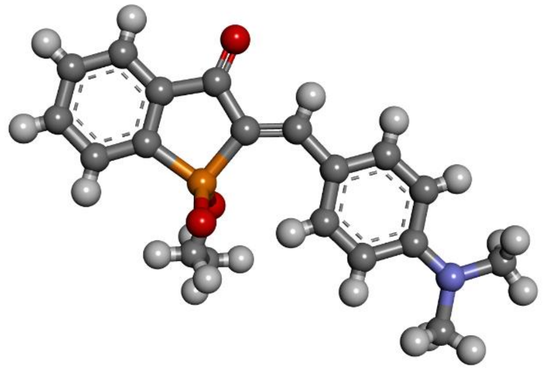

HPI **10** ((*R*)-enantiomer, HPLC E1)

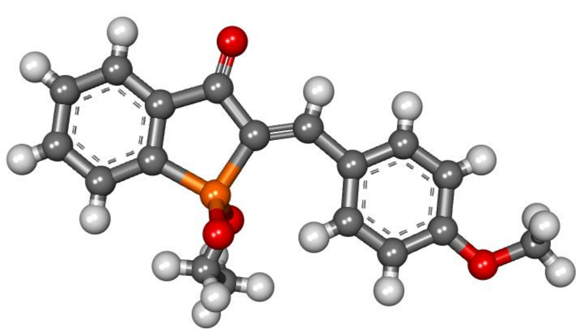

HPI **10** ((*S*)-enantiomer, HPLC E2)

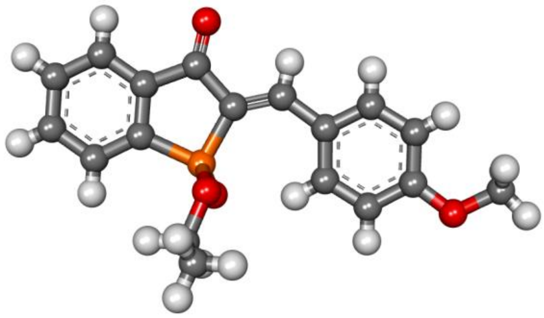

HPI **12**

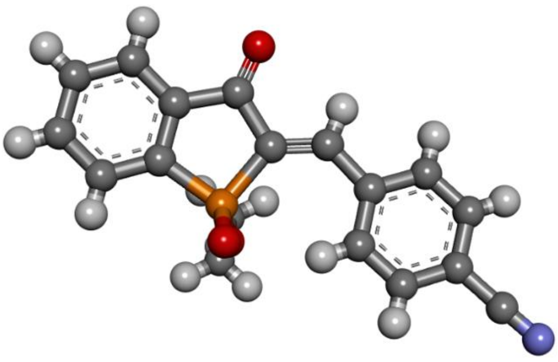

HPI **13**

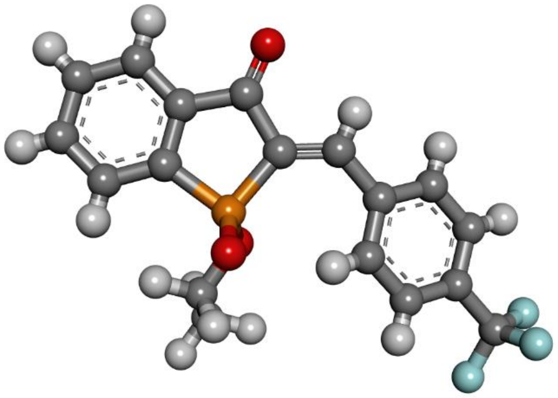

Supplementary Table 13. Crystal structure analysis of HPI **1** and **2**.

Compound	HPI 1 (22Dub_FK05 CCDC 2309092)	HPI 2 (22Dub_FK02 CCDC 2309091)
Diffractometer	SuperNova, Atlas	SuperNova, Atlas
Formula	$C_{22}H_{25}O_3P$	$C_{20}H_{21}O_3P$
$D_{calc.}$/ g cm^{-3}	1.316	1.268
m/mm^{-1}	1.460	1.481
Formula Weight	368.39	340.34
Colour	clear yellowish colourless	clear light colourless
Shape	block-shaped	block-shaped
Size/mm^3	0.22×0.19×0.07	0.19×0.19×0.09
T/K	153.00(10)	153.00(10)
Crystal System	orthorhombic	orthorhombic
Space Group	*Pbca*	*Pbca*
a/Å	11.98150(10)	18.9317(3)
b/Å	16.5507(2)	9.4621(2)
c/Å	18.7511(2)	19.9122(3)
a/°	90	90
b/°	90	90
g/°	90	90
V/Å^3	3718.38(7)	3566.95(11)
Z	8	8
Z'	1	1
Wavelength/Å	1.54184	1.54184
Radiation type	Cu K_α	Cu K_α
Q_{min}/°	4.717	4.441
Q_{max}/°	72.528	71.601
Measured Refl.	11147	20929
Independent Refl.	3597	3457
Reflections with I > 2(I)	3109	2995
R_{int}	0.0280	0.0349
Parameters	242	230
Restraints	0	1
Largest Peak	0.259	0.222
Deepest Hole	-0.305	-0.282
GooF	1.041	1.082
wR_2 (all data)	0.1023	0.1082
wR_2	0.0959	0.1036
R_1 (all data)	0.0434	0.0469
R_1	0.0366	0.0401

Supplementary Table 14. Crystal structure analysis of HPI **3** and **4**.

Compound	HPI 3 (22Dub_FK_11_2 CCDC 2309093)	HPI 4 (22Dub_FK06 CCDC 2309094)
Diffractometer	SuperNova, Atlas	SuperNova, Atlas
Formula	$C_{20}H_{21}O_6P$	$C_{19}H_{19}O_5P$
$D_{calc.}$/ g cm^{-3}	1.393	1.408
m/mm^{-1}	1.624	1.685
Formula Weight	388.34	358.31
Colour	clear light colourless	clear light colourless
Shape	block-shaped	block-shaped
Size/mm^3	0.21×0.12×0.05	0.26×0.19×0.14
T/K	153.00(10)	153.00(10)
Crystal System	monoclinic	triclinic
Space Group	$P2_1/c$	P-1
a/Å	11.1805(2)	7.4947(3)
b/Å	9.22660(10)	8.6650(3)
c/Å	18.6978(3)	13.9362(4)
a/°	90	102.273(3)
b/°	106.267(2)	100.447(3)
g/°	90	100.846(3)
V/Å^3	1851.61(5)	845.14(5)
Z	4	2
Z'	1	1
Wavelength/Å	1.54184	1.54184
Radiation type	Cu K_α	Cu K_α
Q_{min}/°	4.119	3.335
Q_{max}/°	71.544	72.270
Measured Refl.	12806	13654
Independent Refl.	3566	3259
Reflections with I > 2(I)	3034	3030
R_{int}	0.0323	0.0239
Parameters	248	229
Restraints	0	0
Largest Peak	0.265	0.286
Deepest Hole	-0.363	-0.371
GooF	1.031	1.021
wR_2 (all data)	0.0866	0.0848
wR_2	0.0810	0.0827
R_1 (all data)	0.0419	0.0337
R_1	0.0324	0.0313

Supplementary Table 15. Crystal structure analysis of HPI **5** and **6**.

**Compound**	**HPI 5 (22Dub_FK12_2 CCDC 2309098)**	**HPI 6 (23Dub_FK01 CCDC 2309100)**
Diffractometer	SuperNova, Atlas	SuperNova, Atlas
Formula	$C_{20}H_{21}O_4P$	$C_{20}H_{21}O_4P$
$D_{calc.}$/ g cm^{-3}	1.313	1.333
m/mm^{-1}	1.533	1.556
Formula Weight	356.34	356.34
Colour	clear light yellow	clear light colourless
Shape	block-shaped	block-shaped
Size/mm^3	0.21×0.11×0.08	0.34×0.28×0.20
T/K	153.00(10)	156(6)
Crystal System	triclinic	monoclinic
Space Group	*P*-1	$P2_1/c$
a/Å	8.9542(4)	15.6058(3)
b/Å	9.8212(4)	8.03371(11)
c/Å	10.9107(5)	14.6249(3)
a/°	76.154(4)	90
b/°	75.493(4)	104.4827(16)
g/°	88.912(3)	90
V/Å^3	901.06(7)	1775.30(5)
Z	2	4
Z'	1	1
Wavelength/Å	1.54184	1.54184
Radiation type	Cu K_α	Cu K_α
Q_{min}/°	4.314	6.204
Q_{max}/°	71.420	71.458
Measured Refl.	13907	11017
Independent Refl.	3433	3390
Reflections with I > 2(I)	2957	3087
R_{int}	0.0293	0.0302
Parameters	238	231
Restraints	2	0
Largest Peak	0.806	0.248
Deepest Hole	-0.346	-0.328
GooF	1.042	1.066
wR_2 (all data)	0.1411	0.0987
wR_2	0.1334	0.0961
R_1 (all data)	0.0565	0.0391
R_1	0.0485	0.0351

Supplementary Table 16. Crystal structure analysis of HPI **8** and **9**.

**Compound**	**HPI 8 (22Dub_FK08_2 CCDC 2309099)**	**HPI 9 (22Dub_FK03_2 CCDC 2309097)**
Diffractometer	SuperNova, Atlas	SuperNova, Atlas
Formula	$C_{23}H_{24}NO_3P$	$C_{19}H_{20}NO_3P$
$D_{calc.}$/ g cm^{-3}	1.377	1.356
m/mm^{-1}	1.485	1.599
Formula Weight	393.40	341.33
Colour	clear light red	clear orangish red
Shape	block-shaped	plate-shaped
Size/mm^3	0.16×0.15×0.07	0.24×0.20×0.08
T/K	153(50)	153.00(10)
Crystal System	triclinic	monoclinic
Space Group	*P*-1	$P2_1/c$
a/Å	8.6024(3)	14.2309(4)
b/Å	9.1880(3)	7.4782(2)
c/Å	13.3610(4)	15.7589(5)
a/°	97.531(3)	90
b/°	94.507(3)	94.307(3)
g/°	113.643(4)	90
V/Å^3	949.00(6)	1672.35(8)
Z	2	4
Z'	1	1
Wavelength/Å	1.54184	1.54184
Radiation type	Cu K_α	Cu K_α
Q_{min}/°	3.372	3.114
Q_{max}/°	71.406	71.591
Measured Refl.	13165	13767
Independent Refl.	3607	3228
Reflections with I > 2(I)	3152	2947
R_{int}	0.0453	0.0228
Parameters	254	221
Restraints	0	0
Largest Peak	0.456	0.491
Deepest Hole	-0.428	-0.312
GooF	1.130	1.058
wR_2 (all data)	0.1223	0.1163
wR_2	0.1168	0.1124
R_1 (all data)	0.0526	0.0425
R_1	0.0428	0.0394

Supplementary Table 17. Crystal structure analysis of HPI **10**.

**Compound**	**HPI (*R*)-10 (HPLC E1)** **(23Dub_FK02 CCDC 2309101)**	**HPI (*S*)-10 (HPLC E2)** **(23Dub_FK03 CCDC 2309102)**
Diffractometer	SuperNova, Atlas	SuperNova, Atlas
Formula	$C_{18}H_{17}O_4P$	$C_{18}H_{17}O_4P$
$D_{calc.}$/ g cm^{-3}	1.399	1.398
m/mm^{-1}	1.725	1.725
Formula Weight	328.29	328.29
Colour	clear greenish colourless	clear light colourless
Shape	block-shaped	plate-shaped
Size/mm^3	0.17×0.15×0.06	0.21×0.14×0.05
T/K	153(5)	153.1(4)
Crystal System	orthorhombic	orthorhombic
Flack Parameter	-0.020(16)	-0.011(18)
Hooft Parameter	-0.017(14)	-0.011(15)
Space Group	$P2_12_12_1$	$P2_12_12_1$
a/Å	7.28851(18)	7.28709(15)
b/Å	12.9517(3)	12.9541(2)
c/Å	16.5166(4)	16.5202(3)
a/°	90	90
b/°	90	90
g/°	90	90
V/Å^3	1559.15(6)	1559.48(5)
Z	4	4
Z'	1	1
Wavelength/Å	1.54184	1.54184
Radiation type	Cu K_α	Cu K_α
Q_{min}/°	4.338	4.337
Q_{max}/°	71.421	71.426
Measured Refl.	5357	5395
Independent Refl.	2951	2933
Reflections with I > 2(I)	2769	2727
R_{int}	0.0279	0.0258
Parameters	210	211
Restraints	0	0
Largest Peak	0.220	0.205
Deepest Hole	-0.247	-0.215
GooF	1.060	1.052
wR_2 (all data)	0.0874	0.0786
wR_2	0.0840	0.0763
R_1 (all data)	0.0374	0.0344
R_1	0.0338	0.0304

Supplementary Table 18. Crystal structure analysis of HPI **12** and **13**.

**Compound**	**HPI 12 (22Dub_FK01 CCDC 2309095)**	**HPI 13 (22Dub_FK04 CCDC 2309096)**
Diffractometer	SuperNova, Atlas	SuperNova, Atlas
Formula	$C_{18}H_{14}NO_3P$	$C_{18}H_{14}F_3O_3P$
$D_{calc.}$/ g cm^{-3}	1.391	1.474
m/mm^{-1}	1.709	1.917
Formula Weight	323.27	366.26
Colour	clear light colourless	clear light colourless
Shape	plate-shaped	block-shaped
Size/mm^3	0.23×0.15×0.04	0.29×0.17×0.06
T/K	153.1(4)	153(1)
Crystal System	triclinic	orthorhombic
Flack Parameter		0.50(4)
Hooft Parameter		0.017(15)
Space Group	*P*-1	$Pna2_1$
a/Å	7.0417(5)	20.1303(5)
b/Å	9.3897(7)	7.3467(2)
c/Å	12.2288(10)	22.3262(6)
a/°	73.554(7)	90
b/°	84.381(7)	90
g/°	87.280(6)	90
V/Å^3	771.59(11)	3301.85(15)
Z	2	8
Z'	1	2
Wavelength/Å	1.54184	1.54184
Radiation type	Cu K_α	Cu K_α
Q_{min}/°	3.784	3.960
Q_{max}/°	71.619	72.257
Measured Refl.	12277	7195
Independent Refl.	2949	4565
Reflections with I > 2(I)	2673	4228
R_{int}	0.0315	0.0198
Parameters	210	468
Restraints	0	19
Largest Peak	0.340	0.590
Deepest Hole	-0.308	-0.399
GooF	1.064	1.048
wR_2 (all data)	0.0986	0.1432
wR_2	0.0957	0.1377
R_1 (all data)	0.0399	0.0549
R_1	0.0352	0.0508

Supplementary Note 17: Calculated structures for heterocyclic derivatives

Since no crystal structure could be obtained for the heterocyclic derivatives HPI **15-18**, a geometry optimization on a PBE0/6-311++ G(d,p) level of theory with gd3bj dispersion correction was performed and the resulting optimized structures are shown below. These HPI are generally planar, with a slight tilt towards the double bonded oxygen of the phosphinate group due to hydrogen bonds or C-H/oxygen interaction.

HPI **15**

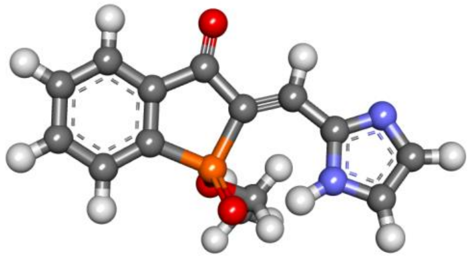

HPI **16**

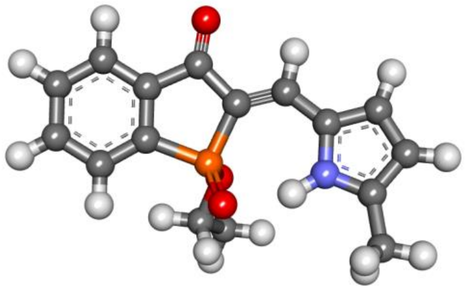

HPI **17**

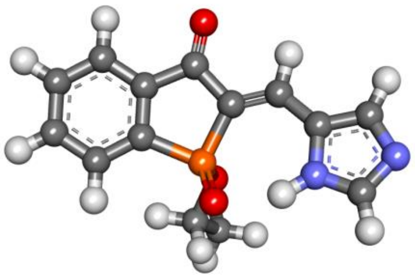

HPI **18**

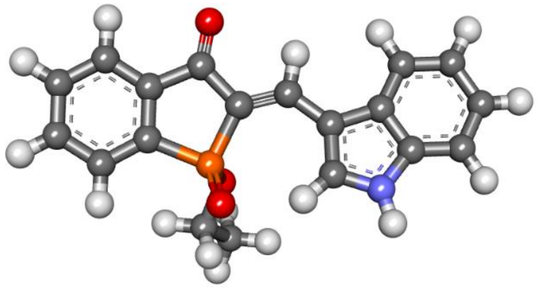

Supplementary Figure 199. Optimized structures for the heterocyclic derivatives HPI **15-18** on a PBE0/6-311++ G(d,p) level of theory.

Supplementary References

[1] J. A. Miles, R. C. Grabiak, C. Cummins, *J. Org. Chem.* **1982**, *47*, 1677-1682.

[2] T. M. Balthazor, *J. Org. Chem.* **1980**, *45*, 2519-2522.

[3] S. Wiedbrauk, B. Maerz, E. Samoylova, P. Mayer, W. Zinth, H. Dube *J. Phys. Chem. Lett.* **2017**, *8*, 1585-1592.

[4] S. Wiedbrauk, B. Maerz, E. Samoylova, A. Reiner, F. Trommer, P. Mayer, W. Zinth, H. Dube, *J. Am. Chem. Soc.* **2016**, *138*, 12219-12227.

[5] R. J. Mayer, N. Hampel, P. Mayer, A. R. Ofial, H. Mayr, *Eur. J. Org. Chem.* **2019**, *2019*, 412-421.

[6] G. Cai, N. Bozhkova, J. Odingo, N. Berova, K. Nakanishi, *J. Am. Chem. Soc.* **1993**, *115*, 7192-7198.

[7] B. Maerz, S. Wiedbrauk, S. Oesterling, E. Samoylova, A. Nenov, P. Mayer, R. de Vivie-Riedle, W. Zinth, H. Dube, *Chem. Eur. J.* **2014**, *20*, 13984-13992.

[8] L. Köttner, M. Schildhauer, S. Wiedbrauk, P. Mayer, H. Dube, *Chem. Eur. J.* **2020**, *26*, 10712-10718.

[9] C. Petermayer, S. Thumser, F. Kink, P. Mayer, H. Dube, *J. Am. Chem. Soc.* **2017**, *139*, 15060-15067.

[10] M. J. Frisch, G. W. Trucks, H. B. Schlegel, G. E. Scuseria, M. A. Robb, J. R. Cheeseman, G. Scalmani, V. Barone, G. A. Petersson, H. Nakatsuji, X. Li, M. Caricato, A. V. Marenich, J. Bloino, B. G. Janesko, R. Gomperts, B. Mennucci, H. P. Hratchian, J. V. Ortiz, A. F. Izmaylov, J. L. Sonnenberg, D. Williams-Young, F. Ding, F. Lipparini, F. Egidi, J. Goings, B. Peng, A. Petrone, T. Henderson, D. Ranasinghe, V. G. Zakrzewski, J. Gao, N. Rega, G. Zheng, W. Liang, M. Hada, M. Ehara, K. Toyota, R. Fukuda, J. Hasegawa, M. Ishida, T. Nakajima, Y. Honda, O. Kitao, H. Nakai, T. Vreven, K. Throssell, J. A. Montgomery, Jr., J. E. Peralta, F. Ogliaro, M. J. Bearpark, J. J. Heyd, E. N. Brothers, K. N. Kudin, V. N. Staroverov, T. A. Keith, R. Kobayashi, J. Normand, K. Raghavachari, A. P. Rendell, J. C. Burant, S. S. Iyengar, J. Tomasi, M. Cossi, J. M. Millam, M. Klene, C. Adamo, R. Cammi, J. W. Ochterski, R. L. Martin, K. Morokuma, O. Farkas, J. B. Foresman, and D. J. Fox, Gaussian, Inc., Wallingford CT, **2016**.

[11] S. Schrödinger Release 2018-3: MacroModel, Schrödinger, LLC, New York, NY, **2018**.

[12] T. Bruhn, A. Schaumlöffel, Y. Hemberger. G. Pescitelli, SpecDis, Version 1.70.1, Berlin, Germany, 2017, https://specdis-software.jimdo.com. T. Bruhn, A. Schaumlöffel, Y. Hemberger, G. Bringmann, *Chirality* **2013**, *25*, 243-249. T. Bruhn, G. Pescitelli, *Chirality* **2016**, *28*, 466-474.

[13] L. Köttner, H. Dube, *Angew. Chem. Int. Ed.* **2024**, *63*, e202409214.

[14] M. Karplus, *J. Am. Chem. Soc.* **1963**, *85*, 2870-2871.

[15] M. Balci, *Spin-spin splitting in 1H-NMR spectra. Basic 1H-and 13C-NMR spectroscopy*, Elsevier, Amsterdam, **2005**.

[16] S. Wiedbrauk, B. Maerz, E. Samoylova, A. Reiner, F. Trommer, P. Mayer, W. Zinth, H. Dube, *J. Am. Chem. Soc.* **2019**, *138*, 12219-12227.

[17] K. Hoffmann, M. Guentner, P. Mayer, H. Dube, *Org. Chem. Front.* **2019**, *6*, 1244-1252.

[18] H. Volfova, Q. Hu, E. Riedle, *EPA Newsletter* **2019**, *June*, 51-68.
