# Supplementary material for: Hemiphosphoindigos as a platform for chiroptical or water soluble photoswitching
Source: Nat Commun. 2025 Feb 19;16:1760. doi: 10.1038/s41467-025-56942-3 (PMC11840110; doi:10.1038/s41467-025-56942-3)
Supplement: Supplementary file 2 — Description of Additional Supplementary Files [file 41467_2025_56942_MOESM2_ESM.pdf]

Description of Additional Supplementary Files

File name: Supplementary Data 1
Description: xyz coordinates of theoretically optimized structures
